# Periodontal Disease Burden in China and G20 Nations: Insights From the Global Burden of Disease 2023 Study

**DOI:** 10.1016/j.identj.2026.109655

**Published:** 2026-05-29

**Authors:** Yilin Zhang, Xin Xu, Xun Wang, Yuan Tang, Fei He, Jing Li

**Affiliations:** a School of Stomatology, Shandong Second Medical University, Weifang, Shandong Province, PR China; b Affiliated Hospital of Shandong Second Medical University, Weifang, Shandong Province, PR China; c Key Laboratory of Shanxi Province for Craniofacial Precision Medicine Research, College of Stomatology, Xi’an Jiaotong University, Xi’an, China; d School of Public Health, Shandong Second Medical University, Weifang, Shandong Province, PR China

**Keywords:** Disability-adjusted life-years, Periodontal Disease, G20 countries, public health

## Abstract

**Aims:**

This study aimed to compare the periodontal disease burden between China and G20 countries from 1990 to 2023, to identify temporal trends using joinpoint regression, to quantify the respective contributions of population growth, aging, and epidemiological changes through demographic decomposition analysis, and to forecast the future burden to 2033 using autoregressive integrated moving average (ARIMA) models.

**Methods:**

Leveraging the most recent Global Burden of Disease Study (GBD) 2023 data, we analysed incidence and disability-adjusted life years (DALY). Joinpoint regression characterized trends. Demographic decomposition analysis quantified contributions from population growth, aging, and epidemiological changes. Future burden was projected using ARIMA models.

**Results:**

The burden varied substantially across G20 nations. In 2023, China showed a moderately high burden with age-standardized incidence rate of 950.20 per 100,000 and DALY rate of 68.5 per 100,000, with marked male predominance (ratio ∼1.2:1) and peak in 45–59 years. Joinpoint analysis revealed an overall slight decline in China's incidence (AAPC = -0.13) but a notable increase during 2010–2014 (APC = 4.79). Decomposition analysis identified aging as the main driver of increased incident cases in China (contributing 67.12% of 7.65 million increase), whereas population growth dominated in G20 countries (+120.87% contribution). ARIMA projections suggest China's DALY rates may stabilize around 64–65 per 100,000 by 2033, while G20 rates remain persistently higher at approximately 78 per 100,000.

**Conclusion:**

Periodontal disease burden in China is primarily driven by population aging, while population growth is the main factor in G20 countries. Tailored public health strategies are essential for each context.

**Clinical Relevance:**

From a public health perspective, these findings highlight need for targeted population-level strategies: In China, resource allocation should prioritize high-risk groups (e.g., middle-aged males and the elderly). In G20 countries, health planning should address persistent high DALY burden through integrated non-communicable disease prevention programs targeting shared risk factors.

## Introduction

Periodontal disease is a chronic inflammatory condition and a leading cause of adult tooth loss, also recognized as an independent risk factor for various systemic diseases.^1^ According to the Global Burden of Disease Study (GBD), severe periodontitis affected approximately 1.1 billion individuals globally in 2019 (prevalence 17%).^2^ As a non-communicable disease (NCD), it shares common risk factors with other NCDs, and its impact on systemic health, masticatory function, and quality of life is well-established.^3^

A comparative study between China and G20 countries holds significant public health relevance.^4^ The G20 represents major global economies, its disease burden mirroring broader health challenges.^5^ A focused comparative study between China and G20 countries holds significant and distinct public health relevance.^6^ The G20, comprising the world’s largest economies, represents a diverse yet strategically important group of nations that collectively account for over 80% of global GDP and approximately 64% of the world’s population.^7^ Its member states encompass a wide spectrum of socioeconomic development stages, demographic structures, and health system maturity. This makes it a highly pertinent macro-comparator for understanding how the burden of non-communicable diseases like periodontitis evolves in both advanced and transitioning economies, with direct implications for health policy within these influential nations.^8^ As the most populous G20 nation undergoing rapid transition, China's oral health profile combines global and local influences.^9^

Several studies have previously utilized historical GBD data to explore the global or regional distribution of periodontal diseases. For instance, Chen et al. systematically analysed the global burden of severe periodontitis from 1990 to 2019,^2^ while Wang et al. specifically evaluated the epidemiological features and trends of periodontal disease in China.^10^ Furthermore, the GBD 2021 Oral Disorders Collaborators provided a comprehensive assessment of the global burden of oral conditions, establishing a crucial foundation for subsequent research.^11^ Nevertheless, although a growing body already exists on periodontal disease burden globally and in China, systematic multinational comparative studies using the latest GBD 2023 data remain limited. The contribution of the present study lies in the use of the updated GBD 2023 dataset, the China-versus-G20 comparative framing, demographic decomposition analysis to quantify drivers, and short-term forecasting to 2033.

The rationale for this study is 3-fold. First, while individual GBD updates are published annually, systematic comparative analyses between China and the G20 using the 2023 data are lacking. Second, previous studies have not quantified the relative contributions of population growth, aging, and epidemiological changes to the changing burden of periodontal disease in these settings. Third, short-term forecasts to 2033—a relevant time horizon for health policy planning—are needed to anticipate future healthcare needs. Therefore, the specific aims of this study are to compare the periodontal disease burden between China and G20 countries from 1990 to 2023, to identify temporal trends and joinpoints using joinpoint regression, to quantify the demographic drivers through decomposition analysis, and to project the burden to 2033 using ARIMA modelling. The findings will inform international oral health collaboration and guide targeted public health strategies in China.

## Methods

### Data sources

This study is a large-scale descriptive epidemiological analysis based on the GBD 2023 dataset, constituting a specific sub-analysis focused on Periodontal Disease across G20 nations. Data were obtained from the GBD 2023 repository via the Global Health Data Exchange (GHDx; http://ghdx.healthdata.org), which provides comprehensive estimates of incidence, prevalence, DALYs, and their temporal trends for 375 diseases and injuries globally.^12^

Inclusion criteria for countries/entities: All 20 member entities of the G20 were included: the 19 sovereign nations (Argentina, Australia, Brazil, Canada, China, France, Germany, India, Indonesia, Italy, Japan, Mexico, Republic of Korea, Russia, Saudi Arabia, South Africa, Turkey, United Kingdom, United States) and the European Union (EU) as the 20th member.^13^ For the EU, we used the aggregate burden estimates provided by the GBD 2023 study for the EU region. Exclusion criteria: No entities were excluded. Inclusion criteria for years: The fulltime range 1990-2023 was included. Inclusion criteria for outcomes: Incidence and DALYs were included as the primary burden metrics. YLL was not analysed as it is negligible for periodontal disease in the GBD framework.

In the GBD 2023 framework, "periodontal disease" refers specifically to severe periodontitis. The reference case definition is: Community Periodontal Index of Treatment Needs (CPITN) Class IV, attachment loss (AL) > 6 gt; 6 mm, or gingival pocket depth (PD) > 5 gt; 5 mm. These definitions correspond to International Classification of Diseases, Ninth Revision (ICD-9) codes 523.0–523.9 and Tenth Revision (ICD-10) codes K05.0–K05.6. To maximize data inclusion, severe periodontitis cases identified using alternative criteria—such as CPITN Class III, AL > 5 gt; 5 mm, or AL > 4 mm—were harmonized with the reference definitions via the MR‑BRT meta‑regression approach, which accounts for and corrects systematic biases arising from varying case definitions. Importantly, gingivitis is explicitly excluded from this GBD entity; the burden estimates capture only chronic periodontitis with established attachment loss.^14^ Relationship to contemporary periodontal classifications: The GBD case definition captures severe periodontitis only, approximately equivalent to Stage III/IV periodontitis with clinical attachment loss ≥5 mm in the 2017 AAP/EFP classification.^15^ Unlike the AAP/EFP framework, the GBD does not distinguish between different stages of periodontitis (mild/moderate vs. severe) nor does it incorporate grading (progression rate). Potential bias from changing diagnostic criteria over time: The diagnostic criteria for periodontal disease have evolved substantially since 1990. The GBD 2023 study attempts to address this by applying a consistent case definition across all years and countries through its modelling framework, and by using MR‑BRT to harmonize data from different criteria. However, residual bias may persist, particularly in older data (1990–2000) where fewer studies used standardized criteria. Readers should interpret long‑term trends with this limitation in mind.

The "G20 average" reported throughout this manuscript refers to the population‑weighted average of all 20 G20 member entities (19 sovereign nations plus the European Union). All averages were calculated using population weights based on the GBD 2023 population estimates for each year, ensuring that larger populations (e.g., China, India, United States, EU) contribute proportionally more to the summary measure.^16^

To quantify the health burden, we evaluated the incidence and DALYs of Periodontal disease.^17^ Incidence was defined as the number of new cases per 100,000 population per year (i.e., an age‑standardized rate, not a proportion). DALYs, a composite metric calculated as the sum of Years Lived with Disability (YLD) and Years of Life Lost (YLL), offer a more comprehensive assessment of disease burden than incidence alone.^18^ For Periodontal Disease, YLL is considered negligible in the GBD framework; thus, DALYs in this analysis are equivalent to YLD.^19^ All age‑standardized rates (ASR) reported in this study were calculated using the direct method, with the GBD 2023 global standard population as the reference. The standard population age groups are: 0-4, 5-9, 10-14, …, 80-84, 85+ years. This standard population, developed by the GBD study, represents a hypothetical population with a stable age structure that facilitates comparisons across countries and over time. The formula used is: ASR = (Σ (age‑specific rate × standard population weight for that age group)) / Σ standard population weights.^20^

### Statistical analysis

This study is a descriptive epidemiological analysis. All analyses are descriptive in nature and are not intended to infer causal relationships. All GBD 2023 estimates are produced using DisMod‑MR 2.1, a Bayesian meta‑regression tool that generates point estimates with 95% uncertainty intervals (UI). In this study, we report point estimates throughout the main text and figures for clarity, but all comparative statements have been verified against the UI. Where UI overlap substantially, we refrain from making definitive claims of difference. All statistical analyses were performed using R software version 4.3.2 (R Foundation for Statistical Computing). Joinpoint regression was conducted using the ‘joinpoint’ package (version 0.1.0). Decomposition analysis was implemented using custom R scripts following the method described by Das Gupta. ARIMA modelling was performed using the ‘forecast' package (version 8.21).

Joinpoint regression was performed to characterize temporal trends in age‑standardized rates from 1990 to 2023. The method models trends by fitting a segmented log‑linear function, where the natural logarithm of the ASR is regressed against time. The slope within each segment is used to calculate the Annual Percent Change (APC). Potential joinpoints were identified through a systematic grid search, with the optimal number of segments (0–5) selected by minimizing the Bayesian Information Criterion (BIC). The final model was validated using Monte Carlo permutation testing with 4,499 permutations and a significance level of p<0.05. Heteroscedasticity was accounted for using the parametric method.^21^ To summarize the overall trend across the entire study period, the Average Annual Percent Change (AAPC)—a duration-weighted composite of the segment-specific APC—was computed along with its 95% confidence interval.^22^

The demographic decomposition analysis disaggregates the total change in incident cases and DALYs between 1990 and 2023 into 3 components: (1) population growth effect—attributable to changes in total population size, calculated by holding age-specific rates and age distribution constant at 1990 levels while allowing total population to change; (2) population aging effect—attributable to shifts in age distribution, calculated by holding age-specific rates constant while allowing the age structure to change; and (3) epidemiological change effect—attributable to changes in age‑specific incidence/DALY rates, calculated as the residual after accounting for population growth and aging.^23^ Important methodological note: The 'epidemiological change effect' is a residual component that captures the net effect of all factors not accounted for by population growth and aging. These include changes in age‑specific incidence/DALY rates due to alterations in risk factor prevalence (e.g., smoking, diabetes, oral hygiene), improvements in treatment and disease management, changes in disease classification or diagnostic practices, and any other time‑varying factors affecting disease occurrence.^24^ This component should not be interpreted as directly measuring any single causal mechanism. Percentage contributions were calculated as (component value / absolute total change) × 100. When a component has the opposite sign to the total change (e.g., epidemiological change negative while total change positive), its contribution is expressed as a negative percentage, indicating that it counteracted the overall increase. When the sum of absolute percentages exceeds 100%, this reflects the fact that components are not independent and some effects offset others.^25^

To project the future burden from 2024 to 2033, the ARIMA model was utilized.^26^ Model development followed a stepwise procedure. The time series of age‑standardized incidence and DALY rates were examined for stationarity, and where non‑stationary, appropriate differencing (d) was applied until stationarity was achieved. The Autocorrelation Function (ACF) and Partial Autocorrelation Function (PACF) plots of the stationary series were then examined to identify candidate values for the autoregressive order (p) and moving average order (q). Candidate models within the range p = 0–5, d = 0–2, q = 0–5 were fitted using maximum likelihood estimation, and the final model for each series was selected by minimizing both the AIC and Bayesian Information Criterion (BIC). Model diagnostics included the Ljung‑Box test for residual autocorrelation (*p* > .05 indicating adequate specification) and inspection of residual ACF/PACF plots for any remaining patterns. The best‑fitting ARIMA model for each series (reported in Supplementary Table S23) was then used to generate point forecasts with 95% prediction intervals for 2024‑2033. All statistical analyses were performed using R software version 4.3.2 with the forecast package; the auto.arima() function was used to automatically select the optimal model based on the corrected Akaike Information Criterion (AICc). Model fit was further evaluated using Mean Absolute Percentage Error (MAPE) and Root Mean Square Error (RMSE) on the training set.^27^ It is critically important to note that ARIMA models are purely statistical extrapolations based on historical temporal patterns (1990‑2023). They do not incorporate covariates such as future policy changes, technological advances in periodontal care, shifts in risk factor prevalence (e.g., smoking, diabetes), or structural disruptions like pandemics. Therefore, these projections should be interpreted as trend‑based illustrations of what would occur if historical patterns continue, not as deterministic predictions. All GBD 2023 estimates are produced using DisMod‑MR 2.1, a Bayesian meta‑regression tool that generates point estimates with 95% uncertainty intervals (UI). Throughout this manuscript, point estimates are presented for clarity, but all conclusions are based on modeled, not directly observed, data. Readers should interpret findings with awareness that 95% UI are available in the supplementary tables.

## Results

### The Burden of Periodontal Disease in G20 Countries and China's Profile

**Fig. 1:**
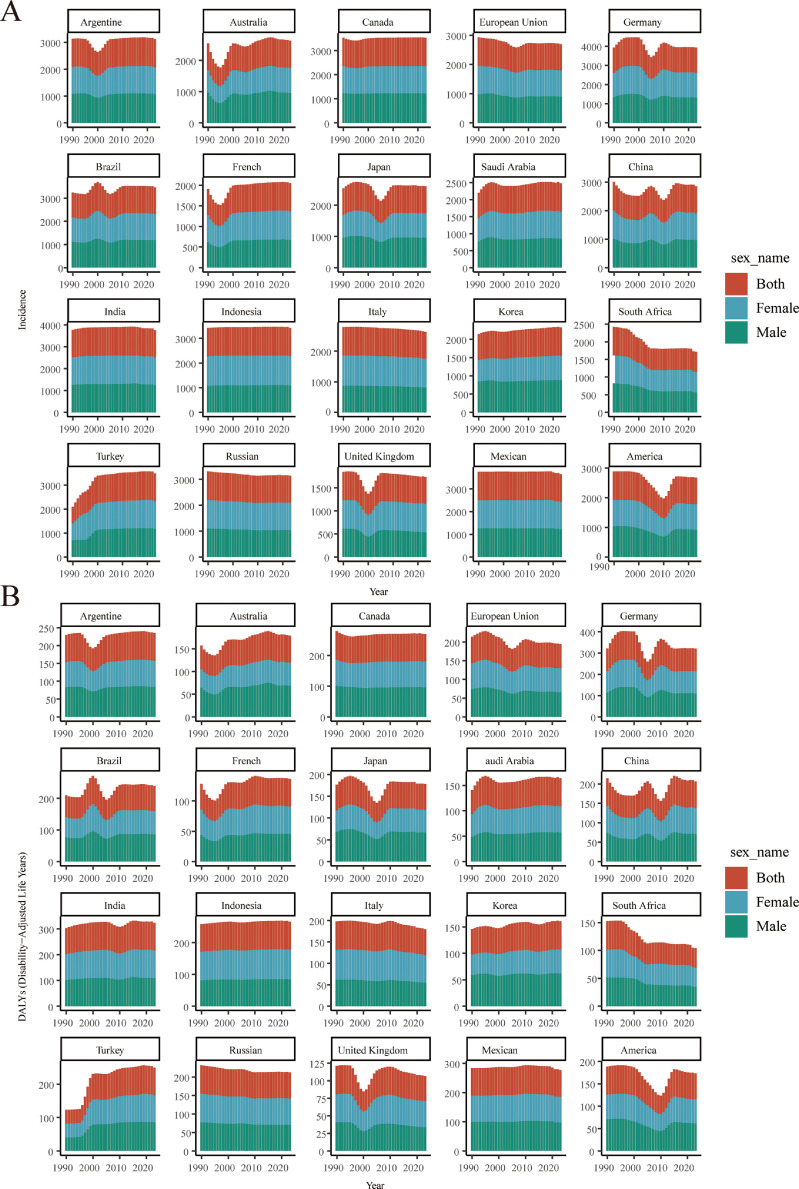
Time trends in the incidence and DALYs of periodontal diseases across the populations of G20 member countries, 1990-2023. (A) Age-standardized incidence rates. (B) Age-standardized DALY rates.

In 2023, Brazil (1157.38 per 100,000), Turkey (1164 per 100,000), and China (950.20 per 100,000) had the highest incidence rates, while France, the United Kingdom, and South Africa had the lowest (Table S1). However, the 95% UI for Brazil and Turkey overlap, indicating that the observed difference is not statistically significant. Similarly, China's rate falls within the overlapping range of several other countries. Japan, Italy, the United States, and the United Kingdom demonstrated long-term declining trends with non‑overlapping UIs between 1990 and 2023 (Figure 1A and B). For DALYs, Germany, Brazil, India, and Mexico carried the highest burdens (Germany peaking at ∼133.7 per 100,000 in 1999; Table S2). France, the United Kingdom, and South Africa maintained low DALY rates. Japan and some European nations improved, while Brazil's burden remained stable at high levels.

Age distribution of burden differed markedly between China and the G20 average. Males exhibited higher incidence and DALY rates than females, a disparity pronounced in Germany, Mexico, and Brazil. China's incidence rate was higher than Japan, Saudi Arabia, and France, but lower than Brazil. Its DALY rate (2023: 68-70 per 100,000) was higher than France and the United Kingdom, but lower than Brazil, India, and Mexico (Table S2). After a decline in the 1990s, China's DALY rate increased in the late 2010s and then showed little change.

### Age-specific patterns of periodontal disease burden in China and the G20

**Fig. 2:**
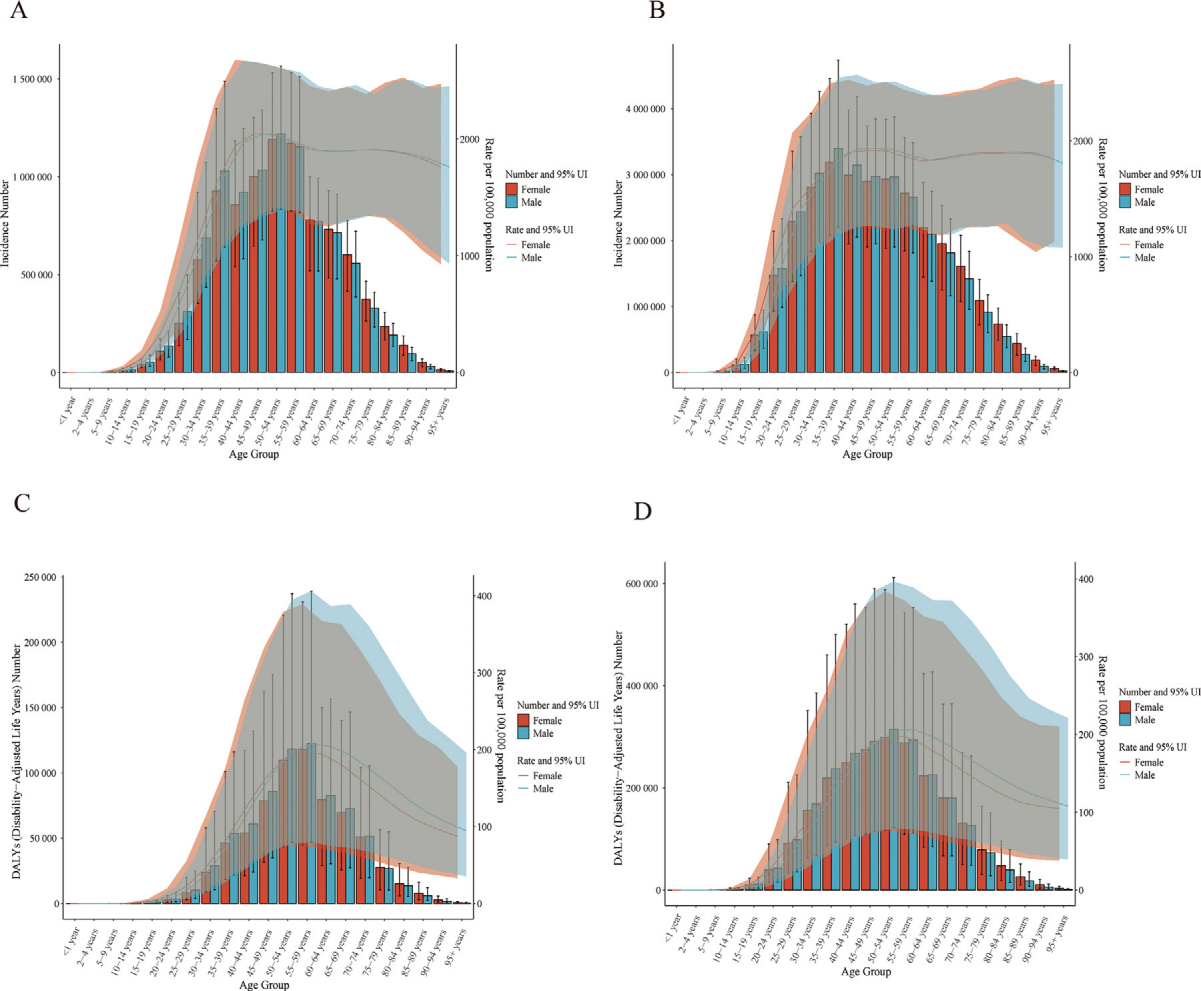
Age-specific incidence and DALY rates of periodontal diseases in China versus the G20 average, 1990-2023. (A) Incidence rates in China, (B) Incidence rates rates in G20, (C) DALYs rates in Chia, (D) DALYs rates in G20.

Significant differences existed in the age distribution of burden between China and the G20 average. In China, incidence increased sharply from adolescence, exceeding 1,000 per 100,000 in the 30–34 age group and peaking in the 45-49 age group, with a distinct male predominance (Figure 2A, Table S3). For the G20 average, the first incidence peak occurred earlier (25-29 years, 1410 per 100,000), and rates remained high (1800–1900 per 100,000) from age 35 onwards, with minimal gender disparity (Fig. 2B, Table S4).

For DALY rates in China, the peak occurred at 55–59 years, with male rates consistently higher than female rates across all ages (Figure 2C, Table S5). In the G20, the peak DALY burden was concentrated in the 50–59year age range (up to 206.78 per 100,000), followed by a decline in older groups. A male disadvantage existed but was less pronounced than in China (Fig. 2D, Table S6).

### Temporal trends in periodontal disease burden in China and G20 countries through joinpoint analysis

**Fig. 3:**
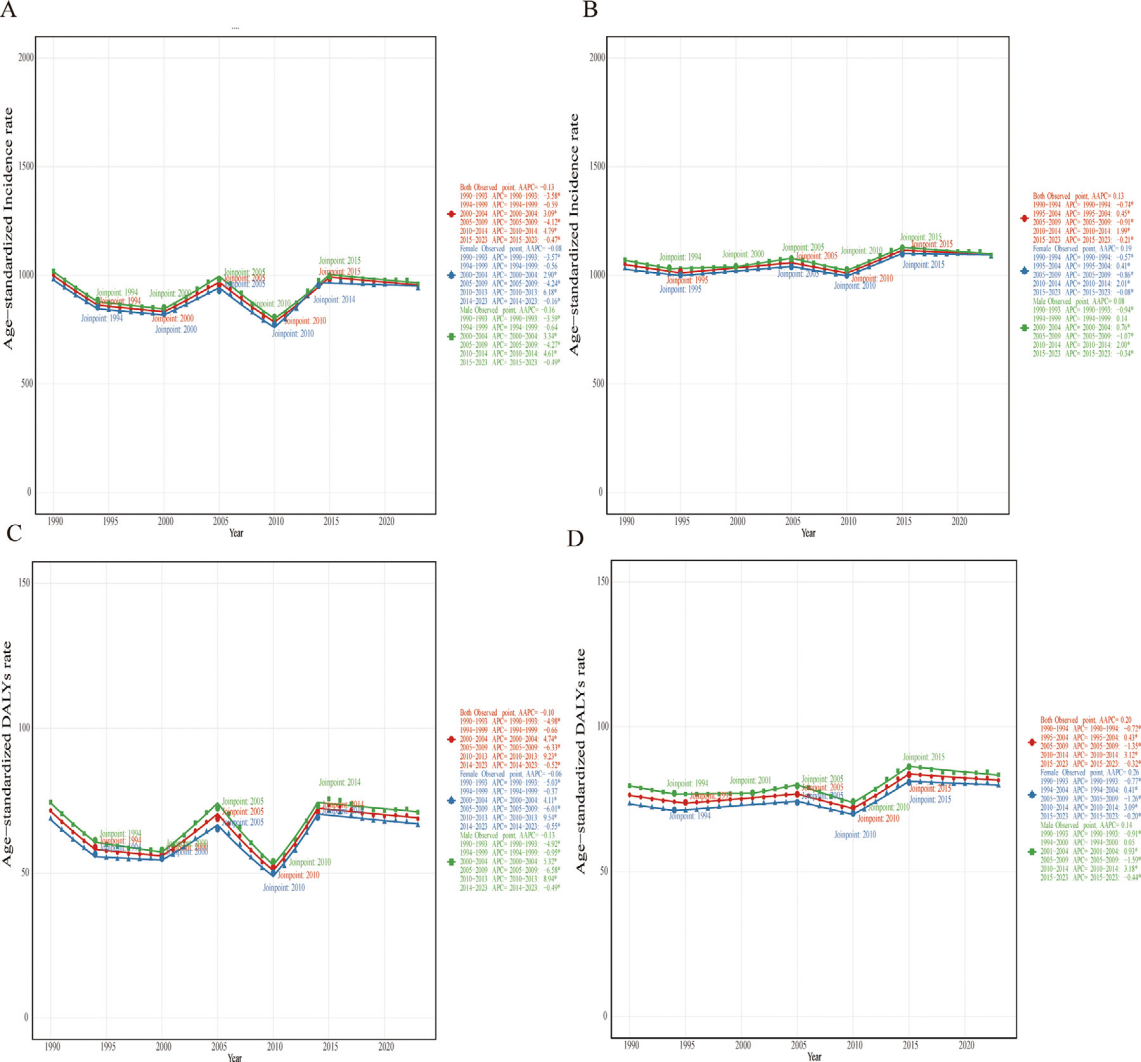
Joinpoint analysis of trends in periodontal diseases incidence and DALYs in China versus the G20 average, 1990–2023. (A) Incidence rates in China, (B) Incidence rates in G20, (C) DALYs rates in China, (D) DALYs rates in G20.

China experienced an overall slight decline in incidence (AAPC = -0.13), with a reduction during 2005–2009 (APC = -4.12) and an increase in 2010–2014 (APC = 4.79) (Figure 3A). G20 countries saw a mild overall increase (AAPC = 0.13), with a decline during 2005-2009 (APC = -0.91) and a rise in 2010–2014 (APC = 2.00) (Figure 3B). Females in China showed the highest incidence increase during 2010–2013 (APC = 6.18).

For DALY rates, China's overall trend showed a slight decline (AAPC = -0.10), with decreases during 1990-1993 (APC = -4.98) and 2005-2009 (APC = -6.33), and an increase during 2010-2013 (APC = 9.23) (Fig. 3C). G20 countries experienced a slight overall increase (AAPC = 0.20), with a decline in 2005–2009 (APC = -1.35) and a rise in 2010–2014 (APC = 3.12) (Figure 3D). Females in China had the highest DALY rate increase during 2010-2013 (APC = 9.54). An elevated trend from 2010 to 2014 was followed by a decline after 2015 in most groups.

### Decomposition analysis of periodontal disease burden in China and G20 countries

**Fig. 4:**
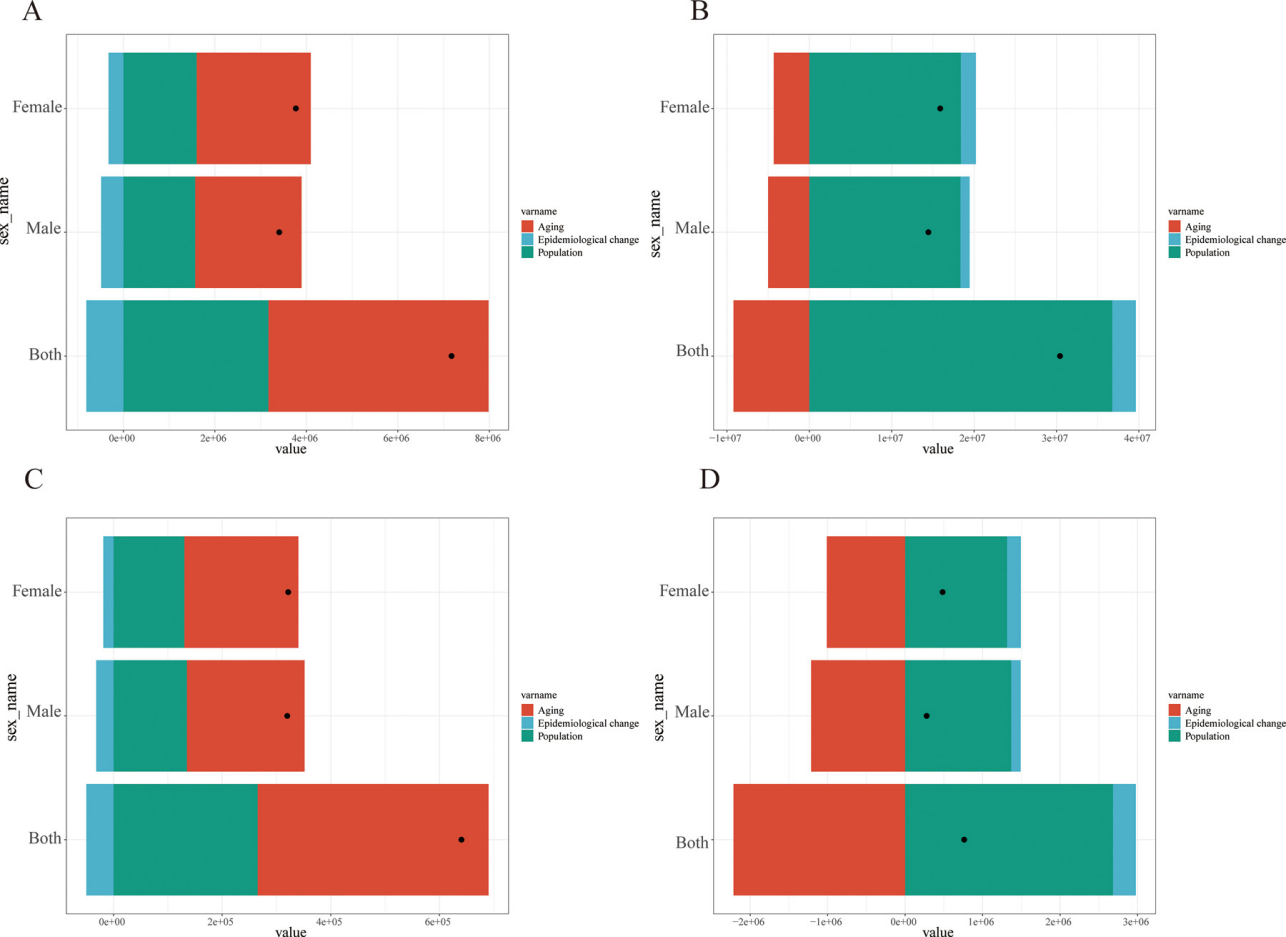
Decomposition analysis of trends in periodontal diseases incidence and DALYs in China versus the G20 average, 1990-2023. (A) Incidence rates in China, (B) Incidence rates in G20, (C) DALYs rates in China, (D) DALY rates in G20.

For the incidence in China, the total number of cases increased by 7.65 million. Aging was the primary contributing factor, accounting for 67.12% of the change, followed by population growth (44.18%). Epidemiological changes partially offset this increase (-11.30%) (Figure 4A and Table S7). In contrast, the G20 saw a 78.6% rise in total cases, driven predominantly by population growth (+120.87%), while aging exerted a substantial negative influence (-30.23%). This negative contribution of aging means that, holding other factors constant, the shift toward older age distributions in G20 countries between 1990 and 2023 would have been expected to reduce total case counts, because older age groups in the G20 have lower age-specific incidence rates than younger groups (as shown in Fig. 4, Fig. 4). However, this protective aging effect was completely overwhelmed by the massive increase in total population size. Epidemiological changes contributed a modest increase (+9.37%), with a stronger effect in females (+11.38%) than in males (+7.67%) (Figure 4B and Table S9).

Divergent drivers were also identified in the decomposition of DALY rates. In China, the increase in DALYs was primarily driven by aging (66.38%) and population growth (41.43%), which were partly counterbalanced by favourable epidemiological changes (-7.80%). Males showed greater improvement in epidemiological factors (-9.95%) compared to females (-5.79%) (Figure 4C and Table S8). Conversely, in the G20, the rise in DALYs was overwhelmingly driven by population growth (+351.69%), which completely offset the substantial protective effect of aging (-290.3%). Epidemiological changes also contributed to the increase (+38.62%), with a larger effect in females (+36.71%) than in males (+43.55%) (Figure 4D and Table S10).

### Projected Trends in periodontal disease burden in China and G20 countries based on ARIMA modelling

**Fig. 5:**
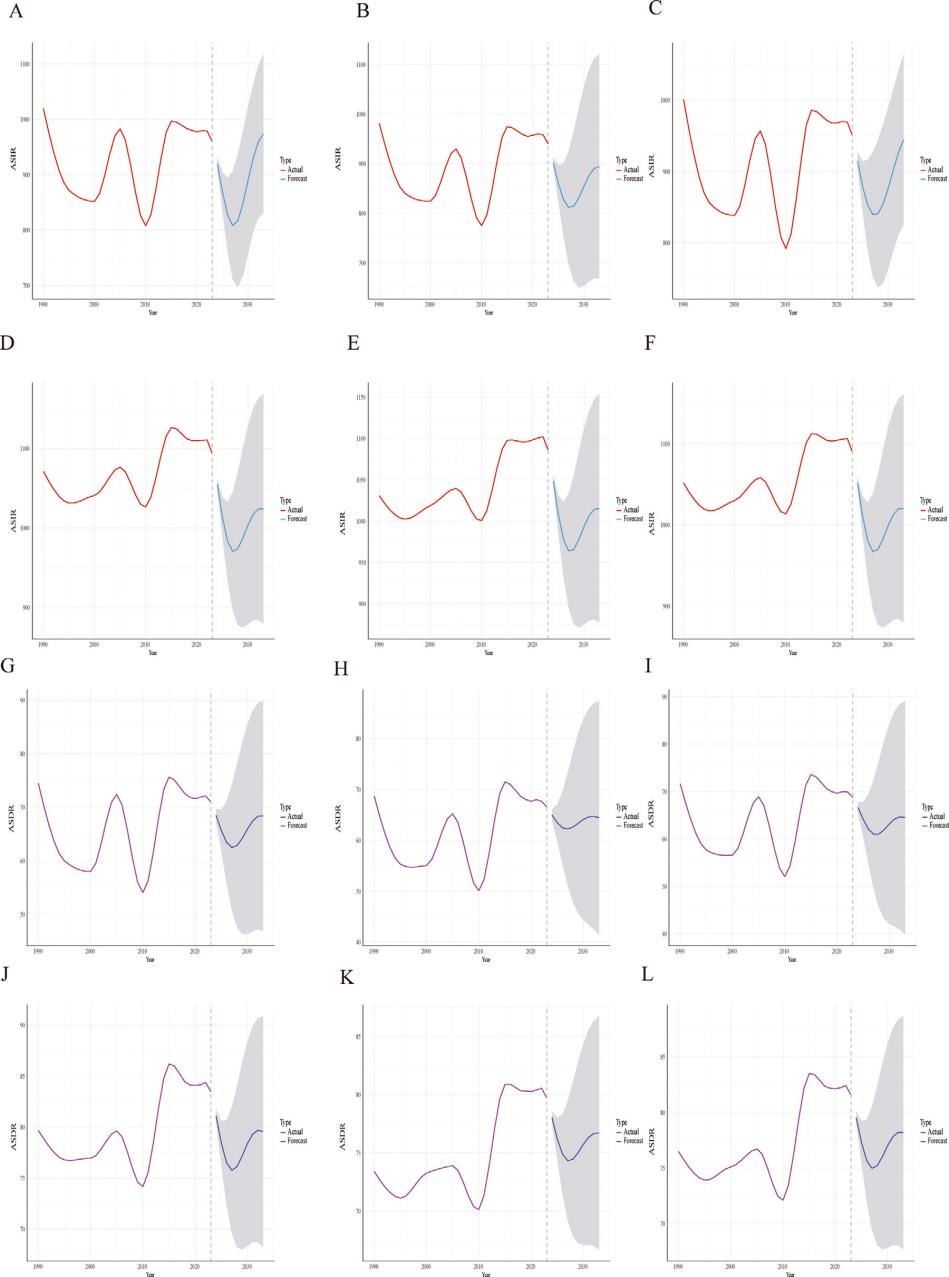
ARIMA-based projections of periodontal diseases incidence and DALYs in China versus the G20 average, by sex, 1990–2023.(A) Incidence rates in China, (B) Incidence rates among males in China, (C) Incidence rates among females in China, (D) Incidence rates in G20, (E) Incidence rates among males in G20, (F) Incidence rates among females in G20, (G) DALY rates in China, (H) DALY rates among males in China, (I) DALY rates among females in China, (J) DALY rates in G20, (K) DALY rates among males in G20, (L) DALY rates among females in G20.

For incidence in China, the model forecasts an increase from 914.79 per 100,000 in 2024 to 944.48 in 2033 for both sexes combined (Figure 5A, Table S11). For males, incidence is projected to decline to 808.12 around 2027-2028 before increasing to 973.68 by 2033 (Figure 5B, Table S12). For females, incidence is projected to decline and then stabilize, reaching 894.04 in 2033 (Figure 5C, Table S13). For the G20, incidence rates are projected to decline to 967.06 in 2027 before increasing to 1020.32 by 2033, a pattern consistent across sexes (Figure 5D, E, F, and Table S14, S15, S16).

For DALY rates in China, the overall rate is projected to decline from 66.61 in 2024 to 61.93 in 2029, then increase to 64.48 by 2033 (Figure 5G, Table S17). For males, the rate is projected to decline to 62.82 in 2028 before increasing to 68.36 in 2033. For females, rates are projected to remain around 64.5 (Figure 5H, I, Table S18, S19). For the G20, the overall DALY rate is projected to decline to 74.97 in 2027 before increasing to 78.17 by 2033 (Figure 5J, Table S20). This pattern is similar for both sexes, with males projected to reach 79.56 and females 76.71 in 2033 (Figure 5K, L, and Table S21, S22).

## Discussion

Our study reveals 3 principal findings: (1) substantial heterogeneity in periodontal disease burden across G20 nations, with China occupying an intermediate-to-high burden position; (2) fundamentally different demographic drivers, with aging dominating in China and population growth in G20 countries; and (3) projected divergence, with China's DALY rates potentially stabilizing while G20 rates remain persistently higher.

The periodontal disease burden among G20 nations exhibits substantial heterogeneity. Results indicate that Brazil (2023: 1157.38 per 100,000) and Turkey (2023: 1164 per 100,000) had the highest incidence rates, while France, the United Kingdom, and South Africa demonstrated the lowest incidence rates. China's position (2023: 950.20 per 100,000) within this spectrum, showing an intermediate to high burden, accurately reflects its unique socioeconomic and epidemiological transition.^28^ On one hand, rapid economic development and increased oral health awareness may have yielded some positive effects; on the other hand, the oral healthcare service system is still under development, coupled with a large population base, resulting in a burden level significantly higher than that of developed countries yet not as severe as some emerging economies.^29^^,^^30^ Furthermore, a consistent male predisposition was evident across all countries, with males exhibiting higher rates in both incidence and DALYs. This disparity was particularly pronounced in China, Germany, and Mexico. This underscores the profound influence of behavioural and socioeconomic factors.^31^ For instance, men globally generally exhibit higher smoking prevalence, which is one of the strongest risk factors for periodontitis, while also typically demonstrating lower utilization of preventive oral care and less proactive healthcare-seeking behaviour compared to women. These well-documented risk factors collectively contribute to the heavier periodontal disease burden observed among men in these countries, particularly in those mentioned.^32^

Distinct age-specific patterns further highlight the different epidemiological profiles between China and the G20. In China, the incidence rate surged rapidly from adolescence,^33^ peaking sharply in the 45-49 age group, with a pronounced male predominance. In contrast, the G20 average exhibited an earlier first peak (25-29 years) and a sustained high plateau from middle age onwards, with minimal gender disparity. This pattern suggests that in many G20 countries, risk factors may manifest earlier in adulthood, potentially linked to dietary habits and oral hygiene practices established in youth.^34^ However, we emphasize that these interpretations are hypotheses based on published literature, not direct findings from our analysis, as our study did not include individual-level behavioural or socioeconomic data. The concentration of the DALYs burden in the 45-59 age group in China signals this demographic as a critical target for intervention, as disease in these productive years can significantly impact overall quality of life and economic productivity.^35^ While these age-specific patterns suggest potential biological or behavioural differences, the possibility of detection bias due to varying screening practices across countries cannot be ruled out. In China, periodontal examinations are not systematically integrated into routine primary care, and case detection often occurs during symptomatic visits or occasional occupational health check-ups, which may disproportionately identify moderate-to-severe cases in middle-aged adults. In contrast, several G20 countries (e.g., Germany, Japan, the United States) have more structured dental recall systems and broader insurance coverage for preventive periodontal assessments, potentially enabling earlier detection in younger age groups. Thus, the earlier incidence peak observed in G20 countries may partly reflect higher screening intensity and diagnostic awareness rather than a purely biological earlier onset of disease.^36^ Without harmonized, population-based screening protocols across nations, cross-country comparisons of age-specific incidence should be interpreted with caution.

Joinpoint regression analysis revealed distinct long-term trends and significant short-term fluctuations in the burden of periodontal disease. From 1990 to 2023, the ASIR in China showed an overall slight downward trend (AAPC = -0.13), but with notable volatility. A marked increase was observed between 2010 and 2014 (APC = 4.79), which coincided with several methodological and health system changes that may have influenced disease reporting. During this period, China’s healthcare reform strengthened primary care infrastructure and began integrating oral health into chronic disease management, likely improving case ascertainment and reporting completeness.^37^, ^38^, ^39^, ^40^ Thus, the observed increase may partially reflect better detection rather than a true rise in disease occurrence.^39^ Additionally, the GBD 2023 framework depends on country-specific surveys; any increase in the number or representativeness of Chinese oral health surveys in the early 2010s could have affected model estimates. Although GBD applies standardized modelling, residual biases from heterogeneous data quality and changes in diagnostic coding cannot be excluded. Future studies linking GBD outputs with national audit data are needed to disentangle true trends from artefacts.

While our data cannot establish causal mechanisms, several concurrent factors may have contributed. For example, although smoking rates have declined overall, smoking among adult males—a high-risk group—remains very high,^41^ and the rapid rise in diabetes prevalence promotes periodontitis.^42^ Furthermore, the population entering middle age between 2010 and 2014 experienced their childhood and youth (the 1980s and 1990s) during the early stage of China economic take-off, when public awareness of oral health and the level of related services were relatively low, and the coverage of preventive measures such as fluoride was limited, so oral health problems accumulated in their early years may have manifested collectively as clinical periodontitis upon entering middle age.^43^ In contrast, the G20 average ASIR showed a slight overall increase (AAPC = 0.13), with a synchronous decline between 2005 and 2009 (APC = -0.91), possibly reflecting global oral health initiatives during that period.^44^ Of particular note is the significant increase in DALY rates among Chinese women between 2010 and 2013 (APC = 9.54), highlighting the need for gender-specific preventive measures addressing women’s unique risk profiles and healthcare access patterns.^45^

Decomposition analysis provided profound insights into the drivers behind the changing burden, revealing fundamentally different demographic stories for China and the G20^11^. In China, the increase of 7.65 million in total incident cases between 1990 and 2023 was predominantly driven by population aging, which accounted for 67.12% of this change.^46^ This finding highlights the immense pressure that demographic transition places on China's healthcare system.^47^ Population growth served as a secondary contributor (44.18%), while favorable epidemiological changes (specifically, declining age-specific incidence rates) modestly mitigated the overall increase (-11.30%). In stark contrast, for the G20, which experienced a 78.6% rise in total cases, population growth was the overwhelming driver (+120.87%), completely offsetting the substantial protective effect of aging (-30.23%). Furthermore, unfavorable epidemiological changes in the G20 contributed significantly to the rising burden (+9.37%),^48^ indicating a potential deterioration in age-specific risk profiles across many member states, possibly linked to the global rise of diabetes and unhealthy dietary patterns.^49^^,^^50^ These interpretations should be viewed as hypotheses generated by the decomposition framework. The epidemiological change component is a residual term that captures the net effect of all factors not accounted for by population growth and aging, including changes in risk factor prevalence (e.g., smoking, diabetes, oral hygiene practices), improvements in treatment, and changes in disease classification or detection. Without independent data on these specific factors, we cannot definitively attribute the epidemiological component to any single mechanism.

An important unmeasured confounder in our analysis is the substantial rural-urban disparity in oral health within China. Epidemiological surveys have consistently shown that periodontal indices are significantly worse in rural populations than in urban ones, with rural residents exhibiting poorer oral hygiene practices, lower awareness of periodontal disease prevention, and less access to dental scaling services.^51^ These disparities persist in more recent surveys, where rural adults consistently show higher prevalence of gingival bleeding and dental calculus compared with their urban counterparts. Consequently, under detection of periodontal disease in rural areas due to limited access to dental services and lower oral health literacy may lead to underestimation of the national incidence and DALY burden. Conversely, delayed care in rural settings may result in a higher proportion of severe cases presenting at later ages, potentially shifting the age specific peak toward older age groups.^52^ The GBD framework does not disaggregate data by subnational regions, and thus these within country heterogeneities remain unaccounted for. Future research should incorporate subnational analyses to capture such disparities and inform geographically targeted public health interventions.

The ARIMA model projections present contrasting futures for periodontal disease burden through 2033. China is projected to experience a fluctuating but overall increasing incidence rate, rising from 914.79 per 100,000 in 2024 to 944.48 in 2033 for both sexes combined. However, its overall DALYs rate is forecasted to decline from 66.61 in 2024 to 61.93 in 2029 before a slight increase to 64.48 by 2033. From a health policy and service planning perspective, this divergence suggests that while the number of new cases might rise, potentially due to persistent risk factors and improved surveillance, the health loss per case could be reducing. This favorable trend in DALYs rates is potentially attributable to ongoing improvements in treatment accessibility, quality of periodontal care, and earlier intervention strategies at the population level.^53^ For the G20, the projections indicate a potential decrease in incidence to a low of 967.06 in 2027 before a modest recovery, yet the persistence of significantly higher DALYs rates compared to China (reaching 78.17 by 2033) is particularly concerning. This implies that G20 health systems may face substantial challenges in reducing the severity, complications, and disability associated with existing cases of periodontitis, pointing to an urgent need for more effective population-based management strategies for advanced stages of the disease and better integration of oral health within primary care systems.^54^^,^^55^

The persistently higher DALY rates projected for G20 countries through 2033 warrant attention not only from a health perspective but also from an economic standpoint. The total worldwide economic impact of oral conditions in 2019 reached $710 billion, comprising $387 billion in direct treatment costs and $323 billion in productivity losses.^1^ Notably, most of these indirect costs were attributable to tooth loss and periodontitis, which together accounted for approximately 3 quarters of total productivity losses. Focusing specifically on G20 economies, a detailed assessment of the United States and Europe found that periodontal disease caused an estimated economic loss of $154 billion in the US and €159 billion in Europe in 2018, with indirect costs representing 0.73% of US GDP and 0.99% of European GDP.^56^ These figures underscore that the DALY burden we observed is accompanied by substantial economic consequences. The persistently high DALY rates in G20 nations—despite declining or stabilizing incidence in some countries—suggest that existing clinical care models may be managing new cases but are not effectively reducing the long-term disability and associated economic losses attributable to periodontitis. This disconnect implies that G20 health systems may benefit from shifting from a predominantly treatment-oriented approach toward population-based prevention strategies targeting common risk factors (e.g., smoking, diabetes control, sugar reduction), which could simultaneously alleviate both the clinical and economic burden.

Emerging evidence suggests that salivary biomarkers, including inflammatory cytokines and microbial metabolites, may offer non-invasive tools for periodontitis screening and monitoring. As recent work has demonstrated, such biomarkers could enhance early detection in population-based surveillance, potentially improving the accuracy of future GBD estimates by reducing reliance on clinical examination alone.^57^

The relationship between periodontitis and cardiovascular outcomes has been further elucidated in recent studies. Periodontal treatment may reduce systemic inflammatory biomarkers, suggesting that the systemic health implications of periodontitis extend beyond the oral cavity.^58^ This reinforces the public health importance of our findings, as the DALY burden we report likely underestimates the full health impact by not accounting for downstream cardiovascular events potentially attributable to periodontitis.

This study has several limitations. First, the case definition of periodontal disease in the GBD framework primarily relies on population-based surveys and models, which may not fully capture the clinical severity or individual-level risk profiles. Second, while our analysis identified demographic drivers, it did not incorporate specific behavioural, socioeconomic, or healthcare access variables due to data constraints, limiting the exploration of underlying mechanisms behind the observed disparities. Third, the forecasting analysis using ARIMA models is based solely on extrapolation of historical temporal trends. While useful for identifying near-term trajectory patterns, these models do not incorporate covariates such as future socioeconomic development, policy changes, or risk factor prevalence, which are key determinants of disease burden. Therefore, our projections should be interpreted as trend-based illustrations rather than deterministic predictions. Finally, our study period includes the initial phase of the COVID-19 pandemic (2020-2021), during which routine and elective dental services were severely disrupted globally. The GBD 2023 estimates may not fully capture the long-term impacts of these disruptions on the incidence, progression, and management of periodontal disease. The pandemic may have led to reduced detection of new cases (biasing incidence estimates downward) while potentially increasing disease progression among existing cases due to delayed care (biasing DALY estimates). The ARIMA models, being based on historical trends up to 2023, incorporate the 2020-2021 data points but cannot predict how post-pandemic recovery patterns might alter long-term trends. Readers should interpret projections for the immediate post-pandemic years (2024-2025) with particular caution given this structural break in the time series. Importantly, several contextual explanations offered in this Discussion—including those related to healthcare system reform, smoking trends, diabetes prevalence, fluoride exposure, and health-seeking behaviours—are plausible but remain speculative in the absence of direct empirical testing within our analytical framework. Readers should interpret these as hypothesis-generating observations rather than causal conclusions drawn from our data. The annual iterative updates of the GBD study, which incorporate the latest data, enable dynamic documentation of the long-term association between shifting socioeconomic trajectories and the burden of oral diseases. Analyses of these longitudinal trends offer a critical evidence base for evaluating the effectiveness of oral health policies across various stages of national development.

## Conclusion

In conclusion, this analysis reveals distinct periodontal disease burden profiles and driving factors between China and G20 countries from 1990 to 2023. China exhibits a moderately high burden, primarily driven by population aging and characterized by significant male predisposition, while population growth serves as the main driver in G20 nations. Projections to 2033 suggest potential stabilization of health loss in China, contrasting with persistently higher DALY rates in G20 countries. These findings highlight the necessity for tailored public health strategies: focusing on high-risk demographic groups in China (particularly middle-aged males and the aging population) and enhancing population-level management of advanced diseases and shared risk factors in G20 countries to mitigate the global burden of periodontal disease.

## Declaration of generative AI and AI-assisted technologies

During the preparation of this work, the authors did not use any generative AI or AI-assisted technologies for data analysis, interpretation, or manuscript writing. Grammarly Business was used solely for grammar and spelling checking. The authors take full responsibility for the content and integrity of this work

## Ethics approval and consent to participate

Ethical approval was not required for this study as it relied exclusively on publicly available, anonymized data from the Global Burden of Disease Study 2023.

## Consent for publication

Not applicable.

## Data availability statement

The data used in this study are from the Global Burden of Disease 2023 study (GBD 2023) and are publicly available through the Global Health Data Exchange (GHDx) at: https://ghdx.healthdata.org/gbd-2023. All data can be freely accessed via the above link.

## Author contributions

Yilin Zhang was responsible for conceptualization, methodology, formal analysis, writing – original draft, and visualization. Xin Xu contributed to conceptualization, methodology, supervision, project administration, and funding acquisition. Xun Wang and Yuan Tang performed data curation, validation, and formal analysis. Fei He provided resources, critical revisions, and technical support. Jing Li as the corresponding author, oversaw the project, contributed to methodology, writing – review & editing, and supervised the overall research process.

## Funding

This study was supported by the Shandong Provincial Natural Science Foundation (Grant No. ZR2025QC908) and the National Traditional Chinese Medicine Comprehensive Reform Demonstration Zone Science and Technology Co-construction Project (Grant No. GZY-KJS-SD-2024-106). The funders had no role in study design, data collection, analysis, interpretation, manuscript writing, or the decision to submit for publication.

## Conflict of interest

None disclosed.

## Appendix Supplementary materials

Image, application 1

## Footnotes

Supplementary material associated with this article can be found in the online version at doi:10.1016/j.identj.2026.109655.
