# Supplementary material for: Periodontal Disease Burden in China and G20 Nations: Insights From the Global Burden of Disease 2023 Study
Source: Int Dent J. 2026 May 29;76(4):109655. doi: 10.1016/j.identj.2026.109655 (PMC13235353; doi:10.1016/j.identj.2026.109655)
Supplement: Supplementary file 1 [file mmc1.pdf]

Supplementary Materials

Table S1. Trends in age-standardized incidence of periodontal diseases in China and G20 countries, 1990–2023 1
Table S2. Trends in age-standardized DALY rates of periodontal diseases in China and G20 countries, 1990–2023 110
Table S3. Age distribution of periodontal diseases incidence in China 207
Table S4. Age distribution of DALYs due to periodontal diseases in China 212
Table S5. Age distribution of periodontal diseases incidence in G20 countries 217
Table S6. Age distribution of DALYs due to periodontal diseases in G20 countries 222
Table S7. Decomposition analysis of temporal changes in periodontal diseases incidence in China 226
Table S8. Decomposition analysis of temporal changes in DALYs due to periodontal diseases in China 227
Table S9. Decomposition analysis of temporal changes in periodontal diseases incidence in G20 countries 228
Table S10. Decomposition analysis of temporal changes in DALYs due to periodontal diseases in G20 countries 229
Table S11. ARIMA model forecasts of periodontal diseases incidence in China 230
Table S12. ARIMA model forecasts of periodontal diseases incidence among males in China 232
Table S13. ARIMA model forecasts of periodontal diseases incidence among females in China 234
Table S14. ARIMA model forecasts of periodontal diseases incidence in G20 countries 236
Table S15. ARIMA model forecasts of periodontal diseases incidence among males in G20 countries 238
Table S16. ARIMA model forecasts of periodontal diseases incidence among females in G20 countries 240
Table S17. ARIMA model forecasts of DALYs due to periodontal diseases in China 242
Table S18. ARIMA model forecasts of DALYs due to periodontal diseases among males in China 244
Table S19. ARIMA model forecasts of DALYs due to periodontal diseases among females in China 246
Table S20. ARIMA model forecasts of DALYs due to periodontal diseases in G20 countries 248
Table S21. ARIMA model forecasts of DALYs due to periodontal diseases among males in G20 countries 250
Table S22. ARIMA model forecasts of DALYs due to periodontal diseases among females in G20 countries 252
Table S23. Best-fitting ARIMA model parameters and goodness-of-fit statistics for age-standardized incidence and DALY rates of periodontal diseases in China and G20 countries, by sex, 1990–2023. 255
Table S24. The RECORD statement – checklist of items, extended from the STROBE statement, that should be reported in observational studies using routinely collected health data. 256
Table S25. STROBE Statement—checklist of items that should be included in reports of observational studies 266

Table S1. Trends in age-standardized incidence of periodontal diseases in China and G20 countries, 1990–2023

	measure_name	location_name	sex_name	age_name	cause_name	metric_name	year	val	upper	lower
1	Incidence	French Republic	Male	Age-standardized	Periodontal diseases	Rate	1990	616.41	800.76	422.99
2	Incidence	French Republic	Female	Age-standardized	Periodontal diseases	Rate	1990	653.99	825.05	518.16
3	Incidence	French Republic	Both	Age-standardized	Periodontal diseases	Rate	1990	638.89	759.91	513.16
4	Incidence	French Republic	Male	Age-standardized	Periodontal diseases	Rate	1991	572.71	712.73	426.50
5	Incidence	French Republic	Female	Age-standardized	Periodontal diseases	Rate	1991	601.16	764.68	473.75
6	Incidence	French Republic	Both	Age-standardized	Periodontal diseases	Rate	1991	589.48	702.60	481.35
7	Incidence	French Republic	Male	Age-standardized	Periodontal diseases	Rate	1992	540.25	650.41	423.61
8	Incidence	French Republic	Female	Age-standardized	Periodontal diseases	Rate	1992	560.71	716.94	441.98
9	Incidence	French Republic	Both	Age-standardized	Periodontal diseases	Rate	1992	552.03	669.54	456.24
10	Incidence	French Republic	Male	Age-standardized	Periodontal diseases	Rate	1993	518.24	626.84	415.71
11	Incidence	French Republic	Female	Age-standardized	Periodontal diseases	Rate	1993	532.28	688.94	418.94
12	Incidence	French Republic	Both	Age-standardized	Periodontal diseases	Rate	1993	526.02	654.98	431.82
13	Incidence	French Republic	Male	Age-standardized	Periodontal diseases	Rate	1994	505.89	634.21	406.84
14	Incidence	French Republic	Female	Age-standardized	Periodontal diseases	Rate	1994	515.51	677.81	404.27
15	Incidence	French Republic	Both	Age-standardized	Periodontal diseases	Rate	1994	510.91	654.67	410.30
16	Incidence	French Republic	Male	Age-standardized	Periodontal diseases	Rate	1995	502.42	656.34	388.81
17	Incidence	French Republic	Female	Age-standardized	Periodontal diseases	Rate	1995	510.01	675.22	396.63
18	Incidence	French Republic	Both	Age-standardized	Periodontal diseases	Rate	1995	506.20	664.14	393.25
19	Incidence	French Republic	Male	Age-standardized	Periodontal diseases	Rate	1996	518.66	673.15	401.94
20	Incidence	French Republic	Female	Age-standardized	Periodontal diseases	Rate	1996	526.10	687.00	409.75
21	Incidence	French Republic	Both	Age-standardized	Periodontal diseases	Rate	1996	522.36	678.24	405.09

22	Incidence	French Republic	Male	Age-standardized	Periodontal diseases	Rate	1997	556.53	715.80	429.27
23	Incidence	French Republic	Female	Age-standardized	Periodontal diseases	Rate	1997	564.65	731.23	439.66
24	Incidence	French Republic	Both	Age-standardized	Periodontal diseases	Rate	1997	560.61	721.18	433.21
25	Incidence	French Republic	Male	Age-standardized	Periodontal diseases	Rate	1998	601.88	766.15	462.06
26	Incidence	French Republic	Female	Age-standardized	Periodontal diseases	Rate	1998	611.16	779.85	477.58
27	Incidence	French Republic	Both	Age-standardized	Periodontal diseases	Rate	1998	606.60	773.97	468.09
28	Incidence	French Republic	Male	Age-standardized	Periodontal diseases	Rate	1999	640.53	809.56	490.08
29	Incidence	French Republic	Female	Age-standardized	Periodontal diseases	Rate	1999	651.11	826.09	510.73
30	Incidence	French Republic	Both	Age-standardized	Periodontal diseases	Rate	1999	645.96	817.79	498.11
31	Incidence	French Republic	Male	Age-standardized	Periodontal diseases	Rate	2000	658.33	824.55	503.41
32	Incidence	French Republic	Female	Age-standardized	Periodontal diseases	Rate	2000	670.00	845.62	527.01
33	Incidence	French Republic	Both	Age-standardized	Periodontal diseases	Rate	2000	664.33	833.09	511.90
34	Incidence	French Republic	Male	Age-standardized	Periodontal diseases	Rate	2001	660.55	829.73	505.69
35	Incidence	French Republic	Female	Age-standardized	Periodontal diseases	Rate	2001	672.99	849.62	527.68
36	Incidence	French Republic	Both	Age-standardized	Periodontal diseases	Rate	2001	666.94	839.18	513.98
37	Incidence	French Republic	Male	Age-standardized	Periodontal diseases	Rate	2002	662.08	837.35	507.28
38	Incidence	French Republic	Female	Age-standardized	Periodontal diseases	Rate	2002	675.11	851.92	526.68
39	Incidence	French Republic	Both	Age-standardized	Periodontal diseases	Rate	2002	668.75	843.47	515.90
40	Incidence	French Republic	Male	Age-standardized	Periodontal diseases	Rate	2003	663.15	845.43	508.75
41	Incidence	French Republic	Female	Age-standardized	Periodontal diseases	Rate	2003	676.58	848.99	528.52
42	Incidence	French Republic	Both	Age-standardized	Periodontal diseases	Rate	2003	670.01	847.32	517.50
43	Incidence	French Republic	Male	Age-standardized	Periodontal diseases	Rate	2004	664.01	850.79	510.01

44	Incidence	French Republic	Female	Age-standardized	Periodontal diseases	Rate	2004	677.65	854.97	527.35
45	Incidence	French Republic	Both	Age-standardized	Periodontal diseases	Rate	2004	670.95	849.39	518.75
46	Incidence	French Republic	Male	Age-standardized	Periodontal diseases	Rate	2005	664.88	858.64	511.65
47	Incidence	French Republic	Female	Age-standardized	Periodontal diseases	Rate	2005	678.55	860.23	528.14
48	Incidence	French Republic	Both	Age-standardized	Periodontal diseases	Rate	2005	671.82	853.68	519.80
49	Incidence	French Republic	Male	Age-standardized	Periodontal diseases	Rate	2006	666.68	856.95	513.88
50	Incidence	French Republic	Female	Age-standardized	Periodontal diseases	Rate	2006	679.74	858.87	533.19
51	Incidence	French Republic	Both	Age-standardized	Periodontal diseases	Rate	2006	673.30	852.14	523.68
52	Incidence	French Republic	Male	Age-standardized	Periodontal diseases	Rate	2007	669.64	858.84	517.47
53	Incidence	French Republic	Female	Age-standardized	Periodontal diseases	Rate	2007	681.38	860.03	539.01
54	Incidence	French Republic	Both	Age-standardized	Periodontal diseases	Rate	2007	675.57	854.21	528.36
55	Incidence	French Republic	Male	Age-standardized	Periodontal diseases	Rate	2008	672.96	860.84	521.05
56	Incidence	French Republic	Female	Age-standardized	Periodontal diseases	Rate	2008	683.21	864.69	542.08
57	Incidence	French Republic	Both	Age-standardized	Periodontal diseases	Rate	2008	678.11	859.79	533.29
58	Incidence	French Republic	Male	Age-standardized	Periodontal diseases	Rate	2009	675.84	863.46	523.88
59	Incidence	French Republic	Female	Age-standardized	Periodontal diseases	Rate	2009	684.96	863.18	543.61
60	Incidence	French Republic	Both	Age-standardized	Periodontal diseases	Rate	2009	680.40	863.74	537.22
61	Incidence	French Republic	Male	Age-standardized	Periodontal diseases	Rate	2010	677.47	863.60	526.03
62	Incidence	French Republic	Female	Age-standardized	Periodontal diseases	Rate	2010	686.37	864.29	545.04
63	Incidence	French Republic	Both	Age-standardized	Periodontal diseases	Rate	2010	681.92	866.26	538.75
64	Incidence	French Republic	Male	Age-standardized	Periodontal diseases	Rate	2011	678.31	860.90	526.35
65	Incidence	French Republic	Female	Age-standardized	Periodontal diseases	Rate	2011	688.08	864.55	548.28

66	Incidence	French Republic	Both	Age-standardized	Periodontal diseases	Rate	2011	683.22	864.14	539.32
67	Incidence	French Republic	Male	Age-standardized	Periodontal diseases	Rate	2012	679.24	858.20	524.57
68	Incidence	French Republic	Female	Age-standardized	Periodontal diseases	Rate	2012	690.54	868.95	547.80
69	Incidence	French Republic	Both	Age-standardized	Periodontal diseases	Rate	2012	684.96	862.22	539.12
70	Incidence	French Republic	Male	Age-standardized	Periodontal diseases	Rate	2013	680.14	855.91	522.73
71	Incidence	French Republic	Female	Age-standardized	Periodontal diseases	Rate	2013	693.19	868.79	545.78
72	Incidence	French Republic	Both	Age-standardized	Periodontal diseases	Rate	2013	686.78	861.08	537.68
73	Incidence	French Republic	Male	Age-standardized	Periodontal diseases	Rate	2014	680.88	854.80	522.15
74	Incidence	French Republic	Female	Age-standardized	Periodontal diseases	Rate	2014	695.47	871.11	545.58
75	Incidence	French Republic	Both	Age-standardized	Periodontal diseases	Rate	2014	688.33	860.53	535.81
76	Incidence	French Republic	Male	Age-standardized	Periodontal diseases	Rate	2015	681.33	855.70	523.58
77	Incidence	French Republic	Female	Age-standardized	Periodontal diseases	Rate	2015	696.84	872.59	546.02
78	Incidence	French Republic	Both	Age-standardized	Periodontal diseases	Rate	2015	689.27	860.07	534.86
79	Incidence	French Republic	Male	Age-standardized	Periodontal diseases	Rate	2016	681.52	856.31	523.26
80	Incidence	French Republic	Female	Age-standardized	Periodontal diseases	Rate	2016	697.74	870.02	544.53
81	Incidence	French Republic	Both	Age-standardized	Periodontal diseases	Rate	2016	689.83	863.71	537.29
82	Incidence	French Republic	Male	Age-standardized	Periodontal diseases	Rate	2017	681.67	864.12	522.12
83	Incidence	French Republic	Female	Age-standardized	Periodontal diseases	Rate	2017	698.76	867.55	549.61
84	Incidence	French Republic	Both	Age-standardized	Periodontal diseases	Rate	2017	690.42	867.99	539.40
85	Incidence	French Republic	Male	Age-standardized	Periodontal diseases	Rate	2018	681.95	869.24	521.10
86	Incidence	French Republic	Female	Age-standardized	Periodontal diseases	Rate	2018	699.53	867.52	555.58
87	Incidence	French Republic	Both	Age-standardized	Periodontal diseases	Rate	2018	690.95	870.19	539.45

88	Incidence	French Republic	Male	Age-standardized	Periodontal diseases	Rate	2019	682.55	872.62	522.85
89	Incidence	French Republic	Female	Age-standardized	Periodontal diseases	Rate	2019	699.72	866.25	556.79
90	Incidence	French Republic	Both	Age-standardized	Periodontal diseases	Rate	2019	691.33	873.26	539.06
91	Incidence	French Republic	Male	Age-standardized	Periodontal diseases	Rate	2020	689.83	881.30	540.02
92	Incidence	French Republic	Female	Age-standardized	Periodontal diseases	Rate	2020	695.62	868.62	542.11
93	Incidence	French Republic	Both	Age-standardized	Periodontal diseases	Rate	2020	692.73	874.98	540.04
94	Incidence	French Republic	Male	Age-standardized	Periodontal diseases	Rate	2021	685.26	879.39	533.12
95	Incidence	French Republic	Female	Age-standardized	Periodontal diseases	Rate	2021	697.34	886.87	542.07
96	Incidence	French Republic	Both	Age-standardized	Periodontal diseases	Rate	2021	691.45	873.44	536.57
97	Incidence	French Republic	Male	Age-standardized	Periodontal diseases	Rate	2022	683.11	888.63	541.41
98	Incidence	French Republic	Female	Age-standardized	Periodontal diseases	Rate	2022	695.72	876.31	543.68
99	Incidence	French Republic	Both	Age-standardized	Periodontal diseases	Rate	2022	689.58	879.43	542.78
100	Incidence	French Republic	Male	Age-standardized	Periodontal diseases	Rate	2023	678.00	882.41	536.55
101	Incidence	French Republic	Female	Age-standardized	Periodontal diseases	Rate	2023	690.51	869.74	539.28
102	Incidence	French Republic	Both	Age-standardized	Periodontal diseases	Rate	2023	684.42	873.69	538.56
103	Incidence	Australia	Male	Age-standardized	Periodontal diseases	Rate	1990	973.73	1180.90	744.89
104	Incidence	Australia	Female	Age-standardized	Periodontal diseases	Rate	1990	723.08	905.52	518.73
105	Incidence	Australia	Both	Age-standardized	Periodontal diseases	Rate	1990	847.30	1044.42	631.62
106	Incidence	Australia	Male	Age-standardized	Periodontal diseases	Rate	1991	853.65	972.38	695.29
107	Incidence	Australia	Female	Age-standardized	Periodontal diseases	Rate	1991	657.97	773.50	515.06
108	Incidence	Australia	Both	Age-standardized	Periodontal diseases	Rate	1991	755.01	876.23	604.40
109	Incidence	Australia	Male	Age-standardized	Periodontal diseases	Rate	1992	758.86	845.93	650.35

110	Incidence	Australia	Female	Age-standardized	Periodontal diseases	Rate	1992	608.47	687.63	514.27
111	Incidence	Australia	Both	Age-standardized	Periodontal diseases	Rate	1992	683.12	761.79	583.38
112	Incidence	Australia	Male	Age-standardized	Periodontal diseases	Rate	1993	690.15	758.18	617.57
113	Incidence	Australia	Female	Age-standardized	Periodontal diseases	Rate	1993	573.96	632.21	510.25
114	Incidence	Australia	Both	Age-standardized	Periodontal diseases	Rate	1993	631.70	693.81	561.45
115	Incidence	Australia	Male	Age-standardized	Periodontal diseases	Rate	1994	648.34	709.07	593.65
116	Incidence	Australia	Female	Age-standardized	Periodontal diseases	Rate	1994	553.83	605.48	507.44
117	Incidence	Australia	Both	Age-standardized	Periodontal diseases	Rate	1994	600.83	655.47	550.74
118	Incidence	Australia	Male	Age-standardized	Periodontal diseases	Rate	1995	634.23	703.40	570.58
119	Incidence	Australia	Female	Age-standardized	Periodontal diseases	Rate	1995	547.46	604.79	499.60
120	Incidence	Australia	Both	Age-standardized	Periodontal diseases	Rate	1995	590.60	655.03	537.22
121	Incidence	Australia	Male	Age-standardized	Periodontal diseases	Rate	1996	665.61	747.06	599.37
122	Incidence	Australia	Female	Age-standardized	Periodontal diseases	Rate	1996	568.68	638.28	513.23
123	Incidence	Australia	Both	Age-standardized	Periodontal diseases	Rate	1996	616.74	693.75	555.61
124	Incidence	Australia	Male	Age-standardized	Periodontal diseases	Rate	1997	740.62	834.92	651.92
125	Incidence	Australia	Female	Age-standardized	Periodontal diseases	Rate	1997	619.05	709.02	540.41
126	Incidence	Australia	Both	Age-standardized	Periodontal diseases	Rate	1997	679.13	773.43	595.36
127	Incidence	Australia	Male	Age-standardized	Periodontal diseases	Rate	1998	830.59	959.10	700.24
128	Incidence	Australia	Female	Age-standardized	Periodontal diseases	Rate	1998	679.57	799.64	575.48
129	Incidence	Australia	Both	Age-standardized	Periodontal diseases	Rate	1998	754.05	876.35	637.70
130	Incidence	Australia	Male	Age-standardized	Periodontal diseases	Rate	1999	906.84	1082.63	735.60
131	Incidence	Australia	Female	Age-standardized	Periodontal diseases	Rate	1999	731.25	889.25	598.52

132	Incidence	Australia	Both	Age-standardized	Periodontal diseases	Rate	1999	817.77	978.44	667.27
133	Incidence	Australia	Male	Age-standardized	Periodontal diseases	Rate	2000	940.70	1170.47	738.97
134	Incidence	Australia	Female	Age-standardized	Periodontal diseases	Rate	2000	755.08	949.40	591.92
135	Incidence	Australia	Both	Age-standardized	Periodontal diseases	Rate	2000	846.55	1043.31	655.61
136	Incidence	Australia	Male	Age-standardized	Periodontal diseases	Rate	2001	938.91	1145.56	737.84
137	Incidence	Australia	Female	Age-standardized	Periodontal diseases	Rate	2001	753.90	929.90	594.58
138	Incidence	Australia	Both	Age-standardized	Periodontal diseases	Rate	2001	845.14	1030.14	664.21
139	Incidence	Australia	Male	Age-standardized	Periodontal diseases	Rate	2002	930.17	1113.96	741.48
140	Incidence	Australia	Female	Age-standardized	Periodontal diseases	Rate	2002	745.93	909.39	589.60
141	Incidence	Australia	Both	Age-standardized	Periodontal diseases	Rate	2002	836.84	1006.26	666.04
142	Incidence	Australia	Male	Age-standardized	Periodontal diseases	Rate	2003	918.77	1092.12	747.06
143	Incidence	Australia	Female	Age-standardized	Periodontal diseases	Rate	2003	735.26	895.20	581.29
144	Incidence	Australia	Both	Age-standardized	Periodontal diseases	Rate	2003	825.85	987.09	662.44
145	Incidence	Australia	Male	Age-standardized	Periodontal diseases	Rate	2004	908.97	1071.79	741.87
146	Incidence	Australia	Female	Age-standardized	Periodontal diseases	Rate	2004	725.96	886.12	581.38
147	Incidence	Australia	Both	Age-standardized	Periodontal diseases	Rate	2004	816.32	973.77	661.43
148	Incidence	Australia	Male	Age-standardized	Periodontal diseases	Rate	2005	905.07	1067.57	738.30
149	Incidence	Australia	Female	Age-standardized	Periodontal diseases	Rate	2005	722.12	877.30	583.11
150	Incidence	Australia	Both	Age-standardized	Periodontal diseases	Rate	2005	812.46	970.55	662.67
151	Incidence	Australia	Male	Age-standardized	Periodontal diseases	Rate	2006	910.72	1069.89	747.14
152	Incidence	Australia	Female	Age-standardized	Periodontal diseases	Rate	2006	728.95	890.98	585.67
153	Incidence	Australia	Both	Age-standardized	Periodontal diseases	Rate	2006	818.71	977.14	664.46

154	Incidence	Australia	Male	Age-standardized	Periodontal diseases	Rate	2007	923.88	1089.72	762.22
155	Incidence	Australia	Female	Age-standardized	Periodontal diseases	Rate	2007	744.99	907.85	593.46
156	Incidence	Australia	Both	Age-standardized	Periodontal diseases	Rate	2007	833.34	996.44	669.92
157	Incidence	Australia	Male	Age-standardized	Periodontal diseases	Rate	2008	940.25	1117.35	765.45
158	Incidence	Australia	Female	Age-standardized	Periodontal diseases	Rate	2008	764.29	936.90	602.71
159	Incidence	Australia	Both	Age-standardized	Periodontal diseases	Rate	2008	851.22	1020.89	679.83
160	Incidence	Australia	Male	Age-standardized	Periodontal diseases	Rate	2009	955.53	1156.92	773.13
161	Incidence	Australia	Female	Age-standardized	Periodontal diseases	Rate	2009	780.95	979.88	609.92
162	Incidence	Australia	Both	Age-standardized	Periodontal diseases	Rate	2009	867.20	1047.86	685.14
163	Incidence	Australia	Male	Age-standardized	Periodontal diseases	Rate	2010	965.40	1191.48	775.86
164	Incidence	Australia	Female	Age-standardized	Periodontal diseases	Rate	2010	789.01	1002.84	611.76
165	Incidence	Australia	Both	Age-standardized	Periodontal diseases	Rate	2010	876.17	1073.18	690.72
166	Incidence	Australia	Male	Age-standardized	Periodontal diseases	Rate	2011	975.81	1207.14	784.97
167	Incidence	Australia	Female	Age-standardized	Periodontal diseases	Rate	2011	790.83	997.24	610.03
168	Incidence	Australia	Both	Age-standardized	Periodontal diseases	Rate	2011	882.19	1079.98	696.99
169	Incidence	Australia	Male	Age-standardized	Periodontal diseases	Rate	2012	992.35	1218.48	796.84
170	Incidence	Australia	Female	Age-standardized	Periodontal diseases	Rate	2012	792.65	992.15	611.99
171	Incidence	Australia	Both	Age-standardized	Periodontal diseases	Rate	2012	891.15	1089.12	704.13
172	Incidence	Australia	Male	Age-standardized	Periodontal diseases	Rate	2013	1010.17	1229.41	810.36
173	Incidence	Australia	Female	Age-standardized	Periodontal diseases	Rate	2013	794.37	989.76	616.69
174	Incidence	Australia	Both	Age-standardized	Periodontal diseases	Rate	2013	900.67	1097.36	713.77
175	Incidence	Australia	Male	Age-standardized	Periodontal diseases	Rate	2014	1024.43	1235.38	819.66

176	Incidence	Australia	Female	Age-standardized	Periodontal diseases	Rate	2014	795.89	989.93	623.12
177	Incidence	Australia	Both	Age-standardized	Periodontal diseases	Rate	2014	908.33	1102.92	720.95
178	Incidence	Australia	Male	Age-standardized	Periodontal diseases	Rate	2015	1030.29	1236.27	820.91
179	Incidence	Australia	Female	Age-standardized	Periodontal diseases	Rate	2015	797.12	991.34	628.93
180	Incidence	Australia	Both	Age-standardized	Periodontal diseases	Rate	2015	911.77	1104.87	725.73
181	Incidence	Australia	Male	Age-standardized	Periodontal diseases	Rate	2016	1021.68	1223.90	811.84
182	Incidence	Australia	Female	Age-standardized	Periodontal diseases	Rate	2016	799.05	988.39	629.45
183	Incidence	Australia	Both	Age-standardized	Periodontal diseases	Rate	2016	908.56	1099.66	719.97
184	Incidence	Australia	Male	Age-standardized	Periodontal diseases	Rate	2017	1002.46	1201.75	794.26
185	Incidence	Australia	Female	Age-standardized	Periodontal diseases	Rate	2017	801.96	997.56	632.49
186	Incidence	Australia	Both	Age-standardized	Periodontal diseases	Rate	2017	900.70	1090.20	710.94
187	Incidence	Australia	Male	Age-standardized	Periodontal diseases	Rate	2018	982.69	1180.66	776.29
188	Incidence	Australia	Female	Age-standardized	Periodontal diseases	Rate	2018	804.72	1002.42	635.81
189	Incidence	Australia	Both	Age-standardized	Periodontal diseases	Rate	2018	892.51	1081.96	704.21
190	Incidence	Australia	Male	Age-standardized	Periodontal diseases	Rate	2019	972.44	1171.09	769.87
191	Incidence	Australia	Female	Age-standardized	Periodontal diseases	Rate	2019	806.18	1008.89	637.92
192	Incidence	Australia	Both	Age-standardized	Periodontal diseases	Rate	2019	888.28	1078.29	701.92
193	Incidence	Australia	Male	Age-standardized	Periodontal diseases	Rate	2020	976.12	1184.38	770.60
194	Incidence	Australia	Female	Age-standardized	Periodontal diseases	Rate	2020	802.96	1012.05	626.26
195	Incidence	Australia	Both	Age-standardized	Periodontal diseases	Rate	2020	888.41	1089.73	696.69
196	Incidence	Australia	Male	Age-standardized	Periodontal diseases	Rate	2021	975.81	1218.46	790.30
197	Incidence	Australia	Female	Age-standardized	Periodontal diseases	Rate	2021	797.22	999.21	629.29

198	Incidence	Australia	Both	Age-standardized	Periodontal diseases	Rate	2021	885.43	1098.06	708.73
199	Incidence	Australia	Male	Age-standardized	Periodontal diseases	Rate	2022	971.63	1198.08	760.38
200	Incidence	Australia	Female	Age-standardized	Periodontal diseases	Rate	2022	798.51	995.22	626.98
201	Incidence	Australia	Both	Age-standardized	Periodontal diseases	Rate	2022	883.98	1086.45	692.83
202	Incidence	Australia	Male	Age-standardized	Periodontal diseases	Rate	2023	963.19	1187.94	752.99
203	Incidence	Australia	Female	Age-standardized	Periodontal diseases	Rate	2023	791.56	986.64	620.74
204	Incidence	Australia	Both	Age-standardized	Periodontal diseases	Rate	2023	876.31	1077.60	686.44
205	Incidence	Republic of Korea	Male	Age-standardized	Periodontal diseases	Rate	1990	846.40	1030.33	627.33
206	Incidence	Republic of Korea	Female	Age-standardized	Periodontal diseases	Rate	1990	585.54	735.18	410.90
207	Incidence	Republic of Korea	Both	Age-standardized	Periodontal diseases	Rate	1990	713.62	878.48	524.01
208	Incidence	Republic of Korea	Male	Age-standardized	Periodontal diseases	Rate	1991	856.18	1041.00	638.91
209	Incidence	Republic of Korea	Female	Age-standardized	Periodontal diseases	Rate	1991	592.10	721.40	444.34
210	Incidence	Republic of Korea	Both	Age-standardized	Periodontal diseases	Rate	1991	722.53	871.51	543.31
211	Incidence	Republic of Korea	Male	Age-standardized	Periodontal diseases	Rate	1992	863.45	1049.78	647.36
212	Incidence	Republic of Korea	Female	Age-standardized	Periodontal diseases	Rate	1992	598.53	720.12	470.39
213	Incidence	Republic of Korea	Both	Age-standardized	Periodontal diseases	Rate	1992	730.06	871.21	564.60
214	Incidence	Republic of Korea	Male	Age-standardized	Periodontal diseases	Rate	1993	868.54	1058.49	654.18
215	Incidence	Republic of Korea	Female	Age-standardized	Periodontal diseases	Rate	1993	604.38	716.24	494.48
216	Incidence	Republic of Korea	Both	Age-standardized	Periodontal diseases	Rate	1993	736.03	867.11	581.75
217	Incidence	Republic of Korea	Male	Age-standardized	Periodontal diseases	Rate	1994	871.75	1067.04	659.50
218	Incidence	Republic of Korea	Female	Age-standardized	Periodontal diseases	Rate	1994	609.16	737.83	495.36
219	Incidence	Republic of Korea	Both	Age-standardized	Periodontal diseases	Rate	1994	740.39	868.36	593.27

220	Incidence	Republic of Korea	Male	Age-standardized	Periodontal diseases	Rate	1995	873.40	1074.91	660.76
221	Incidence	Republic of Korea	Female	Age-standardized	Periodontal diseases	Rate	1995	612.42	772.16	474.70
222	Incidence	Republic of Korea	Both	Age-standardized	Periodontal diseases	Rate	1995	742.95	874.89	607.72
223	Incidence	Republic of Korea	Male	Age-standardized	Periodontal diseases	Rate	1996	870.17	1028.32	692.85
224	Incidence	Republic of Korea	Female	Age-standardized	Periodontal diseases	Rate	1996	615.43	766.08	480.45
225	Incidence	Republic of Korea	Both	Age-standardized	Periodontal diseases	Rate	1996	742.85	867.50	623.23
226	Incidence	Republic of Korea	Male	Age-standardized	Periodontal diseases	Rate	1997	861.12	993.78	715.25
227	Incidence	Republic of Korea	Female	Age-standardized	Periodontal diseases	Rate	1997	619.36	765.54	486.56
228	Incidence	Republic of Korea	Both	Age-standardized	Periodontal diseases	Rate	1997	740.25	860.35	626.89
229	Incidence	Republic of Korea	Male	Age-standardized	Periodontal diseases	Rate	1998	850.09	966.55	721.94
230	Incidence	Republic of Korea	Female	Age-standardized	Periodontal diseases	Rate	1998	623.56	771.41	490.37
231	Incidence	Republic of Korea	Both	Age-standardized	Periodontal diseases	Rate	1998	736.72	863.42	622.01
232	Incidence	Republic of Korea	Male	Age-standardized	Periodontal diseases	Rate	1999	840.93	984.33	707.57
233	Incidence	Republic of Korea	Female	Age-standardized	Periodontal diseases	Rate	1999	627.38	779.43	495.50
234	Incidence	Republic of Korea	Both	Age-standardized	Periodontal diseases	Rate	1999	733.96	874.83	603.51
235	Incidence	Republic of Korea	Male	Age-standardized	Periodontal diseases	Rate	2000	837.48	1017.16	663.45
236	Incidence	Republic of Korea	Female	Age-standardized	Periodontal diseases	Rate	2000	630.18	787.11	499.86
237	Incidence	Republic of Korea	Both	Age-standardized	Periodontal diseases	Rate	2000	733.56	892.15	579.10
238	Incidence	Republic of Korea	Male	Age-standardized	Periodontal diseases	Rate	2001	839.43	1017.79	667.23
239	Incidence	Republic of Korea	Female	Age-standardized	Periodontal diseases	Rate	2001	632.55	788.22	499.71
240	Incidence	Republic of Korea	Both	Age-standardized	Periodontal diseases	Rate	2001	735.72	898.01	580.92
241	Incidence	Republic of Korea	Male	Age-standardized	Periodontal diseases	Rate	2002	843.15	1019.22	673.08

242	Incidence	Republic of Korea	Female	Age-standardized	Periodontal diseases	Rate	2002	635.29	791.81	499.30
243	Incidence	Republic of Korea	Both	Age-standardized	Periodontal diseases	Rate	2002	738.95	904.11	583.52
244	Incidence	Republic of Korea	Male	Age-standardized	Periodontal diseases	Rate	2003	847.61	1020.96	679.92
245	Incidence	Republic of Korea	Female	Age-standardized	Periodontal diseases	Rate	2003	638.08	799.51	499.57
246	Incidence	Republic of Korea	Both	Age-standardized	Periodontal diseases	Rate	2003	742.56	909.19	586.95
247	Incidence	Republic of Korea	Male	Age-standardized	Periodontal diseases	Rate	2004	851.73	1015.69	685.04
248	Incidence	Republic of Korea	Female	Age-standardized	Periodontal diseases	Rate	2004	640.56	807.93	499.59
249	Incidence	Republic of Korea	Both	Age-standardized	Periodontal diseases	Rate	2004	745.85	911.86	591.45
250	Incidence	Republic of Korea	Male	Age-standardized	Periodontal diseases	Rate	2005	854.46	1021.04	687.56
251	Incidence	Republic of Korea	Female	Age-standardized	Periodontal diseases	Rate	2005	642.39	817.47	500.76
252	Incidence	Republic of Korea	Both	Age-standardized	Periodontal diseases	Rate	2005	748.13	912.00	593.80
253	Incidence	Republic of Korea	Male	Age-standardized	Periodontal diseases	Rate	2006	856.53	1022.59	688.94
254	Incidence	Republic of Korea	Female	Age-standardized	Periodontal diseases	Rate	2006	644.09	816.29	503.81
255	Incidence	Republic of Korea	Both	Age-standardized	Periodontal diseases	Rate	2006	750.04	917.87	597.45
256	Incidence	Republic of Korea	Male	Age-standardized	Periodontal diseases	Rate	2007	859.08	1031.03	692.59
257	Incidence	Republic of Korea	Female	Age-standardized	Periodontal diseases	Rate	2007	646.21	811.62	508.12
258	Incidence	Republic of Korea	Both	Age-standardized	Periodontal diseases	Rate	2007	752.43	923.29	601.12
259	Incidence	Republic of Korea	Male	Age-standardized	Periodontal diseases	Rate	2008	861.66	1040.13	698.33
260	Incidence	Republic of Korea	Female	Age-standardized	Periodontal diseases	Rate	2008	648.45	814.41	508.82
261	Incidence	Republic of Korea	Both	Age-standardized	Periodontal diseases	Rate	2008	754.93	926.59	605.78
262	Incidence	Republic of Korea	Male	Age-standardized	Periodontal diseases	Rate	2009	863.82	1047.56	702.11
263	Incidence	Republic of Korea	Female	Age-standardized	Periodontal diseases	Rate	2009	650.53	818.32	509.50

264	Incidence	Republic of Korea	Both	Age-standardized	Periodontal diseases	Rate	2009	757.21	927.89	609.28
265	Incidence	Republic of Korea	Male	Age-standardized	Periodontal diseases	Rate	2010	865.10	1052.92	705.44
266	Incidence	Republic of Korea	Female	Age-standardized	Periodontal diseases	Rate	2010	652.16	818.85	512.54
267	Incidence	Republic of Korea	Both	Age-standardized	Periodontal diseases	Rate	2010	758.89	932.24	609.70
268	Incidence	Republic of Korea	Male	Age-standardized	Periodontal diseases	Rate	2011	865.95	1054.05	703.31
269	Incidence	Republic of Korea	Female	Age-standardized	Periodontal diseases	Rate	2011	653.86	819.03	512.43
270	Incidence	Republic of Korea	Both	Age-standardized	Periodontal diseases	Rate	2011	760.45	932.56	611.63
271	Incidence	Republic of Korea	Male	Age-standardized	Periodontal diseases	Rate	2012	867.02	1050.18	700.03
272	Incidence	Republic of Korea	Female	Age-standardized	Periodontal diseases	Rate	2012	656.09	817.84	515.23
273	Incidence	Republic of Korea	Both	Age-standardized	Periodontal diseases	Rate	2012	762.43	935.41	610.94
274	Incidence	Republic of Korea	Male	Age-standardized	Periodontal diseases	Rate	2013	868.20	1045.95	699.33
275	Incidence	Republic of Korea	Female	Age-standardized	Periodontal diseases	Rate	2013	658.44	818.33	518.44
276	Incidence	Republic of Korea	Both	Age-standardized	Periodontal diseases	Rate	2013	764.57	936.60	610.23
277	Incidence	Republic of Korea	Male	Age-standardized	Periodontal diseases	Rate	2014	869.39	1042.13	700.57
278	Incidence	Republic of Korea	Female	Age-standardized	Periodontal diseases	Rate	2014	660.51	819.59	520.13
279	Incidence	Republic of Korea	Both	Age-standardized	Periodontal diseases	Rate	2014	766.60	935.39	613.67
280	Incidence	Republic of Korea	Male	Age-standardized	Periodontal diseases	Rate	2015	870.49	1039.62	702.78
281	Incidence	Republic of Korea	Female	Age-standardized	Periodontal diseases	Rate	2015	661.90	819.82	524.39
282	Incidence	Republic of Korea	Both	Age-standardized	Periodontal diseases	Rate	2015	768.24	933.45	615.75
283	Incidence	Republic of Korea	Male	Age-standardized	Periodontal diseases	Rate	2016	871.96	1043.46	696.82
284	Incidence	Republic of Korea	Female	Age-standardized	Periodontal diseases	Rate	2016	662.93	822.11	524.96
285	Incidence	Republic of Korea	Both	Age-standardized	Periodontal diseases	Rate	2016	769.82	935.97	614.85

286	Incidence	Republic of Korea	Male	Age-standardized	Periodontal diseases	Rate	2017	874.11	1049.36	694.72
287	Incidence	Republic of Korea	Female	Age-standardized	Periodontal diseases	Rate	2017	664.07	825.26	523.89
288	Incidence	Republic of Korea	Both	Age-standardized	Periodontal diseases	Rate	2017	771.73	939.44	614.51
289	Incidence	Republic of Korea	Male	Age-standardized	Periodontal diseases	Rate	2018	876.55	1052.85	696.35
290	Incidence	Republic of Korea	Female	Age-standardized	Periodontal diseases	Rate	2018	665.14	829.44	521.89
291	Incidence	Republic of Korea	Both	Age-standardized	Periodontal diseases	Rate	2018	773.74	943.52	613.70
292	Incidence	Republic of Korea	Male	Age-standardized	Periodontal diseases	Rate	2019	878.91	1054.20	696.94
293	Incidence	Republic of Korea	Female	Age-standardized	Periodontal diseases	Rate	2019	665.98	834.45	519.74
294	Incidence	Republic of Korea	Both	Age-standardized	Periodontal diseases	Rate	2019	775.55	939.15	612.34
295	Incidence	Republic of Korea	Male	Age-standardized	Periodontal diseases	Rate	2020	884.05	1063.83	704.47
296	Incidence	Republic of Korea	Female	Age-standardized	Periodontal diseases	Rate	2020	667.55	830.77	517.07
297	Incidence	Republic of Korea	Both	Age-standardized	Periodontal diseases	Rate	2020	779.12	947.77	610.75
298	Incidence	Republic of Korea	Male	Age-standardized	Periodontal diseases	Rate	2021	884.85	1070.77	716.92
299	Incidence	Republic of Korea	Female	Age-standardized	Periodontal diseases	Rate	2021	666.29	838.82	523.93
300	Incidence	Republic of Korea	Both	Age-standardized	Periodontal diseases	Rate	2021	779.06	953.03	619.49
301	Incidence	Republic of Korea	Male	Age-standardized	Periodontal diseases	Rate	2022	886.48	1063.94	707.01
302	Incidence	Republic of Korea	Female	Age-standardized	Periodontal diseases	Rate	2022	673.96	847.61	518.72
303	Incidence	Republic of Korea	Both	Age-standardized	Periodontal diseases	Rate	2022	783.92	950.68	614.19
304	Incidence	Republic of Korea	Male	Age-standardized	Periodontal diseases	Rate	2023	878.56	1055.37	699.78
305	Incidence	Republic of Korea	Female	Age-standardized	Periodontal diseases	Rate	2023	667.92	839.75	513.71
306	Incidence	Republic of Korea	Both	Age-standardized	Periodontal diseases	Rate	2023	777.06	942.67	608.53
307	Incidence	Russian Federation	Male	Age-standardized	Periodontal diseases	Rate	1990	1100.05	1281.63	882.54

308	Incidence	Russian Federation	Female	Age-standardized	Periodontal diseases	Rate	1990	1104.57	1284.54	889.28
309	Incidence	Russian Federation	Both	Age-standardized	Periodontal diseases	Rate	1990	1102.03	1282.01	885.39
310	Incidence	Russian Federation	Male	Age-standardized	Periodontal diseases	Rate	1991	1097.20	1275.08	881.08
311	Incidence	Russian Federation	Female	Age-standardized	Periodontal diseases	Rate	1991	1100.63	1277.69	888.12
312	Incidence	Russian Federation	Both	Age-standardized	Periodontal diseases	Rate	1991	1098.66	1276.16	883.74
313	Incidence	Russian Federation	Male	Age-standardized	Periodontal diseases	Rate	1992	1094.34	1269.64	879.41
314	Incidence	Russian Federation	Female	Age-standardized	Periodontal diseases	Rate	1992	1096.91	1270.98	888.95
315	Incidence	Russian Federation	Both	Age-standardized	Periodontal diseases	Rate	1992	1095.39	1270.73	883.10
316	Incidence	Russian Federation	Male	Age-standardized	Periodontal diseases	Rate	1993	1091.62	1264.91	877.86
317	Incidence	Russian Federation	Female	Age-standardized	Periodontal diseases	Rate	1993	1093.52	1264.39	890.38
318	Incidence	Russian Federation	Both	Age-standardized	Periodontal diseases	Rate	1993	1092.35	1264.63	884.22
319	Incidence	Russian Federation	Male	Age-standardized	Periodontal diseases	Rate	1994	1089.16	1260.59	878.88
320	Incidence	Russian Federation	Female	Age-standardized	Periodontal diseases	Rate	1994	1090.61	1258.98	892.19
321	Incidence	Russian Federation	Both	Age-standardized	Periodontal diseases	Rate	1994	1089.68	1258.83	885.93
322	Incidence	Russian Federation	Male	Age-standardized	Periodontal diseases	Rate	1995	1087.15	1256.36	880.62
323	Incidence	Russian Federation	Female	Age-standardized	Periodontal diseases	Rate	1995	1088.30	1254.45	894.74
324	Incidence	Russian Federation	Both	Age-standardized	Periodontal diseases	Rate	1995	1087.54	1253.93	888.32
325	Incidence	Russian Federation	Male	Age-standardized	Periodontal diseases	Rate	1996	1084.73	1253.09	880.27
326	Incidence	Russian Federation	Female	Age-standardized	Periodontal diseases	Rate	1996	1086.40	1250.31	893.26
327	Incidence	Russian Federation	Both	Age-standardized	Periodontal diseases	Rate	1996	1085.38	1250.96	887.47
328	Incidence	Russian Federation	Male	Age-standardized	Periodontal diseases	Rate	1997	1081.43	1248.79	878.95
329	Incidence	Russian Federation	Female	Age-standardized	Periodontal diseases	Rate	1997	1084.57	1246.77	891.99

330	Incidence	Russian Federation	Both	Age-standardized	Periodontal diseases	Rate	1997	1082.83	1247.30	885.93
331	Incidence	Russian Federation	Male	Age-standardized	Periodontal diseases	Rate	1998	1077.96	1244.10	877.32
332	Incidence	Russian Federation	Female	Age-standardized	Periodontal diseases	Rate	1998	1082.82	1244.14	890.71
333	Incidence	Russian Federation	Both	Age-standardized	Periodontal diseases	Rate	1998	1080.21	1243.70	884.22
334	Incidence	Russian Federation	Male	Age-standardized	Periodontal diseases	Rate	1999	1075.08	1239.95	875.71
335	Incidence	Russian Federation	Female	Age-standardized	Periodontal diseases	Rate	1999	1081.14	1241.85	889.40
336	Incidence	Russian Federation	Both	Age-standardized	Periodontal diseases	Rate	1999	1077.93	1240.57	882.72
337	Incidence	Russian Federation	Male	Age-standardized	Periodontal diseases	Rate	2000	1073.53	1237.51	875.38
338	Incidence	Russian Federation	Female	Age-standardized	Periodontal diseases	Rate	2000	1079.55	1240.20	888.04
339	Incidence	Russian Federation	Both	Age-standardized	Periodontal diseases	Rate	2000	1076.37	1238.62	881.85
340	Incidence	Russian Federation	Male	Age-standardized	Periodontal diseases	Rate	2001	1072.38	1232.92	884.35
341	Incidence	Russian Federation	Female	Age-standardized	Periodontal diseases	Rate	2001	1075.86	1223.03	908.52
342	Incidence	Russian Federation	Both	Age-standardized	Periodontal diseases	Rate	2001	1073.93	1226.03	901.35
343	Incidence	Russian Federation	Male	Age-standardized	Periodontal diseases	Rate	2002	1070.53	1227.41	894.59
344	Incidence	Russian Federation	Female	Age-standardized	Periodontal diseases	Rate	2002	1069.28	1204.79	931.78
345	Incidence	Russian Federation	Both	Age-standardized	Periodontal diseases	Rate	2002	1069.68	1214.23	908.75
346	Incidence	Russian Federation	Male	Age-standardized	Periodontal diseases	Rate	2003	1068.52	1217.99	898.65
347	Incidence	Russian Federation	Female	Age-standardized	Periodontal diseases	Rate	2003	1061.95	1198.23	938.68
348	Incidence	Russian Federation	Both	Age-standardized	Periodontal diseases	Rate	2003	1064.94	1199.23	926.58
349	Incidence	Russian Federation	Male	Age-standardized	Periodontal diseases	Rate	2004	1066.86	1214.73	902.98
350	Incidence	Russian Federation	Female	Age-standardized	Periodontal diseases	Rate	2004	1056.02	1207.67	934.43
351	Incidence	Russian Federation	Both	Age-standardized	Periodontal diseases	Rate	2004	1061.09	1196.45	932.11

352	Incidence	Russian Federation	Male	Age-standardized	Periodontal diseases	Rate	2005	1066.12	1203.77	910.28
353	Incidence	Russian Federation	Female	Age-standardized	Periodontal diseases	Rate	2005	1053.65	1223.49	923.96
354	Incidence	Russian Federation	Both	Age-standardized	Periodontal diseases	Rate	2005	1059.51	1201.05	934.72
355	Incidence	Russian Federation	Male	Age-standardized	Periodontal diseases	Rate	2006	1062.30	1195.84	929.38
356	Incidence	Russian Federation	Female	Age-standardized	Periodontal diseases	Rate	2006	1054.17	1231.58	920.70
357	Incidence	Russian Federation	Both	Age-standardized	Periodontal diseases	Rate	2006	1057.89	1206.23	934.87
358	Incidence	Russian Federation	Male	Age-standardized	Periodontal diseases	Rate	2007	1053.33	1192.90	926.04
359	Incidence	Russian Federation	Female	Age-standardized	Periodontal diseases	Rate	2007	1055.30	1241.31	915.64
360	Incidence	Russian Federation	Both	Age-standardized	Periodontal diseases	Rate	2007	1054.06	1210.98	925.85
361	Incidence	Russian Federation	Male	Age-standardized	Periodontal diseases	Rate	2008	1042.73	1198.67	909.74
362	Incidence	Russian Federation	Female	Age-standardized	Periodontal diseases	Rate	2008	1056.78	1254.38	906.53
363	Incidence	Russian Federation	Both	Age-standardized	Periodontal diseases	Rate	2008	1049.61	1222.41	913.74
364	Incidence	Russian Federation	Male	Age-standardized	Periodontal diseases	Rate	2009	1033.98	1218.20	885.75
365	Incidence	Russian Federation	Female	Age-standardized	Periodontal diseases	Rate	2009	1058.37	1265.67	898.74
366	Incidence	Russian Federation	Both	Age-standardized	Periodontal diseases	Rate	2009	1046.12	1242.25	893.95
367	Incidence	Russian Federation	Male	Age-standardized	Periodontal diseases	Rate	2010	1030.59	1231.32	857.36
368	Incidence	Russian Federation	Female	Age-standardized	Periodontal diseases	Rate	2010	1059.81	1271.53	892.65
369	Incidence	Russian Federation	Both	Age-standardized	Periodontal diseases	Rate	2010	1045.17	1251.59	874.70
370	Incidence	Russian Federation	Male	Age-standardized	Periodontal diseases	Rate	2011	1031.65	1233.57	858.92
371	Incidence	Russian Federation	Female	Age-standardized	Periodontal diseases	Rate	2011	1061.21	1273.53	894.92
372	Incidence	Russian Federation	Both	Age-standardized	Periodontal diseases	Rate	2011	1046.40	1252.75	875.80
373	Incidence	Russian Federation	Male	Age-standardized	Periodontal diseases	Rate	2012	1033.56	1236.58	861.33

374	Incidence	Russian Federation	Female	Age-standardized	Periodontal diseases	Rate	2012	1062.73	1275.56	897.49
375	Incidence	Russian Federation	Both	Age-standardized	Periodontal diseases	Rate	2012	1048.11	1254.54	877.85
376	Incidence	Russian Federation	Male	Age-standardized	Periodontal diseases	Rate	2013	1035.78	1239.75	864.07
377	Incidence	Russian Federation	Female	Age-standardized	Periodontal diseases	Rate	2013	1064.20	1277.48	900.04
378	Incidence	Russian Federation	Both	Age-standardized	Periodontal diseases	Rate	2013	1049.96	1257.05	880.11
379	Incidence	Russian Federation	Male	Age-standardized	Periodontal diseases	Rate	2014	1037.80	1242.46	865.79
380	Incidence	Russian Federation	Female	Age-standardized	Periodontal diseases	Rate	2014	1065.45	1279.16	902.40
381	Incidence	Russian Federation	Both	Age-standardized	Periodontal diseases	Rate	2014	1051.59	1259.25	882.19
382	Incidence	Russian Federation	Male	Age-standardized	Periodontal diseases	Rate	2015	1039.10	1242.86	866.85
383	Incidence	Russian Federation	Female	Age-standardized	Periodontal diseases	Rate	2015	1066.30	1280.44	904.39
384	Incidence	Russian Federation	Both	Age-standardized	Periodontal diseases	Rate	2015	1052.66	1260.83	883.69
385	Incidence	Russian Federation	Male	Age-standardized	Periodontal diseases	Rate	2016	1039.96	1243.93	867.09
386	Incidence	Russian Federation	Female	Age-standardized	Periodontal diseases	Rate	2016	1066.82	1280.17	904.48
387	Incidence	Russian Federation	Both	Age-standardized	Periodontal diseases	Rate	2016	1053.33	1261.80	884.09
388	Incidence	Russian Federation	Male	Age-standardized	Periodontal diseases	Rate	2017	1040.85	1245.91	867.32
389	Incidence	Russian Federation	Female	Age-standardized	Periodontal diseases	Rate	2017	1067.18	1279.65	904.09
390	Incidence	Russian Federation	Both	Age-standardized	Periodontal diseases	Rate	2017	1053.92	1262.67	884.36
391	Incidence	Russian Federation	Male	Age-standardized	Periodontal diseases	Rate	2018	1041.59	1247.96	867.45
392	Incidence	Russian Federation	Female	Age-standardized	Periodontal diseases	Rate	2018	1067.46	1278.95	903.38
393	Incidence	Russian Federation	Both	Age-standardized	Periodontal diseases	Rate	2018	1054.40	1263.34	884.55
394	Incidence	Russian Federation	Male	Age-standardized	Periodontal diseases	Rate	2019	1042.02	1249.57	867.71
395	Incidence	Russian Federation	Female	Age-standardized	Periodontal diseases	Rate	2019	1067.71	1278.07	904.24

396	Incidence	Russian Federation	Both	Age-standardized	Periodontal diseases	Rate	2019	1054.74	1263.75	886.25
397	Incidence	Russian Federation	Male	Age-standardized	Periodontal diseases	Rate	2020	1040.07	1244.33	865.73
398	Incidence	Russian Federation	Female	Age-standardized	Periodontal diseases	Rate	2020	1068.23	1276.54	905.55
399	Incidence	Russian Federation	Both	Age-standardized	Periodontal diseases	Rate	2020	1054.09	1260.34	883.85
400	Incidence	Russian Federation	Male	Age-standardized	Periodontal diseases	Rate	2021	1038.43	1242.74	865.32
401	Incidence	Russian Federation	Female	Age-standardized	Periodontal diseases	Rate	2021	1069.26	1279.68	907.47
402	Incidence	Russian Federation	Both	Age-standardized	Periodontal diseases	Rate	2021	1053.79	1259.54	884.05
403	Incidence	Russian Federation	Male	Age-standardized	Periodontal diseases	Rate	2022	1041.32	1250.42	869.82
404	Incidence	Russian Federation	Female	Age-standardized	Periodontal diseases	Rate	2022	1067.09	1284.08	902.01
405	Incidence	Russian Federation	Both	Age-standardized	Periodontal diseases	Rate	2022	1054.21	1267.02	884.92
406	Incidence	Russian Federation	Male	Age-standardized	Periodontal diseases	Rate	2023	1033.55	1240.90	863.17
407	Incidence	Russian Federation	Female	Age-standardized	Periodontal diseases	Rate	2023	1059.08	1274.14	894.96
408	Incidence	Russian Federation	Both	Age-standardized	Periodontal diseases	Rate	2023	1046.32	1257.27	878.09
409	Incidence	Japan	Male	Age-standardized	Periodontal diseases	Rate	1990	963.74	1161.59	744.60
410	Incidence	Japan	Female	Age-standardized	Periodontal diseases	Rate	1990	728.63	897.72	537.08
411	Incidence	Japan	Both	Age-standardized	Periodontal diseases	Rate	1990	845.82	1024.10	642.64
412	Incidence	Japan	Male	Age-standardized	Periodontal diseases	Rate	1991	981.13	1174.96	766.52
413	Incidence	Japan	Female	Age-standardized	Periodontal diseases	Rate	1991	753.07	916.02	572.84
414	Incidence	Japan	Both	Age-standardized	Periodontal diseases	Rate	1991	867.10	1039.05	669.23
415	Incidence	Japan	Male	Age-standardized	Periodontal diseases	Rate	1992	995.22	1187.82	784.93
416	Incidence	Japan	Female	Age-standardized	Periodontal diseases	Rate	1992	774.96	922.54	604.54
417	Incidence	Japan	Both	Age-standardized	Periodontal diseases	Rate	1992	885.41	1051.38	699.91

418	Incidence	Japan	Male	Age-standardized	Periodontal diseases	Rate	1993	1005.77	1197.10	799.51
419	Incidence	Japan	Female	Age-standardized	Periodontal diseases	Rate	1993	792.77	931.80	639.71
420	Incidence	Japan	Both	Age-standardized	Periodontal diseases	Rate	1993	899.86	1059.94	724.29
421	Incidence	Japan	Male	Age-standardized	Periodontal diseases	Rate	1994	1012.54	1196.67	808.84
422	Incidence	Japan	Female	Age-standardized	Periodontal diseases	Rate	1994	804.97	932.81	658.98
423	Incidence	Japan	Both	Age-standardized	Periodontal diseases	Rate	1994	909.52	1057.17	743.02
424	Incidence	Japan	Male	Age-standardized	Periodontal diseases	Rate	1995	1015.30	1190.79	814.79
425	Incidence	Japan	Female	Age-standardized	Periodontal diseases	Rate	1995	810.02	933.16	677.07
426	Incidence	Japan	Both	Age-standardized	Periodontal diseases	Rate	1995	913.52	1051.47	754.74
427	Incidence	Japan	Male	Age-standardized	Periodontal diseases	Rate	1996	1013.49	1165.62	834.47
428	Incidence	Japan	Female	Age-standardized	Periodontal diseases	Rate	1996	810.46	932.99	680.05
429	Incidence	Japan	Both	Age-standardized	Periodontal diseases	Rate	1996	912.85	1042.65	767.59
430	Incidence	Japan	Male	Age-standardized	Periodontal diseases	Rate	1997	1007.55	1158.12	844.85
431	Incidence	Japan	Female	Age-standardized	Periodontal diseases	Rate	1997	809.86	930.69	681.16
432	Incidence	Japan	Both	Age-standardized	Periodontal diseases	Rate	1997	909.58	1038.31	770.31
433	Incidence	Japan	Male	Age-standardized	Periodontal diseases	Rate	1998	998.69	1133.31	855.04
434	Incidence	Japan	Female	Age-standardized	Periodontal diseases	Rate	1998	808.26	927.23	678.76
435	Incidence	Japan	Both	Age-standardized	Periodontal diseases	Rate	1998	904.32	1025.75	769.90
436	Incidence	Japan	Male	Age-standardized	Periodontal diseases	Rate	1999	988.14	1112.52	859.40
437	Incidence	Japan	Female	Age-standardized	Periodontal diseases	Rate	1999	805.71	922.58	675.98
438	Incidence	Japan	Both	Age-standardized	Periodontal diseases	Rate	1999	897.74	1020.91	769.65
439	Incidence	Japan	Male	Age-standardized	Periodontal diseases	Rate	2000	977.15	1108.18	844.22

440	Incidence	Japan	Female	Age-standardized	Periodontal diseases	Rate	2000	802.26	917.78	672.34
441	Incidence	Japan	Both	Age-standardized	Periodontal diseases	Rate	2000	890.49	1015.96	760.21
442	Incidence	Japan	Male	Age-standardized	Periodontal diseases	Rate	2001	954.12	1084.42	819.00
443	Incidence	Japan	Female	Age-standardized	Periodontal diseases	Rate	2001	778.82	895.73	653.31
444	Incidence	Japan	Both	Age-standardized	Periodontal diseases	Rate	2001	867.14	995.02	739.38
445	Incidence	Japan	Male	Age-standardized	Periodontal diseases	Rate	2002	914.83	1043.26	780.45
446	Incidence	Japan	Female	Age-standardized	Periodontal diseases	Rate	2002	728.57	847.31	609.25
447	Incidence	Japan	Both	Age-standardized	Periodontal diseases	Rate	2002	822.17	947.00	698.24
448	Incidence	Japan	Male	Age-standardized	Periodontal diseases	Rate	2003	871.54	998.20	743.41
449	Incidence	Japan	Female	Age-standardized	Periodontal diseases	Rate	2003	669.91	787.76	560.66
450	Incidence	Japan	Both	Age-standardized	Periodontal diseases	Rate	2003	770.99	892.02	651.85
451	Incidence	Japan	Male	Age-standardized	Periodontal diseases	Rate	2004	836.53	959.13	709.55
452	Incidence	Japan	Female	Age-standardized	Periodontal diseases	Rate	2004	621.23	738.31	522.90
453	Incidence	Japan	Both	Age-standardized	Periodontal diseases	Rate	2004	729.00	845.16	614.56
454	Incidence	Japan	Male	Age-standardized	Periodontal diseases	Rate	2005	822.04	945.56	692.89
455	Incidence	Japan	Female	Age-standardized	Periodontal diseases	Rate	2005	600.98	727.60	502.05
456	Incidence	Japan	Both	Age-standardized	Periodontal diseases	Rate	2005	711.59	831.47	598.04
457	Incidence	Japan	Male	Age-standardized	Periodontal diseases	Rate	2006	836.43	964.47	709.06
458	Incidence	Japan	Female	Age-standardized	Periodontal diseases	Rate	2006	620.31	745.40	519.48
459	Incidence	Japan	Both	Age-standardized	Periodontal diseases	Rate	2006	728.56	852.19	615.01
460	Incidence	Japan	Male	Age-standardized	Periodontal diseases	Rate	2007	871.06	1004.24	748.25
461	Incidence	Japan	Female	Age-standardized	Periodontal diseases	Rate	2007	666.43	795.29	560.75

462	Incidence	Japan	Both	Age-standardized	Periodontal diseases	Rate	2007	769.15	896.83	654.45
463	Incidence	Japan	Male	Age-standardized	Periodontal diseases	Rate	2008	912.62	1048.93	785.26
464	Incidence	Japan	Female	Age-standardized	Periodontal diseases	Rate	2008	721.77	858.13	604.96
465	Incidence	Japan	Both	Age-standardized	Periodontal diseases	Rate	2008	817.84	952.83	694.46
466	Incidence	Japan	Male	Age-standardized	Periodontal diseases	Rate	2009	947.80	1087.82	809.81
467	Incidence	Japan	Female	Age-standardized	Periodontal diseases	Rate	2009	768.74	912.69	644.64
468	Incidence	Japan	Both	Age-standardized	Periodontal diseases	Rate	2009	859.10	1002.31	729.11
469	Incidence	Japan	Male	Age-standardized	Periodontal diseases	Rate	2010	963.29	1111.39	819.15
470	Incidence	Japan	Female	Age-standardized	Periodontal diseases	Rate	2010	789.76	935.31	661.28
471	Incidence	Japan	Both	Age-standardized	Periodontal diseases	Rate	2010	877.44	1022.65	743.08
472	Incidence	Japan	Male	Age-standardized	Periodontal diseases	Rate	2011	963.81	1112.53	819.92
473	Incidence	Japan	Female	Age-standardized	Periodontal diseases	Rate	2011	791.27	936.84	662.34
474	Incidence	Japan	Both	Age-standardized	Periodontal diseases	Rate	2011	878.48	1023.44	743.91
475	Incidence	Japan	Male	Age-standardized	Periodontal diseases	Rate	2012	963.35	1112.39	819.76
476	Incidence	Japan	Female	Age-standardized	Periodontal diseases	Rate	2012	791.76	936.10	662.48
477	Incidence	Japan	Both	Age-standardized	Periodontal diseases	Rate	2012	878.52	1023.08	743.86
478	Incidence	Japan	Male	Age-standardized	Periodontal diseases	Rate	2013	962.42	1111.66	819.10
479	Incidence	Japan	Female	Age-standardized	Periodontal diseases	Rate	2013	791.63	934.58	662.25
480	Incidence	Japan	Both	Age-standardized	Periodontal diseases	Rate	2013	878.02	1022.15	743.34
481	Incidence	Japan	Male	Age-standardized	Periodontal diseases	Rate	2014	961.55	1111.30	817.64
482	Incidence	Japan	Female	Age-standardized	Periodontal diseases	Rate	2014	791.27	932.74	661.96
483	Incidence	Japan	Both	Age-standardized	Periodontal diseases	Rate	2014	877.42	1021.15	742.57

484	Incidence	Japan	Male	Age-standardized	Periodontal diseases	Rate	2015	961.24	1112.13	816.62
485	Incidence	Japan	Female	Age-standardized	Periodontal diseases	Rate	2015	791.08	931.03	662.15
486	Incidence	Japan	Both	Age-standardized	Periodontal diseases	Rate	2015	877.20	1020.61	742.08
487	Incidence	Japan	Male	Age-standardized	Periodontal diseases	Rate	2016	962.08	1112.74	817.55
488	Incidence	Japan	Female	Age-standardized	Periodontal diseases	Rate	2016	790.61	933.73	661.03
489	Incidence	Japan	Both	Age-standardized	Periodontal diseases	Rate	2016	877.42	1022.01	741.34
490	Incidence	Japan	Male	Age-standardized	Periodontal diseases	Rate	2017	963.72	1115.15	819.91
491	Incidence	Japan	Female	Age-standardized	Periodontal diseases	Rate	2017	789.53	935.97	659.20
492	Incidence	Japan	Both	Age-standardized	Periodontal diseases	Rate	2017	877.76	1023.57	740.49
493	Incidence	Japan	Male	Age-standardized	Periodontal diseases	Rate	2018	965.32	1117.70	821.86
494	Incidence	Japan	Female	Age-standardized	Periodontal diseases	Rate	2018	788.41	937.61	654.88
495	Incidence	Japan	Both	Age-standardized	Periodontal diseases	Rate	2018	878.06	1025.27	740.46
496	Incidence	Japan	Male	Age-standardized	Periodontal diseases	Rate	2019	966.05	1119.39	822.73
497	Incidence	Japan	Female	Age-standardized	Periodontal diseases	Rate	2019	787.81	937.94	650.75
498	Incidence	Japan	Both	Age-standardized	Periodontal diseases	Rate	2019	878.18	1026.89	740.43
499	Incidence	Japan	Male	Age-standardized	Periodontal diseases	Rate	2020	954.05	1131.19	781.10
500	Incidence	Japan	Female	Age-standardized	Periodontal diseases	Rate	2020	785.99	952.54	622.66
501	Incidence	Japan	Both	Age-standardized	Periodontal diseases	Rate	2020	871.29	1042.21	705.95
502	Incidence	Japan	Male	Age-standardized	Periodontal diseases	Rate	2021	955.50	1132.61	780.45
503	Incidence	Japan	Female	Age-standardized	Periodontal diseases	Rate	2021	786.16	947.08	626.67
504	Incidence	Japan	Both	Age-standardized	Periodontal diseases	Rate	2021	872.15	1040.50	704.76
505	Incidence	Japan	Male	Age-standardized	Periodontal diseases	Rate	2022	956.73	1129.76	785.58

506	Incidence	Japan	Female	Age-standardized	Periodontal diseases	Rate	2022	784.17	945.16	627.98
507	Incidence	Japan	Both	Age-standardized	Periodontal diseases	Rate	2022	871.84	1036.03	705.93
508	Incidence	Japan	Male	Age-standardized	Periodontal diseases	Rate	2023	951.39	1123.44	781.05
509	Incidence	Japan	Female	Age-standardized	Periodontal diseases	Rate	2023	779.82	939.86	624.44
510	Incidence	Japan	Both	Age-standardized	Periodontal diseases	Rate	2023	867.04	1030.32	701.69
511	Incidence	People's Republic of China	Male	Age-standardized	Periodontal diseases	Rate	1990	1019.56	1205.88	805.81
512	Incidence	People's Republic of China	Female	Age-standardized	Periodontal diseases	Rate	1990	981.24	1163.72	767.66
513	Incidence	People's Republic of China	Both	Age-standardized	Periodontal diseases	Rate	1990	1001.09	1188.84	788.21
514	Incidence	People's Republic of China	Male	Age-standardized	Periodontal diseases	Rate	1991	978.67	1158.69	769.30
515	Incidence	People's Republic of China	Female	Age-standardized	Periodontal diseases	Rate	1991	942.02	1113.87	736.44
516	Incidence	People's Republic of China	Both	Age-standardized	Periodontal diseases	Rate	1991	961.03	1137.05	753.11
517	Incidence	People's Republic of China	Male	Age-standardized	Periodontal diseases	Rate	1992	941.31	1111.20	736.53
518	Incidence	People's Republic of China	Female	Age-standardized	Periodontal diseases	Rate	1992	906.33	1076.17	703.60
519	Incidence	People's Republic of China	Both	Age-standardized	Periodontal diseases	Rate	1992	924.48	1093.31	720.30
520	Incidence	People's Republic of China	Male	Age-standardized	Periodontal diseases	Rate	1993	909.64	1079.44	704.73
521	Incidence	People's Republic of China	Female	Age-standardized	Periodontal diseases	Rate	1993	876.15	1046.84	676.40
522	Incidence	People's	Both	Age-standardized	Periodontal	Rate	1993	893.53	1063.14	691.12

		Republic of China			diseases					
523	Incidence	People's Republic of China	Male	Age-standardized	Periodontal diseases	Rate	1994	885.72	1060.57	684.08
524	Incidence	People's Republic of China	Female	Age-standardized	Periodontal diseases	Rate	1994	853.37	1023.58	657.60
525	Incidence	People's Republic of China	Both	Age-standardized	Periodontal diseases	Rate	1994	870.15	1045.05	671.36
526	Incidence	People's Republic of China	Male	Age-standardized	Periodontal diseases	Rate	1995	871.74	1048.92	671.72
527	Incidence	People's Republic of China	Female	Age-standardized	Periodontal diseases	Rate	1995	840.03	1012.21	644.75
528	Incidence	People's Republic of China	Both	Age-standardized	Periodontal diseases	Rate	1995	856.48	1032.17	659.32
529	Incidence	People's Republic of China	Male	Age-standardized	Periodontal diseases	Rate	1996	864.36	1040.32	666.97
530	Incidence	People's Republic of China	Female	Age-standardized	Periodontal diseases	Rate	1996	833.25	1002.83	640.51
531	Incidence	People's Republic of China	Both	Age-standardized	Periodontal diseases	Rate	1996	849.38	1022.30	654.73
532	Incidence	People's Republic of China	Male	Age-standardized	Periodontal diseases	Rate	1997	858.68	1034.76	663.33
533	Incidence	People's Republic of China	Female	Age-standardized	Periodontal diseases	Rate	1997	828.57	995.75	637.96
534	Incidence	People's Republic of China	Both	Age-standardized	Periodontal diseases	Rate	1997	844.16	1015.25	651.41
535	Incidence	People's Republic of China	Male	Age-standardized	Periodontal diseases	Rate	1998	854.73	1030.62	660.77
536	Incidence	People's Republic of China	Female	Age-standardized	Periodontal diseases	Rate	1998	825.71	992.51	636.78

537	Incidence	People's Republic of China	Both	Age-standardized	Periodontal diseases	Rate	1998	840.72	1010.51	649.26
538	Incidence	People's Republic of China	Male	Age-standardized	Periodontal diseases	Rate	1999	852.43	1027.19	658.95
539	Incidence	People's Republic of China	Female	Age-standardized	Periodontal diseases	Rate	1999	824.32	990.12	636.58
540	Incidence	People's Republic of China	Both	Age-standardized	Periodontal diseases	Rate	1999	838.84	1008.11	648.24
541	Incidence	People's Republic of China	Male	Age-standardized	Periodontal diseases	Rate	2000	851.82	1026.32	658.22
542	Incidence	People's Republic of China	Female	Age-standardized	Periodontal diseases	Rate	2000	824.19	989.77	637.20
543	Incidence	People's Republic of China	Both	Age-standardized	Periodontal diseases	Rate	2000	838.45	1008.11	648.17
544	Incidence	People's Republic of China	Male	Age-standardized	Periodontal diseases	Rate	2001	865.94	1033.88	675.06
545	Incidence	People's Republic of China	Female	Age-standardized	Periodontal diseases	Rate	2001	835.80	997.47	652.83
546	Incidence	People's Republic of China	Both	Age-standardized	Periodontal diseases	Rate	2001	851.32	1019.44	664.79
547	Incidence	People's Republic of China	Male	Age-standardized	Periodontal diseases	Rate	2002	899.10	1067.11	705.28
548	Incidence	People's Republic of China	Female	Age-standardized	Periodontal diseases	Rate	2002	862.70	1024.37	679.74
549	Incidence	People's Republic of China	Both	Age-standardized	Periodontal diseases	Rate	2002	881.41	1047.41	693.22
550	Incidence	People's Republic of China	Male	Age-standardized	Periodontal diseases	Rate	2003	938.27	1107.73	746.36
551	Incidence	People's Republic of	Female	Age-standardized	Periodontal diseases	Rate	2003	894.33	1057.90	713.01

		China									
552	Incidence	People's Republic of China	Both	Age-standardized	Periodontal diseases	Rate	2003	916.88	1082.67	730.13
553	Incidence	People's Republic of China	Male	Age-standardized	Periodontal diseases	Rate	2004	970.42	1143.09	775.62
554	Incidence	People's Republic of China	Female	Age-standardized	Periodontal diseases	Rate	2004	920.09	1082.51	741.96
555	Incidence	People's Republic of China	Both	Age-standardized	Periodontal diseases	Rate	2004	945.87	1112.21	759.58
556	Incidence	People's Republic of China	Male	Age-standardized	Periodontal diseases	Rate	2005	982.58	1154.59	788.45
557	Incidence	People's Republic of China	Female	Age-standardized	Periodontal diseases	Rate	2005	929.51	1085.73	757.69
558	Incidence	People's Republic of China	Both	Age-standardized	Periodontal diseases	Rate	2005	956.65	1121.71	774.38
559	Incidence	People's Republic of China	Male	Age-standardized	Periodontal diseases	Rate	2006	963.05	1131.46	775.84
560	Incidence	People's Republic of China	Female	Age-standardized	Periodontal diseases	Rate	2006	911.94	1062.94	741.49
561	Incidence	People's Republic of China	Both	Age-standardized	Periodontal diseases	Rate	2006	938.06	1100.31	759.05
562	Incidence	People's Republic of China	Male	Age-standardized	Periodontal diseases	Rate	2007	919.60	1083.66	740.93
563	Incidence	People's Republic of China	Female	Age-standardized	Periodontal diseases	Rate	2007	873.37	1017.19	705.85
564	Incidence	People's Republic of China	Both	Age-standardized	Periodontal diseases	Rate	2007	896.98	1050.31	723.86
565	Incidence	People's Republic of China	Male	Age-standardized	Periodontal diseases	Rate	2008	868.47	1021.17	695.92
566	Incidence	People's	Female	Age-standardized	Periodontal	Rate	2008	828.15	972.94	668.99

		Republic of China			diseases					
567	Incidence	People's Republic of China	Both	Age-standardized	Periodontal diseases	Rate	2008	848.74	995.42	680.38
568	Incidence	People's Republic of China	Male	Age-standardized	Periodontal diseases	Rate	2009	825.85	976.61	657.60
569	Incidence	People's Republic of China	Female	Age-standardized	Periodontal diseases	Rate	2009	790.59	928.33	628.81
570	Incidence	People's Republic of China	Both	Age-standardized	Periodontal diseases	Rate	2009	808.59	953.55	644.20
571	Incidence	People's Republic of China	Male	Age-standardized	Periodontal diseases	Rate	2010	807.91	953.54	641.05
572	Incidence	People's Republic of China	Female	Age-standardized	Periodontal diseases	Rate	2010	774.99	917.28	612.79
573	Incidence	People's Republic of China	Both	Age-standardized	Periodontal diseases	Rate	2010	791.78	934.62	627.20
574	Incidence	People's Republic of China	Male	Age-standardized	Periodontal diseases	Rate	2011	827.25	969.14	667.15
575	Incidence	People's Republic of China	Female	Age-standardized	Periodontal diseases	Rate	2011	795.73	926.93	640.98
576	Incidence	People's Republic of China	Both	Age-standardized	Periodontal diseases	Rate	2011	811.80	947.91	653.77
577	Incidence	People's Republic of China	Male	Age-standardized	Periodontal diseases	Rate	2012	873.76	1001.67	716.13
578	Incidence	People's Republic of China	Female	Age-standardized	Periodontal diseases	Rate	2012	845.00	977.71	696.98
579	Incidence	People's Republic of China	Both	Age-standardized	Periodontal diseases	Rate	2012	859.65	990.13	710.07
580	Incidence	People's Republic of China	Male	Age-standardized	Periodontal diseases	Rate	2013	929.44	1067.58	774.96

581	Incidence	People's Republic of China	Female	Age-standardized	Periodontal diseases	Rate	2013	903.82	1039.95	760.69
582	Incidence	People's Republic of China	Both	Age-standardized	Periodontal diseases	Rate	2013	916.87	1057.35	767.46
583	Incidence	People's Republic of China	Male	Age-standardized	Periodontal diseases	Rate	2014	976.36	1117.22	824.25
584	Incidence	People's Republic of China	Female	Age-standardized	Periodontal diseases	Rate	2014	953.27	1092.25	810.64
585	Incidence	People's Republic of China	Both	Age-standardized	Periodontal diseases	Rate	2014	965.05	1104.63	818.39
586	Incidence	People's Republic of China	Male	Age-standardized	Periodontal diseases	Rate	2015	996.66	1136.43	848.89
587	Incidence	People's Republic of China	Female	Age-standardized	Periodontal diseases	Rate	2015	974.50	1105.50	833.42
588	Incidence	People's Republic of China	Both	Age-standardized	Periodontal diseases	Rate	2015	985.82	1122.53	841.00
589	Incidence	People's Republic of China	Male	Age-standardized	Periodontal diseases	Rate	2016	994.85	1133.27	848.35
590	Incidence	People's Republic of China	Female	Age-standardized	Periodontal diseases	Rate	2016	972.15	1101.87	833.58
591	Incidence	People's Republic of China	Both	Age-standardized	Periodontal diseases	Rate	2016	983.77	1118.44	840.19
592	Incidence	People's Republic of China	Male	Age-standardized	Periodontal diseases	Rate	2017	989.42	1125.07	843.50
593	Incidence	People's Republic of China	Female	Age-standardized	Periodontal diseases	Rate	2017	965.74	1094.32	829.30
594	Incidence	People's Republic of China	Both	Age-standardized	Periodontal diseases	Rate	2017	977.89	1110.19	835.85
595	Incidence	People's Republic of	Male	Age-standardized	Periodontal diseases	Rate	2018	983.42	1116.16	838.31

		China								
596	Incidence	People's Republic of China	Female	Age-standardized	Periodontal diseases	Rate	2018	958.77	1085.09	824.03
597	Incidence	People's Republic of China	Both	Age-standardized	Periodontal diseases	Rate	2018	971.45	1101.26	832.29
598	Incidence	People's Republic of China	Male	Age-standardized	Periodontal diseases	Rate	2019	979.88	1109.44	835.47
599	Incidence	People's Republic of China	Female	Age-standardized	Periodontal diseases	Rate	2019	954.76	1080.63	821.48
600	Incidence	People's Republic of China	Both	Age-standardized	Periodontal diseases	Rate	2019	967.73	1096.43	829.81
601	Incidence	People's Republic of China	Male	Age-standardized	Periodontal diseases	Rate	2020	977.53	1107.26	839.19
602	Incidence	People's Republic of China	Female	Age-standardized	Periodontal diseases	Rate	2020	957.28	1083.99	821.03
603	Incidence	People's Republic of China	Both	Age-standardized	Periodontal diseases	Rate	2020	967.78	1095.50	832.12
604	Incidence	People's Republic of China	Male	Age-standardized	Periodontal diseases	Rate	2021	979.30	1110.31	843.26
605	Incidence	People's Republic of China	Female	Age-standardized	Periodontal diseases	Rate	2021	959.97	1084.55	823.69
606	Incidence	People's Republic of China	Both	Age-standardized	Periodontal diseases	Rate	2021	970.06	1099.29	833.97
607	Incidence	People's Republic of China	Male	Age-standardized	Periodontal diseases	Rate	2022	978.92	1113.14	843.66
608	Incidence	People's Republic of China	Female	Age-standardized	Periodontal diseases	Rate	2022	958.36	1083.08	824.79
609	Incidence	People's Republic of China	Both	Age-standardized	Periodontal diseases	Rate	2022	969.13	1098.10	834.09
610	Incidence	People's	Male	Age-standardized	Periodontal	Rate	2023	959.68	1091.28	827.84

		Republic of China			diseases					
611	Incidence	People's Republic of China	Female	Age-standardized	Periodontal diseases	Rate	2023	939.66	1062.19	809.18
612	Incidence	People's Republic of China	Both	Age-standardized	Periodontal diseases	Rate	2023	950.20	1076.95	819.11
613	Incidence	Republic of Indonesia	Male	Age-standardized	Periodontal diseases	Rate	1990	1087.21	1285.16	853.90
614	Incidence	Republic of Indonesia	Female	Age-standardized	Periodontal diseases	Rate	1990	1193.35	1392.23	953.02
615	Incidence	Republic of Indonesia	Both	Age-standardized	Periodontal diseases	Rate	1990	1141.19	1339.92	901.64
616	Incidence	Republic of Indonesia	Male	Age-standardized	Periodontal diseases	Rate	1991	1091.00	1286.30	857.55
617	Incidence	Republic of Indonesia	Female	Age-standardized	Periodontal diseases	Rate	1991	1195.03	1394.24	954.92
618	Incidence	Republic of Indonesia	Both	Age-standardized	Periodontal diseases	Rate	1991	1143.93	1341.08	904.88
619	Incidence	Republic of Indonesia	Male	Age-standardized	Periodontal diseases	Rate	1992	1094.33	1287.04	860.94
620	Incidence	Republic of Indonesia	Female	Age-standardized	Periodontal diseases	Rate	1992	1196.59	1396.30	956.62
621	Incidence	Republic of Indonesia	Both	Age-standardized	Periodontal diseases	Rate	1992	1146.37	1342.05	907.82
622	Incidence	Republic of Indonesia	Male	Age-standardized	Periodontal diseases	Rate	1993	1097.10	1287.63	863.91
623	Incidence	Republic of Indonesia	Female	Age-standardized	Periodontal diseases	Rate	1993	1197.97	1398.48	958.11
624	Incidence	Republic of Indonesia	Both	Age-standardized	Periodontal diseases	Rate	1993	1148.43	1342.77	910.41
625	Incidence	Republic of Indonesia	Male	Age-standardized	Periodontal diseases	Rate	1994	1099.17	1287.76	866.30
626	Incidence	Republic of Indonesia	Female	Age-standardized	Periodontal diseases	Rate	1994	1199.11	1399.38	959.38
627	Incidence	Republic of Indonesia	Both	Age-standardized	Periodontal diseases	Rate	1994	1150.02	1343.15	912.56
628	Incidence	Republic of Indonesia	Male	Age-standardized	Periodontal diseases	Rate	1995	1100.45	1287.12	867.96
629	Incidence	Republic of Indonesia	Female	Age-standardized	Periodontal diseases	Rate	1995	1199.97	1399.46	960.43
630	Incidence	Republic of Indonesia	Both	Age-standardized	Periodontal diseases	Rate	1995	1151.05	1343.48	914.01

631	Incidence	Republic of Indonesia	Male	Age-standardized	Periodontal diseases	Rate	1996	1101.02	1287.74	868.45
632	Incidence	Republic of Indonesia	Female	Age-standardized	Periodontal diseases	Rate	1996	1200.65	1398.11	961.06
633	Incidence	Republic of Indonesia	Both	Age-standardized	Periodontal diseases	Rate	1996	1151.65	1346.01	915.13
634	Incidence	Republic of Indonesia	Male	Age-standardized	Periodontal diseases	Rate	1997	1101.14	1288.34	868.50
635	Incidence	Republic of Indonesia	Female	Age-standardized	Periodontal diseases	Rate	1997	1201.29	1397.31	961.75
636	Incidence	Republic of Indonesia	Both	Age-standardized	Periodontal diseases	Rate	1997	1151.99	1347.01	916.02
637	Incidence	Republic of Indonesia	Male	Age-standardized	Periodontal diseases	Rate	1998	1100.97	1289.15	867.77
638	Incidence	Republic of Indonesia	Female	Age-standardized	Periodontal diseases	Rate	1998	1201.89	1397.98	962.35
639	Incidence	Republic of Indonesia	Both	Age-standardized	Periodontal diseases	Rate	1998	1152.14	1346.23	916.77
640	Incidence	Republic of Indonesia	Male	Age-standardized	Periodontal diseases	Rate	1999	1100.70	1289.83	867.10
641	Incidence	Republic of Indonesia	Female	Age-standardized	Periodontal diseases	Rate	1999	1202.42	1398.93	962.37
642	Incidence	Republic of Indonesia	Both	Age-standardized	Periodontal diseases	Rate	1999	1152.21	1346.31	917.46
643	Incidence	Republic of Indonesia	Male	Age-standardized	Periodontal diseases	Rate	2000	1100.51	1290.55	865.91
644	Incidence	Republic of Indonesia	Female	Age-standardized	Periodontal diseases	Rate	2000	1202.88	1399.92	962.19
645	Incidence	Republic of Indonesia	Both	Age-standardized	Periodontal diseases	Rate	2000	1152.28	1346.73	918.09
646	Incidence	Republic of Indonesia	Male	Age-standardized	Periodontal diseases	Rate	2001	1100.15	1290.17	866.74
647	Incidence	Republic of Indonesia	Female	Age-standardized	Periodontal diseases	Rate	2001	1203.31	1398.75	961.35
648	Incidence	Republic of Indonesia	Both	Age-standardized	Periodontal diseases	Rate	2001	1152.26	1346.41	917.42
649	Incidence	Republic of Indonesia	Male	Age-standardized	Periodontal diseases	Rate	2002	1099.44	1289.70	868.13
650	Incidence	Republic of Indonesia	Female	Age-standardized	Periodontal diseases	Rate	2002	1203.75	1398.92	959.57
651	Incidence	Republic of Indonesia	Both	Age-standardized	Periodontal diseases	Rate	2002	1152.07	1346.87	916.50
652	Incidence	Republic of Indonesia	Male	Age-standardized	Periodontal diseases	Rate	2003	1098.65	1289.34	869.60

653	Incidence	Republic of Indonesia	Female	Age-standardized	Periodontal diseases	Rate	2003	1204.17	1400.46	957.72
654	Incidence	Republic of Indonesia	Both	Age-standardized	Periodontal diseases	Rate	2003	1151.83	1347.65	915.50
655	Incidence	Republic of Indonesia	Male	Age-standardized	Periodontal diseases	Rate	2004	1098.08	1289.47	870.55
656	Incidence	Republic of Indonesia	Female	Age-standardized	Periodontal diseases	Rate	2004	1204.54	1402.41	955.73
657	Incidence	Republic of Indonesia	Both	Age-standardized	Periodontal diseases	Rate	2004	1151.65	1348.81	914.48
658	Incidence	Republic of Indonesia	Male	Age-standardized	Periodontal diseases	Rate	2005	1098.03	1290.09	871.43
659	Incidence	Republic of Indonesia	Female	Age-standardized	Periodontal diseases	Rate	2005	1204.85	1404.26	953.63
660	Incidence	Republic of Indonesia	Both	Age-standardized	Periodontal diseases	Rate	2005	1151.70	1350.07	913.57
661	Incidence	Republic of Indonesia	Male	Age-standardized	Periodontal diseases	Rate	2006	1098.92	1292.01	871.19
662	Incidence	Republic of Indonesia	Female	Age-standardized	Periodontal diseases	Rate	2006	1205.25	1404.65	957.45
663	Incidence	Republic of Indonesia	Both	Age-standardized	Periodontal diseases	Rate	2006	1152.26	1351.02	915.41
664	Incidence	Republic of Indonesia	Male	Age-standardized	Periodontal diseases	Rate	2007	1100.59	1294.54	872.37
665	Incidence	Republic of Indonesia	Female	Age-standardized	Periodontal diseases	Rate	2007	1205.78	1405.04	961.34
666	Incidence	Republic of Indonesia	Both	Age-standardized	Periodontal diseases	Rate	2007	1153.27	1351.83	917.59
667	Incidence	Republic of Indonesia	Male	Age-standardized	Periodontal diseases	Rate	2008	1102.56	1297.30	874.08
668	Incidence	Republic of Indonesia	Female	Age-standardized	Periodontal diseases	Rate	2008	1206.33	1405.38	965.22
669	Incidence	Republic of Indonesia	Both	Age-standardized	Periodontal diseases	Rate	2008	1154.45	1352.28	919.84
670	Incidence	Republic of Indonesia	Male	Age-standardized	Periodontal diseases	Rate	2009	1104.33	1298.14	874.95
671	Incidence	Republic of Indonesia	Female	Age-standardized	Periodontal diseases	Rate	2009	1206.80	1405.65	968.98
672	Incidence	Republic of Indonesia	Both	Age-standardized	Periodontal diseases	Rate	2009	1155.48	1352.76	921.90
673	Incidence	Republic of Indonesia	Male	Age-standardized	Periodontal diseases	Rate	2010	1105.43	1297.23	874.77
674	Incidence	Republic of Indonesia	Female	Age-standardized	Periodontal diseases	Rate	2010	1207.09	1405.56	970.61

675	Incidence	Republic of Indonesia	Both	Age-standardized	Periodontal diseases	Rate	2010	1156.09	1354.25	923.46
676	Incidence	Republic of Indonesia	Male	Age-standardized	Periodontal diseases	Rate	2011	1106.15	1297.54	875.98
677	Incidence	Republic of Indonesia	Female	Age-standardized	Periodontal diseases	Rate	2011	1207.39	1405.53	971.11
678	Incidence	Republic of Indonesia	Both	Age-standardized	Periodontal diseases	Rate	2011	1156.52	1353.11	923.44
679	Incidence	Republic of Indonesia	Male	Age-standardized	Periodontal diseases	Rate	2012	1107.03	1298.10	876.07
680	Incidence	Republic of Indonesia	Female	Age-standardized	Periodontal diseases	Rate	2012	1207.85	1406.60	969.66
681	Incidence	Republic of Indonesia	Both	Age-standardized	Periodontal diseases	Rate	2012	1157.12	1352.78	923.48
682	Incidence	Republic of Indonesia	Male	Age-standardized	Periodontal diseases	Rate	2013	1107.89	1298.68	877.57
683	Incidence	Republic of Indonesia	Female	Age-standardized	Periodontal diseases	Rate	2013	1208.35	1407.51	968.24
684	Incidence	Republic of Indonesia	Both	Age-standardized	Periodontal diseases	Rate	2013	1157.72	1352.99	923.49
685	Incidence	Republic of Indonesia	Male	Age-standardized	Periodontal diseases	Rate	2014	1108.56	1298.58	878.89
686	Incidence	Republic of Indonesia	Female	Age-standardized	Periodontal diseases	Rate	2014	1208.73	1407.95	966.78
687	Incidence	Republic of Indonesia	Both	Age-standardized	Periodontal diseases	Rate	2014	1158.16	1353.17	923.35
688	Incidence	Republic of Indonesia	Male	Age-standardized	Periodontal diseases	Rate	2015	1108.85	1297.06	879.35
689	Incidence	Republic of Indonesia	Female	Age-standardized	Periodontal diseases	Rate	2015	1208.85	1408.05	965.21
690	Incidence	Republic of Indonesia	Both	Age-standardized	Periodontal diseases	Rate	2015	1158.28	1352.91	922.97
691	Incidence	Republic of Indonesia	Male	Age-standardized	Periodontal diseases	Rate	2016	1108.76	1299.40	879.18
692	Incidence	Republic of Indonesia	Female	Age-standardized	Periodontal diseases	Rate	2016	1208.63	1409.76	966.10
693	Incidence	Republic of Indonesia	Both	Age-standardized	Periodontal diseases	Rate	2016	1158.03	1353.45	923.81
694	Incidence	Republic of Indonesia	Male	Age-standardized	Periodontal diseases	Rate	2017	1108.49	1301.91	878.68
695	Incidence	Republic of Indonesia	Female	Age-standardized	Periodontal diseases	Rate	2017	1208.23	1411.24	966.68
696	Incidence	Republic of Indonesia	Both	Age-standardized	Periodontal diseases	Rate	2017	1157.59	1353.29	923.27

697	Incidence	Republic of Indonesia	Male	Age-standardized	Periodontal diseases	Rate	2018	1108.19	1303.75	877.66
698	Incidence	Republic of Indonesia	Female	Age-standardized	Periodontal diseases	Rate	2018	1207.82	1411.08	967.23
699	Incidence	Republic of Indonesia	Both	Age-standardized	Periodontal diseases	Rate	2018	1157.13	1354.33	922.65
700	Incidence	Republic of Indonesia	Male	Age-standardized	Periodontal diseases	Rate	2019	1108.02	1306.06	876.48
701	Incidence	Republic of Indonesia	Female	Age-standardized	Periodontal diseases	Rate	2019	1207.61	1410.24	968.08
702	Incidence	Republic of Indonesia	Both	Age-standardized	Periodontal diseases	Rate	2019	1156.83	1355.48	922.17
703	Incidence	Republic of Indonesia	Male	Age-standardized	Periodontal diseases	Rate	2020	1108.67	1306.39	876.77
704	Incidence	Republic of Indonesia	Female	Age-standardized	Periodontal diseases	Rate	2020	1208.87	1408.66	974.59
705	Incidence	Republic of Indonesia	Both	Age-standardized	Periodontal diseases	Rate	2020	1157.67	1351.57	925.28
706	Incidence	Republic of Indonesia	Male	Age-standardized	Periodontal diseases	Rate	2021	1100.58	1297.27	866.56
707	Incidence	Republic of Indonesia	Female	Age-standardized	Periodontal diseases	Rate	2021	1209.81	1411.91	965.47
708	Incidence	Republic of Indonesia	Both	Age-standardized	Periodontal diseases	Rate	2021	1153.89	1345.44	913.99
709	Incidence	Republic of Indonesia	Male	Age-standardized	Periodontal diseases	Rate	2022	1106.81	1302.62	873.86
710	Incidence	Republic of Indonesia	Female	Age-standardized	Periodontal diseases	Rate	2022	1208.75	1407.73	967.16
711	Incidence	Republic of Indonesia	Both	Age-standardized	Periodontal diseases	Rate	2022	1156.44	1351.67	921.89
712	Incidence	Republic of Indonesia	Male	Age-standardized	Periodontal diseases	Rate	2023	1091.16	1283.20	863.25
713	Incidence	Republic of Indonesia	Female	Age-standardized	Periodontal diseases	Rate	2023	1191.66	1386.58	953.99
714	Incidence	Republic of Indonesia	Both	Age-standardized	Periodontal diseases	Rate	2023	1139.99	1331.60	909.22
715	Incidence	Republic of Italy	Male	Age-standardized	Periodontal diseases	Rate	1990	872.59	1071.91	641.34
716	Incidence	Republic of Italy	Female	Age-standardized	Periodontal diseases	Rate	1990	986.82	1180.31	767.20
717	Incidence	Republic of Italy	Both	Age-standardized	Periodontal diseases	Rate	1990	930.80	1127.51	706.38
718	Incidence	Republic of Italy	Male	Age-standardized	Periodontal diseases	Rate	1991	874.46	1072.14	645.83

719	Incidence	Republic of Italy	Female	Age-standardized	Periodontal diseases	Rate	1991	987.85	1178.50	769.59
720	Incidence	Republic of Italy	Both	Age-standardized	Periodontal diseases	Rate	1991	932.22	1125.77	709.52
721	Incidence	Republic of Italy	Male	Age-standardized	Periodontal diseases	Rate	1992	875.89	1070.52	649.20
722	Incidence	Republic of Italy	Female	Age-standardized	Periodontal diseases	Rate	1992	988.64	1175.95	773.74
723	Incidence	Republic of Italy	Both	Age-standardized	Periodontal diseases	Rate	1992	933.29	1123.86	711.93
724	Incidence	Republic of Italy	Male	Age-standardized	Periodontal diseases	Rate	1993	876.90	1069.23	652.21
725	Incidence	Republic of Italy	Female	Age-standardized	Periodontal diseases	Rate	1993	989.24	1172.59	779.09
726	Incidence	Republic of Italy	Both	Age-standardized	Periodontal diseases	Rate	1993	934.06	1121.44	714.74
727	Incidence	Republic of Italy	Male	Age-standardized	Periodontal diseases	Rate	1994	877.54	1067.46	654.89
728	Incidence	Republic of Italy	Female	Age-standardized	Periodontal diseases	Rate	1994	989.68	1168.29	787.05
729	Incidence	Republic of Italy	Both	Age-standardized	Periodontal diseases	Rate	1994	934.56	1118.30	719.73
730	Incidence	Republic of Italy	Male	Age-standardized	Periodontal diseases	Rate	1995	877.82	1066.70	657.25
731	Incidence	Republic of Italy	Female	Age-standardized	Periodontal diseases	Rate	1995	989.97	1163.49	794.92
732	Incidence	Republic of Italy	Both	Age-standardized	Periodontal diseases	Rate	1995	934.81	1114.68	724.50
733	Incidence	Republic of Italy	Male	Age-standardized	Periodontal diseases	Rate	1996	877.10	1058.12	664.42
734	Incidence	Republic of Italy	Female	Age-standardized	Periodontal diseases	Rate	1996	989.81	1158.65	804.02
735	Incidence	Republic of Italy	Both	Age-standardized	Periodontal diseases	Rate	1996	934.31	1107.98	735.23
736	Incidence	Republic of Italy	Male	Age-standardized	Periodontal diseases	Rate	1997	875.16	1050.21	675.06
737	Incidence	Republic of Italy	Female	Age-standardized	Periodontal diseases	Rate	1997	989.08	1151.22	812.84
738	Incidence	Republic of Italy	Both	Age-standardized	Periodontal diseases	Rate	1997	932.89	1100.36	744.85
739	Incidence	Republic of Italy	Male	Age-standardized	Periodontal diseases	Rate	1998	872.70	1040.12	680.41
740	Incidence	Republic of Italy	Female	Age-standardized	Periodontal diseases	Rate	1998	988.06	1142.17	816.78

741	Incidence	Republic of Italy	Both	Age-standardized	Periodontal diseases	Rate	1998	931.08	1092.12	751.08
742	Incidence	Republic of Italy	Male	Age-standardized	Periodontal diseases	Rate	1999	870.42	1027.80	684.97
743	Incidence	Republic of Italy	Female	Age-standardized	Periodontal diseases	Rate	1999	987.07	1128.04	818.54
744	Incidence	Republic of Italy	Both	Age-standardized	Periodontal diseases	Rate	1999	929.38	1079.43	757.10
745	Incidence	Republic of Italy	Male	Age-standardized	Periodontal diseases	Rate	2000	868.99	1023.26	689.99
746	Incidence	Republic of Italy	Female	Age-standardized	Periodontal diseases	Rate	2000	986.39	1121.68	820.92
747	Incidence	Republic of Italy	Both	Age-standardized	Periodontal diseases	Rate	2000	928.28	1067.53	762.26
748	Incidence	Republic of Italy	Male	Age-standardized	Periodontal diseases	Rate	2001	867.41	1021.42	690.45
749	Incidence	Republic of Italy	Female	Age-standardized	Periodontal diseases	Rate	2001	985.60	1120.60	820.23
750	Incidence	Republic of Italy	Both	Age-standardized	Periodontal diseases	Rate	2001	927.08	1066.14	762.65
751	Incidence	Republic of Italy	Male	Age-standardized	Periodontal diseases	Rate	2002	864.76	1017.31	691.95
752	Incidence	Republic of Italy	Female	Age-standardized	Periodontal diseases	Rate	2002	984.31	1119.06	819.75
753	Incidence	Republic of Italy	Both	Age-standardized	Periodontal diseases	Rate	2002	925.08	1064.89	762.24
754	Incidence	Republic of Italy	Male	Age-standardized	Periodontal diseases	Rate	2003	861.77	1012.50	692.67
755	Incidence	Republic of Italy	Female	Age-standardized	Periodontal diseases	Rate	2003	982.81	1117.61	819.63
756	Incidence	Republic of Italy	Both	Age-standardized	Periodontal diseases	Rate	2003	922.81	1063.96	761.44
757	Incidence	Republic of Italy	Male	Age-standardized	Periodontal diseases	Rate	2004	859.19	1008.49	693.31
758	Incidence	Republic of Italy	Female	Age-standardized	Periodontal diseases	Rate	2004	981.42	1116.33	819.61
759	Incidence	Republic of Italy	Both	Age-standardized	Periodontal diseases	Rate	2004	920.79	1063.46	761.50
760	Incidence	Republic of Italy	Male	Age-standardized	Periodontal diseases	Rate	2005	857.76	1008.23	693.27
761	Incidence	Republic of Italy	Female	Age-standardized	Periodontal diseases	Rate	2005	980.43	1115.71	819.92
762	Incidence	Republic of Italy	Both	Age-standardized	Periodontal diseases	Rate	2005	919.56	1063.62	761.58

763	Incidence	Republic of Italy	Male	Age-standardized	Periodontal diseases	Rate	2006	857.21	1006.19	692.22
764	Incidence	Republic of Italy	Female	Age-standardized	Periodontal diseases	Rate	2006	979.50	1116.25	825.43
765	Incidence	Republic of Italy	Both	Age-standardized	Periodontal diseases	Rate	2006	918.82	1061.86	762.75
766	Incidence	Republic of Italy	Male	Age-standardized	Periodontal diseases	Rate	2007	856.74	1004.18	691.25
767	Incidence	Republic of Italy	Female	Age-standardized	Periodontal diseases	Rate	2007	978.25	1114.58	829.66
768	Incidence	Republic of Italy	Both	Age-standardized	Periodontal diseases	Rate	2007	917.99	1061.17	763.15
769	Incidence	Republic of Italy	Male	Age-standardized	Periodontal diseases	Rate	2008	856.29	1002.07	690.28
770	Incidence	Republic of Italy	Female	Age-standardized	Periodontal diseases	Rate	2008	976.89	1111.54	828.58
771	Incidence	Republic of Italy	Both	Age-standardized	Periodontal diseases	Rate	2008	917.14	1060.29	763.13
772	Incidence	Republic of Italy	Male	Age-standardized	Periodontal diseases	Rate	2009	855.78	999.97	689.77
773	Incidence	Republic of Italy	Female	Age-standardized	Periodontal diseases	Rate	2009	975.62	1109.89	827.02
774	Incidence	Republic of Italy	Both	Age-standardized	Periodontal diseases	Rate	2009	916.31	1059.12	763.09
775	Incidence	Republic of Italy	Male	Age-standardized	Periodontal diseases	Rate	2010	855.16	997.61	689.29
776	Incidence	Republic of Italy	Female	Age-standardized	Periodontal diseases	Rate	2010	974.66	1108.71	825.70
777	Incidence	Republic of Italy	Both	Age-standardized	Periodontal diseases	Rate	2010	915.56	1058.57	763.20
778	Incidence	Republic of Italy	Male	Age-standardized	Periodontal diseases	Rate	2011	853.64	1003.13	689.35
779	Incidence	Republic of Italy	Female	Age-standardized	Periodontal diseases	Rate	2011	973.42	1110.20	827.96
780	Incidence	Republic of Italy	Both	Age-standardized	Periodontal diseases	Rate	2011	914.21	1057.75	762.59
781	Incidence	Republic of Italy	Male	Age-standardized	Periodontal diseases	Rate	2012	850.98	1006.89	689.65
782	Incidence	Republic of Italy	Female	Age-standardized	Periodontal diseases	Rate	2012	971.50	1111.42	824.97
783	Incidence	Republic of Italy	Both	Age-standardized	Periodontal diseases	Rate	2012	911.91	1056.46	760.97
784	Incidence	Republic of Italy	Male	Age-standardized	Periodontal diseases	Rate	2013	847.90	1001.60	689.23

785	Incidence	Republic of Italy	Female	Age-standardized	Periodontal diseases	Rate	2013	969.32	1110.19	821.59
786	Incidence	Republic of Italy	Both	Age-standardized	Periodontal diseases	Rate	2013	909.23	1055.52	761.07
787	Incidence	Republic of Italy	Male	Age-standardized	Periodontal diseases	Rate	2014	845.10	996.38	687.54
788	Incidence	Republic of Italy	Female	Age-standardized	Periodontal diseases	Rate	2014	967.34	1105.59	818.31
789	Incidence	Republic of Italy	Both	Age-standardized	Periodontal diseases	Rate	2014	906.76	1053.42	760.67
790	Incidence	Republic of Italy	Male	Age-standardized	Periodontal diseases	Rate	2015	843.29	991.94	686.60
791	Incidence	Republic of Italy	Female	Age-standardized	Periodontal diseases	Rate	2015	965.98	1102.49	816.13
792	Incidence	Republic of Italy	Both	Age-standardized	Periodontal diseases	Rate	2015	905.10	1049.88	760.56
793	Incidence	Republic of Italy	Male	Age-standardized	Periodontal diseases	Rate	2016	841.31	984.45	689.30
794	Incidence	Republic of Italy	Female	Age-standardized	Periodontal diseases	Rate	2016	964.60	1101.65	824.81
795	Incidence	Republic of Italy	Both	Age-standardized	Periodontal diseases	Rate	2016	903.33	1037.19	762.22
796	Incidence	Republic of Italy	Male	Age-standardized	Periodontal diseases	Rate	2017	838.19	975.12	691.09
797	Incidence	Republic of Italy	Female	Age-standardized	Periodontal diseases	Rate	2017	962.70	1109.44	829.92
798	Incidence	Republic of Italy	Both	Age-standardized	Periodontal diseases	Rate	2017	900.71	1043.49	769.83
799	Incidence	Republic of Italy	Male	Age-standardized	Periodontal diseases	Rate	2018	834.85	971.60	693.04
800	Incidence	Republic of Italy	Female	Age-standardized	Periodontal diseases	Rate	2018	960.96	1105.41	819.60
801	Incidence	Republic of Italy	Both	Age-standardized	Periodontal diseases	Rate	2018	898.07	1037.51	768.41
802	Incidence	Republic of Italy	Male	Age-standardized	Periodontal diseases	Rate	2019	832.25	973.44	694.13
803	Incidence	Republic of Italy	Female	Age-standardized	Periodontal diseases	Rate	2019	960.06	1120.87	800.93
804	Incidence	Republic of Italy	Both	Age-standardized	Periodontal diseases	Rate	2019	896.22	1042.62	759.33
805	Incidence	Republic of Italy	Male	Age-standardized	Periodontal diseases	Rate	2020	821.43	992.33	661.88
806	Incidence	Republic of Italy	Female	Age-standardized	Periodontal diseases	Rate	2020	961.33	1137.79	784.48

807	Incidence	Republic of Italy	Both	Age-standardized	Periodontal diseases	Rate	2020	891.09	1060.36	726.34
808	Incidence	Republic of Italy	Male	Age-standardized	Periodontal diseases	Rate	2021	822.15	998.15	642.58
809	Incidence	Republic of Italy	Female	Age-standardized	Periodontal diseases	Rate	2021	957.66	1134.35	778.58
810	Incidence	Republic of Italy	Both	Age-standardized	Periodontal diseases	Rate	2021	889.39	1063.93	714.42
811	Incidence	Republic of Italy	Male	Age-standardized	Periodontal diseases	Rate	2022	816.15	993.80	636.30
812	Incidence	Republic of Italy	Female	Age-standardized	Periodontal diseases	Rate	2022	953.02	1130.96	773.64
813	Incidence	Republic of Italy	Both	Age-standardized	Periodontal diseases	Rate	2022	884.01	1057.73	705.90
814	Incidence	Republic of Italy	Male	Age-standardized	Periodontal diseases	Rate	2023	809.25	985.79	630.71
815	Incidence	Republic of Italy	Female	Age-standardized	Periodontal diseases	Rate	2023	944.97	1121.46	766.85
816	Incidence	Republic of Italy	Both	Age-standardized	Periodontal diseases	Rate	2023	876.43	1048.77	699.60
817	Incidence	United States of America	Male	Age-standardized	Periodontal diseases	Rate	1990	1039.52	1212.68	851.01
818	Incidence	United States of America	Female	Age-standardized	Periodontal diseases	Rate	1990	884.10	1063.79	681.94
819	Incidence	United States of America	Both	Age-standardized	Periodontal diseases	Rate	1990	962.02	1130.22	766.54
820	Incidence	United States of America	Male	Age-standardized	Periodontal diseases	Rate	1991	1041.61	1216.90	852.14
821	Incidence	United States of America	Female	Age-standardized	Periodontal diseases	Rate	1991	883.14	1063.36	680.12
822	Incidence	United States of America	Both	Age-standardized	Periodontal diseases	Rate	1991	962.61	1131.39	767.41
823	Incidence	United States of America	Male	Age-standardized	Periodontal diseases	Rate	1992	1042.61	1219.98	852.25
824	Incidence	United States of America	Female	Age-standardized	Periodontal diseases	Rate	1992	882.66	1063.20	678.90
825	Incidence	United States of America	Both	Age-standardized	Periodontal diseases	Rate	1992	962.91	1132.40	767.26
826	Incidence	United States of America	Male	Age-standardized	Periodontal diseases	Rate	1993	1042.73	1221.88	851.54
827	Incidence	United States of America	Female	Age-standardized	Periodontal diseases	Rate	1993	882.56	1063.26	678.15
828	Incidence	United States of America	Both	Age-standardized	Periodontal diseases	Rate	1993	962.96	1133.29	766.80

829	Incidence	United States of America	Male	Age-standardized	Periodontal diseases	Rate	1994	1042.21	1222.92	850.23
830	Incidence	United States of America	Female	Age-standardized	Periodontal diseases	Rate	1994	882.74	1063.64	677.74
831	Incidence	United States of America	Both	Age-standardized	Periodontal diseases	Rate	1994	962.83	1134.07	765.98
832	Incidence	United States of America	Male	Age-standardized	Periodontal diseases	Rate	1995	1041.27	1223.25	848.54
833	Incidence	United States of America	Female	Age-standardized	Periodontal diseases	Rate	1995	883.11	1064.19	677.56
834	Incidence	United States of America	Both	Age-standardized	Periodontal diseases	Rate	1995	962.59	1134.76	765.03
835	Incidence	United States of America	Male	Age-standardized	Periodontal diseases	Rate	1996	1036.54	1217.93	842.19
836	Incidence	United States of America	Female	Age-standardized	Periodontal diseases	Rate	1996	886.27	1065.04	682.21
837	Incidence	United States of America	Both	Age-standardized	Periodontal diseases	Rate	1996	961.81	1136.23	762.50
838	Incidence	United States of America	Male	Age-standardized	Periodontal diseases	Rate	1997	1025.82	1206.63	825.73
839	Incidence	United States of America	Female	Age-standardized	Periodontal diseases	Rate	1997	893.17	1070.71	689.32
840	Incidence	United States of America	Both	Age-standardized	Periodontal diseases	Rate	1997	959.89	1134.10	757.68
841	Incidence	United States of America	Male	Age-standardized	Periodontal diseases	Rate	1998	1011.14	1194.92	802.57
842	Incidence	United States of America	Female	Age-standardized	Periodontal diseases	Rate	1998	901.22	1077.94	696.69
843	Incidence	United States of America	Both	Age-standardized	Periodontal diseases	Rate	1998	956.54	1130.35	750.28
844	Incidence	United States of America	Male	Age-standardized	Periodontal diseases	Rate	1999	994.50	1179.10	783.02
845	Incidence	United States of America	Female	Age-standardized	Periodontal diseases	Rate	1999	907.81	1083.50	702.77
846	Incidence	United States of America	Both	Age-standardized	Periodontal diseases	Rate	1999	951.48	1124.44	745.46
847	Incidence	United States of America	Male	Age-standardized	Periodontal diseases	Rate	2000	977.94	1161.15	764.80
848	Incidence	United States of America	Female	Age-standardized	Periodontal diseases	Rate	2000	910.33	1084.94	707.03
849	Incidence	United States of America	Both	Age-standardized	Periodontal diseases	Rate	2000	944.43	1115.73	738.99
850	Incidence	United States of America	Male	Age-standardized	Periodontal diseases	Rate	2001	956.54	1129.80	754.29

851	Incidence	United States of America	Female	Age-standardized	Periodontal diseases	Rate	2001	899.71	1068.75	706.95
852	Incidence	United States of America	Both	Age-standardized	Periodontal diseases	Rate	2001	928.41	1095.94	731.07
853	Incidence	United States of America	Male	Age-standardized	Periodontal diseases	Rate	2002	927.24	1089.77	734.05
854	Incidence	United States of America	Female	Age-standardized	Periodontal diseases	Rate	2002	873.70	1030.59	691.87
855	Incidence	United States of America	Both	Age-standardized	Periodontal diseases	Rate	2002	900.73	1062.61	713.35
856	Incidence	United States of America	Male	Age-standardized	Periodontal diseases	Rate	2003	894.73	1052.69	710.17
857	Incidence	United States of America	Female	Age-standardized	Periodontal diseases	Rate	2003	839.86	991.91	668.67
858	Incidence	United States of America	Both	Age-standardized	Periodontal diseases	Rate	2003	867.51	1019.65	689.91
859	Incidence	United States of America	Male	Age-standardized	Periodontal diseases	Rate	2004	863.68	1016.08	686.86
860	Incidence	United States of America	Female	Age-standardized	Periodontal diseases	Rate	2004	805.72	949.60	645.42
861	Incidence	United States of America	Both	Age-standardized	Periodontal diseases	Rate	2004	834.88	983.91	666.44
862	Incidence	United States of America	Male	Age-standardized	Periodontal diseases	Rate	2005	838.73	984.84	670.27
863	Incidence	United States of America	Female	Age-standardized	Periodontal diseases	Rate	2005	778.80	914.25	628.83
864	Incidence	United States of America	Both	Age-standardized	Periodontal diseases	Rate	2005	808.90	950.21	649.63
865	Incidence	United States of America	Male	Age-standardized	Periodontal diseases	Rate	2006	810.63	953.99	644.25
866	Incidence	United States of America	Female	Age-standardized	Periodontal diseases	Rate	2006	749.01	879.34	601.29
867	Incidence	United States of America	Both	Age-standardized	Periodontal diseases	Rate	2006	779.88	918.93	626.87
868	Incidence	United States of America	Male	Age-standardized	Periodontal diseases	Rate	2007	773.05	914.45	614.71
869	Incidence	United States of America	Female	Age-standardized	Periodontal diseases	Rate	2007	708.03	835.13	563.66
870	Incidence	United States of America	Both	Age-standardized	Periodontal diseases	Rate	2007	740.48	876.24	591.55
871	Incidence	United States of America	Male	Age-standardized	Periodontal diseases	Rate	2008	735.14	873.95	580.63
872	Incidence	United States of America	Female	Age-standardized	Periodontal diseases	Rate	2008	666.18	791.40	524.95

873	Incidence	United States of America	Both	Age-standardized	Periodontal diseases	Rate	2008	700.47	832.90	554.46
874	Incidence	United States of America	Male	Age-standardized	Periodontal diseases	Rate	2009	705.95	841.61	551.60
875	Incidence	United States of America	Female	Age-standardized	Periodontal diseases	Rate	2009	633.74	758.52	495.59
876	Incidence	United States of America	Both	Age-standardized	Periodontal diseases	Rate	2009	669.56	798.23	524.54
877	Incidence	United States of America	Male	Age-standardized	Periodontal diseases	Rate	2010	694.57	828.99	538.82
878	Incidence	United States of America	Female	Age-standardized	Periodontal diseases	Rate	2010	620.99	744.98	482.90
879	Incidence	United States of America	Both	Age-standardized	Periodontal diseases	Rate	2010	657.46	786.66	511.17
880	Incidence	United States of America	Male	Age-standardized	Periodontal diseases	Rate	2011	720.64	853.81	569.36
881	Incidence	United States of America	Female	Age-standardized	Periodontal diseases	Rate	2011	646.37	766.17	509.59
882	Incidence	United States of America	Both	Age-standardized	Periodontal diseases	Rate	2011	683.25	808.32	540.25
883	Incidence	United States of America	Male	Age-standardized	Periodontal diseases	Rate	2012	782.06	917.75	632.47
884	Incidence	United States of America	Female	Age-standardized	Periodontal diseases	Rate	2012	706.22	825.66	568.92
885	Incidence	United States of America	Both	Age-standardized	Periodontal diseases	Rate	2012	744.02	871.40	601.25
886	Incidence	United States of America	Male	Age-standardized	Periodontal diseases	Rate	2013	855.50	992.27	702.65
887	Incidence	United States of America	Female	Age-standardized	Periodontal diseases	Rate	2013	777.82	902.55	638.52
888	Incidence	United States of America	Both	Age-standardized	Periodontal diseases	Rate	2013	816.70	947.47	673.57
889	Incidence	United States of America	Male	Age-standardized	Periodontal diseases	Rate	2014	917.62	1053.84	760.11
890	Incidence	United States of America	Female	Age-standardized	Periodontal diseases	Rate	2014	838.42	968.65	696.76
891	Incidence	United States of America	Both	Age-standardized	Periodontal diseases	Rate	2014	878.19	1014.68	734.80
892	Incidence	United States of America	Male	Age-standardized	Periodontal diseases	Rate	2015	945.09	1081.89	788.44
893	Incidence	United States of America	Female	Age-standardized	Periodontal diseases	Rate	2015	865.28	996.30	725.84
894	Incidence	United States of America	Both	Age-standardized	Periodontal diseases	Rate	2015	905.43	1038.71	764.52

895	Incidence	United States of America	Male	Age-standardized	Periodontal diseases	Rate	2016	945.62	1082.12	800.12
896	Incidence	United States of America	Female	Age-standardized	Periodontal diseases	Rate	2016	866.64	996.94	728.37
897	Incidence	United States of America	Both	Age-standardized	Periodontal diseases	Rate	2016	906.42	1038.06	766.75
898	Incidence	United States of America	Male	Age-standardized	Periodontal diseases	Rate	2017	943.72	1077.22	801.66
899	Incidence	United States of America	Female	Age-standardized	Periodontal diseases	Rate	2017	866.48	997.79	728.42
900	Incidence	United States of America	Both	Age-standardized	Periodontal diseases	Rate	2017	905.42	1038.86	765.55
901	Incidence	United States of America	Male	Age-standardized	Periodontal diseases	Rate	2018	941.17	1077.14	800.35
902	Incidence	United States of America	Female	Age-standardized	Periodontal diseases	Rate	2018	865.65	998.29	727.44
903	Incidence	United States of America	Both	Age-standardized	Periodontal diseases	Rate	2018	903.77	1038.65	765.37
904	Incidence	United States of America	Male	Age-standardized	Periodontal diseases	Rate	2019	939.71	1072.69	800.31
905	Incidence	United States of America	Female	Age-standardized	Periodontal diseases	Rate	2019	864.96	998.74	727.13
906	Incidence	United States of America	Both	Age-standardized	Periodontal diseases	Rate	2019	902.72	1037.79	763.98
907	Incidence	United States of America	Male	Age-standardized	Periodontal diseases	Rate	2020	934.47	1066.08	801.77
908	Incidence	United States of America	Female	Age-standardized	Periodontal diseases	Rate	2020	858.19	989.46	724.00
909	Incidence	United States of America	Both	Age-standardized	Periodontal diseases	Rate	2020	896.76	1028.34	763.57
910	Incidence	United States of America	Male	Age-standardized	Periodontal diseases	Rate	2021	933.93	1066.11	796.50
911	Incidence	United States of America	Female	Age-standardized	Periodontal diseases	Rate	2021	860.10	986.10	734.11
912	Incidence	United States of America	Both	Age-standardized	Periodontal diseases	Rate	2021	897.46	1027.34	767.12
913	Incidence	United States of America	Male	Age-standardized	Periodontal diseases	Rate	2022	933.44	1062.81	798.43
914	Incidence	United States of America	Female	Age-standardized	Periodontal diseases	Rate	2022	861.37	990.06	731.00
915	Incidence	United States of America	Both	Age-standardized	Periodontal diseases	Rate	2022	897.87	1026.93	767.86
916	Incidence	United States of America	Male	Age-standardized	Periodontal diseases	Rate	2023	927.18	1055.52	792.91

917	Incidence	United States of America	Female	Age-standardized	Periodontal diseases	Rate	2023	855.64	983.33	726.15
918	Incidence	United States of America	Both	Age-standardized	Periodontal diseases	Rate	2023	891.90	1020.41	762.62
919	Incidence	Federal Republic of Germany	Male	Age-standardized	Periodontal diseases	Rate	1990	1356.74	1500.30	1193.13
920	Incidence	Federal Republic of Germany	Female	Age-standardized	Periodontal diseases	Rate	1990	1261.89	1390.36	1085.51
921	Incidence	Federal Republic of Germany	Both	Age-standardized	Periodontal diseases	Rate	1990	1310.46	1446.16	1151.27
922	Incidence	Federal Republic of Germany	Male	Age-standardized	Periodontal diseases	Rate	1991	1402.08	1539.60	1246.07
923	Incidence	Federal Republic of Germany	Female	Age-standardized	Periodontal diseases	Rate	1991	1318.35	1430.99	1181.42
924	Incidence	Federal Republic of Germany	Both	Age-standardized	Periodontal diseases	Rate	1991	1361.46	1474.73	1210.17
925	Incidence	Federal Republic of Germany	Male	Age-standardized	Periodontal diseases	Rate	1992	1443.33	1573.12	1289.03
926	Incidence	Federal Republic of Germany	Female	Age-standardized	Periodontal diseases	Rate	1992	1370.10	1459.18	1260.71
927	Incidence	Federal Republic of Germany	Both	Age-standardized	Periodontal diseases	Rate	1992	1408.01	1504.43	1275.53
928	Incidence	Federal Republic of Germany	Male	Age-standardized	Periodontal diseases	Rate	1993	1477.33	1602.08	1322.35
929	Incidence	Federal Republic of Germany	Female	Age-standardized	Periodontal diseases	Rate	1993	1412.93	1515.49	1304.09
930	Incidence	Federal Republic of Germany	Both	Age-standardized	Periodontal diseases	Rate	1993	1446.41	1526.18	1335.35
931	Incidence	Federal Republic of Germany	Male	Age-standardized	Periodontal diseases	Rate	1994	1500.91	1626.61	1343.89
932	Incidence	Federal	Female	Age-standardized	Periodontal	Rate	1994	1442.63	1559.85	1340.61

		Republic of Germany			diseases					
933	Incidence	Federal Republic of Germany	Both	Age-standardized	Periodontal diseases	Rate	1994	1473.00	1564.29	1375.31
934	Incidence	Federal Republic of Germany	Male	Age-standardized	Periodontal diseases	Rate	1995	1510.89	1639.10	1352.28
935	Incidence	Federal Republic of Germany	Female	Age-standardized	Periodontal diseases	Rate	1995	1454.99	1588.17	1336.23
936	Incidence	Federal Republic of Germany	Both	Age-standardized	Periodontal diseases	Rate	1995	1484.12	1581.99	1370.63
937	Incidence	Federal Republic of Germany	Male	Age-standardized	Periodontal diseases	Rate	1996	1512.78	1611.11	1374.75
938	Incidence	Federal Republic of Germany	Female	Age-standardized	Periodontal diseases	Rate	1996	1457.15	1588.69	1338.63
939	Incidence	Federal Republic of Germany	Both	Age-standardized	Periodontal diseases	Rate	1996	1486.09	1576.31	1378.65
940	Incidence	Federal Republic of Germany	Male	Age-standardized	Periodontal diseases	Rate	1997	1513.94	1599.44	1394.61
941	Incidence	Federal Republic of Germany	Female	Age-standardized	Periodontal diseases	Rate	1997	1458.93	1592.49	1340.47
942	Incidence	Federal Republic of Germany	Both	Age-standardized	Periodontal diseases	Rate	1997	1487.47	1584.42	1383.79
943	Incidence	Federal Republic of Germany	Male	Age-standardized	Periodontal diseases	Rate	1998	1513.95	1611.00	1415.41
944	Incidence	Federal Republic of Germany	Female	Age-standardized	Periodontal diseases	Rate	1998	1460.16	1597.01	1342.41
945	Incidence	Federal Republic of Germany	Both	Age-standardized	Periodontal diseases	Rate	1998	1487.96	1594.21	1387.81
946	Incidence	Federal Republic of Germany	Male	Age-standardized	Periodontal diseases	Rate	1999	1512.43	1626.58	1408.13

947	Incidence	Federal Republic of Germany	Female	Age-standardized	Periodontal diseases	Rate	1999	1460.65	1600.26	1339.87
948	Incidence	Federal Republic of Germany	Both	Age-standardized	Periodontal diseases	Rate	1999	1487.29	1607.16	1377.43
949	Incidence	Federal Republic of Germany	Male	Age-standardized	Periodontal diseases	Rate	2000	1508.98	1640.21	1377.06
950	Incidence	Federal Republic of Germany	Female	Age-standardized	Periodontal diseases	Rate	2000	1460.23	1599.95	1337.47
951	Incidence	Federal Republic of Germany	Both	Age-standardized	Periodontal diseases	Rate	2000	1485.19	1617.05	1361.17
952	Incidence	Federal Republic of Germany	Male	Age-standardized	Periodontal diseases	Rate	2001	1474.76	1597.63	1351.68
953	Incidence	Federal Republic of Germany	Female	Age-standardized	Periodontal diseases	Rate	2001	1420.05	1543.55	1304.26
954	Incidence	Federal Republic of Germany	Both	Age-standardized	Periodontal diseases	Rate	2001	1448.02	1564.13	1333.34
955	Incidence	Federal Republic of Germany	Male	Age-standardized	Periodontal diseases	Rate	2002	1399.75	1505.34	1289.19
956	Incidence	Federal Republic of Germany	Female	Age-standardized	Periodontal diseases	Rate	2002	1325.61	1434.12	1211.15
957	Incidence	Federal Republic of Germany	Both	Age-standardized	Periodontal diseases	Rate	2002	1363.57	1468.63	1255.09
958	Incidence	Federal Republic of Germany	Male	Age-standardized	Periodontal diseases	Rate	2003	1311.79	1416.48	1211.58
959	Incidence	Federal Republic of Germany	Female	Age-standardized	Periodontal diseases	Rate	2003	1213.22	1314.30	1106.75
960	Incidence	Federal Republic of Germany	Both	Age-standardized	Periodontal diseases	Rate	2003	1263.74	1361.16	1164.33
961	Incidence	Federal Republic of	Male	Age-standardized	Periodontal diseases	Rate	2004	1238.73	1335.83	1140.80

		Germany								
962	Incidence	Federal Republic of Germany	Female	Age-standardized	Periodontal diseases	Rate	2004	1119.23	1225.30	1028.04
963	Incidence	Federal Republic of Germany	Both	Age-standardized	Periodontal diseases	Rate	2004	1180.48	1271.70	1080.61
964	Incidence	Federal Republic of Germany	Male	Age-standardized	Periodontal diseases	Rate	2005	1208.41	1311.32	1112.64
965	Incidence	Federal Republic of Germany	Female	Age-standardized	Periodontal diseases	Rate	2005	1079.96	1192.07	981.91
966	Incidence	Federal Republic of Germany	Both	Age-standardized	Periodontal diseases	Rate	2005	1145.73	1248.14	1046.93
967	Incidence	Federal Republic of Germany	Male	Age-standardized	Periodontal diseases	Rate	2006	1230.67	1333.65	1136.24
968	Incidence	Federal Republic of Germany	Female	Age-standardized	Periodontal diseases	Rate	2006	1110.76	1219.03	1016.30
969	Incidence	Federal Republic of Germany	Both	Age-standardized	Periodontal diseases	Rate	2006	1172.06	1270.53	1075.45
970	Incidence	Federal Republic of Germany	Male	Age-standardized	Periodontal diseases	Rate	2007	1283.11	1396.28	1182.61
971	Incidence	Federal Republic of Germany	Female	Age-standardized	Periodontal diseases	Rate	2007	1183.91	1292.20	1081.95
972	Incidence	Federal Republic of Germany	Both	Age-standardized	Periodontal diseases	Rate	2007	1234.52	1345.46	1139.14
973	Incidence	Federal Republic of Germany	Male	Age-standardized	Periodontal diseases	Rate	2008	1345.19	1466.01	1236.31
974	Incidence	Federal Republic of Germany	Female	Age-standardized	Periodontal diseases	Rate	2008	1271.15	1397.21	1166.30
975	Incidence	Federal Republic of Germany	Both	Age-standardized	Periodontal diseases	Rate	2008	1308.84	1436.71	1205.60
976	Incidence	Federal	Male	Age-standardized	Periodontal	Rate	2009	1396.42	1540.29	1272.02

		Republic of Germany			diseases					
977	Incidence	Federal Republic of Germany	Female	Age-standardized	Periodontal diseases	Rate	2009	1344.21	1496.85	1228.46
978	Incidence	Federal Republic of Germany	Both	Age-standardized	Periodontal diseases	Rate	2009	1370.72	1513.91	1251.06
979	Incidence	Federal Republic of Germany	Male	Age-standardized	Periodontal diseases	Rate	2010	1416.25	1576.74	1276.61
980	Incidence	Federal Republic of Germany	Female	Age-standardized	Periodontal diseases	Rate	2010	1374.83	1553.47	1242.83
981	Incidence	Federal Republic of Germany	Both	Age-standardized	Periodontal diseases	Rate	2010	1395.85	1557.52	1265.37
982	Incidence	Federal Republic of Germany	Male	Age-standardized	Periodontal diseases	Rate	2011	1406.41	1565.17	1267.85
983	Incidence	Federal Republic of Germany	Female	Age-standardized	Periodontal diseases	Rate	2011	1366.43	1542.01	1239.69
984	Incidence	Federal Republic of Germany	Both	Age-standardized	Periodontal diseases	Rate	2011	1386.74	1548.59	1258.69
985	Incidence	Federal Republic of Germany	Male	Age-standardized	Periodontal diseases	Rate	2012	1386.79	1548.99	1250.10
986	Incidence	Federal Republic of Germany	Female	Age-standardized	Periodontal diseases	Rate	2012	1346.39	1510.05	1216.70
987	Incidence	Federal Republic of Germany	Both	Age-standardized	Periodontal diseases	Rate	2012	1366.95	1529.83	1234.89
988	Incidence	Federal Republic of Germany	Male	Age-standardized	Periodontal diseases	Rate	2013	1364.14	1535.02	1227.29
989	Incidence	Federal Republic of Germany	Female	Age-standardized	Periodontal diseases	Rate	2013	1322.40	1496.06	1189.37
990	Incidence	Federal Republic of Germany	Both	Age-standardized	Periodontal diseases	Rate	2013	1343.68	1508.88	1205.13

991	Incidence	Federal Republic of Germany	Male	Age-standardized	Periodontal diseases	Rate	2014	1345.17	1525.55	1199.64
992	Incidence	Federal Republic of Germany	Female	Age-standardized	Periodontal diseases	Rate	2014	1302.13	1476.14	1159.48
993	Incidence	Federal Republic of Germany	Both	Age-standardized	Periodontal diseases	Rate	2014	1324.13	1500.41	1180.50
994	Incidence	Federal Republic of Germany	Male	Age-standardized	Periodontal diseases	Rate	2015	1336.64	1529.54	1180.22
995	Incidence	Federal Republic of Germany	Female	Age-standardized	Periodontal diseases	Rate	2015	1293.26	1476.74	1141.60
996	Incidence	Federal Republic of Germany	Both	Age-standardized	Periodontal diseases	Rate	2015	1315.50	1502.00	1162.54
997	Incidence	Federal Republic of Germany	Male	Age-standardized	Periodontal diseases	Rate	2016	1336.42	1522.25	1177.53
998	Incidence	Federal Republic of Germany	Female	Age-standardized	Periodontal diseases	Rate	2016	1293.82	1475.79	1139.16
999	Incidence	Federal Republic of Germany	Both	Age-standardized	Periodontal diseases	Rate	2016	1315.71	1499.38	1161.20
1000	Incidence	Federal Republic of Germany	Male	Age-standardized	Periodontal diseases	Rate	2017	1337.34	1518.52	1179.17
1001	Incidence	Federal Republic of Germany	Female	Age-standardized	Periodontal diseases	Rate	2017	1295.90	1492.34	1139.03
1002	Incidence	Federal Republic of Germany	Both	Age-standardized	Periodontal diseases	Rate	2017	1317.20	1502.89	1161.85
1003	Incidence	Federal Republic of Germany	Male	Age-standardized	Periodontal diseases	Rate	2018	1338.54	1522.68	1177.64
1004	Incidence	Federal Republic of Germany	Female	Age-standardized	Periodontal diseases	Rate	2018	1298.23	1493.72	1139.92
1005	Incidence	Federal Republic of	Both	Age-standardized	Periodontal diseases	Rate	2018	1318.95	1502.00	1161.26

		Germany								
1006	Incidence	Federal Republic of Germany	Male	Age-standardized	Periodontal diseases	Rate	2019	1339.17	1522.70	1171.28
1007	Incidence	Federal Republic of Germany	Female	Age-standardized	Periodontal diseases	Rate	2019	1299.55	1496.98	1143.03
1008	Incidence	Federal Republic of Germany	Both	Age-standardized	Periodontal diseases	Rate	2019	1319.89	1502.72	1160.33
1009	Incidence	Federal Republic of Germany	Male	Age-standardized	Periodontal diseases	Rate	2020	1338.60	1519.63	1175.73
1010	Incidence	Federal Republic of Germany	Female	Age-standardized	Periodontal diseases	Rate	2020	1296.03	1485.05	1136.35
1011	Incidence	Federal Republic of Germany	Both	Age-standardized	Periodontal diseases	Rate	2020	1317.93	1494.98	1163.18
1012	Incidence	Federal Republic of Germany	Male	Age-standardized	Periodontal diseases	Rate	2021	1337.68	1521.89	1175.42
1013	Incidence	Federal Republic of Germany	Female	Age-standardized	Periodontal diseases	Rate	2021	1293.29	1473.91	1140.30
1014	Incidence	Federal Republic of Germany	Both	Age-standardized	Periodontal diseases	Rate	2021	1316.08	1494.29	1160.33
1015	Incidence	Federal Republic of Germany	Male	Age-standardized	Periodontal diseases	Rate	2022	1335.50	1521.74	1186.04
1016	Incidence	Federal Republic of Germany	Female	Age-standardized	Periodontal diseases	Rate	2022	1297.34	1497.30	1134.37
1017	Incidence	Federal Republic of Germany	Both	Age-standardized	Periodontal diseases	Rate	2022	1316.99	1497.94	1162.32
1018	Incidence	Federal Republic of Germany	Male	Age-standardized	Periodontal diseases	Rate	2023	1327.24	1512.34	1178.94
1019	Incidence	Federal Republic of Germany	Female	Age-standardized	Periodontal diseases	Rate	2023	1289.29	1488.08	1127.23
1020	Incidence	Federal	Both	Age-standardized	Periodontal	Rate	2023	1308.84	1489.14	1155.09

		Republic of Germany			diseases					
1021	Incidence	United Mexican States	Male	Age-standardized	Periodontal diseases	Rate	1990	1266.36	1461.52	1003.92
1022	Incidence	United Mexican States	Female	Age-standardized	Periodontal diseases	Rate	1990	1233.53	1435.71	984.27
1023	Incidence	United Mexican States	Both	Age-standardized	Periodontal diseases	Rate	1990	1249.34	1448.04	993.77
1024	Incidence	United Mexican States	Male	Age-standardized	Periodontal diseases	Rate	1991	1266.65	1459.17	1005.43
1025	Incidence	United Mexican States	Female	Age-standardized	Periodontal diseases	Rate	1991	1232.50	1432.17	982.92
1026	Incidence	United Mexican States	Both	Age-standardized	Periodontal diseases	Rate	1991	1248.96	1446.02	993.79
1027	Incidence	United Mexican States	Male	Age-standardized	Periodontal diseases	Rate	1992	1266.89	1459.34	1006.85
1028	Incidence	United Mexican States	Female	Age-standardized	Periodontal diseases	Rate	1992	1231.78	1428.70	981.87
1029	Incidence	United Mexican States	Both	Age-standardized	Periodontal diseases	Rate	1992	1248.72	1444.09	993.93
1030	Incidence	United Mexican States	Male	Age-standardized	Periodontal diseases	Rate	1993	1267.09	1460.12	1008.19
1031	Incidence	United Mexican States	Female	Age-standardized	Periodontal diseases	Rate	1993	1231.34	1424.88	981.07
1032	Incidence	United Mexican States	Both	Age-standardized	Periodontal diseases	Rate	1993	1248.60	1442.22	994.15
1033	Incidence	United Mexican States	Male	Age-standardized	Periodontal diseases	Rate	1994	1267.23	1460.71	1009.48
1034	Incidence	United Mexican States	Female	Age-standardized	Periodontal diseases	Rate	1994	1231.16	1419.93	980.47

1035	Incidence	United Mexican States	Both	Age-standardized	Periodontal diseases	Rate	1994	1248.58	1440.51	994.46
1036	Incidence	United Mexican States	Male	Age-standardized	Periodontal diseases	Rate	1995	1267.31	1461.06	1010.77
1037	Incidence	United Mexican States	Female	Age-standardized	Periodontal diseases	Rate	1995	1231.21	1415.85	980.04
1038	Incidence	United Mexican States	Both	Age-standardized	Periodontal diseases	Rate	1995	1248.65	1439.01	994.84
1039	Incidence	United Mexican States	Male	Age-standardized	Periodontal diseases	Rate	1996	1267.20	1459.58	1011.33
1040	Incidence	United Mexican States	Female	Age-standardized	Periodontal diseases	Rate	1996	1232.01	1418.06	981.18
1041	Incidence	United Mexican States	Both	Age-standardized	Periodontal diseases	Rate	1996	1248.99	1439.61	995.70
1042	Incidence	United Mexican States	Male	Age-standardized	Periodontal diseases	Rate	1997	1266.83	1457.88	1011.61
1043	Incidence	United Mexican States	Female	Age-standardized	Periodontal diseases	Rate	1997	1233.66	1420.70	982.90
1044	Incidence	United Mexican States	Both	Age-standardized	Periodontal diseases	Rate	1997	1249.64	1440.54	996.72
1045	Incidence	United Mexican States	Male	Age-standardized	Periodontal diseases	Rate	1998	1266.36	1457.53	1011.76
1046	Incidence	United Mexican States	Female	Age-standardized	Periodontal diseases	Rate	1998	1235.58	1423.00	984.79
1047	Incidence	United Mexican States	Both	Age-standardized	Periodontal diseases	Rate	1998	1250.39	1441.56	997.76
1048	Incidence	United Mexican States	Male	Age-standardized	Periodontal diseases	Rate	1999	1265.94	1457.33	1011.89
1049	Incidence	United Mexican	Female	Age-standardized	Periodontal diseases	Rate	1999	1237.16	1425.91	986.46

		States								
1050	Incidence	United Mexican States	Both	Age-standardized	Periodontal diseases	Rate	1999	1250.99	1442.69	998.68
1051	Incidence	United Mexican States	Male	Age-standardized	Periodontal diseases	Rate	2000	1265.73	1458.01	1012.16
1052	Incidence	United Mexican States	Female	Age-standardized	Periodontal diseases	Rate	2000	1237.85	1425.53	987.55
1053	Incidence	United Mexican States	Both	Age-standardized	Periodontal diseases	Rate	2000	1251.23	1443.36	999.37
1054	Incidence	United Mexican States	Male	Age-standardized	Periodontal diseases	Rate	2001	1265.49	1456.66	1010.65
1055	Incidence	United Mexican States	Female	Age-standardized	Periodontal diseases	Rate	2001	1237.53	1425.16	987.69
1056	Incidence	United Mexican States	Both	Age-standardized	Periodontal diseases	Rate	2001	1250.94	1440.82	998.70
1057	Incidence	United Mexican States	Male	Age-standardized	Periodontal diseases	Rate	2002	1265.02	1455.74	1011.13
1058	Incidence	United Mexican States	Female	Age-standardized	Periodontal diseases	Rate	2002	1236.68	1423.15	987.56
1059	Incidence	United Mexican States	Both	Age-standardized	Periodontal diseases	Rate	2002	1250.28	1438.22	997.66
1060	Incidence	United Mexican States	Male	Age-standardized	Periodontal diseases	Rate	2003	1264.48	1454.68	1012.32
1061	Incidence	United Mexican States	Female	Age-standardized	Periodontal diseases	Rate	2003	1235.62	1420.08	987.87
1062	Incidence	United Mexican States	Both	Age-standardized	Periodontal diseases	Rate	2003	1249.48	1435.59	999.10
1063	Incidence	United Mexican States	Male	Age-standardized	Periodontal diseases	Rate	2004	1264.04	1452.91	1013.79
1064	Incidence	United	Female	Age-standardized	Periodontal	Rate	2004	1234.67	1417.03	989.42

		Mexican States			diseases					
1065	Incidence	United Mexican States	Both	Age-standardized	Periodontal diseases	Rate	2004	1248.80	1432.90	1001.12
1066	Incidence	United Mexican States	Male	Age-standardized	Periodontal diseases	Rate	2005	1263.87	1451.14	1015.83
1067	Incidence	United Mexican States	Female	Age-standardized	Periodontal diseases	Rate	2005	1234.14	1415.35	992.28
1068	Incidence	United Mexican States	Both	Age-standardized	Periodontal diseases	Rate	2005	1248.47	1430.51	1003.60
1069	Incidence	United Mexican States	Male	Age-standardized	Periodontal diseases	Rate	2006	1264.05	1449.62	1017.14
1070	Incidence	United Mexican States	Female	Age-standardized	Periodontal diseases	Rate	2006	1234.05	1415.34	993.44
1071	Incidence	United Mexican States	Both	Age-standardized	Periodontal diseases	Rate	2006	1248.54	1431.19	1004.87
1072	Incidence	United Mexican States	Male	Age-standardized	Periodontal diseases	Rate	2007	1264.48	1448.71	1018.73
1073	Incidence	United Mexican States	Female	Age-standardized	Periodontal diseases	Rate	2007	1234.13	1416.29	994.74
1074	Incidence	United Mexican States	Both	Age-standardized	Periodontal diseases	Rate	2007	1248.82	1432.18	1006.34
1075	Incidence	United Mexican States	Male	Age-standardized	Periodontal diseases	Rate	2008	1265.04	1448.67	1020.42
1076	Incidence	United Mexican States	Female	Age-standardized	Periodontal diseases	Rate	2008	1234.31	1417.60	996.04
1077	Incidence	United Mexican States	Both	Age-standardized	Periodontal diseases	Rate	2008	1249.21	1433.76	1007.86
1078	Incidence	United Mexican States	Male	Age-standardized	Periodontal diseases	Rate	2009	1265.60	1448.60	1022.04

1079	Incidence	United Mexican States	Female	Age-standardized	Periodontal diseases	Rate	2009	1234.48	1418.37	997.23
1080	Incidence	United Mexican States	Both	Age-standardized	Periodontal diseases	Rate	2009	1249.61	1433.97	1009.29
1081	Incidence	United Mexican States	Male	Age-standardized	Periodontal diseases	Rate	2010	1266.05	1448.25	1023.50
1082	Incidence	United Mexican States	Female	Age-standardized	Periodontal diseases	Rate	2010	1234.57	1418.89	998.32
1083	Incidence	United Mexican States	Both	Age-standardized	Periodontal diseases	Rate	2010	1249.91	1434.18	1010.59
1084	Incidence	United Mexican States	Male	Age-standardized	Periodontal diseases	Rate	2011	1266.66	1449.72	1024.87
1085	Incidence	United Mexican States	Female	Age-standardized	Periodontal diseases	Rate	2011	1234.50	1417.70	996.99
1086	Incidence	United Mexican States	Both	Age-standardized	Periodontal diseases	Rate	2011	1250.20	1434.40	1010.59
1087	Incidence	United Mexican States	Male	Age-standardized	Periodontal diseases	Rate	2012	1267.58	1451.73	1026.49
1088	Incidence	United Mexican States	Female	Age-standardized	Periodontal diseases	Rate	2012	1234.32	1416.41	995.49
1089	Incidence	United Mexican States	Both	Age-standardized	Periodontal diseases	Rate	2012	1250.59	1436.26	1010.61
1090	Incidence	United Mexican States	Male	Age-standardized	Periodontal diseases	Rate	2013	1268.58	1454.28	1028.11
1091	Incidence	United Mexican States	Female	Age-standardized	Periodontal diseases	Rate	2013	1234.19	1416.79	993.81
1092	Incidence	United Mexican States	Both	Age-standardized	Periodontal diseases	Rate	2013	1251.04	1437.40	1010.54
1093	Incidence	United Mexican	Male	Age-standardized	Periodontal diseases	Rate	2014	1269.38	1457.53	1029.46

		States								
1094	Incidence	United Mexican States	Female	Age-standardized	Periodontal diseases	Rate	2014	1234.24	1417.55	992.44
1095	Incidence	United Mexican States	Both	Age-standardized	Periodontal diseases	Rate	2014	1251.48	1438.18	1010.31
1096	Incidence	United Mexican States	Male	Age-standardized	Periodontal diseases	Rate	2015	1269.71	1457.90	1030.37
1097	Incidence	United Mexican States	Female	Age-standardized	Periodontal diseases	Rate	2015	1234.62	1418.45	993.44
1098	Incidence	United Mexican States	Both	Age-standardized	Periodontal diseases	Rate	2015	1251.86	1438.72	1009.94
1099	Incidence	United Mexican States	Male	Age-standardized	Periodontal diseases	Rate	2016	1269.27	1457.79	1028.00
1100	Incidence	United Mexican States	Female	Age-standardized	Periodontal diseases	Rate	2016	1236.58	1417.47	996.85
1101	Incidence	United Mexican States	Both	Age-standardized	Periodontal diseases	Rate	2016	1252.66	1438.33	1010.86
1102	Incidence	United Mexican States	Male	Age-standardized	Periodontal diseases	Rate	2017	1268.26	1455.76	1024.77
1103	Incidence	United Mexican States	Female	Age-standardized	Periodontal diseases	Rate	2017	1240.09	1419.78	1001.53
1104	Incidence	United Mexican States	Both	Age-standardized	Periodontal diseases	Rate	2017	1253.96	1438.68	1011.77
1105	Incidence	United Mexican States	Male	Age-standardized	Periodontal diseases	Rate	2018	1267.18	1454.91	1021.26
1106	Incidence	United Mexican States	Female	Age-standardized	Periodontal diseases	Rate	2018	1243.38	1422.87	1005.98
1107	Incidence	United Mexican States	Both	Age-standardized	Periodontal diseases	Rate	2018	1255.11	1439.45	1012.41
1108	Incidence	United	Male	Age-standardized	Periodontal	Rate	2019	1266.54	1452.26	1018.13

		Mexican States			diseases					
1109	Incidence	United Mexican States	Female	Age-standardized	Periodontal diseases	Rate	2019	1244.66	1423.12	1009.25
1110	Incidence	United Mexican States	Both	Age-standardized	Periodontal diseases	Rate	2019	1255.45	1438.70	1012.58
1111	Incidence	United Mexican States	Male	Age-standardized	Periodontal diseases	Rate	2020	1263.31	1423.05	1064.88
1112	Incidence	United Mexican States	Female	Age-standardized	Periodontal diseases	Rate	2020	1223.04	1370.71	1079.00
1113	Incidence	United Mexican States	Both	Age-standardized	Periodontal diseases	Rate	2020	1242.84	1399.27	1076.74
1114	Incidence	United Mexican States	Male	Age-standardized	Periodontal diseases	Rate	2021	1237.65	1389.58	1098.01
1115	Incidence	United Mexican States	Female	Age-standardized	Periodontal diseases	Rate	2021	1220.68	1371.04	1084.21
1116	Incidence	United Mexican States	Both	Age-standardized	Periodontal diseases	Rate	2021	1229.07	1380.14	1091.38
1117	Incidence	United Mexican States	Male	Age-standardized	Periodontal diseases	Rate	2022	1241.44	1391.69	1104.76
1118	Incidence	United Mexican States	Female	Age-standardized	Periodontal diseases	Rate	2022	1222.77	1374.28	1088.20
1119	Incidence	United Mexican States	Both	Age-standardized	Periodontal diseases	Rate	2022	1232.02	1383.33	1097.99
1120	Incidence	United Mexican States	Male	Age-standardized	Periodontal diseases	Rate	2023	1225.00	1373.40	1090.62
1121	Incidence	United Mexican States	Female	Age-standardized	Periodontal diseases	Rate	2023	1206.56	1355.80	1073.53
1122	Incidence	United Mexican States	Both	Age-standardized	Periodontal diseases	Rate	2023	1215.71	1365.19	1083.29

1123	Incidence	Kingdom of Saudi Arabia	Male	Age-standardized	Periodontal diseases	Rate	1990	769.17	913.04	613.44
1124	Incidence	Kingdom of Saudi Arabia	Female	Age-standardized	Periodontal diseases	Rate	1990	693.65	841.15	551.70
1125	Incidence	Kingdom of Saudi Arabia	Both	Age-standardized	Periodontal diseases	Rate	1990	739.81	880.24	589.10
1126	Incidence	Kingdom of Saudi Arabia	Male	Age-standardized	Periodontal diseases	Rate	1991	810.48	951.25	644.25
1127	Incidence	Kingdom of Saudi Arabia	Female	Age-standardized	Periodontal diseases	Rate	1991	716.76	845.54	596.49
1128	Incidence	Kingdom of Saudi Arabia	Both	Age-standardized	Periodontal diseases	Rate	1991	773.83	910.84	625.70
1129	Incidence	Kingdom of Saudi Arabia	Male	Age-standardized	Periodontal diseases	Rate	1992	845.34	989.03	666.52
1130	Incidence	Kingdom of Saudi Arabia	Female	Age-standardized	Periodontal diseases	Rate	1992	737.84	854.66	622.83
1131	Incidence	Kingdom of Saudi Arabia	Both	Age-standardized	Periodontal diseases	Rate	1992	803.05	930.68	653.14
1132	Incidence	Kingdom of Saudi Arabia	Male	Age-standardized	Periodontal diseases	Rate	1993	872.25	1023.27	678.22
1133	Incidence	Kingdom of Saudi Arabia	Female	Age-standardized	Periodontal diseases	Rate	1993	755.10	887.75	641.96
1134	Incidence	Kingdom of Saudi Arabia	Both	Age-standardized	Periodontal diseases	Rate	1993	825.91	949.66	673.22
1135	Incidence	Kingdom of Saudi Arabia	Male	Age-standardized	Periodontal diseases	Rate	1994	889.70	1063.68	681.37
1136	Incidence	Kingdom of Saudi Arabia	Female	Age-standardized	Periodontal diseases	Rate	1994	766.73	924.80	637.53
1137	Incidence	Kingdom of Saudi Arabia	Both	Age-standardized	Periodontal diseases	Rate	1994	840.88	971.71	685.80
1138	Incidence	Kingdom of Saudi Arabia	Male	Age-standardized	Periodontal diseases	Rate	1995	896.18	1093.82	673.40
1139	Incidence	Kingdom of Saudi Arabia	Female	Age-standardized	Periodontal diseases	Rate	1995	770.95	961.83	601.80
1140	Incidence	Kingdom of Saudi Arabia	Both	Age-standardized	Periodontal diseases	Rate	1995	846.44	983.63	692.92
1141	Incidence	Kingdom of Saudi Arabia	Male	Age-standardized	Periodontal diseases	Rate	1996	889.95	1044.96	713.79
1142	Incidence	Kingdom of Saudi Arabia	Female	Age-standardized	Periodontal diseases	Rate	1996	770.58	955.24	596.96
1143	Incidence	Kingdom of Saudi Arabia	Both	Age-standardized	Periodontal diseases	Rate	1996	842.43	977.69	696.73
1144	Incidence	Kingdom of Saudi Arabia	Male	Age-standardized	Periodontal diseases	Rate	1997	874.30	1007.65	726.51

1145	Incidence	Kingdom of Saudi Arabia	Female	Age-standardized	Periodontal diseases	Rate	1997	769.80	953.35	593.47
1146	Incidence	Kingdom of Saudi Arabia	Both	Age-standardized	Periodontal diseases	Rate	1997	832.59	975.19	701.94
1147	Incidence	Kingdom of Saudi Arabia	Male	Age-standardized	Periodontal diseases	Rate	1998	855.28	1001.44	715.69
1148	Incidence	Kingdom of Saudi Arabia	Female	Age-standardized	Periodontal diseases	Rate	1998	768.88	952.51	591.18
1149	Incidence	Kingdom of Saudi Arabia	Both	Age-standardized	Periodontal diseases	Rate	1998	820.74	975.74	688.87
1150	Incidence	Kingdom of Saudi Arabia	Male	Age-standardized	Periodontal diseases	Rate	1999	838.95	1007.55	697.94
1151	Incidence	Kingdom of Saudi Arabia	Female	Age-standardized	Periodontal diseases	Rate	1999	768.05	952.13	591.36
1152	Incidence	Kingdom of Saudi Arabia	Both	Age-standardized	Periodontal diseases	Rate	1999	810.57	978.87	657.51
1153	Incidence	Kingdom of Saudi Arabia	Male	Age-standardized	Periodontal diseases	Rate	2000	831.39	1022.99	648.63
1154	Incidence	Kingdom of Saudi Arabia	Female	Age-standardized	Periodontal diseases	Rate	2000	767.59	952.13	592.01
1155	Incidence	Kingdom of Saudi Arabia	Both	Age-standardized	Periodontal diseases	Rate	2000	805.75	987.20	625.06
1156	Incidence	Kingdom of Saudi Arabia	Male	Age-standardized	Periodontal diseases	Rate	2001	830.46	1021.24	647.97
1157	Incidence	Kingdom of Saudi Arabia	Female	Age-standardized	Periodontal diseases	Rate	2001	767.46	951.85	593.50
1158	Incidence	Kingdom of Saudi Arabia	Both	Age-standardized	Periodontal diseases	Rate	2001	805.05	984.76	624.60
1159	Incidence	Kingdom of Saudi Arabia	Male	Age-standardized	Periodontal diseases	Rate	2002	829.99	1018.12	648.08
1160	Incidence	Kingdom of Saudi Arabia	Female	Age-standardized	Periodontal diseases	Rate	2002	767.51	952.52	594.23
1161	Incidence	Kingdom of Saudi Arabia	Both	Age-standardized	Periodontal diseases	Rate	2002	804.72	982.90	625.60
1162	Incidence	Kingdom of Saudi Arabia	Male	Age-standardized	Periodontal diseases	Rate	2003	829.92	1016.80	649.17
1163	Incidence	Kingdom of Saudi Arabia	Female	Age-standardized	Periodontal diseases	Rate	2003	767.85	953.77	595.24
1164	Incidence	Kingdom of Saudi Arabia	Both	Age-standardized	Periodontal diseases	Rate	2003	804.75	982.46	627.98
1165	Incidence	Kingdom of Saudi Arabia	Male	Age-standardized	Periodontal diseases	Rate	2004	830.20	1020.67	649.19
1166	Incidence	Kingdom of Saudi Arabia	Female	Age-standardized	Periodontal diseases	Rate	2004	768.55	946.98	596.62

1167	Incidence	Kingdom of Saudi Arabia	Both	Age-standardized	Periodontal diseases	Rate	2004	805.14	984.83	629.80
1168	Incidence	Kingdom of Saudi Arabia	Male	Age-standardized	Periodontal diseases	Rate	2005	830.79	1020.81	646.68
1169	Incidence	Kingdom of Saudi Arabia	Female	Age-standardized	Periodontal diseases	Rate	2005	769.69	940.51	598.16
1170	Incidence	Kingdom of Saudi Arabia	Both	Age-standardized	Periodontal diseases	Rate	2005	805.89	988.16	632.78
1171	Incidence	Kingdom of Saudi Arabia	Male	Age-standardized	Periodontal diseases	Rate	2006	832.33	1022.24	653.20
1172	Incidence	Kingdom of Saudi Arabia	Female	Age-standardized	Periodontal diseases	Rate	2006	772.29	945.70	598.62
1173	Incidence	Kingdom of Saudi Arabia	Both	Age-standardized	Periodontal diseases	Rate	2006	807.80	991.23	630.90
1174	Incidence	Kingdom of Saudi Arabia	Male	Age-standardized	Periodontal diseases	Rate	2007	835.11	1021.60	659.46
1175	Incidence	Kingdom of Saudi Arabia	Female	Age-standardized	Periodontal diseases	Rate	2007	776.64	954.07	600.86
1176	Incidence	Kingdom of Saudi Arabia	Both	Age-standardized	Periodontal diseases	Rate	2007	811.18	997.31	633.26
1177	Incidence	Kingdom of Saudi Arabia	Male	Age-standardized	Periodontal diseases	Rate	2008	838.55	1021.31	662.31
1178	Incidence	Kingdom of Saudi Arabia	Female	Age-standardized	Periodontal diseases	Rate	2008	781.74	964.70	603.90
1179	Incidence	Kingdom of Saudi Arabia	Both	Age-standardized	Periodontal diseases	Rate	2008	815.29	1000.93	638.00
1180	Incidence	Kingdom of Saudi Arabia	Male	Age-standardized	Periodontal diseases	Rate	2009	842.08	1025.58	662.44
1181	Incidence	Kingdom of Saudi Arabia	Female	Age-standardized	Periodontal diseases	Rate	2009	786.57	975.85	603.99
1182	Incidence	Kingdom of Saudi Arabia	Both	Age-standardized	Periodontal diseases	Rate	2009	819.39	1005.08	640.50
1183	Incidence	Kingdom of Saudi Arabia	Male	Age-standardized	Periodontal diseases	Rate	2010	845.11	1036.07	664.07
1184	Incidence	Kingdom of Saudi Arabia	Female	Age-standardized	Periodontal diseases	Rate	2010	790.13	984.54	605.67
1185	Incidence	Kingdom of Saudi Arabia	Both	Age-standardized	Periodontal diseases	Rate	2010	822.73	1008.44	641.62
1186	Incidence	Kingdom of Saudi Arabia	Male	Age-standardized	Periodontal diseases	Rate	2011	848.88	1038.72	666.47
1187	Incidence	Kingdom of Saudi Arabia	Female	Age-standardized	Periodontal diseases	Rate	2011	793.21	986.38	607.47
1188	Incidence	Kingdom of Saudi Arabia	Both	Age-standardized	Periodontal diseases	Rate	2011	826.40	1010.52	644.72

1189	Incidence	Kingdom of Saudi Arabia	Male	Age-standardized	Periodontal diseases	Rate	2012	854.22	1043.66	672.05
1190	Incidence	Kingdom of Saudi Arabia	Female	Age-standardized	Periodontal diseases	Rate	2012	796.85	990.10	615.01
1191	Incidence	Kingdom of Saudi Arabia	Both	Age-standardized	Periodontal diseases	Rate	2012	831.31	1013.61	648.63
1192	Incidence	Kingdom of Saudi Arabia	Male	Age-standardized	Periodontal diseases	Rate	2013	859.93	1046.34	674.80
1193	Incidence	Kingdom of Saudi Arabia	Female	Age-standardized	Periodontal diseases	Rate	2013	800.40	990.81	620.23
1194	Incidence	Kingdom of Saudi Arabia	Both	Age-standardized	Periodontal diseases	Rate	2013	836.47	1020.83	652.67
1195	Incidence	Kingdom of Saudi Arabia	Male	Age-standardized	Periodontal diseases	Rate	2014	864.82	1055.82	683.30
1196	Incidence	Kingdom of Saudi Arabia	Female	Age-standardized	Periodontal diseases	Rate	2014	803.21	999.42	624.61
1197	Incidence	Kingdom of Saudi Arabia	Both	Age-standardized	Periodontal diseases	Rate	2014	840.89	1027.52	655.41
1198	Incidence	Kingdom of Saudi Arabia	Male	Age-standardized	Periodontal diseases	Rate	2015	867.68	1068.91	688.91
1199	Incidence	Kingdom of Saudi Arabia	Female	Age-standardized	Periodontal diseases	Rate	2015	804.63	1007.20	625.78
1200	Incidence	Kingdom of Saudi Arabia	Both	Age-standardized	Periodontal diseases	Rate	2015	843.53	1033.10	657.15
1201	Incidence	Kingdom of Saudi Arabia	Male	Age-standardized	Periodontal diseases	Rate	2016	869.06	1065.27	684.78
1202	Incidence	Kingdom of Saudi Arabia	Female	Age-standardized	Periodontal diseases	Rate	2016	804.48	1001.05	625.82
1203	Incidence	Kingdom of Saudi Arabia	Both	Age-standardized	Periodontal diseases	Rate	2016	844.72	1030.92	658.72
1204	Incidence	Kingdom of Saudi Arabia	Male	Age-standardized	Periodontal diseases	Rate	2017	870.21	1062.91	680.93
1205	Incidence	Kingdom of Saudi Arabia	Female	Age-standardized	Periodontal diseases	Rate	2017	803.47	997.14	624.70
1206	Incidence	Kingdom of Saudi Arabia	Both	Age-standardized	Periodontal diseases	Rate	2017	845.48	1032.09	659.06
1207	Incidence	Kingdom of Saudi Arabia	Male	Age-standardized	Periodontal diseases	Rate	2018	870.96	1062.64	679.44
1208	Incidence	Kingdom of Saudi Arabia	Female	Age-standardized	Periodontal diseases	Rate	2018	802.27	998.36	621.89
1209	Incidence	Kingdom of Saudi Arabia	Both	Age-standardized	Periodontal diseases	Rate	2018	845.96	1033.12	659.61
1210	Incidence	Kingdom of Saudi Arabia	Male	Age-standardized	Periodontal diseases	Rate	2019	871.16	1057.06	682.42

1211	Incidence	Kingdom of Saudi Arabia	Female	Age-standardized	Periodontal diseases	Rate	2019	801.52	1000.38	619.35
1212	Incidence	Kingdom of Saudi Arabia	Both	Age-standardized	Periodontal diseases	Rate	2019	846.26	1033.88	657.28
1213	Incidence	Kingdom of Saudi Arabia	Male	Age-standardized	Periodontal diseases	Rate	2020	865.44	1069.35	680.04
1214	Incidence	Kingdom of Saudi Arabia	Female	Age-standardized	Periodontal diseases	Rate	2020	800.82	988.70	621.74
1215	Incidence	Kingdom of Saudi Arabia	Both	Age-standardized	Periodontal diseases	Rate	2020	842.55	1037.05	658.06
1216	Incidence	Kingdom of Saudi Arabia	Male	Age-standardized	Periodontal diseases	Rate	2021	859.22	1055.88	663.66
1217	Incidence	Kingdom of Saudi Arabia	Female	Age-standardized	Periodontal diseases	Rate	2021	800.95	991.66	623.04
1218	Incidence	Kingdom of Saudi Arabia	Both	Age-standardized	Periodontal diseases	Rate	2021	838.59	1025.42	647.95
1219	Incidence	Kingdom of Saudi Arabia	Male	Age-standardized	Periodontal diseases	Rate	2022	867.00	1046.44	682.66
1220	Incidence	Kingdom of Saudi Arabia	Female	Age-standardized	Periodontal diseases	Rate	2022	803.78	1003.81	631.17
1221	Incidence	Kingdom of Saudi Arabia	Both	Age-standardized	Periodontal diseases	Rate	2022	845.39	1030.07	662.95
1222	Incidence	Kingdom of Saudi Arabia	Male	Age-standardized	Periodontal diseases	Rate	2023	854.54	1031.87	671.97
1223	Incidence	Kingdom of Saudi Arabia	Female	Age-standardized	Periodontal diseases	Rate	2023	792.19	989.70	621.90
1224	Incidence	Kingdom of Saudi Arabia	Both	Age-standardized	Periodontal diseases	Rate	2023	833.49	1016.38	653.27
1225	Incidence	Republic of Turkey	Male	Age-standardized	Periodontal diseases	Rate	1990	704.03	897.17	506.00
1226	Incidence	Republic of Turkey	Female	Age-standardized	Periodontal diseases	Rate	1990	698.27	876.65	501.94
1227	Incidence	Republic of Turkey	Both	Age-standardized	Periodontal diseases	Rate	1990	701.20	884.99	502.47
1228	Incidence	Republic of Turkey	Male	Age-standardized	Periodontal diseases	Rate	1991	704.65	895.11	508.79
1229	Incidence	Republic of Turkey	Female	Age-standardized	Periodontal diseases	Rate	1991	816.52	979.74	637.59
1230	Incidence	Republic of Turkey	Both	Age-standardized	Periodontal diseases	Rate	1991	760.22	934.89	573.26
1231	Incidence	Republic of Turkey	Male	Age-standardized	Periodontal diseases	Rate	1992	706.83	894.13	511.47
1232	Incidence	Republic of Turkey	Female	Age-standardized	Periodontal diseases	Rate	1992	924.80	1081.20	767.85

1233	Incidence	Republic of Turkey	Both	Age-standardized	Periodontal diseases	Rate	1992	815.07	976.95	649.06
1234	Incidence	Republic of Turkey	Male	Age-standardized	Periodontal diseases	Rate	1993	710.39	896.47	517.63
1235	Incidence	Republic of Turkey	Female	Age-standardized	Periodontal diseases	Rate	1993	1014.53	1173.48	865.76
1236	Incidence	Republic of Turkey	Both	Age-standardized	Periodontal diseases	Rate	1993	861.41	1013.17	707.43
1237	Incidence	Republic of Turkey	Male	Age-standardized	Periodontal diseases	Rate	1994	715.13	900.44	522.50
1238	Incidence	Republic of Turkey	Female	Age-standardized	Periodontal diseases	Rate	1994	1077.11	1258.41	933.69
1239	Incidence	Republic of Turkey	Both	Age-standardized	Periodontal diseases	Rate	1994	894.90	1047.24	743.52
1240	Incidence	Republic of Turkey	Male	Age-standardized	Periodontal diseases	Rate	1995	720.89	902.96	525.75
1241	Incidence	Republic of Turkey	Female	Age-standardized	Periodontal diseases	Rate	1995	1103.98	1299.59	935.26
1242	Incidence	Republic of Turkey	Both	Age-standardized	Periodontal diseases	Rate	1995	911.21	1069.39	770.17
1243	Incidence	Republic of Turkey	Male	Age-standardized	Periodontal diseases	Rate	1996	767.64	929.67	601.69
1244	Incidence	Republic of Turkey	Female	Age-standardized	Periodontal diseases	Rate	1996	1109.19	1304.78	941.14
1245	Incidence	Republic of Turkey	Both	Age-standardized	Periodontal diseases	Rate	1996	937.37	1087.02	806.11
1246	Incidence	Republic of Turkey	Male	Age-standardized	Periodontal diseases	Rate	1997	870.63	1022.66	727.48
1247	Incidence	Republic of Turkey	Female	Age-standardized	Periodontal diseases	Rate	1997	1112.78	1314.78	945.54
1248	Incidence	Republic of Turkey	Both	Age-standardized	Periodontal diseases	Rate	1997	991.02	1137.65	863.24
1249	Incidence	Republic of Turkey	Male	Age-standardized	Periodontal diseases	Rate	1998	992.63	1144.46	863.15
1250	Incidence	Republic of Turkey	Female	Age-standardized	Periodontal diseases	Rate	1998	1115.09	1326.86	946.25
1251	Incidence	Republic of Turkey	Both	Age-standardized	Periodontal diseases	Rate	1998	1053.60	1223.20	917.98
1252	Incidence	Republic of Turkey	Male	Age-standardized	Periodontal diseases	Rate	1999	1096.40	1276.86	959.15
1253	Incidence	Republic of Turkey	Female	Age-standardized	Periodontal diseases	Rate	1999	1116.49	1337.78	946.05
1254	Incidence	Republic of Turkey	Both	Age-standardized	Periodontal diseases	Rate	1999	1106.54	1306.64	956.99

1255	Incidence	Republic of Turkey	Male	Age-standardized	Periodontal diseases	Rate	2000	1144.70	1375.38	973.48
1256	Incidence	Republic of Turkey	Female	Age-standardized	Periodontal diseases	Rate	2000	1117.31	1347.65	944.47
1257	Incidence	Republic of Turkey	Both	Age-standardized	Periodontal diseases	Rate	2000	1131.30	1363.53	963.52
1258	Incidence	Republic of Turkey	Male	Age-standardized	Periodontal diseases	Rate	2001	1151.95	1373.59	982.43
1259	Incidence	Republic of Turkey	Female	Age-standardized	Periodontal diseases	Rate	2001	1119.26	1353.71	944.80
1260	Incidence	Republic of Turkey	Both	Age-standardized	Periodontal diseases	Rate	2001	1135.98	1365.23	969.67
1261	Incidence	Republic of Turkey	Male	Age-standardized	Periodontal diseases	Rate	2002	1157.59	1370.46	990.47
1262	Incidence	Republic of Turkey	Female	Age-standardized	Periodontal diseases	Rate	2002	1123.09	1362.03	952.49
1263	Incidence	Republic of Turkey	Both	Age-standardized	Periodontal diseases	Rate	2002	1140.76	1367.83	977.45
1264	Incidence	Republic of Turkey	Male	Age-standardized	Periodontal diseases	Rate	2003	1161.88	1368.41	999.47
1265	Incidence	Republic of Turkey	Female	Age-standardized	Periodontal diseases	Rate	2003	1127.72	1371.84	962.02
1266	Incidence	Republic of Turkey	Both	Age-standardized	Periodontal diseases	Rate	2003	1145.25	1370.47	984.43
1267	Incidence	Republic of Turkey	Male	Age-standardized	Periodontal diseases	Rate	2004	1165.08	1370.82	1005.78
1268	Incidence	Republic of Turkey	Female	Age-standardized	Periodontal diseases	Rate	2004	1132.09	1381.07	971.73
1269	Incidence	Republic of Turkey	Both	Age-standardized	Periodontal diseases	Rate	2004	1149.03	1375.01	988.70
1270	Incidence	Republic of Turkey	Male	Age-standardized	Periodontal diseases	Rate	2005	1167.46	1372.12	1008.95
1271	Incidence	Republic of Turkey	Female	Age-standardized	Periodontal diseases	Rate	2005	1135.13	1388.17	975.63
1272	Incidence	Republic of Turkey	Both	Age-standardized	Periodontal diseases	Rate	2005	1151.74	1379.13	991.56
1273	Incidence	Republic of Turkey	Male	Age-standardized	Periodontal diseases	Rate	2006	1170.33	1385.53	1010.33
1274	Incidence	Republic of Turkey	Female	Age-standardized	Periodontal diseases	Rate	2006	1138.62	1386.06	977.51
1275	Incidence	Republic of Turkey	Both	Age-standardized	Periodontal diseases	Rate	2006	1154.91	1383.54	992.98
1276	Incidence	Republic of Turkey	Male	Age-standardized	Periodontal diseases	Rate	2007	1174.41	1400.65	1012.49

1277	Incidence	Republic of Turkey	Female	Age-standardized	Periodontal diseases	Rate	2007	1144.20	1386.54	986.85
1278	Incidence	Republic of Turkey	Both	Age-standardized	Periodontal diseases	Rate	2007	1159.71	1392.61	998.14
1279	Incidence	Republic of Turkey	Male	Age-standardized	Periodontal diseases	Rate	2008	1179.12	1411.81	1014.79
1280	Incidence	Republic of Turkey	Female	Age-standardized	Periodontal diseases	Rate	2008	1150.53	1390.80	997.32
1281	Incidence	Republic of Turkey	Both	Age-standardized	Periodontal diseases	Rate	2008	1165.21	1402.71	1003.82
1282	Incidence	Republic of Turkey	Male	Age-standardized	Periodontal diseases	Rate	2009	1183.86	1416.73	1013.75
1283	Incidence	Republic of Turkey	Female	Age-standardized	Periodontal diseases	Rate	2009	1156.29	1393.75	1002.29
1284	Incidence	Republic of Turkey	Both	Age-standardized	Periodontal diseases	Rate	2009	1170.43	1411.22	1008.11
1285	Incidence	Republic of Turkey	Male	Age-standardized	Periodontal diseases	Rate	2010	1188.03	1419.01	1014.69
1286	Incidence	Republic of Turkey	Female	Age-standardized	Periodontal diseases	Rate	2010	1160.15	1394.45	1003.17
1287	Incidence	Republic of Turkey	Both	Age-standardized	Periodontal diseases	Rate	2010	1174.42	1415.71	1008.87
1288	Incidence	Republic of Turkey	Male	Age-standardized	Periodontal diseases	Rate	2011	1191.89	1412.91	1027.28
1289	Incidence	Republic of Turkey	Female	Age-standardized	Periodontal diseases	Rate	2011	1161.44	1387.38	1004.32
1290	Incidence	Republic of Turkey	Both	Age-standardized	Periodontal diseases	Rate	2011	1176.99	1410.64	1016.40
1291	Incidence	Republic of Turkey	Male	Age-standardized	Periodontal diseases	Rate	2012	1195.84	1404.69	1044.14
1292	Incidence	Republic of Turkey	Female	Age-standardized	Periodontal diseases	Rate	2012	1160.98	1375.18	1010.83
1293	Incidence	Republic of Turkey	Both	Age-standardized	Periodontal diseases	Rate	2012	1178.73	1400.54	1027.64
1294	Incidence	Republic of Turkey	Male	Age-standardized	Periodontal diseases	Rate	2013	1199.47	1395.42	1052.94
1295	Incidence	Republic of Turkey	Female	Age-standardized	Periodontal diseases	Rate	2013	1159.74	1363.14	1020.89
1296	Incidence	Republic of Turkey	Both	Age-standardized	Periodontal diseases	Rate	2013	1179.91	1388.65	1034.54
1297	Incidence	Republic of Turkey	Male	Age-standardized	Periodontal diseases	Rate	2014	1202.34	1386.47	1058.18
1298	Incidence	Republic of Turkey	Female	Age-standardized	Periodontal diseases	Rate	2014	1158.65	1354.31	1018.92

1299	Incidence	Republic of Turkey	Both	Age-standardized	Periodontal diseases	Rate	2014	1180.80	1375.92	1044.00
1300	Incidence	Republic of Turkey	Male	Age-standardized	Periodontal diseases	Rate	2015	1204.05	1380.72	1062.46
1301	Incidence	Republic of Turkey	Female	Age-standardized	Periodontal diseases	Rate	2015	1158.65	1350.49	1017.12
1302	Incidence	Republic of Turkey	Both	Age-standardized	Periodontal diseases	Rate	2015	1181.68	1367.57	1041.88
1303	Incidence	Republic of Turkey	Male	Age-standardized	Periodontal diseases	Rate	2016	1205.35	1378.35	1065.61
1304	Incidence	Republic of Turkey	Female	Age-standardized	Periodontal diseases	Rate	2016	1161.64	1355.50	1020.62
1305	Incidence	Republic of Turkey	Both	Age-standardized	Periodontal diseases	Rate	2016	1183.87	1367.19	1044.66
1306	Incidence	Republic of Turkey	Male	Age-standardized	Periodontal diseases	Rate	2017	1206.82	1382.80	1055.96
1307	Incidence	Republic of Turkey	Female	Age-standardized	Periodontal diseases	Rate	2017	1167.19	1369.06	1022.52
1308	Incidence	Republic of Turkey	Both	Age-standardized	Periodontal diseases	Rate	2017	1187.38	1371.19	1042.60
1309	Incidence	Republic of Turkey	Male	Age-standardized	Periodontal diseases	Rate	2018	1207.77	1390.29	1051.08
1310	Incidence	Republic of Turkey	Female	Age-standardized	Periodontal diseases	Rate	2018	1172.82	1377.40	1020.19
1311	Incidence	Republic of Turkey	Both	Age-standardized	Periodontal diseases	Rate	2018	1190.65	1379.95	1035.95
1312	Incidence	Republic of Turkey	Male	Age-standardized	Periodontal diseases	Rate	2019	1207.48	1401.63	1038.32
1313	Incidence	Republic of Turkey	Female	Age-standardized	Periodontal diseases	Rate	2019	1176.04	1387.24	1020.00
1314	Incidence	Republic of Turkey	Both	Age-standardized	Periodontal diseases	Rate	2019	1192.11	1384.46	1038.00
1315	Incidence	Republic of Turkey	Male	Age-standardized	Periodontal diseases	Rate	2020	1204.51	1401.54	1037.06
1316	Incidence	Republic of Turkey	Female	Age-standardized	Periodontal diseases	Rate	2020	1176.49	1386.88	1013.85
1317	Incidence	Republic of Turkey	Both	Age-standardized	Periodontal diseases	Rate	2020	1190.86	1392.35	1033.43
1318	Incidence	Republic of Turkey	Male	Age-standardized	Periodontal diseases	Rate	2021	1205.58	1418.70	1047.19
1319	Incidence	Republic of Turkey	Female	Age-standardized	Periodontal diseases	Rate	2021	1177.16	1408.23	1023.22
1320	Incidence	Republic of Turkey	Both	Age-standardized	Periodontal diseases	Rate	2021	1191.61	1416.76	1034.48

1321	Incidence	Republic of Turkey	Male	Age-standardized	Periodontal diseases	Rate	2022	1204.94	1407.97	1046.99
1322	Incidence	Republic of Turkey	Female	Age-standardized	Periodontal diseases	Rate	2022	1173.16	1393.46	1014.25
1323	Incidence	Republic of Turkey	Both	Age-standardized	Periodontal diseases	Rate	2022	1189.32	1389.60	1032.80
1324	Incidence	Republic of Turkey	Male	Age-standardized	Periodontal diseases	Rate	2023	1179.74	1379.90	1024.65
1325	Incidence	Republic of Turkey	Female	Age-standardized	Periodontal diseases	Rate	2023	1148.55	1364.63	992.10
1326	Incidence	Republic of Turkey	Both	Age-standardized	Periodontal diseases	Rate	2023	1164.41	1361.38	1010.25
1327	Incidence	Canada	Male	Age-standardized	Periodontal diseases	Rate	1990	1233.54	1424.45	1049.94
1328	Incidence	Canada	Female	Age-standardized	Periodontal diseases	Rate	1990	1121.75	1322.91	905.87
1329	Incidence	Canada	Both	Age-standardized	Periodontal diseases	Rate	1990	1177.66	1370.81	980.26
1330	Incidence	Canada	Male	Age-standardized	Periodontal diseases	Rate	1991	1226.30	1378.32	1059.38
1331	Incidence	Canada	Female	Age-standardized	Periodontal diseases	Rate	1991	1100.09	1239.12	927.76
1332	Incidence	Canada	Both	Age-standardized	Periodontal diseases	Rate	1991	1163.25	1306.79	989.88
1333	Incidence	Canada	Male	Age-standardized	Periodontal diseases	Rate	1992	1220.39	1351.25	1066.47
1334	Incidence	Canada	Female	Age-standardized	Periodontal diseases	Rate	1992	1083.28	1192.46	964.48
1335	Incidence	Canada	Both	Age-standardized	Periodontal diseases	Rate	1992	1151.89	1267.00	1015.27
1336	Incidence	Canada	Male	Age-standardized	Periodontal diseases	Rate	1993	1215.92	1334.57	1082.13
1337	Incidence	Canada	Female	Age-standardized	Periodontal diseases	Rate	1993	1071.22	1181.65	970.76
1338	Incidence	Canada	Both	Age-standardized	Periodontal diseases	Rate	1993	1143.61	1239.77	1031.62
1339	Incidence	Canada	Male	Age-standardized	Periodontal diseases	Rate	1994	1212.99	1326.78	1083.62
1340	Incidence	Canada	Female	Age-standardized	Periodontal diseases	Rate	1994	1063.83	1182.60	965.13
1341	Incidence	Canada	Both	Age-standardized	Periodontal diseases	Rate	1994	1138.40	1230.79	1035.54
1342	Incidence	Canada	Male	Age-standardized	Periodontal diseases	Rate	1995	1211.72	1323.52	1083.41

1343	Incidence	Canada	Female	Age-standardized	Periodontal diseases	Rate	1995	1061.01	1186.14	932.67
1344	Incidence	Canada	Both	Age-standardized	Periodontal diseases	Rate	1995	1136.27	1229.51	1044.38
1345	Incidence	Canada	Male	Age-standardized	Periodontal diseases	Rate	1996	1212.10	1308.72	1100.33
1346	Incidence	Canada	Female	Age-standardized	Periodontal diseases	Rate	1996	1066.44	1195.74	941.58
1347	Incidence	Canada	Both	Age-standardized	Periodontal diseases	Rate	1996	1139.10	1237.81	1047.89
1348	Incidence	Canada	Male	Age-standardized	Periodontal diseases	Rate	1997	1213.63	1319.03	1110.09
1349	Incidence	Canada	Female	Age-standardized	Periodontal diseases	Rate	1997	1080.29	1216.86	950.94
1350	Incidence	Canada	Both	Age-standardized	Periodontal diseases	Rate	1997	1146.75	1259.20	1050.72
1351	Incidence	Canada	Male	Age-standardized	Periodontal diseases	Rate	1998	1215.65	1344.23	1097.34
1352	Incidence	Canada	Female	Age-standardized	Periodontal diseases	Rate	1998	1097.20	1232.54	960.08
1353	Incidence	Canada	Both	Age-standardized	Periodontal diseases	Rate	1998	1156.19	1278.65	1041.56
1354	Incidence	Canada	Male	Age-standardized	Periodontal diseases	Rate	1999	1217.51	1378.37	1082.75
1355	Incidence	Canada	Female	Age-standardized	Periodontal diseases	Rate	1999	1111.84	1266.17	955.09
1356	Incidence	Canada	Both	Age-standardized	Periodontal diseases	Rate	1999	1164.42	1328.27	1032.77
1357	Incidence	Canada	Male	Age-standardized	Periodontal diseases	Rate	2000	1218.55	1414.79	1059.38
1358	Incidence	Canada	Female	Age-standardized	Periodontal diseases	Rate	2000	1118.85	1311.15	950.93
1359	Incidence	Canada	Both	Age-standardized	Periodontal diseases	Rate	2000	1168.42	1370.36	1015.79
1360	Incidence	Canada	Male	Age-standardized	Periodontal diseases	Rate	2001	1219.56	1412.07	1064.46
1361	Incidence	Canada	Female	Age-standardized	Periodontal diseases	Rate	2001	1120.31	1325.19	951.95
1362	Incidence	Canada	Both	Age-standardized	Periodontal diseases	Rate	2001	1169.62	1376.35	1019.34
1363	Incidence	Canada	Male	Age-standardized	Periodontal diseases	Rate	2002	1221.39	1410.64	1065.82
1364	Incidence	Canada	Female	Age-standardized	Periodontal diseases	Rate	2002	1121.80	1338.81	953.02

1365	Incidence	Canada	Both	Age-standardized	Periodontal diseases	Rate	2002	1171.25	1380.49	1023.34
1366	Incidence	Canada	Male	Age-standardized	Periodontal diseases	Rate	2003	1223.49	1411.24	1077.82
1367	Incidence	Canada	Female	Age-standardized	Periodontal diseases	Rate	2003	1123.17	1348.58	955.86
1368	Incidence	Canada	Both	Age-standardized	Periodontal diseases	Rate	2003	1172.96	1383.07	1023.68
1369	Incidence	Canada	Male	Age-standardized	Periodontal diseases	Rate	2004	1225.29	1411.17	1083.89
1370	Incidence	Canada	Female	Age-standardized	Periodontal diseases	Rate	2004	1124.30	1354.07	959.57
1371	Incidence	Canada	Both	Age-standardized	Periodontal diseases	Rate	2004	1174.40	1384.94	1026.86
1372	Incidence	Canada	Male	Age-standardized	Periodontal diseases	Rate	2005	1226.24	1410.07	1088.90
1373	Incidence	Canada	Female	Age-standardized	Periodontal diseases	Rate	2005	1125.03	1366.31	962.83
1374	Incidence	Canada	Both	Age-standardized	Periodontal diseases	Rate	2005	1175.22	1387.37	1029.33
1375	Incidence	Canada	Male	Age-standardized	Periodontal diseases	Rate	2006	1226.52	1411.61	1091.27
1376	Incidence	Canada	Female	Age-standardized	Periodontal diseases	Rate	2006	1125.73	1363.71	963.97
1377	Incidence	Canada	Both	Age-standardized	Periodontal diseases	Rate	2006	1175.70	1386.82	1030.03
1378	Incidence	Canada	Male	Age-standardized	Periodontal diseases	Rate	2007	1226.71	1413.20	1088.25
1379	Incidence	Canada	Female	Age-standardized	Periodontal diseases	Rate	2007	1126.68	1358.95	975.79
1380	Incidence	Canada	Both	Age-standardized	Periodontal diseases	Rate	2007	1176.28	1386.92	1034.09
1381	Incidence	Canada	Male	Age-standardized	Periodontal diseases	Rate	2008	1226.83	1412.85	1083.72
1382	Incidence	Canada	Female	Age-standardized	Periodontal diseases	Rate	2008	1127.69	1355.62	978.41
1383	Incidence	Canada	Both	Age-standardized	Periodontal diseases	Rate	2008	1176.87	1384.63	1033.30
1384	Incidence	Canada	Male	Age-standardized	Periodontal diseases	Rate	2009	1226.93	1413.82	1081.71
1385	Incidence	Canada	Female	Age-standardized	Periodontal diseases	Rate	2009	1128.52	1358.05	983.67
1386	Incidence	Canada	Both	Age-standardized	Periodontal diseases	Rate	2009	1177.38	1384.11	1036.48

1387	Incidence	Canada	Male	Age-standardized	Periodontal diseases	Rate	2010	1227.03	1416.26	1078.10
1388	Incidence	Canada	Female	Age-standardized	Periodontal diseases	Rate	2010	1128.98	1356.83	982.76
1389	Incidence	Canada	Both	Age-standardized	Periodontal diseases	Rate	2010	1177.69	1384.18	1035.05
1390	Incidence	Canada	Male	Age-standardized	Periodontal diseases	Rate	2011	1227.14	1420.82	1080.85
1391	Incidence	Canada	Female	Age-standardized	Periodontal diseases	Rate	2011	1129.08	1358.02	984.42
1392	Incidence	Canada	Both	Age-standardized	Periodontal diseases	Rate	2011	1177.83	1386.30	1036.96
1393	Incidence	Canada	Male	Age-standardized	Periodontal diseases	Rate	2012	1227.22	1424.58	1082.32
1394	Incidence	Canada	Female	Age-standardized	Periodontal diseases	Rate	2012	1129.09	1356.78	981.22
1395	Incidence	Canada	Both	Age-standardized	Periodontal diseases	Rate	2012	1177.91	1389.09	1033.85
1396	Incidence	Canada	Male	Age-standardized	Periodontal diseases	Rate	2013	1227.27	1420.20	1079.56
1397	Incidence	Canada	Female	Age-standardized	Periodontal diseases	Rate	2013	1129.12	1360.55	978.85
1398	Incidence	Canada	Both	Age-standardized	Periodontal diseases	Rate	2013	1177.98	1389.83	1029.56
1399	Incidence	Canada	Male	Age-standardized	Periodontal diseases	Rate	2014	1227.28	1420.37	1078.82
1400	Incidence	Canada	Female	Age-standardized	Periodontal diseases	Rate	2014	1129.28	1353.91	975.05
1401	Incidence	Canada	Both	Age-standardized	Periodontal diseases	Rate	2014	1178.12	1385.93	1027.38
1402	Incidence	Canada	Male	Age-standardized	Periodontal diseases	Rate	2015	1227.25	1423.51	1075.19
1403	Incidence	Canada	Female	Age-standardized	Periodontal diseases	Rate	2015	1129.69	1348.42	971.29
1404	Incidence	Canada	Both	Age-standardized	Periodontal diseases	Rate	2015	1178.37	1384.52	1027.28
1405	Incidence	Canada	Male	Age-standardized	Periodontal diseases	Rate	2016	1226.85	1418.84	1074.45
1406	Incidence	Canada	Female	Age-standardized	Periodontal diseases	Rate	2016	1131.32	1349.65	971.88
1407	Incidence	Canada	Both	Age-standardized	Periodontal diseases	Rate	2016	1179.05	1383.34	1027.72
1408	Incidence	Canada	Male	Age-standardized	Periodontal diseases	Rate	2017	1226.05	1420.46	1073.72

1409	Incidence	Canada	Female	Age-standardized	Periodontal diseases	Rate	2017	1134.16	1354.76	971.56
1410	Incidence	Canada	Both	Age-standardized	Periodontal diseases	Rate	2017	1180.16	1384.83	1026.28
1411	Incidence	Canada	Male	Age-standardized	Periodontal diseases	Rate	2018	1225.23	1427.16	1073.74
1412	Incidence	Canada	Female	Age-standardized	Periodontal diseases	Rate	2018	1136.91	1355.66	974.82
1413	Incidence	Canada	Both	Age-standardized	Periodontal diseases	Rate	2018	1181.21	1388.18	1024.89
1414	Incidence	Canada	Male	Age-standardized	Periodontal diseases	Rate	2019	1224.79	1429.13	1069.77
1415	Incidence	Canada	Female	Age-standardized	Periodontal diseases	Rate	2019	1138.25	1354.49	973.94
1416	Incidence	Canada	Both	Age-standardized	Periodontal diseases	Rate	2019	1181.74	1386.31	1024.95
1417	Incidence	Canada	Male	Age-standardized	Periodontal diseases	Rate	2020	1226.27	1410.62	1062.92
1418	Incidence	Canada	Female	Age-standardized	Periodontal diseases	Rate	2020	1134.48	1332.64	977.21
1419	Incidence	Canada	Both	Age-standardized	Periodontal diseases	Rate	2020	1180.71	1357.12	1022.56
1420	Incidence	Canada	Male	Age-standardized	Periodontal diseases	Rate	2021	1229.89	1405.07	1083.94
1421	Incidence	Canada	Female	Age-standardized	Periodontal diseases	Rate	2021	1134.89	1356.83	982.61
1422	Incidence	Canada	Both	Age-standardized	Periodontal diseases	Rate	2021	1182.84	1380.02	1033.67
1423	Incidence	Canada	Male	Age-standardized	Periodontal diseases	Rate	2022	1225.42	1417.20	1068.05
1424	Incidence	Canada	Female	Age-standardized	Periodontal diseases	Rate	2022	1137.79	1345.24	976.90
1425	Incidence	Canada	Both	Age-standardized	Periodontal diseases	Rate	2022	1182.12	1371.22	1024.02
1426	Incidence	Canada	Male	Age-standardized	Periodontal diseases	Rate	2023	1219.93	1410.92	1063.27
1427	Incidence	Canada	Female	Age-standardized	Periodontal diseases	Rate	2023	1132.68	1338.94	972.56
1428	Incidence	Canada	Both	Age-standardized	Periodontal diseases	Rate	2023	1176.91	1366.00	1019.37
1429	Incidence	Federative Republic of Brazil	Male	Age-standardized	Periodontal diseases	Rate	1990	1120.23	1304.99	904.01
1430	Incidence	Federative	Female	Age-standardized	Periodontal	Rate	1990	1046.03	1220.70	838.71

		Republic of Brazil			diseases					
1431	Incidence	Federative Republic of Brazil	Both	Age-standardized	Periodontal diseases	Rate	1990	1082.59	1264.88	871.07
1432	Incidence	Federative Republic of Brazil	Male	Age-standardized	Periodontal diseases	Rate	1991	1106.13	1292.42	891.43
1433	Incidence	Federative Republic of Brazil	Female	Age-standardized	Periodontal diseases	Rate	1991	1039.65	1215.05	834.07
1434	Incidence	Federative Republic of Brazil	Both	Age-standardized	Periodontal diseases	Rate	1991	1072.37	1252.12	864.89
1435	Incidence	Federative Republic of Brazil	Male	Age-standardized	Periodontal diseases	Rate	1992	1096.81	1282.28	884.83
1436	Incidence	Federative Republic of Brazil	Female	Age-standardized	Periodontal diseases	Rate	1992	1035.83	1213.54	831.34
1437	Incidence	Federative Republic of Brazil	Both	Age-standardized	Periodontal diseases	Rate	1992	1065.82	1246.49	859.99
1438	Incidence	Federative Republic of Brazil	Male	Age-standardized	Periodontal diseases	Rate	1993	1091.27	1277.45	880.07
1439	Incidence	Federative Republic of Brazil	Female	Age-standardized	Periodontal diseases	Rate	1993	1033.91	1211.86	829.99
1440	Incidence	Federative Republic of Brazil	Both	Age-standardized	Periodontal diseases	Rate	1993	1062.11	1243.47	856.39
1441	Incidence	Federative Republic of Brazil	Male	Age-standardized	Periodontal diseases	Rate	1994	1088.48	1274.97	876.77
1442	Incidence	Federative Republic of Brazil	Female	Age-standardized	Periodontal diseases	Rate	1994	1033.20	1209.52	829.46
1443	Incidence	Federative Republic of Brazil	Both	Age-standardized	Periodontal diseases	Rate	1994	1060.37	1242.39	854.11
1444	Incidence	Federative Republic of Brazil	Male	Age-standardized	Periodontal diseases	Rate	1995	1087.36	1274.94	874.46

1445	Incidence	Federative Republic of Brazil	Female	Age-standardized	Periodontal diseases	Rate	1995	1033.06	1208.67	829.14
1446	Incidence	Federative Republic of Brazil	Both	Age-standardized	Periodontal diseases	Rate	1995	1059.75	1242.94	852.67
1447	Incidence	Federative Republic of Brazil	Male	Age-standardized	Periodontal diseases	Rate	1996	1104.17	1290.85	882.96
1448	Incidence	Federative Republic of Brazil	Female	Age-standardized	Periodontal diseases	Rate	1996	1051.81	1223.27	843.81
1449	Incidence	Federative Republic of Brazil	Both	Age-standardized	Periodontal diseases	Rate	1996	1077.57	1257.81	863.75
1450	Incidence	Federative Republic of Brazil	Male	Age-standardized	Periodontal diseases	Rate	1997	1145.22	1333.31	907.62
1451	Incidence	Federative Republic of Brazil	Female	Age-standardized	Periodontal diseases	Rate	1997	1096.78	1277.35	873.25
1452	Incidence	Federative Republic of Brazil	Both	Age-standardized	Periodontal diseases	Rate	1997	1120.65	1305.21	889.24
1453	Incidence	Federative Republic of Brazil	Male	Age-standardized	Periodontal diseases	Rate	1998	1194.49	1386.42	938.27
1454	Incidence	Federative Republic of Brazil	Female	Age-standardized	Periodontal diseases	Rate	1998	1150.56	1344.26	906.87
1455	Incidence	Federative Republic of Brazil	Both	Age-standardized	Periodontal diseases	Rate	1998	1172.26	1367.50	922.33
1456	Incidence	Federative Republic of Brazil	Male	Age-standardized	Periodontal diseases	Rate	1999	1235.83	1436.54	962.49
1457	Incidence	Federative Republic of Brazil	Female	Age-standardized	Periodontal diseases	Rate	1999	1195.61	1400.06	936.74
1458	Incidence	Federative Republic of Brazil	Both	Age-standardized	Periodontal diseases	Rate	1999	1215.52	1413.70	950.28
1459	Incidence	Federative Republic of	Male	Age-standardized	Periodontal diseases	Rate	2000	1252.96	1455.46	966.81

		Brazil								
1460	Incidence	Federative Republic of Brazil	Female	Age-standardized	Periodontal diseases	Rate	2000	1214.28	1420.43	949.60
1461	Incidence	Federative Republic of Brazil	Both	Age-standardized	Periodontal diseases	Rate	2000	1233.48	1431.16	958.32
1462	Incidence	Federative Republic of Brazil	Male	Age-standardized	Periodontal diseases	Rate	2001	1235.47	1435.72	958.93
1463	Incidence	Federative Republic of Brazil	Female	Age-standardized	Periodontal diseases	Rate	2001	1194.66	1399.12	934.85
1464	Incidence	Federative Republic of Brazil	Both	Age-standardized	Periodontal diseases	Rate	2001	1214.93	1416.56	947.22
1465	Incidence	Federative Republic of Brazil	Male	Age-standardized	Periodontal diseases	Rate	2002	1194.34	1392.00	929.91
1466	Incidence	Federative Republic of Brazil	Female	Age-standardized	Periodontal diseases	Rate	2002	1148.42	1349.17	900.17
1467	Incidence	Federative Republic of Brazil	Both	Age-standardized	Periodontal diseases	Rate	2002	1171.23	1371.19	914.94
1468	Incidence	Federative Republic of Brazil	Male	Age-standardized	Periodontal diseases	Rate	2003	1145.42	1341.59	897.80
1469	Incidence	Federative Republic of Brazil	Female	Age-standardized	Periodontal diseases	Rate	2003	1093.39	1292.89	859.36
1470	Incidence	Federative Republic of Brazil	Both	Age-standardized	Periodontal diseases	Rate	2003	1119.24	1317.17	878.44
1471	Incidence	Federative Republic of Brazil	Male	Age-standardized	Periodontal diseases	Rate	2004	1104.75	1298.90	868.92
1472	Incidence	Federative Republic of Brazil	Female	Age-standardized	Periodontal diseases	Rate	2004	1047.52	1247.67	825.53
1473	Incidence	Federative Republic of Brazil	Both	Age-standardized	Periodontal diseases	Rate	2004	1075.95	1272.90	847.17
1474	Incidence	Federative	Male	Age-standardized	Periodontal	Rate	2005	1088.50	1287.78	857.65

		Republic of Brazil			diseases					
1475	Incidence	Federative Republic of Brazil	Female	Age-standardized	Periodontal diseases	Rate	2005	1028.98	1231.53	812.35
1476	Incidence	Federative Republic of Brazil	Both	Age-standardized	Periodontal diseases	Rate	2005	1058.56	1257.94	834.76
1477	Incidence	Federative Republic of Brazil	Male	Age-standardized	Periodontal diseases	Rate	2006	1100.50	1295.36	872.87
1478	Incidence	Federative Republic of Brazil	Female	Age-standardized	Periodontal diseases	Rate	2006	1040.31	1237.81	826.76
1479	Incidence	Federative Republic of Brazil	Both	Age-standardized	Periodontal diseases	Rate	2006	1070.26	1266.24	849.77
1480	Incidence	Federative Republic of Brazil	Male	Age-standardized	Periodontal diseases	Rate	2007	1127.43	1321.18	903.25
1481	Incidence	Federative Republic of Brazil	Female	Age-standardized	Periodontal diseases	Rate	2007	1066.02	1257.69	855.63
1482	Incidence	Federative Republic of Brazil	Both	Age-standardized	Periodontal diseases	Rate	2007	1096.63	1289.88	879.60
1483	Incidence	Federative Republic of Brazil	Male	Age-standardized	Periodontal diseases	Rate	2008	1159.93	1351.69	932.18
1484	Incidence	Federative Republic of Brazil	Female	Age-standardized	Periodontal diseases	Rate	2008	1097.32	1283.38	884.32
1485	Incidence	Federative Republic of Brazil	Both	Age-standardized	Periodontal diseases	Rate	2008	1128.58	1316.91	908.54
1486	Incidence	Federative Republic of Brazil	Male	Age-standardized	Periodontal diseases	Rate	2009	1188.55	1373.61	957.04
1487	Incidence	Federative Republic of Brazil	Female	Age-standardized	Periodontal diseases	Rate	2009	1125.35	1306.72	908.74
1488	Incidence	Federative Republic of Brazil	Both	Age-standardized	Periodontal diseases	Rate	2009	1156.95	1344.87	933.53

1489	Incidence	Federative Republic of Brazil	Male	Age-standardized	Periodontal diseases	Rate	2010	1203.78	1390.70	971.91
1490	Incidence	Federative Republic of Brazil	Female	Age-standardized	Periodontal diseases	Rate	2010	1141.25	1317.36	923.04
1491	Incidence	Federative Republic of Brazil	Both	Age-standardized	Periodontal diseases	Rate	2010	1172.54	1354.51	947.22
1492	Incidence	Federative Republic of Brazil	Male	Age-standardized	Periodontal diseases	Rate	2011	1206.88	1396.66	971.80
1493	Incidence	Federative Republic of Brazil	Female	Age-standardized	Periodontal diseases	Rate	2011	1146.71	1324.52	925.80
1494	Incidence	Federative Republic of Brazil	Both	Age-standardized	Periodontal diseases	Rate	2011	1176.83	1360.90	948.51
1495	Incidence	Federative Republic of Brazil	Male	Age-standardized	Periodontal diseases	Rate	2012	1206.57	1397.82	968.32
1496	Incidence	Federative Republic of Brazil	Female	Age-standardized	Periodontal diseases	Rate	2012	1149.85	1332.50	926.47
1497	Incidence	Federative Republic of Brazil	Both	Age-standardized	Periodontal diseases	Rate	2012	1178.24	1365.55	946.69
1498	Incidence	Federative Republic of Brazil	Male	Age-standardized	Periodontal diseases	Rate	2013	1204.44	1398.13	963.12
1499	Incidence	Federative Republic of Brazil	Female	Age-standardized	Periodontal diseases	Rate	2013	1151.35	1340.43	925.22
1500	Incidence	Federative Republic of Brazil	Both	Age-standardized	Periodontal diseases	Rate	2013	1177.91	1366.55	943.15
1501	Incidence	Federative Republic of Brazil	Male	Age-standardized	Periodontal diseases	Rate	2014	1202.07	1396.99	957.91
1502	Incidence	Federative Republic of Brazil	Female	Age-standardized	Periodontal diseases	Rate	2014	1151.84	1346.87	923.83
1503	Incidence	Federative Republic of	Both	Age-standardized	Periodontal diseases	Rate	2014	1176.95	1366.58	939.37

		Brazil								
1504	Incidence	Federative Republic of Brazil	Male	Age-standardized	Periodontal diseases	Rate	2015	1201.03	1396.50	954.40
1505	Incidence	Federative Republic of Brazil	Female	Age-standardized	Periodontal diseases	Rate	2015	1151.93	1350.10	923.60
1506	Incidence	Federative Republic of Brazil	Both	Age-standardized	Periodontal diseases	Rate	2015	1176.46	1366.67	936.84
1507	Incidence	Federative Republic of Brazil	Male	Age-standardized	Periodontal diseases	Rate	2016	1201.31	1399.46	957.62
1508	Incidence	Federative Republic of Brazil	Female	Age-standardized	Periodontal diseases	Rate	2016	1151.98	1349.43	921.53
1509	Incidence	Federative Republic of Brazil	Both	Age-standardized	Periodontal diseases	Rate	2016	1176.61	1367.29	938.61
1510	Incidence	Federative Republic of Brazil	Male	Age-standardized	Periodontal diseases	Rate	2017	1201.74	1400.53	961.05
1511	Incidence	Federative Republic of Brazil	Female	Age-standardized	Periodontal diseases	Rate	2017	1151.94	1347.92	920.27
1512	Incidence	Federative Republic of Brazil	Both	Age-standardized	Periodontal diseases	Rate	2017	1176.78	1367.89	940.51
1513	Incidence	Federative Republic of Brazil	Male	Age-standardized	Periodontal diseases	Rate	2018	1202.07	1400.70	964.44
1514	Incidence	Federative Republic of Brazil	Female	Age-standardized	Periodontal diseases	Rate	2018	1151.85	1346.91	920.28
1515	Incidence	Federative Republic of Brazil	Both	Age-standardized	Periodontal diseases	Rate	2018	1176.88	1368.38	942.39
1516	Incidence	Federative Republic of Brazil	Male	Age-standardized	Periodontal diseases	Rate	2019	1202.07	1400.62	967.45
1517	Incidence	Federative Republic of Brazil	Female	Age-standardized	Periodontal diseases	Rate	2019	1151.76	1345.86	919.04
1518	Incidence	Federative	Both	Age-standardized	Periodontal	Rate	2019	1176.82	1368.74	944.07

		Republic of Brazil			diseases					
1519	Incidence	Federative Republic of Brazil	Male	Age-standardized	Periodontal diseases	Rate	2020	1198.16	1383.63	958.12
1520	Incidence	Federative Republic of Brazil	Female	Age-standardized	Periodontal diseases	Rate	2020	1149.91	1329.84	939.18
1521	Incidence	Federative Republic of Brazil	Both	Age-standardized	Periodontal diseases	Rate	2020	1173.96	1355.04	950.93
1522	Incidence	Federative Republic of Brazil	Male	Age-standardized	Periodontal diseases	Rate	2021	1196.29	1341.90	1035.47
1523	Incidence	Federative Republic of Brazil	Female	Age-standardized	Periodontal diseases	Rate	2021	1144.58	1289.14	989.13
1524	Incidence	Federative Republic of Brazil	Both	Age-standardized	Periodontal diseases	Rate	2021	1170.33	1315.19	1011.26
1525	Incidence	Federative Republic of Brazil	Male	Age-standardized	Periodontal diseases	Rate	2022	1196.90	1345.94	1029.27
1526	Incidence	Federative Republic of Brazil	Female	Age-standardized	Periodontal diseases	Rate	2022	1145.14	1291.18	986.20
1527	Incidence	Federative Republic of Brazil	Both	Age-standardized	Periodontal diseases	Rate	2022	1170.94	1315.30	1008.40
1528	Incidence	Federative Republic of Brazil	Male	Age-standardized	Periodontal diseases	Rate	2023	1183.03	1329.92	1018.52
1529	Incidence	Federative Republic of Brazil	Female	Age-standardized	Periodontal diseases	Rate	2023	1131.85	1275.88	975.13
1530	Incidence	Federative Republic of Brazil	Both	Age-standardized	Periodontal diseases	Rate	2023	1157.38	1299.98	997.81
1531	Incidence	United Kingdom of Great Britain and Northern Ireland	Male	Age-standardized	Periodontal diseases	Rate	1990	611.94	777.14	431.35
1532	Incidence	United	Female	Age-standardized	Periodontal	Rate	1990	616.26	779.11	439.70

		Kingdom of Great Britain and Northern Ireland			diseases					
1533	Incidence	United Kingdom of Great Britain and Northern Ireland	Both	Age-standardized	Periodontal diseases	Rate	1990	614.52	778.53	436.04
1534	Incidence	United Kingdom of Great Britain and Northern Ireland	Male	Age-standardized	Periodontal diseases	Rate	1991	613.29	777.24	434.91
1535	Incidence	United Kingdom of Great Britain and Northern Ireland	Female	Age-standardized	Periodontal diseases	Rate	1991	621.28	774.66	449.47
1536	Incidence	United Kingdom of Great Britain and Northern Ireland	Both	Age-standardized	Periodontal diseases	Rate	1991	617.85	778.54	443.32
1537	Incidence	United Kingdom of Great Britain and Northern Ireland	Male	Age-standardized	Periodontal diseases	Rate	1992	612.99	775.27	436.68
1538	Incidence	United Kingdom of Great Britain and Northern Ireland	Female	Age-standardized	Periodontal diseases	Rate	1992	623.35	765.31	457.99
1539	Incidence	United Kingdom of Great Britain and Northern Ireland	Both	Age-standardized	Periodontal diseases	Rate	1992	618.82	771.73	447.65
1540	Incidence	United Kingdom of Great Britain and Northern Ireland	Male	Age-standardized	Periodontal diseases	Rate	1993	611.35	771.66	437.06

1541	Incidence	United Kingdom of Great Britain and Northern Ireland	Female	Age-standardized	Periodontal diseases	Rate	1993	622.99	760.94	462.00
1542	Incidence	United Kingdom of Great Britain and Northern Ireland	Both	Age-standardized	Periodontal diseases	Rate	1993	617.88	763.82	449.96
1543	Incidence	United Kingdom of Great Britain and Northern Ireland	Male	Age-standardized	Periodontal diseases	Rate	1994	608.73	766.87	436.50
1544	Incidence	United Kingdom of Great Britain and Northern Ireland	Female	Age-standardized	Periodontal diseases	Rate	1994	620.89	756.30	462.98
1545	Incidence	United Kingdom of Great Britain and Northern Ireland	Both	Age-standardized	Periodontal diseases	Rate	1994	615.53	755.12	451.24
1546	Incidence	United Kingdom of Great Britain and Northern Ireland	Male	Age-standardized	Periodontal diseases	Rate	1995	605.35	760.46	435.22
1547	Incidence	United Kingdom of Great Britain and Northern Ireland	Female	Age-standardized	Periodontal diseases	Rate	1995	617.50	748.59	465.18
1548	Incidence	United Kingdom of Great Britain and Northern Ireland	Both	Age-standardized	Periodontal diseases	Rate	1995	612.14	747.42	450.23
1549	Incidence	United Kingdom of Great Britain and Northern	Male	Age-standardized	Periodontal diseases	Rate	1996	586.57	719.13	429.31

		Ireland								
1550	Incidence	United Kingdom of Great Britain and Northern Ireland	Female	Age-standardized	Periodontal diseases	Rate	1996	599.49	721.56	459.14
1551	Incidence	United Kingdom of Great Britain and Northern Ireland	Both	Age-standardized	Periodontal diseases	Rate	1996	593.70	718.19	442.03
1552	Incidence	United Kingdom of Great Britain and Northern Ireland	Male	Age-standardized	Periodontal diseases	Rate	1997	547.09	661.27	407.18
1553	Incidence	United Kingdom of Great Britain and Northern Ireland	Female	Age-standardized	Periodontal diseases	Rate	1997	562.05	670.61	437.44
1554	Incidence	United Kingdom of Great Britain and Northern Ireland	Both	Age-standardized	Periodontal diseases	Rate	1997	555.17	665.28	423.77
1555	Incidence	United Kingdom of Great Britain and Northern Ireland	Male	Age-standardized	Periodontal diseases	Rate	1998	501.16	599.56	384.91
1556	Incidence	United Kingdom of Great Britain and Northern Ireland	Female	Age-standardized	Periodontal diseases	Rate	1998	518.62	614.39	408.28
1557	Incidence	United Kingdom of Great Britain and Northern Ireland	Both	Age-standardized	Periodontal diseases	Rate	1998	510.44	606.27	398.16
1558	Incidence	United Kingdom of Great Britain	Male	Age-standardized	Periodontal diseases	Rate	1999	463.11	550.48	363.77

		and Northern Ireland								
1559	Incidence	United Kingdom of Great Britain and Northern Ireland	Female	Age-standardized	Periodontal diseases	Rate	1999	482.67	573.26	383.58
1560	Incidence	United Kingdom of Great Britain and Northern Ireland	Both	Age-standardized	Periodontal diseases	Rate	1999	473.39	561.53	374.27
1561	Incidence	United Kingdom of Great Britain and Northern Ireland	Male	Age-standardized	Periodontal diseases	Rate	2000	447.32	533.29	355.25
1562	Incidence	United Kingdom of Great Britain and Northern Ireland	Female	Age-standardized	Periodontal diseases	Rate	2000	467.75	557.75	373.76
1563	Incidence	United Kingdom of Great Britain and Northern Ireland	Both	Age-standardized	Periodontal diseases	Rate	2000	457.99	545.96	364.84
1564	Incidence	United Kingdom of Great Britain and Northern Ireland	Male	Age-standardized	Periodontal diseases	Rate	2001	461.52	551.45	365.59
1565	Incidence	United Kingdom of Great Britain and Northern Ireland	Female	Age-standardized	Periodontal diseases	Rate	2001	483.71	576.10	387.40
1566	Incidence	United Kingdom of Great Britain and Northern Ireland	Both	Age-standardized	Periodontal diseases	Rate	2001	473.10	565.11	376.98
1567	Incidence	United Kingdom of	Male	Age-standardized	Periodontal diseases	Rate	2002	495.38	593.63	391.01

		Great Britain and Northern Ireland								
1568	Incidence	United Kingdom of Great Britain and Northern Ireland	Female	Age-standardized	Periodontal diseases	Rate	2002	521.81	618.75	418.40
1569	Incidence	United Kingdom of Great Britain and Northern Ireland	Both	Age-standardized	Periodontal diseases	Rate	2002	509.16	605.68	405.28
1570	Incidence	United Kingdom of Great Britain and Northern Ireland	Male	Age-standardized	Periodontal diseases	Rate	2003	535.96	641.44	420.96
1571	Incidence	United Kingdom of Great Britain and Northern Ireland	Female	Age-standardized	Periodontal diseases	Rate	2003	567.54	670.56	455.27
1572	Incidence	United Kingdom of Great Britain and Northern Ireland	Both	Age-standardized	Periodontal diseases	Rate	2003	552.42	656.73	438.87
1573	Incidence	United Kingdom of Great Britain and Northern Ireland	Male	Age-standardized	Periodontal diseases	Rate	2004	570.37	682.09	445.64
1574	Incidence	United Kingdom of Great Britain and Northern Ireland	Female	Age-standardized	Periodontal diseases	Rate	2004	606.36	717.39	486.73
1575	Incidence	United Kingdom of Great Britain and Northern Ireland	Both	Age-standardized	Periodontal diseases	Rate	2004	589.09	702.37	467.02
1576	Incidence	United	Male	Age-standardized	Periodontal	Rate	2005	585.56	702.97	455.39

		Kingdom of Great Britain and Northern Ireland			diseases					
1577	Incidence	United Kingdom of Great Britain and Northern Ireland	Female	Age-standardized	Periodontal diseases	Rate	2005	623.71	740.90	500.43
1578	Incidence	United Kingdom of Great Britain and Northern Ireland	Both	Age-standardized	Periodontal diseases	Rate	2005	605.38	722.60	479.10
1579	Incidence	United Kingdom of Great Britain and Northern Ireland	Male	Age-standardized	Periodontal diseases	Rate	2006	585.71	703.72	457.39
1580	Incidence	United Kingdom of Great Britain and Northern Ireland	Female	Age-standardized	Periodontal diseases	Rate	2006	624.92	743.20	501.52
1581	Incidence	United Kingdom of Great Britain and Northern Ireland	Both	Age-standardized	Periodontal diseases	Rate	2006	606.02	722.58	480.82
1582	Incidence	United Kingdom of Great Britain and Northern Ireland	Male	Age-standardized	Periodontal diseases	Rate	2007	584.14	702.37	458.36
1583	Incidence	United Kingdom of Great Britain and Northern Ireland	Female	Age-standardized	Periodontal diseases	Rate	2007	625.22	742.92	502.06
1584	Incidence	United Kingdom of Great Britain and Northern Ireland	Both	Age-standardized	Periodontal diseases	Rate	2007	605.36	721.91	481.70

1585	Incidence	United Kingdom of Great Britain and Northern Ireland	Male	Age-standardized	Periodontal diseases	Rate	2008	581.71	695.66	458.68
1586	Incidence	United Kingdom of Great Britain and Northern Ireland	Female	Age-standardized	Periodontal diseases	Rate	2008	624.97	742.03	502.98
1587	Incidence	United Kingdom of Great Britain and Northern Ireland	Both	Age-standardized	Periodontal diseases	Rate	2008	603.99	720.65	481.52
1588	Incidence	United Kingdom of Great Britain and Northern Ireland	Male	Age-standardized	Periodontal diseases	Rate	2009	579.22	694.13	458.61
1589	Incidence	United Kingdom of Great Britain and Northern Ireland	Female	Age-standardized	Periodontal diseases	Rate	2009	624.53	741.33	504.46
1590	Incidence	United Kingdom of Great Britain and Northern Ireland	Both	Age-standardized	Periodontal diseases	Rate	2009	602.50	718.55	480.99
1591	Incidence	United Kingdom of Great Britain and Northern Ireland	Male	Age-standardized	Periodontal diseases	Rate	2010	577.54	694.19	458.29
1592	Incidence	United Kingdom of Great Britain and Northern Ireland	Female	Age-standardized	Periodontal diseases	Rate	2010	624.25	741.74	505.02
1593	Incidence	United Kingdom of Great Britain and Northern	Both	Age-standardized	Periodontal diseases	Rate	2010	601.51	718.10	480.52

		Ireland								
1594	Incidence	United Kingdom of Great Britain and Northern Ireland	Male	Age-standardized	Periodontal diseases	Rate	2011	576.06	691.46	458.48
1595	Incidence	United Kingdom of Great Britain and Northern Ireland	Female	Age-standardized	Periodontal diseases	Rate	2011	623.65	741.47	508.37
1596	Incidence	United Kingdom of Great Britain and Northern Ireland	Both	Age-standardized	Periodontal diseases	Rate	2011	600.47	716.83	483.92
1597	Incidence	United Kingdom of Great Britain and Northern Ireland	Male	Age-standardized	Periodontal diseases	Rate	2012	573.88	688.66	458.04
1598	Incidence	United Kingdom of Great Britain and Northern Ireland	Female	Age-standardized	Periodontal diseases	Rate	2012	622.29	743.18	507.41
1599	Incidence	United Kingdom of Great Britain and Northern Ireland	Both	Age-standardized	Periodontal diseases	Rate	2012	598.73	715.70	485.70
1600	Incidence	United Kingdom of Great Britain and Northern Ireland	Male	Age-standardized	Periodontal diseases	Rate	2013	571.36	685.79	457.29
1601	Incidence	United Kingdom of Great Britain and Northern Ireland	Female	Age-standardized	Periodontal diseases	Rate	2013	620.67	744.55	511.85
1602	Incidence	United Kingdom of Great Britain	Both	Age-standardized	Periodontal diseases	Rate	2013	596.69	715.39	484.42

		and Northern Ireland								
1603	Incidence	United Kingdom of Great Britain and Northern Ireland	Male	Age-standardized	Periodontal diseases	Rate	2014	568.85	684.08	456.88
1604	Incidence	United Kingdom of Great Britain and Northern Ireland	Female	Age-standardized	Periodontal diseases	Rate	2014	619.28	746.01	511.87
1605	Incidence	United Kingdom of Great Britain and Northern Ireland	Both	Age-standardized	Periodontal diseases	Rate	2014	594.77	715.43	485.57
1606	Incidence	United Kingdom of Great Britain and Northern Ireland	Male	Age-standardized	Periodontal diseases	Rate	2015	566.68	680.95	456.03
1607	Incidence	United Kingdom of Great Britain and Northern Ireland	Female	Age-standardized	Periodontal diseases	Rate	2015	618.62	747.36	515.42
1608	Incidence	United Kingdom of Great Britain and Northern Ireland	Both	Age-standardized	Periodontal diseases	Rate	2015	593.38	715.48	488.09
1609	Incidence	United Kingdom of Great Britain and Northern Ireland	Male	Age-standardized	Periodontal diseases	Rate	2016	563.27	680.02	456.67
1610	Incidence	United Kingdom of Great Britain and Northern Ireland	Female	Age-standardized	Periodontal diseases	Rate	2016	618.91	748.86	515.36
1611	Incidence	United Kingdom of	Both	Age-standardized	Periodontal diseases	Rate	2016	591.83	715.16	487.84

		Great Britain and Northern Ireland								
1612	Incidence	United Kingdom of Great Britain and Northern Ireland	Male	Age-standardized	Periodontal diseases	Rate	2017	557.88	677.31	456.76
1613	Incidence	United Kingdom of Great Britain and Northern Ireland	Female	Age-standardized	Periodontal diseases	Rate	2017	619.69	751.36	515.27
1614	Incidence	United Kingdom of Great Britain and Northern Ireland	Both	Age-standardized	Periodontal diseases	Rate	2017	589.56	715.01	487.53
1615	Incidence	United Kingdom of Great Britain and Northern Ireland	Male	Age-standardized	Periodontal diseases	Rate	2018	552.31	674.25	452.68
1616	Incidence	United Kingdom of Great Britain and Northern Ireland	Female	Age-standardized	Periodontal diseases	Rate	2018	620.44	753.47	515.49
1617	Incidence	United Kingdom of Great Britain and Northern Ireland	Both	Age-standardized	Periodontal diseases	Rate	2018	587.19	714.68	487.22
1618	Incidence	United Kingdom of Great Britain and Northern Ireland	Male	Age-standardized	Periodontal diseases	Rate	2019	548.32	672.56	453.17
1619	Incidence	United Kingdom of Great Britain and Northern Ireland	Female	Age-standardized	Periodontal diseases	Rate	2019	620.64	756.99	515.25
1620	Incidence	United	Both	Age-standardized	Periodontal	Rate	2019	585.35	714.51	486.06

		Kingdom of Great Britain and Northern Ireland			diseases					
1621	Incidence	United Kingdom of Great Britain and Northern Ireland	Male	Age-standardized	Periodontal diseases	Rate	2020	545.51	668.77	450.83
1622	Incidence	United Kingdom of Great Britain and Northern Ireland	Female	Age-standardized	Periodontal diseases	Rate	2020	618.31	753.49	513.37
1623	Incidence	United Kingdom of Great Britain and Northern Ireland	Both	Age-standardized	Periodontal diseases	Rate	2020	582.83	709.99	482.99
1624	Incidence	United Kingdom of Great Britain and Northern Ireland	Male	Age-standardized	Periodontal diseases	Rate	2021	545.66	671.34	449.76
1625	Incidence	United Kingdom of Great Britain and Northern Ireland	Female	Age-standardized	Periodontal diseases	Rate	2021	615.12	761.25	508.78
1626	Incidence	United Kingdom of Great Britain and Northern Ireland	Both	Age-standardized	Periodontal diseases	Rate	2021	581.32	713.12	479.55
1627	Incidence	United Kingdom of Great Britain and Northern Ireland	Male	Age-standardized	Periodontal diseases	Rate	2022	544.45	674.32	445.82
1628	Incidence	United Kingdom of Great Britain and Northern Ireland	Female	Age-standardized	Periodontal diseases	Rate	2022	619.18	768.20	510.64

1629	Incidence	United Kingdom of Great Britain and Northern Ireland	Both	Age-standardized	Periodontal diseases	Rate	2022	582.82	719.70	480.19
1630	Incidence	United Kingdom of Great Britain and Northern Ireland	Male	Age-standardized	Periodontal diseases	Rate	2023	541.00	670.07	442.88
1631	Incidence	United Kingdom of Great Britain and Northern Ireland	Female	Age-standardized	Periodontal diseases	Rate	2023	615.25	763.41	507.23
1632	Incidence	United Kingdom of Great Britain and Northern Ireland	Both	Age-standardized	Periodontal diseases	Rate	2023	579.16	715.24	477.02
1633	Incidence	Republic of India	Male	Age-standardized	Periodontal diseases	Rate	1990	1256.34	1460.66	999.31
1634	Incidence	Republic of India	Female	Age-standardized	Periodontal diseases	Rate	1990	1258.66	1462.70	994.52
1635	Incidence	Republic of India	Both	Age-standardized	Periodontal diseases	Rate	1990	1257.47	1461.33	997.28
1636	Incidence	Republic of India	Male	Age-standardized	Periodontal diseases	Rate	1991	1266.06	1472.56	1005.81
1637	Incidence	Republic of India	Female	Age-standardized	Periodontal diseases	Rate	1991	1265.61	1467.40	1000.23
1638	Incidence	Republic of India	Both	Age-standardized	Periodontal diseases	Rate	1991	1265.87	1471.12	1003.21
1639	Incidence	Republic of India	Male	Age-standardized	Periodontal diseases	Rate	1992	1274.83	1484.31	1010.79
1640	Incidence	Republic of India	Female	Age-standardized	Periodontal diseases	Rate	1992	1272.04	1472.03	1005.67
1641	Incidence	Republic of India	Both	Age-standardized	Periodontal diseases	Rate	1992	1273.51	1478.28	1008.31
1642	Incidence	Republic of India	Male	Age-standardized	Periodontal diseases	Rate	1993	1282.26	1494.11	1014.56
1643	Incidence	Republic of India	Female	Age-standardized	Periodontal diseases	Rate	1993	1277.66	1473.13	1014.20
1644	Incidence	Republic of India	Both	Age-standardized	Periodontal diseases	Rate	1993	1280.06	1484.19	1012.61

1645	Incidence	Republic of India	Male	Age-standardized	Periodontal diseases	Rate	1994	1287.98	1498.22	1017.13
1646	Incidence	Republic of India	Female	Age-standardized	Periodontal diseases	Rate	1994	1282.20	1474.13	1024.05
1647	Incidence	Republic of India	Both	Age-standardized	Periodontal diseases	Rate	1994	1285.21	1486.89	1015.86
1648	Incidence	Republic of India	Male	Age-standardized	Periodontal diseases	Rate	1995	1291.60	1499.79	1018.37
1649	Incidence	Republic of India	Female	Age-standardized	Periodontal diseases	Rate	1995	1285.39	1475.01	1032.97
1650	Incidence	Republic of India	Both	Age-standardized	Periodontal diseases	Rate	1995	1288.62	1488.07	1018.08
1651	Incidence	Republic of India	Male	Age-standardized	Periodontal diseases	Rate	1996	1293.41	1498.57	1022.02
1652	Incidence	Republic of India	Female	Age-standardized	Periodontal diseases	Rate	1996	1287.52	1475.39	1036.48
1653	Incidence	Republic of India	Both	Age-standardized	Periodontal diseases	Rate	1996	1290.57	1488.28	1022.01
1654	Incidence	Republic of India	Male	Age-standardized	Periodontal diseases	Rate	1997	1294.28	1496.58	1024.08
1655	Incidence	Republic of India	Female	Age-standardized	Periodontal diseases	Rate	1997	1289.15	1475.24	1039.77
1656	Incidence	Republic of India	Both	Age-standardized	Periodontal diseases	Rate	1997	1291.80	1486.65	1027.22
1657	Incidence	Republic of India	Male	Age-standardized	Periodontal diseases	Rate	1998	1294.71	1494.12	1025.58
1658	Incidence	Republic of India	Female	Age-standardized	Periodontal diseases	Rate	1998	1290.48	1474.72	1042.79
1659	Incidence	Republic of India	Both	Age-standardized	Periodontal diseases	Rate	1998	1292.66	1484.66	1031.91
1660	Incidence	Republic of India	Male	Age-standardized	Periodontal diseases	Rate	1999	1295.17	1491.19	1027.46
1661	Incidence	Republic of India	Female	Age-standardized	Periodontal diseases	Rate	1999	1291.69	1474.09	1045.55
1662	Incidence	Republic of India	Both	Age-standardized	Periodontal diseases	Rate	1999	1293.49	1482.73	1036.24
1663	Incidence	Republic of India	Male	Age-standardized	Periodontal diseases	Rate	2000	1296.10	1488.63	1033.32
1664	Incidence	Republic of India	Female	Age-standardized	Periodontal diseases	Rate	2000	1292.98	1473.46	1048.12
1665	Incidence	Republic of India	Both	Age-standardized	Periodontal diseases	Rate	2000	1294.59	1481.11	1040.53
1666	Incidence	Republic of India	Male	Age-standardized	Periodontal diseases	Rate	2001	1296.97	1488.51	1036.93

1667	Incidence	Republic of India	Female	Age-standardized	Periodontal diseases	Rate	2001	1294.22	1474.06	1052.70
1668	Incidence	Republic of India	Both	Age-standardized	Periodontal diseases	Rate	2001	1295.64	1481.66	1044.60
1669	Incidence	Republic of India	Male	Age-standardized	Periodontal diseases	Rate	2002	1297.21	1487.37	1039.99
1670	Incidence	Republic of India	Female	Age-standardized	Periodontal diseases	Rate	2002	1295.22	1474.23	1056.81
1671	Incidence	Republic of India	Both	Age-standardized	Periodontal diseases	Rate	2002	1296.24	1481.01	1048.16
1672	Incidence	Republic of India	Male	Age-standardized	Periodontal diseases	Rate	2003	1297.22	1482.82	1042.73
1673	Incidence	Republic of India	Female	Age-standardized	Periodontal diseases	Rate	2003	1296.13	1474.78	1060.54
1674	Incidence	Republic of India	Both	Age-standardized	Periodontal diseases	Rate	2003	1296.69	1478.64	1051.37
1675	Incidence	Republic of India	Male	Age-standardized	Periodontal diseases	Rate	2004	1297.40	1477.97	1045.53
1676	Incidence	Republic of India	Female	Age-standardized	Periodontal diseases	Rate	2004	1297.10	1474.30	1063.99
1677	Incidence	Republic of India	Both	Age-standardized	Periodontal diseases	Rate	2004	1297.26	1476.32	1054.53
1678	Incidence	Republic of India	Male	Age-standardized	Periodontal diseases	Rate	2005	1298.17	1473.92	1048.89
1679	Incidence	Republic of India	Female	Age-standardized	Periodontal diseases	Rate	2005	1298.24	1474.42	1065.80
1680	Incidence	Republic of India	Both	Age-standardized	Periodontal diseases	Rate	2005	1298.21	1475.03	1058.00
1681	Incidence	Republic of India	Male	Age-standardized	Periodontal diseases	Rate	2006	1299.31	1475.07	1050.93
1682	Incidence	Republic of India	Female	Age-standardized	Periodontal diseases	Rate	2006	1298.98	1473.97	1070.14
1683	Incidence	Republic of India	Both	Age-standardized	Periodontal diseases	Rate	2006	1299.17	1474.87	1062.32
1684	Incidence	Republic of India	Male	Age-standardized	Periodontal diseases	Rate	2007	1300.43	1476.66	1052.72
1685	Incidence	Republic of India	Female	Age-standardized	Periodontal diseases	Rate	2007	1299.05	1470.84	1073.74
1686	Incidence	Republic of India	Both	Age-standardized	Periodontal diseases	Rate	2007	1299.79	1473.91	1064.94
1687	Incidence	Republic of India	Male	Age-standardized	Periodontal diseases	Rate	2008	1301.63	1478.71	1054.25
1688	Incidence	Republic of India	Female	Age-standardized	Periodontal diseases	Rate	2008	1298.86	1468.08	1075.86

1689	Incidence	Republic of India	Both	Age-standardized	Periodontal diseases	Rate	2008	1300.33	1472.78	1067.26
1690	Incidence	Republic of India	Male	Age-standardized	Periodontal diseases	Rate	2009	1302.97	1481.23	1055.57
1691	Incidence	Republic of India	Female	Age-standardized	Periodontal diseases	Rate	2009	1298.82	1465.80	1077.91
1692	Incidence	Republic of India	Both	Age-standardized	Periodontal diseases	Rate	2009	1301.01	1472.67	1069.40
1693	Incidence	Republic of India	Male	Age-standardized	Periodontal diseases	Rate	2010	1304.48	1481.83	1056.77
1694	Incidence	Republic of India	Female	Age-standardized	Periodontal diseases	Rate	2010	1299.33	1463.89	1080.23
1695	Incidence	Republic of India	Both	Age-standardized	Periodontal diseases	Rate	2010	1302.04	1473.58	1071.60
1696	Incidence	Republic of India	Male	Age-standardized	Periodontal diseases	Rate	2011	1307.28	1480.98	1072.16
1697	Incidence	Republic of India	Female	Age-standardized	Periodontal diseases	Rate	2011	1299.05	1452.18	1099.86
1698	Incidence	Republic of India	Both	Age-standardized	Periodontal diseases	Rate	2011	1303.36	1467.26	1084.28
1699	Incidence	Republic of India	Male	Age-standardized	Periodontal diseases	Rate	2012	1311.57	1486.83	1085.28
1700	Incidence	Republic of India	Female	Age-standardized	Periodontal diseases	Rate	2012	1296.94	1444.91	1113.65
1701	Incidence	Republic of India	Both	Age-standardized	Periodontal diseases	Rate	2012	1304.55	1462.88	1102.16
1702	Incidence	Republic of India	Male	Age-standardized	Periodontal diseases	Rate	2013	1315.97	1488.85	1095.31
1703	Incidence	Republic of India	Female	Age-standardized	Periodontal diseases	Rate	2013	1293.86	1440.47	1121.52
1704	Incidence	Republic of India	Both	Age-standardized	Periodontal diseases	Rate	2013	1305.34	1461.01	1115.22
1705	Incidence	Republic of India	Male	Age-standardized	Periodontal diseases	Rate	2014	1319.14	1488.85	1109.46
1706	Incidence	Republic of India	Female	Age-standardized	Periodontal diseases	Rate	2014	1290.74	1440.85	1132.30
1707	Incidence	Republic of India	Both	Age-standardized	Periodontal diseases	Rate	2014	1305.46	1458.61	1124.41
1708	Incidence	Republic of India	Male	Age-standardized	Periodontal diseases	Rate	2015	1319.69	1487.60	1122.32
1709	Incidence	Republic of India	Female	Age-standardized	Periodontal diseases	Rate	2015	1288.53	1446.79	1130.33
1710	Incidence	Republic of India	Both	Age-standardized	Periodontal diseases	Rate	2015	1304.67	1457.32	1129.71

1711	Incidence	Republic of India	Male	Age-standardized	Periodontal diseases	Rate	2016	1312.76	1472.52	1129.24
1712	Incidence	Republic of India	Female	Age-standardized	Periodontal diseases	Rate	2016	1286.55	1440.19	1130.27
1713	Incidence	Republic of India	Both	Age-standardized	Periodontal diseases	Rate	2016	1300.12	1450.17	1137.95
1714	Incidence	Republic of India	Male	Age-standardized	Periodontal diseases	Rate	2017	1299.05	1456.13	1130.81
1715	Incidence	Republic of India	Female	Age-standardized	Periodontal diseases	Rate	2017	1284.17	1435.38	1130.46
1716	Incidence	Republic of India	Both	Age-standardized	Periodontal diseases	Rate	2017	1291.89	1440.69	1136.13
1717	Incidence	Republic of India	Male	Age-standardized	Periodontal diseases	Rate	2018	1285.53	1436.18	1127.83
1718	Incidence	Republic of India	Female	Age-standardized	Periodontal diseases	Rate	2018	1282.50	1432.82	1131.56
1719	Incidence	Republic of India	Both	Age-standardized	Periodontal diseases	Rate	2018	1284.10	1434.62	1129.36
1720	Incidence	Republic of India	Male	Age-standardized	Periodontal diseases	Rate	2019	1279.21	1430.93	1121.92
1721	Incidence	Republic of India	Female	Age-standardized	Periodontal diseases	Rate	2019	1282.63	1433.87	1134.46
1722	Incidence	Republic of India	Both	Age-standardized	Periodontal diseases	Rate	2019	1280.91	1432.38	1127.45
1723	Incidence	Republic of India	Male	Age-standardized	Periodontal diseases	Rate	2020	1279.35	1435.11	1130.42
1724	Incidence	Republic of India	Female	Age-standardized	Periodontal diseases	Rate	2020	1282.98	1433.80	1134.97
1725	Incidence	Republic of India	Both	Age-standardized	Periodontal diseases	Rate	2020	1281.12	1434.60	1133.01
1726	Incidence	Republic of India	Male	Age-standardized	Periodontal diseases	Rate	2021	1278.25	1434.35	1128.62
1727	Incidence	Republic of India	Female	Age-standardized	Periodontal diseases	Rate	2021	1282.06	1432.46	1134.14
1728	Incidence	Republic of India	Both	Age-standardized	Periodontal diseases	Rate	2021	1280.14	1433.88	1130.83
1729	Incidence	Republic of India	Male	Age-standardized	Periodontal diseases	Rate	2022	1276.03	1426.15	1127.15
1730	Incidence	Republic of India	Female	Age-standardized	Periodontal diseases	Rate	2022	1282.72	1439.17	1135.65
1731	Incidence	Republic of India	Both	Age-standardized	Periodontal diseases	Rate	2022	1279.30	1432.29	1131.64
1732	Incidence	Republic of India	Male	Age-standardized	Periodontal diseases	Rate	2023	1249.36	1396.68	1103.30

1733	Incidence	Republic of India	Female	Age-standardized	Periodontal diseases	Rate	2023	1255.92	1409.18	1112.55
1734	Incidence	Republic of India	Both	Age-standardized	Periodontal diseases	Rate	2023	1252.56	1402.77	1107.95
1735	Incidence	Argentine Republic	Male	Age-standardized	Periodontal diseases	Rate	1990	1088.24	1307.24	844.58
1736	Incidence	Argentine Republic	Female	Age-standardized	Periodontal diseases	Rate	1990	1003.70	1220.31	751.02
1737	Incidence	Argentine Republic	Both	Age-standardized	Periodontal diseases	Rate	1990	1045.67	1267.28	796.92
1738	Incidence	Argentine Republic	Male	Age-standardized	Periodontal diseases	Rate	1991	1093.86	1311.06	849.94
1739	Incidence	Argentine Republic	Female	Age-standardized	Periodontal diseases	Rate	1991	1003.64	1186.34	798.43
1740	Incidence	Argentine Republic	Both	Age-standardized	Periodontal diseases	Rate	1991	1048.26	1252.92	816.43
1741	Incidence	Argentine Republic	Male	Age-standardized	Periodontal diseases	Rate	1992	1097.42	1317.77	848.14
1742	Incidence	Argentine Republic	Female	Age-standardized	Periodontal diseases	Rate	1992	1000.61	1155.63	829.29
1743	Incidence	Argentine Republic	Both	Age-standardized	Periodontal diseases	Rate	1992	1048.34	1226.53	844.24
1744	Incidence	Argentine Republic	Male	Age-standardized	Periodontal diseases	Rate	1993	1099.38	1320.85	842.88
1745	Incidence	Argentine Republic	Female	Age-standardized	Periodontal diseases	Rate	1993	995.41	1153.27	839.01
1746	Incidence	Argentine Republic	Both	Age-standardized	Periodontal diseases	Rate	1993	1046.54	1217.06	867.79
1747	Incidence	Argentine Republic	Male	Age-standardized	Periodontal diseases	Rate	1994	1100.20	1322.32	833.85
1748	Incidence	Argentine Republic	Female	Age-standardized	Periodontal diseases	Rate	1994	988.87	1168.76	825.80
1749	Incidence	Argentine Republic	Both	Age-standardized	Periodontal diseases	Rate	1994	1043.52	1193.45	869.88
1750	Incidence	Argentine Republic	Male	Age-standardized	Periodontal diseases	Rate	1995	1100.36	1323.41	831.12
1751	Incidence	Argentine Republic	Female	Age-standardized	Periodontal diseases	Rate	1995	981.78	1186.50	778.46
1752	Incidence	Argentine Republic	Both	Age-standardized	Periodontal diseases	Rate	1995	1039.91	1191.72	879.69
1753	Incidence	Argentine Republic	Male	Age-standardized	Periodontal diseases	Rate	1996	1083.59	1249.32	873.64
1754	Incidence	Argentine Republic	Female	Age-standardized	Periodontal diseases	Rate	1996	960.51	1119.92	798.29

1755	Incidence	Argentine Republic	Both	Age-standardized	Periodontal diseases	Rate	1996	1020.78	1144.90	897.20
1756	Incidence	Argentine Republic	Male	Age-standardized	Periodontal diseases	Rate	1997	1043.59	1163.50	898.65
1757	Incidence	Argentine Republic	Female	Age-standardized	Periodontal diseases	Rate	1997	920.04	1051.78	801.44
1758	Incidence	Argentine Republic	Both	Age-standardized	Periodontal diseases	Rate	1997	980.49	1091.15	874.84
1759	Incidence	Argentine Republic	Male	Age-standardized	Periodontal diseases	Rate	1998	995.79	1085.41	894.66
1760	Incidence	Argentine Republic	Female	Age-standardized	Periodontal diseases	Rate	1998	874.12	982.67	781.76
1761	Incidence	Argentine Republic	Both	Age-standardized	Periodontal diseases	Rate	1998	933.63	1025.07	854.69
1762	Incidence	Argentine Republic	Male	Age-standardized	Periodontal diseases	Rate	1999	955.60	1041.18	886.22
1763	Incidence	Argentine Republic	Female	Age-standardized	Periodontal diseases	Rate	1999	836.49	927.42	760.92
1764	Incidence	Argentine Republic	Both	Age-standardized	Periodontal diseases	Rate	1999	894.76	980.36	823.01
1765	Incidence	Argentine Republic	Male	Age-standardized	Periodontal diseases	Rate	2000	938.45	1026.04	863.74
1766	Incidence	Argentine Republic	Female	Age-standardized	Periodontal diseases	Rate	2000	820.91	907.03	750.90
1767	Incidence	Argentine Republic	Both	Age-standardized	Periodontal diseases	Rate	2000	878.43	964.08	807.45
1768	Incidence	Argentine Republic	Male	Age-standardized	Periodontal diseases	Rate	2001	951.96	1048.05	870.06
1769	Incidence	Argentine Republic	Female	Age-standardized	Periodontal diseases	Rate	2001	838.60	929.16	762.59
1770	Incidence	Argentine Republic	Both	Age-standardized	Periodontal diseases	Rate	2001	894.13	985.10	817.25
1771	Incidence	Argentine Republic	Male	Age-standardized	Periodontal diseases	Rate	2002	985.09	1092.18	885.90
1772	Incidence	Argentine Republic	Female	Age-standardized	Periodontal diseases	Rate	2002	881.02	991.79	787.16
1773	Incidence	Argentine Republic	Both	Age-standardized	Periodontal diseases	Rate	2002	932.09	1037.65	835.09
1774	Incidence	Argentine Republic	Male	Age-standardized	Periodontal diseases	Rate	2003	1025.28	1160.74	889.77
1775	Incidence	Argentine Republic	Female	Age-standardized	Periodontal diseases	Rate	2003	932.27	1067.06	806.74
1776	Incidence	Argentine Republic	Both	Age-standardized	Periodontal diseases	Rate	2003	978.04	1104.06	852.03

1777	Incidence	Argentine Republic	Male	Age-standardized	Periodontal diseases	Rate	2004	1059.98	1238.09	879.13
1778	Incidence	Argentine Republic	Female	Age-standardized	Periodontal diseases	Rate	2004	976.46	1154.30	819.88
1779	Incidence	Argentine Republic	Both	Age-standardized	Periodontal diseases	Rate	2004	1017.68	1185.24	856.32
1780	Incidence	Argentine Republic	Male	Age-standardized	Periodontal diseases	Rate	2005	1076.66	1297.91	859.51
1781	Incidence	Argentine Republic	Female	Age-standardized	Periodontal diseases	Rate	2005	997.67	1227.66	795.13
1782	Incidence	Argentine Republic	Both	Age-standardized	Periodontal diseases	Rate	2005	1036.71	1244.97	829.90
1783	Incidence	Argentine Republic	Male	Age-standardized	Periodontal diseases	Rate	2006	1080.10	1297.12	862.10
1784	Incidence	Argentine Republic	Female	Age-standardized	Periodontal diseases	Rate	2006	1001.69	1224.00	800.32
1785	Incidence	Argentine Republic	Both	Age-standardized	Periodontal diseases	Rate	2006	1040.45	1250.25	836.46
1786	Incidence	Argentine Republic	Male	Age-standardized	Periodontal diseases	Rate	2007	1083.41	1292.75	869.17
1787	Incidence	Argentine Republic	Female	Age-standardized	Periodontal diseases	Rate	2007	1004.98	1222.52	807.04
1788	Incidence	Argentine Republic	Both	Age-standardized	Periodontal diseases	Rate	2007	1043.74	1254.31	841.66
1789	Incidence	Argentine Republic	Male	Age-standardized	Periodontal diseases	Rate	2008	1086.44	1292.78	876.60
1790	Incidence	Argentine Republic	Female	Age-standardized	Periodontal diseases	Rate	2008	1007.65	1222.29	809.60
1791	Incidence	Argentine Republic	Both	Age-standardized	Periodontal diseases	Rate	2008	1046.56	1255.88	844.64
1792	Incidence	Argentine Republic	Male	Age-standardized	Periodontal diseases	Rate	2009	1089.07	1294.91	882.28
1793	Incidence	Argentine Republic	Female	Age-standardized	Periodontal diseases	Rate	2009	1009.83	1222.09	809.50
1794	Incidence	Argentine Republic	Both	Age-standardized	Periodontal diseases	Rate	2009	1048.93	1253.79	849.46
1795	Incidence	Argentine Republic	Male	Age-standardized	Periodontal diseases	Rate	2010	1091.16	1297.64	887.42
1796	Incidence	Argentine Republic	Female	Age-standardized	Periodontal diseases	Rate	2010	1011.66	1230.59	812.15
1797	Incidence	Argentine Republic	Both	Age-standardized	Periodontal diseases	Rate	2010	1050.87	1259.30	848.58
1798	Incidence	Argentine Republic	Male	Age-standardized	Periodontal diseases	Rate	2011	1093.13	1302.08	888.64

1799	Incidence	Argentine Republic	Female	Age-standardized	Periodontal diseases	Rate	2011	1013.46	1227.35	813.71
1800	Incidence	Argentine Republic	Both	Age-standardized	Periodontal diseases	Rate	2011	1052.74	1255.94	852.00
1801	Incidence	Argentine Republic	Male	Age-standardized	Periodontal diseases	Rate	2012	1095.30	1309.81	891.90
1802	Incidence	Argentine Republic	Female	Age-standardized	Periodontal diseases	Rate	2012	1015.37	1226.11	814.13
1803	Incidence	Argentine Republic	Both	Age-standardized	Periodontal diseases	Rate	2012	1054.78	1260.32	855.09
1804	Incidence	Argentine Republic	Male	Age-standardized	Periodontal diseases	Rate	2013	1097.38	1318.28	895.97
1805	Incidence	Argentine Republic	Female	Age-standardized	Periodontal diseases	Rate	2013	1017.25	1231.16	817.16
1806	Incidence	Argentine Republic	Both	Age-standardized	Periodontal diseases	Rate	2013	1056.74	1264.52	857.82
1807	Incidence	Argentine Republic	Male	Age-standardized	Periodontal diseases	Rate	2014	1099.06	1325.65	898.85
1808	Incidence	Argentine Republic	Female	Age-standardized	Periodontal diseases	Rate	2014	1018.93	1231.68	818.03
1809	Incidence	Argentine Republic	Both	Age-standardized	Periodontal diseases	Rate	2014	1058.41	1270.58	859.96
1810	Incidence	Argentine Republic	Male	Age-standardized	Periodontal diseases	Rate	2015	1100.06	1331.79	902.16
1811	Incidence	Argentine Republic	Female	Age-standardized	Periodontal diseases	Rate	2015	1020.27	1232.37	817.81
1812	Incidence	Argentine Republic	Both	Age-standardized	Periodontal diseases	Rate	2015	1059.56	1279.79	861.98
1813	Incidence	Argentine Republic	Male	Age-standardized	Periodontal diseases	Rate	2016	1100.25	1327.67	901.83
1814	Incidence	Argentine Republic	Female	Age-standardized	Periodontal diseases	Rate	2016	1021.93	1234.21	826.27
1815	Incidence	Argentine Republic	Both	Age-standardized	Periodontal diseases	Rate	2016	1060.48	1275.97	863.67
1816	Incidence	Argentine Republic	Male	Age-standardized	Periodontal diseases	Rate	2017	1099.89	1327.78	903.81
1817	Incidence	Argentine Republic	Female	Age-standardized	Periodontal diseases	Rate	2017	1024.11	1242.78	832.52
1818	Incidence	Argentine Republic	Both	Age-standardized	Periodontal diseases	Rate	2017	1061.41	1272.77	866.72
1819	Incidence	Argentine Republic	Male	Age-standardized	Periodontal diseases	Rate	2018	1099.29	1326.82	905.81
1820	Incidence	Argentine Republic	Female	Age-standardized	Periodontal diseases	Rate	2018	1025.97	1237.96	832.45

1821	Incidence	Argentine Republic	Both	Age-standardized	Periodontal diseases	Rate	2018	1062.07	1278.38	867.86
1822	Incidence	Argentine Republic	Male	Age-standardized	Periodontal diseases	Rate	2019	1098.75	1324.73	907.27
1823	Incidence	Argentine Republic	Female	Age-standardized	Periodontal diseases	Rate	2019	1026.68	1239.93	827.54
1824	Incidence	Argentine Republic	Both	Age-standardized	Periodontal diseases	Rate	2019	1062.18	1273.37	868.62
1825	Incidence	Argentine Republic	Male	Age-standardized	Periodontal diseases	Rate	2020	1095.52	1303.85	912.58
1826	Incidence	Argentine Republic	Female	Age-standardized	Periodontal diseases	Rate	2020	1019.41	1247.19	808.01
1827	Incidence	Argentine Republic	Both	Age-standardized	Periodontal diseases	Rate	2020	1056.73	1275.26	860.90
1828	Incidence	Argentine Republic	Male	Age-standardized	Periodontal diseases	Rate	2021	1088.58	1319.18	879.45
1829	Incidence	Argentine Republic	Female	Age-standardized	Periodontal diseases	Rate	2021	1021.75	1244.88	821.33
1830	Incidence	Argentine Republic	Both	Age-standardized	Periodontal diseases	Rate	2021	1054.66	1277.28	852.91
1831	Incidence	Argentine Republic	Male	Age-standardized	Periodontal diseases	Rate	2022	1091.91	1299.50	874.46
1832	Incidence	Argentine Republic	Female	Age-standardized	Periodontal diseases	Rate	2022	1015.66	1250.44	819.72
1833	Incidence	Argentine Republic	Both	Age-standardized	Periodontal diseases	Rate	2022	1053.19	1268.51	850.24
1834	Incidence	Argentine Republic	Male	Age-standardized	Periodontal diseases	Rate	2023	1079.59	1285.41	864.70
1835	Incidence	Argentine Republic	Female	Age-standardized	Periodontal diseases	Rate	2023	1004.18	1236.10	810.09
1836	Incidence	Argentine Republic	Both	Age-standardized	Periodontal diseases	Rate	2023	1041.31	1255.04	840.59
1837	Incidence	Republic of South Africa	Male	Age-standardized	Periodontal diseases	Rate	1990	818.91	1024.46	597.71
1838	Incidence	Republic of South Africa	Female	Age-standardized	Periodontal diseases	Rate	1990	801.09	993.99	583.16
1839	Incidence	Republic of South Africa	Both	Age-standardized	Periodontal diseases	Rate	1990	809.62	1007.46	589.98
1840	Incidence	Republic of South Africa	Male	Age-standardized	Periodontal diseases	Rate	1991	815.52	1020.98	595.02
1841	Incidence	Republic of South Africa	Female	Age-standardized	Periodontal diseases	Rate	1991	795.77	985.92	583.75
1842	Incidence	Republic of South Africa	Both	Age-standardized	Periodontal diseases	Rate	1991	805.20	1002.17	589.23

1843	Incidence	Republic of South Africa	Male	Age-standardized	Periodontal diseases	Rate	1992	812.21	1016.53	593.05
1844	Incidence	Republic of South Africa	Female	Age-standardized	Periodontal diseases	Rate	1992	790.59	978.57	584.20
1845	Incidence	Republic of South Africa	Both	Age-standardized	Periodontal diseases	Rate	1992	800.89	997.31	588.20
1846	Incidence	Republic of South Africa	Male	Age-standardized	Periodontal diseases	Rate	1993	809.15	1011.06	590.87
1847	Incidence	Republic of South Africa	Female	Age-standardized	Periodontal diseases	Rate	1993	786.06	972.37	584.53
1848	Incidence	Republic of South Africa	Both	Age-standardized	Periodontal diseases	Rate	1993	797.02	991.21	587.25
1849	Incidence	Republic of South Africa	Male	Age-standardized	Periodontal diseases	Rate	1994	806.47	1006.98	588.96
1850	Incidence	Republic of South Africa	Female	Age-standardized	Periodontal diseases	Rate	1994	782.64	965.86	585.37
1851	Incidence	Republic of South Africa	Both	Age-standardized	Periodontal diseases	Rate	1994	793.93	984.83	586.49
1852	Incidence	Republic of South Africa	Male	Age-standardized	Periodontal diseases	Rate	1995	804.34	1003.85	587.39
1853	Incidence	Republic of South Africa	Female	Age-standardized	Periodontal diseases	Rate	1995	780.81	958.96	587.36
1854	Incidence	Republic of South Africa	Both	Age-standardized	Periodontal diseases	Rate	1995	791.93	980.07	586.51
1855	Incidence	Republic of South Africa	Male	Age-standardized	Periodontal diseases	Rate	1996	796.13	987.26	593.28
1856	Incidence	Republic of South Africa	Female	Age-standardized	Periodontal diseases	Rate	1996	769.07	935.75	587.95
1857	Incidence	Republic of South Africa	Both	Age-standardized	Periodontal diseases	Rate	1996	781.83	957.42	593.18
1858	Incidence	Republic of South Africa	Male	Age-standardized	Periodontal diseases	Rate	1997	779.33	955.92	588.36
1859	Incidence	Republic of South Africa	Female	Age-standardized	Periodontal diseases	Rate	1997	742.11	892.05	578.94
1860	Incidence	Republic of South Africa	Both	Age-standardized	Periodontal diseases	Rate	1997	759.60	921.02	581.61
1861	Incidence	Republic of South Africa	Male	Age-standardized	Periodontal diseases	Rate	1998	760.11	923.22	574.49
1862	Incidence	Republic of South Africa	Female	Age-standardized	Periodontal diseases	Rate	1998	709.82	842.84	558.33
1863	Incidence	Republic of South Africa	Both	Age-standardized	Periodontal diseases	Rate	1998	733.37	881.20	568.52
1864	Incidence	Republic of South Africa	Male	Age-standardized	Periodontal diseases	Rate	1999	744.39	900.57	569.45

1865	Incidence	Republic of South Africa	Female	Age-standardized	Periodontal diseases	Rate	1999	682.40	804.56	538.94
1866	Incidence	Republic of South Africa	Both	Age-standardized	Periodontal diseases	Rate	1999	711.35	846.09	554.56
1867	Incidence	Republic of South Africa	Male	Age-standardized	Periodontal diseases	Rate	2000	737.85	884.54	571.20
1868	Incidence	Republic of South Africa	Female	Age-standardized	Periodontal diseases	Rate	2000	670.41	792.42	531.52
1869	Incidence	Republic of South Africa	Both	Age-standardized	Periodontal diseases	Rate	2000	701.83	828.42	551.30
1870	Incidence	Republic of South Africa	Male	Age-standardized	Periodontal diseases	Rate	2001	725.27	853.01	573.28
1871	Incidence	Republic of South Africa	Female	Age-standardized	Periodontal diseases	Rate	2001	661.33	783.12	533.47
1872	Incidence	Republic of South Africa	Both	Age-standardized	Periodontal diseases	Rate	2001	691.08	814.38	552.10
1873	Incidence	Republic of South Africa	Male	Age-standardized	Periodontal diseases	Rate	2002	695.41	817.07	558.13
1874	Incidence	Republic of South Africa	Female	Age-standardized	Periodontal diseases	Rate	2002	640.83	764.64	524.85
1875	Incidence	Republic of South Africa	Both	Age-standardized	Periodontal diseases	Rate	2002	666.24	787.34	540.10
1876	Incidence	Republic of South Africa	Male	Age-standardized	Periodontal diseases	Rate	2003	659.80	781.92	534.93
1877	Incidence	Republic of South Africa	Female	Age-standardized	Periodontal diseases	Rate	2003	616.92	744.86	512.05
1878	Incidence	Republic of South Africa	Both	Age-standardized	Periodontal diseases	Rate	2003	636.95	759.61	523.23
1879	Incidence	Republic of South Africa	Male	Age-standardized	Periodontal diseases	Rate	2004	630.14	753.57	516.27
1880	Incidence	Republic of South Africa	Female	Age-standardized	Periodontal diseases	Rate	2004	597.48	731.19	493.68
1881	Incidence	Republic of South Africa	Both	Age-standardized	Periodontal diseases	Rate	2004	612.82	737.40	506.23
1882	Incidence	Republic of South Africa	Male	Age-standardized	Periodontal diseases	Rate	2005	618.35	746.48	513.47
1883	Incidence	Republic of South Africa	Female	Age-standardized	Periodontal diseases	Rate	2005	590.29	734.21	484.46
1884	Incidence	Republic of South Africa	Both	Age-standardized	Periodontal diseases	Rate	2005	603.53	736.79	502.81
1885	Incidence	Republic of South Africa	Male	Age-standardized	Periodontal diseases	Rate	2006	616.90	746.30	512.31
1886	Incidence	Republic of South Africa	Female	Age-standardized	Periodontal diseases	Rate	2006	592.47	736.98	486.26

1887	Incidence	Republic of South Africa	Both	Age-standardized	Periodontal diseases	Rate	2006	604.05	737.77	502.49
1888	Incidence	Republic of South Africa	Male	Age-standardized	Periodontal diseases	Rate	2007	611.61	744.60	509.67
1889	Incidence	Republic of South Africa	Female	Age-standardized	Periodontal diseases	Rate	2007	595.50	740.35	488.70
1890	Incidence	Republic of South Africa	Both	Age-standardized	Periodontal diseases	Rate	2007	603.25	737.83	499.24
1891	Incidence	Republic of South Africa	Male	Age-standardized	Periodontal diseases	Rate	2008	604.76	738.76	503.95
1892	Incidence	Republic of South Africa	Female	Age-standardized	Periodontal diseases	Rate	2008	598.77	744.01	491.16
1893	Incidence	Republic of South Africa	Both	Age-standardized	Periodontal diseases	Rate	2008	601.82	737.88	497.11
1894	Incidence	Republic of South Africa	Male	Age-standardized	Periodontal diseases	Rate	2009	598.86	734.04	493.56
1895	Incidence	Republic of South Africa	Female	Age-standardized	Periodontal diseases	Rate	2009	601.64	747.53	493.41
1896	Incidence	Republic of South Africa	Both	Age-standardized	Periodontal diseases	Rate	2009	600.62	738.94	495.34
1897	Incidence	Republic of South Africa	Male	Age-standardized	Periodontal diseases	Rate	2010	596.60	733.98	487.98
1898	Incidence	Republic of South Africa	Female	Age-standardized	Periodontal diseases	Rate	2010	603.52	749.99	494.75
1899	Incidence	Republic of South Africa	Both	Age-standardized	Periodontal diseases	Rate	2010	600.56	741.73	494.47
1900	Incidence	Republic of South Africa	Male	Age-standardized	Periodontal diseases	Rate	2011	597.20	736.34	489.71
1901	Incidence	Republic of South Africa	Female	Age-standardized	Periodontal diseases	Rate	2011	604.82	750.85	495.32
1902	Incidence	Republic of South Africa	Both	Age-standardized	Periodontal diseases	Rate	2011	601.53	742.82	494.84
1903	Incidence	Republic of South Africa	Male	Age-standardized	Periodontal diseases	Rate	2012	597.91	739.33	491.41
1904	Incidence	Republic of South Africa	Female	Age-standardized	Periodontal diseases	Rate	2012	606.22	752.21	495.62
1905	Incidence	Republic of South Africa	Both	Age-standardized	Periodontal diseases	Rate	2012	602.60	743.85	495.33
1906	Incidence	Republic of South Africa	Male	Age-standardized	Periodontal diseases	Rate	2013	598.61	742.90	492.72
1907	Incidence	Republic of South Africa	Female	Age-standardized	Periodontal diseases	Rate	2013	607.57	753.49	495.99
1908	Incidence	Republic of South Africa	Both	Age-standardized	Periodontal diseases	Rate	2013	603.64	744.83	495.74

1909	Incidence	Republic of South Africa	Male	Age-standardized	Periodontal diseases	Rate	2014	599.17	746.42	493.73
1910	Incidence	Republic of South Africa	Female	Age-standardized	Periodontal diseases	Rate	2014	608.66	754.62	496.42
1911	Incidence	Republic of South Africa	Both	Age-standardized	Periodontal diseases	Rate	2014	604.48	746.10	495.89
1912	Incidence	Republic of South Africa	Male	Age-standardized	Periodontal diseases	Rate	2015	599.45	747.51	493.77
1913	Incidence	Republic of South Africa	Female	Age-standardized	Periodontal diseases	Rate	2015	609.34	755.94	495.88
1914	Incidence	Republic of South Africa	Both	Age-standardized	Periodontal diseases	Rate	2015	604.97	748.10	495.86
1915	Incidence	Republic of South Africa	Male	Age-standardized	Periodontal diseases	Rate	2016	599.84	746.36	494.66
1916	Incidence	Republic of South Africa	Female	Age-standardized	Periodontal diseases	Rate	2016	609.99	755.37	497.99
1917	Incidence	Republic of South Africa	Both	Age-standardized	Periodontal diseases	Rate	2016	605.50	747.24	496.69
1918	Incidence	Republic of South Africa	Male	Age-standardized	Periodontal diseases	Rate	2017	600.58	745.25	493.25
1919	Incidence	Republic of South Africa	Female	Age-standardized	Periodontal diseases	Rate	2017	610.88	755.31	500.98
1920	Incidence	Republic of South Africa	Both	Age-standardized	Periodontal diseases	Rate	2017	606.34	748.96	497.77
1921	Incidence	Republic of South Africa	Male	Age-standardized	Periodontal diseases	Rate	2018	601.27	746.30	491.74
1922	Incidence	Republic of South Africa	Female	Age-standardized	Periodontal diseases	Rate	2018	611.65	755.65	503.77
1923	Incidence	Republic of South Africa	Both	Age-standardized	Periodontal diseases	Rate	2018	607.08	750.81	498.81
1924	Incidence	Republic of South Africa	Male	Age-standardized	Periodontal diseases	Rate	2019	601.49	748.08	490.42
1925	Incidence	Republic of South Africa	Female	Age-standardized	Periodontal diseases	Rate	2019	611.92	755.65	505.38
1926	Incidence	Republic of South Africa	Both	Age-standardized	Periodontal diseases	Rate	2019	607.34	751.83	499.39
1927	Incidence	Republic of South Africa	Male	Age-standardized	Periodontal diseases	Rate	2020	599.49	741.12	491.13
1928	Incidence	Republic of South Africa	Female	Age-standardized	Periodontal diseases	Rate	2020	606.84	752.34	492.80
1929	Incidence	Republic of South Africa	Both	Age-standardized	Periodontal diseases	Rate	2020	603.67	745.45	492.65
1930	Incidence	Republic of South Africa	Male	Age-standardized	Periodontal diseases	Rate	2021	596.92	741.29	492.32

1931	Incidence	Republic of South Africa	Female	Age-standardized	Periodontal diseases	Rate	2021	606.52	752.36	497.33
1932	Incidence	Republic of South Africa	Both	Age-standardized	Periodontal diseases	Rate	2021	602.16	745.48	495.71
1933	Incidence	Republic of South Africa	Male	Age-standardized	Periodontal diseases	Rate	2022	575.86	738.36	462.49
1934	Incidence	Republic of South Africa	Female	Age-standardized	Periodontal diseases	Rate	2022	578.63	738.20	458.09
1935	Incidence	Republic of South Africa	Both	Age-standardized	Periodontal diseases	Rate	2022	577.67	737.52	462.09
1936	Incidence	Republic of South Africa	Male	Age-standardized	Periodontal diseases	Rate	2023	570.77	732.32	458.09
1937	Incidence	Republic of South Africa	Female	Age-standardized	Periodontal diseases	Rate	2023	573.71	732.41	453.73
1938	Incidence	Republic of South Africa	Both	Age-standardized	Periodontal diseases	Rate	2023	572.66	731.48	457.83
1939	Incidence	European Union	Male	Age-standardized	Periodontal diseases	Rate	1990	989.07	1151.69	793.66
1940	Incidence	European Union	Female	Age-standardized	Periodontal diseases	Rate	1990	963.27	1102.51	790.71
1941	Incidence	European Union	Both	Age-standardized	Periodontal diseases	Rate	1990	977.34	1122.71	795.86
1942	Incidence	European Union	Male	Age-standardized	Periodontal diseases	Rate	1991	994.80	1149.50	809.81
1943	Incidence	European Union	Female	Age-standardized	Periodontal diseases	Rate	1991	947.79	1068.49	794.02
1944	Incidence	European Union	Both	Age-standardized	Periodontal diseases	Rate	1991	972.05	1104.97	803.68
1945	Incidence	European Union	Male	Age-standardized	Periodontal diseases	Rate	1992	1000.52	1147.45	824.62
1946	Incidence	European Union	Female	Age-standardized	Periodontal diseases	Rate	1992	934.72	1045.60	798.34
1947	Incidence	European Union	Both	Age-standardized	Periodontal diseases	Rate	1992	968.00	1089.45	811.23
1948	Incidence	European Union	Male	Age-standardized	Periodontal diseases	Rate	1993	1005.25	1144.61	832.37
1949	Incidence	European Union	Female	Age-standardized	Periodontal diseases	Rate	1993	924.32	1029.80	801.72
1950	Incidence	European Union	Both	Age-standardized	Periodontal diseases	Rate	1993	964.86	1080.20	817.40
1951	Incidence	European Union	Male	Age-standardized	Periodontal diseases	Rate	1994	1008.24	1140.98	837.97
1952	Incidence	European Union	Female	Age-standardized	Periodontal diseases	Rate	1994	917.09	1020.57	804.98

1953	Incidence	European Union	Both	Age-standardized	Periodontal diseases	Rate	1994	962.52	1068.05	823.10
1954	Incidence	European Union	Male	Age-standardized	Periodontal diseases	Rate	1995	1009.07	1135.11	841.98
1955	Incidence	European Union	Female	Age-standardized	Periodontal diseases	Rate	1995	913.86	1027.51	806.84
1956	Incidence	European Union	Both	Age-standardized	Periodontal diseases	Rate	1995	961.23	1063.94	828.01
1957	Incidence	European Union	Male	Age-standardized	Periodontal diseases	Rate	1996	1001.58	1112.38	855.22
1958	Incidence	European Union	Female	Age-standardized	Periodontal diseases	Rate	1996	914.53	1027.13	808.74
1959	Incidence	European Union	Both	Age-standardized	Periodontal diseases	Rate	1996	957.90	1060.60	835.81
1960	Incidence	European Union	Male	Age-standardized	Periodontal diseases	Rate	1997	984.12	1088.42	854.03
1961	Incidence	European Union	Female	Age-standardized	Periodontal diseases	Rate	1997	917.47	1024.98	816.14
1962	Incidence	European Union	Both	Age-standardized	Periodontal diseases	Rate	1997	950.85	1055.83	840.23
1963	Incidence	European Union	Male	Age-standardized	Periodontal diseases	Rate	1998	962.65	1065.67	847.78
1964	Incidence	European Union	Female	Age-standardized	Periodontal diseases	Rate	1998	921.03	1030.38	821.99
1965	Incidence	European Union	Both	Age-standardized	Periodontal diseases	Rate	1998	942.14	1050.42	835.19
1966	Incidence	European Union	Male	Age-standardized	Periodontal diseases	Rate	1999	943.46	1047.24	834.84
1967	Incidence	European Union	Female	Age-standardized	Periodontal diseases	Rate	1999	923.76	1039.68	820.53
1968	Incidence	European Union	Both	Age-standardized	Periodontal diseases	Rate	1999	934.12	1038.34	828.97
1969	Incidence	European Union	Male	Age-standardized	Periodontal diseases	Rate	2000	933.35	1040.65	825.24
1970	Incidence	European Union	Female	Age-standardized	Periodontal diseases	Rate	2000	924.26	1043.12	820.60
1971	Incidence	European Union	Both	Age-standardized	Periodontal diseases	Rate	2000	929.37	1035.27	829.62
1972	Incidence	European Union	Male	Age-standardized	Periodontal diseases	Rate	2001	924.19	1029.26	819.17
1973	Incidence	European Union	Female	Age-standardized	Periodontal diseases	Rate	2001	915.67	1033.46	811.25
1974	Incidence	European Union	Both	Age-standardized	Periodontal diseases	Rate	2001	920.48	1027.38	821.50

1975	Incidence	European Union	Male	Age-standardized	Periodontal diseases	Rate	2002	907.75	1014.36	807.56
1976	Incidence	European Union	Female	Age-standardized	Periodontal diseases	Rate	2002	897.38	1013.97	795.41
1977	Incidence	European Union	Both	Age-standardized	Periodontal diseases	Rate	2002	903.12	1014.20	803.81
1978	Incidence	European Union	Male	Age-standardized	Periodontal diseases	Rate	2003	889.56	998.32	794.93
1979	Incidence	European Union	Female	Age-standardized	Periodontal diseases	Rate	2003	876.28	993.18	774.32
1980	Incidence	European Union	Both	Age-standardized	Periodontal diseases	Rate	2003	883.48	996.38	786.29
1981	Incidence	European Union	Male	Age-standardized	Periodontal diseases	Rate	2004	874.79	985.71	780.62
1982	Incidence	European Union	Female	Age-standardized	Periodontal diseases	Rate	2004	858.98	977.82	756.63
1983	Incidence	European Union	Both	Age-standardized	Periodontal diseases	Rate	2004	867.44	981.69	769.49
1984	Incidence	European Union	Male	Age-standardized	Periodontal diseases	Rate	2005	868.53	983.99	772.13
1985	Incidence	European Union	Female	Age-standardized	Periodontal diseases	Rate	2005	851.99	970.55	746.91
1986	Incidence	European Union	Both	Age-standardized	Periodontal diseases	Rate	2005	860.79	979.29	759.08
1987	Incidence	European Union	Male	Age-standardized	Periodontal diseases	Rate	2006	872.74	991.69	773.11
1988	Incidence	European Union	Female	Age-standardized	Periodontal diseases	Rate	2006	858.19	980.03	752.81
1989	Incidence	European Union	Both	Age-standardized	Periodontal diseases	Rate	2006	865.97	987.82	763.16
1990	Incidence	European Union	Male	Age-standardized	Periodontal diseases	Rate	2007	883.37	1007.76	779.99
1991	Incidence	European Union	Female	Age-standardized	Periodontal diseases	Rate	2007	872.54	999.27	764.79
1992	Incidence	European Union	Both	Age-standardized	Periodontal diseases	Rate	2007	878.42	1005.24	774.65
1993	Incidence	European Union	Male	Age-standardized	Periodontal diseases	Rate	2008	896.08	1028.15	792.53
1994	Incidence	European Union	Female	Age-standardized	Periodontal diseases	Rate	2008	889.52	1020.13	778.52
1995	Incidence	European Union	Both	Age-standardized	Periodontal diseases	Rate	2008	893.23	1025.95	786.07
1996	Incidence	European Union	Male	Age-standardized	Periodontal diseases	Rate	2009	906.79	1045.67	798.54

1997	Incidence	European Union	Female	Age-standardized	Periodontal diseases	Rate	2009	903.80	1036.85	790.01
1998	Incidence	European Union	Both	Age-standardized	Periodontal diseases	Rate	2009	905.69	1042.84	794.40
1999	Incidence	European Union	Male	Age-standardized	Periodontal diseases	Rate	2010	911.67	1055.34	797.11
2000	Incidence	European Union	Female	Age-standardized	Periodontal diseases	Rate	2010	910.45	1046.72	792.67
2001	Incidence	European Union	Both	Age-standardized	Periodontal diseases	Rate	2010	911.44	1050.96	794.00
2002	Incidence	European Union	Male	Age-standardized	Periodontal diseases	Rate	2011	911.11	1057.07	795.39
2003	Incidence	European Union	Female	Age-standardized	Periodontal diseases	Rate	2011	910.55	1049.34	792.22
2004	Incidence	European Union	Both	Age-standardized	Periodontal diseases	Rate	2011	911.21	1053.87	792.54
2005	Incidence	European Union	Male	Age-standardized	Periodontal diseases	Rate	2012	909.25	1058.04	791.56
2006	Incidence	European Union	Female	Age-standardized	Periodontal diseases	Rate	2012	909.41	1050.96	789.09
2007	Incidence	European Union	Both	Age-standardized	Periodontal diseases	Rate	2012	909.73	1056.12	788.97
2008	Incidence	European Union	Male	Age-standardized	Periodontal diseases	Rate	2013	907.13	1056.49	787.18
2009	Incidence	European Union	Female	Age-standardized	Periodontal diseases	Rate	2013	907.89	1052.31	784.84
2010	Incidence	European Union	Both	Age-standardized	Periodontal diseases	Rate	2013	907.92	1056.63	785.59
2011	Incidence	European Union	Male	Age-standardized	Periodontal diseases	Rate	2014	905.63	1056.91	783.38
2012	Incidence	European Union	Female	Age-standardized	Periodontal diseases	Rate	2014	906.68	1052.92	783.12
2013	Incidence	European Union	Both	Age-standardized	Periodontal diseases	Rate	2014	906.58	1056.88	782.91
2014	Incidence	European Union	Male	Age-standardized	Periodontal diseases	Rate	2015	905.94	1059.80	782.24
2015	Incidence	European Union	Female	Age-standardized	Periodontal diseases	Rate	2015	906.81	1053.93	782.09
2016	Incidence	European Union	Both	Age-standardized	Periodontal diseases	Rate	2015	906.80	1058.46	782.24
2017	Incidence	European Union	Male	Age-standardized	Periodontal diseases	Rate	2016	907.56	1062.00	782.33
2018	Incidence	European Union	Female	Age-standardized	Periodontal diseases	Rate	2016	908.11	1055.42	780.59

2019	Incidence	European Union	Both	Age-standardized	Periodontal diseases	Rate	2016	908.25	1060.16	782.54
2020	Incidence	European Union	Male	Age-standardized	Periodontal diseases	Rate	2017	909.47	1064.46	782.78
2021	Incidence	European Union	Female	Age-standardized	Periodontal diseases	Rate	2017	909.80	1057.53	778.38
2022	Incidence	European Union	Both	Age-standardized	Periodontal diseases	Rate	2017	910.03	1062.44	781.43
2023	Incidence	European Union	Male	Age-standardized	Periodontal diseases	Rate	2018	911.14	1066.16	781.06
2024	Incidence	European Union	Female	Age-standardized	Periodontal diseases	Rate	2018	911.42	1060.86	777.91
2025	Incidence	European Union	Both	Age-standardized	Periodontal diseases	Rate	2018	911.65	1064.77	779.42
2026	Incidence	European Union	Male	Age-standardized	Periodontal diseases	Rate	2019	911.64	1065.86	780.47
2027	Incidence	European Union	Female	Age-standardized	Periodontal diseases	Rate	2019	912.07	1063.03	776.44
2028	Incidence	European Union	Both	Age-standardized	Periodontal diseases	Rate	2019	912.21	1064.81	778.12
2029	Incidence	European Union	Male	Age-standardized	Periodontal diseases	Rate	2020	910.82	1061.17	774.52
2030	Incidence	European Union	Female	Age-standardized	Periodontal diseases	Rate	2020	911.20	1064.40	767.52
2031	Incidence	European Union	Both	Age-standardized	Periodontal diseases	Rate	2020	911.32	1063.11	772.22
2032	Incidence	European Union	Male	Age-standardized	Periodontal diseases	Rate	2021	906.94	1060.41	759.09
2033	Incidence	European Union	Female	Age-standardized	Periodontal diseases	Rate	2021	907.25	1067.07	760.68
2034	Incidence	European Union	Both	Age-standardized	Periodontal diseases	Rate	2021	907.38	1062.83	759.52
2035	Incidence	European Union	Male	Age-standardized	Periodontal diseases	Rate	2022	904.04	1063.16	756.16
2036	Incidence	European Union	Female	Age-standardized	Periodontal diseases	Rate	2022	907.37	1060.91	763.36
2037	Incidence	European Union	Both	Age-standardized	Periodontal diseases	Rate	2022	905.99	1063.81	759.18
2038	Incidence	European Union	Male	Age-standardized	Periodontal diseases	Rate	2023	897.37	1054.97	750.44
2039	Incidence	European Union	Female	Age-standardized	Periodontal diseases	Rate	2023	900.74	1053.58	757.61
2040	Incidence	European Union	Both	Age-standardized	Periodontal diseases	Rate	2023	899.34	1056.31	753.42

Table S2. Trends in age-standardized DALY rates of periodontal diseases in China and G20 countries, 1990–2023

	measure_name	location_name	sex_name	age_name	cause_name	metric_name	year	val	upper	lower
1	DALYs (Disability-Adjusted Life Years)	Republic of Turkey	Male	Age-standardized	Periodontal diseases	Rate	1990	40.78	93.13	15.11
2	DALYs (Disability-Adjusted Life Years)	Republic of Turkey	Female	Age-standardized	Periodontal diseases	Rate	1990	41.29	91.55	15.74
3	DALYs (Disability-Adjusted Life Years)	Republic of Turkey	Both	Age-standardized	Periodontal diseases	Rate	1990	41.11	92.44	15.34
4	DALYs (Disability-Adjusted Life Years)	Argentine Republic	Male	Age-standardized	Periodontal diseases	Rate	1990	83.23	176.29	32.86
5	DALYs (Disability-Adjusted Life Years)	Argentine Republic	Female	Age-standardized	Periodontal diseases	Rate	1990	70.37	151.44	27.05
6	DALYs (Disability-Adjusted Life Years)	Argentine Republic	Both	Age-standardized	Periodontal diseases	Rate	1990	76.46	162.84	29.83
7	DALYs (Disability-Adjusted Life Years)	Russian Federation	Male	Age-standardized	Periodontal diseases	Rate	1990	77.18	161.48	30.89
8	DALYs (Disability-Adjusted Life Years)	Russian Federation	Female	Age-standardized	Periodontal diseases	Rate	1990	77.34	162.09	31.45
9	DALYs (Disability-Adjusted Life Years)	Russian Federation	Both	Age-standardized	Periodontal diseases	Rate	1990	77.11	161.49	31.13
10	DALYs (Disability-Adjusted Life Years)	Republic of Italy	Male	Age-standardized	Periodontal diseases	Rate	1990	61.65	135.39	24.02
11	DALYs (Disability-Adjusted Life Years)	Republic of Italy	Female	Age-standardized	Periodontal diseases	Rate	1990	70.42	149.08	27.78
12	DALYs (Disability-Adjusted Life Years)	Republic of Italy	Both	Age-standardized	Periodontal diseases	Rate	1990	66.14	143.64	25.72
13	DALYs (Disability-Adjusted Life Years)	Federative Republic of Brazil	Male	Age-standardized	Periodontal diseases	Rate	1990	76.86	160.16	31.15
14	DALYs (Disability-Adjusted Life	Federative Republic of	Female	Age-standardized	Periodontal diseases	Rate	1990	63.27	135.01	25.30

	Years)	Brazil								
15	DALYs (Disability-Adjusted Life Years)	Federative Republic of Brazil	Both	Age-standardized	Periodontal diseases	Rate	1990	69.83	146.99	28.49
16	DALYs (Disability-Adjusted Life Years)	French Republic	Male	Age-standardized	Periodontal diseases	Rate	1991	39.97	87.88	15.05
17	DALYs (Disability-Adjusted Life Years)	French Republic	Female	Age-standardized	Periodontal diseases	Rate	1991	38.84	83.49	15.18
18	DALYs (Disability-Adjusted Life Years)	French Republic	Both	Age-standardized	Periodontal diseases	Rate	1991	39.42	78.17	15.66
19	DALYs (Disability-Adjusted Life Years)	Republic of South Africa	Male	Age-standardized	Periodontal diseases	Rate	1990	51.80	112.16	19.38
20	DALYs (Disability-Adjusted Life Years)	Republic of South Africa	Female	Age-standardized	Periodontal diseases	Rate	1990	49.72	107.65	18.59
21	DALYs (Disability-Adjusted Life Years)	Republic of South Africa	Both	Age-standardized	Periodontal diseases	Rate	1990	50.71	109.76	18.94
22	DALYs (Disability-Adjusted Life Years)	Republic of India	Male	Age-standardized	Periodontal diseases	Rate	1991	103.05	211.42	41.53
23	DALYs (Disability-Adjusted Life Years)	Republic of India	Female	Age-standardized	Periodontal diseases	Rate	1991	101.57	207.07	40.97
24	DALYs (Disability-Adjusted Life Years)	Republic of India	Both	Age-standardized	Periodontal diseases	Rate	1991	102.33	209.25	41.26
25	DALYs (Disability-Adjusted Life Years)	Federal Republic of Germany	Male	Age-standardized	Periodontal diseases	Rate	1991	121.98	257.31	47.54
26	DALYs (Disability-Adjusted Life Years)	Federal Republic of Germany	Female	Age-standardized	Periodontal diseases	Rate	1991	108.54	229.82	42.72
27	DALYs (Disability-Adjusted Life Years)	Federal Republic of Germany	Both	Age-standardized	Periodontal diseases	Rate	1991	114.98	243.15	44.98
28	DALYs (Disability-Adjusted Life Years)	People's Republic of China	Male	Age-standardized	Periodontal diseases	Rate	1990	74.47	154.96	29.28
29	DALYs	People's	Female	Age-standardized	Periodontal	Rate	1990	68.69	143.30	27.14

	(Disability-Adjusted Life Years)	Republic of China			diseases					
30	DALYs (Disability-Adjusted Life Years)	People's Republic of China	Both	Age-standardized	Periodontal diseases	Rate	1990	71.61	149.23	28.22
31	DALYs (Disability-Adjusted Life Years)	Republic of Indonesia	Male	Age-standardized	Periodontal diseases	Rate	1991	82.18	172.31	32.62
32	DALYs (Disability-Adjusted Life Years)	Republic of Indonesia	Female	Age-standardized	Periodontal diseases	Rate	1991	90.05	192.40	36.16
33	DALYs (Disability-Adjusted Life Years)	Republic of Indonesia	Both	Age-standardized	Periodontal diseases	Rate	1991	86.10	182.36	34.57
34	DALYs (Disability-Adjusted Life Years)	Republic of Korea	Male	Age-standardized	Periodontal diseases	Rate	1991	60.04	128.24	23.48
35	DALYs (Disability-Adjusted Life Years)	Republic of Korea	Female	Age-standardized	Periodontal diseases	Rate	1991	39.02	80.68	14.64
36	DALYs (Disability-Adjusted Life Years)	Republic of Korea	Both	Age-standardized	Periodontal diseases	Rate	1991	48.92	105.21	18.49
37	DALYs (Disability-Adjusted Life Years)	Kingdom of Saudi Arabia	Male	Age-standardized	Periodontal diseases	Rate	1991	51.76	110.24	20.59
38	DALYs (Disability-Adjusted Life Years)	Kingdom of Saudi Arabia	Female	Age-standardized	Periodontal diseases	Rate	1991	47.76	101.87	18.36
39	DALYs (Disability-Adjusted Life Years)	Kingdom of Saudi Arabia	Both	Age-standardized	Periodontal diseases	Rate	1991	50.34	107.04	19.74
40	DALYs (Disability-Adjusted Life Years)	Kingdom of Saudi Arabia	Male	Age-standardized	Periodontal diseases	Rate	1990	48.74	104.90	19.31
41	DALYs (Disability-Adjusted Life Years)	Kingdom of Saudi Arabia	Female	Age-standardized	Periodontal diseases	Rate	1990	44.73	97.50	17.32
42	DALYs (Disability-Adjusted Life Years)	Kingdom of Saudi Arabia	Both	Age-standardized	Periodontal diseases	Rate	1990	47.31	102.44	18.48
43	DALYs (Disability-Adjusted Life Years)	Republic of Korea	Male	Age-standardized	Periodontal diseases	Rate	1990	59.01	127.56	23.17

44	DALYs (Disability-Adjusted Life Years)	Republic of Korea	Female	Age-standardized	Periodontal diseases	Rate	1990	38.79	85.33	14.03
45	DALYs (Disability-Adjusted Life Years)	Republic of Korea	Both	Age-standardized	Periodontal diseases	Rate	1990	48.31	104.06	18.10
46	DALYs (Disability-Adjusted Life Years)	United Kingdom of Great Britain and Northern Ireland	Male	Age-standardized	Periodontal diseases	Rate	1991	41.09	91.01	15.23
47	DALYs (Disability-Adjusted Life Years)	United Kingdom of Great Britain and Northern Ireland	Female	Age-standardized	Periodontal diseases	Rate	1991	40.07	88.78	14.96
48	DALYs (Disability-Adjusted Life Years)	United Kingdom of Great Britain and Northern Ireland	Both	Age-standardized	Periodontal diseases	Rate	1991	40.55	89.80	15.08
49	DALYs (Disability-Adjusted Life Years)	Republic of India	Male	Age-standardized	Periodontal diseases	Rate	1990	101.78	209.96	41.09
50	DALYs (Disability-Adjusted Life Years)	Republic of India	Female	Age-standardized	Periodontal diseases	Rate	1990	100.47	205.78	40.48
51	DALYs (Disability-Adjusted Life Years)	Republic of India	Both	Age-standardized	Periodontal diseases	Rate	1990	101.14	207.94	40.80
52	DALYs (Disability-Adjusted Life Years)	Federal Republic of Germany	Male	Age-standardized	Periodontal diseases	Rate	1990	114.25	238.38	44.40
53	DALYs (Disability-Adjusted Life Years)	Federal Republic of Germany	Female	Age-standardized	Periodontal diseases	Rate	1990	100.20	210.92	39.50
54	DALYs (Disability-Adjusted Life Years)	Federal Republic of Germany	Both	Age-standardized	Periodontal diseases	Rate	1990	106.90	224.16	41.62
55	DALYs (Disability-Adjusted Life Years)	United Mexican States	Male	Age-standardized	Periodontal diseases	Rate	1991	100.22	205.80	40.49
56	DALYs (Disability-Adjusted Life	United Mexican States	Female	Age-standardized	Periodontal diseases	Rate	1991	89.05	188.53	35.52

	Years)									
57	DALYs (Disability-Adjusted Life Years)	United Mexican States	Both	Age-standardized	Periodontal diseases	Rate	1991	94.41	196.83	37.91
58	DALYs (Disability-Adjusted Life Years)	Japan	Male	Age-standardized	Periodontal diseases	Rate	1990	68.91	146.12	27.23
59	DALYs (Disability-Adjusted Life Years)	Japan	Female	Age-standardized	Periodontal diseases	Rate	1990	49.07	104.80	18.60
60	DALYs (Disability-Adjusted Life Years)	Japan	Both	Age-standardized	Periodontal diseases	Rate	1990	58.76	126.88	22.87
61	DALYs (Disability-Adjusted Life Years)	Canada	Male	Age-standardized	Periodontal diseases	Rate	1990	100.41	212.12	40.93
62	DALYs (Disability-Adjusted Life Years)	Canada	Female	Age-standardized	Periodontal diseases	Rate	1990	85.37	176.69	34.65
63	DALYs (Disability-Adjusted Life Years)	Canada	Both	Age-standardized	Periodontal diseases	Rate	1990	92.75	194.72	37.12
64	DALYs (Disability-Adjusted Life Years)	European Union	Male	Age-standardized	Periodontal diseases	Rate	1991	75.81	158.36	29.59
65	DALYs (Disability-Adjusted Life Years)	European Union	Female	Age-standardized	Periodontal diseases	Rate	1991	70.20	145.72	27.34
66	DALYs (Disability-Adjusted Life Years)	European Union	Both	Age-standardized	Periodontal diseases	Rate	1991	72.90	150.88	28.39
67	DALYs (Disability-Adjusted Life Years)	Republic of South Africa	Male	Age-standardized	Periodontal diseases	Rate	1991	51.77	111.89	19.43
68	DALYs (Disability-Adjusted Life Years)	Republic of South Africa	Female	Age-standardized	Periodontal diseases	Rate	1991	50.17	107.96	18.72
69	DALYs (Disability-Adjusted Life Years)	Republic of South Africa	Both	Age-standardized	Periodontal diseases	Rate	1991	50.94	109.83	19.06
70	DALYs (Disability-Adjusted Life Years)	United States of America	Male	Age-standardized	Periodontal diseases	Rate	1990	71.00	148.49	27.76
71	DALYs	United States of	Female	Age-standardized	Periodontal	Rate	1990	54.83	116.99	20.68

	(Disability-Adjusted Life Years)	America			diseases					
72	DALYs (Disability-Adjusted Life Years)	United States of America	Both	Age-standardized	Periodontal diseases	Rate	1990	62.68	133.47	24.08
73	DALYs (Disability-Adjusted Life Years)	Federative Republic of Brazil	Male	Age-standardized	Periodontal diseases	Rate	1991	75.23	156.71	30.59
74	DALYs (Disability-Adjusted Life Years)	Federative Republic of Brazil	Female	Age-standardized	Periodontal diseases	Rate	1991	62.58	133.04	25.03
75	DALYs (Disability-Adjusted Life Years)	Federative Republic of Brazil	Both	Age-standardized	Periodontal diseases	Rate	1991	68.67	144.22	28.04
76	DALYs (Disability-Adjusted Life Years)	Russian Federation	Male	Age-standardized	Periodontal diseases	Rate	1991	77.01	161.13	30.87
77	DALYs (Disability-Adjusted Life Years)	Russian Federation	Female	Age-standardized	Periodontal diseases	Rate	1991	76.90	161.13	31.16
78	DALYs (Disability-Adjusted Life Years)	Russian Federation	Both	Age-standardized	Periodontal diseases	Rate	1991	76.79	160.82	30.97
79	DALYs (Disability-Adjusted Life Years)	Argentine Republic	Male	Age-standardized	Periodontal diseases	Rate	1991	83.97	178.88	33.02
80	DALYs (Disability-Adjusted Life Years)	Argentine Republic	Female	Age-standardized	Periodontal diseases	Rate	1991	71.12	153.91	27.55
81	DALYs (Disability-Adjusted Life Years)	Argentine Republic	Both	Age-standardized	Periodontal diseases	Rate	1991	77.20	165.12	30.18
82	DALYs (Disability-Adjusted Life Years)	French Republic	Male	Age-standardized	Periodontal diseases	Rate	1990	43.54	98.50	16.49
83	DALYs (Disability-Adjusted Life Years)	French Republic	Female	Age-standardized	Periodontal diseases	Rate	1990	41.96	91.59	16.57
84	DALYs (Disability-Adjusted Life Years)	French Republic	Both	Age-standardized	Periodontal diseases	Rate	1990	42.78	83.92	16.65
85	DALYs (Disability-Adjusted Life Years)	Federative Republic of Brazil	Male	Age-standardized	Periodontal diseases	Rate	1992	74.24	154.54	30.29

86	DALYs (Disability-Adjusted Life Years)	Federative Republic of Brazil	Female	Age-standardized	Periodontal diseases	Rate	1992	62.19	132.34	24.93
87	DALYs (Disability-Adjusted Life Years)	Federative Republic of Brazil	Both	Age-standardized	Periodontal diseases	Rate	1992	67.98	142.76	27.70
88	DALYs (Disability-Adjusted Life Years)	Republic of Indonesia	Male	Age-standardized	Periodontal diseases	Rate	1990	81.74	170.93	32.47
89	DALYs (Disability-Adjusted Life Years)	Republic of Indonesia	Female	Age-standardized	Periodontal diseases	Rate	1990	89.84	191.76	36.05
90	DALYs (Disability-Adjusted Life Years)	Republic of Indonesia	Both	Age-standardized	Periodontal diseases	Rate	1990	85.78	181.36	34.48
91	DALYs (Disability-Adjusted Life Years)	Australia	Male	Age-standardized	Periodontal diseases	Rate	1991	58.95	121.01	22.82
92	DALYs (Disability-Adjusted Life Years)	Australia	Female	Age-standardized	Periodontal diseases	Rate	1991	40.15	85.45	15.30
93	DALYs (Disability-Adjusted Life Years)	Australia	Both	Age-standardized	Periodontal diseases	Rate	1991	49.35	103.16	19.09
94	DALYs (Disability-Adjusted Life Years)	Republic of South Africa	Male	Age-standardized	Periodontal diseases	Rate	1992	51.72	111.71	19.35
95	DALYs (Disability-Adjusted Life Years)	Republic of South Africa	Female	Age-standardized	Periodontal diseases	Rate	1992	50.48	108.75	18.86
96	DALYs (Disability-Adjusted Life Years)	Republic of South Africa	Both	Age-standardized	Periodontal diseases	Rate	1992	51.08	110.18	19.10
97	DALYs (Disability-Adjusted Life Years)	Republic of Turkey	Male	Age-standardized	Periodontal diseases	Rate	1991	40.80	93.47	15.00
98	DALYs (Disability-Adjusted Life Years)	Republic of Turkey	Female	Age-standardized	Periodontal diseases	Rate	1991	41.31	90.84	15.74
99	DALYs (Disability-Adjusted Life Years)	Republic of Turkey	Both	Age-standardized	Periodontal diseases	Rate	1991	41.13	92.24	15.34
100	DALYs (Disability-Adjusted Life	Republic of Korea	Male	Age-standardized	Periodontal diseases	Rate	1993	61.31	129.13	23.79

	Years)									
101	DALYs (Disability-Adjusted Life Years)	Republic of Korea	Female	Age-standardized	Periodontal diseases	Rate	1993	39.45	77.80	15.36
102	DALYs (Disability-Adjusted Life Years)	Republic of Korea	Both	Age-standardized	Periodontal diseases	Rate	1993	49.77	103.09	18.74
103	DALYs (Disability-Adjusted Life Years)	Federal Republic of Germany	Male	Age-standardized	Periodontal diseases	Rate	1993	134.70	287.12	52.98
104	DALYs (Disability-Adjusted Life Years)	Federal Republic of Germany	Female	Age-standardized	Periodontal diseases	Rate	1993	122.27	257.88	49.33
105	DALYs (Disability-Adjusted Life Years)	Federal Republic of Germany	Both	Age-standardized	Periodontal diseases	Rate	1993	128.28	272.13	51.01
106	DALYs (Disability-Adjusted Life Years)	People's Republic of China	Male	Age-standardized	Periodontal diseases	Rate	1991	70.46	147.41	27.52
107	DALYs (Disability-Adjusted Life Years)	People's Republic of China	Female	Age-standardized	Periodontal diseases	Rate	1991	64.87	136.31	25.40
108	DALYs (Disability-Adjusted Life Years)	People's Republic of China	Both	Age-standardized	Periodontal diseases	Rate	1991	67.69	141.93	26.46
109	DALYs (Disability-Adjusted Life Years)	Republic of India	Male	Age-standardized	Periodontal diseases	Rate	1992	104.24	212.79	41.95
110	DALYs (Disability-Adjusted Life Years)	Republic of India	Female	Age-standardized	Periodontal diseases	Rate	1992	102.57	208.75	41.42
111	DALYs (Disability-Adjusted Life Years)	Republic of India	Both	Age-standardized	Periodontal diseases	Rate	1992	103.43	210.45	41.69
112	DALYs (Disability-Adjusted Life Years)	Republic of India	Male	Age-standardized	Periodontal diseases	Rate	1993	105.29	213.97	42.27
113	DALYs (Disability-Adjusted Life Years)	Republic of India	Female	Age-standardized	Periodontal diseases	Rate	1993	103.49	209.81	41.84
114	DALYs (Disability-Adjusted Life Years)	Republic of India	Both	Age-standardized	Periodontal diseases	Rate	1993	104.42	211.77	42.06
115	DALYs	Republic of	Male	Age-standardized	Periodontal	Rate	1991	61.88	135.82	24.08

	(Disability-Adjusted Life Years)	Italy			diseases						
116	DALYs (Disability-Adjusted Life Years)	Republic of Italy	Female	Age-standardized	Periodontal diseases	Rate	1991	70.60	149.33	27.85
117	DALYs (Disability-Adjusted Life Years)	Republic of Italy	Both	Age-standardized	Periodontal diseases	Rate	1991	66.34	143.83	25.82
118	DALYs (Disability-Adjusted Life Years)	United Mexican States	Male	Age-standardized	Periodontal diseases	Rate	1990	100.02	205.45	40.43
119	DALYs (Disability-Adjusted Life Years)	United Mexican States	Female	Age-standardized	Periodontal diseases	Rate	1990	89.21	189.01	35.55
120	DALYs (Disability-Adjusted Life Years)	United Mexican States	Both	Age-standardized	Periodontal diseases	Rate	1990	94.40	196.91	37.89
121	DALYs (Disability-Adjusted Life Years)	United States of America	Male	Age-standardized	Periodontal diseases	Rate	1991	71.61	149.60	28.10
122	DALYs (Disability-Adjusted Life Years)	United States of America	Female	Age-standardized	Periodontal diseases	Rate	1991	54.91	117.15	20.68
123	DALYs (Disability-Adjusted Life Years)	United States of America	Both	Age-standardized	Periodontal diseases	Rate	1991	63.00	134.04	24.25
124	DALYs (Disability-Adjusted Life Years)	Kingdom of Saudi Arabia	Male	Age-standardized	Periodontal diseases	Rate	1993	56.31	119.30	22.16
125	DALYs (Disability-Adjusted Life Years)	Kingdom of Saudi Arabia	Female	Age-standardized	Periodontal diseases	Rate	1993	52.33	112.06	20.09
126	DALYs (Disability-Adjusted Life Years)	Kingdom of Saudi Arabia	Both	Age-standardized	Periodontal diseases	Rate	1993	54.91	117.31	21.40
127	DALYs (Disability-Adjusted Life Years)	United Kingdom of Great Britain and Northern Ireland	Male	Age-standardized	Periodontal diseases	Rate	1990	41.01	90.66	15.15
128	DALYs (Disability-Adjusted Life Years)	United Kingdom of Great Britain and Northern	Female	Age-standardized	Periodontal diseases	Rate	1990	39.83	88.53	14.91

		Ireland								
129	DALYs (Disability-Adjusted Life Years)	United Kingdom of Great Britain and Northern Ireland	Both	Age-standardized	Periodontal diseases	Rate	1990	40.38	89.50	15.02
130	DALYs (Disability-Adjusted Life Years)	Canada	Male	Age-standardized	Periodontal diseases	Rate	1991	99.67	201.29	39.91
131	DALYs (Disability-Adjusted Life Years)	Canada	Female	Age-standardized	Periodontal diseases	Rate	1991	82.59	173.13	32.85
132	DALYs (Disability-Adjusted Life Years)	Canada	Both	Age-standardized	Periodontal diseases	Rate	1991	90.99	188.79	35.75
133	DALYs (Disability-Adjusted Life Years)	Republic of Korea	Male	Age-standardized	Periodontal diseases	Rate	1992	60.82	128.41	23.88
134	DALYs (Disability-Adjusted Life Years)	Republic of Korea	Female	Age-standardized	Periodontal diseases	Rate	1992	39.23	78.62	14.75
135	DALYs (Disability-Adjusted Life Years)	Republic of Korea	Both	Age-standardized	Periodontal diseases	Rate	1992	49.41	105.53	18.69
136	DALYs (Disability-Adjusted Life Years)	Federal Republic of Germany	Male	Age-standardized	Periodontal diseases	Rate	1992	128.97	272.98	50.77
137	DALYs (Disability-Adjusted Life Years)	Federal Republic of Germany	Female	Age-standardized	Periodontal diseases	Rate	1992	116.01	245.55	46.13
138	DALYs (Disability-Adjusted Life Years)	Federal Republic of Germany	Both	Age-standardized	Periodontal diseases	Rate	1992	122.25	258.88	48.14
139	DALYs (Disability-Adjusted Life Years)	Australia	Male	Age-standardized	Periodontal diseases	Rate	1990	64.38	139.27	25.41
140	DALYs (Disability-Adjusted Life Years)	Australia	Female	Age-standardized	Periodontal diseases	Rate	1990	40.47	93.47	15.10
141	DALYs (Disability-Adjusted Life Years)	Australia	Both	Age-standardized	Periodontal diseases	Rate	1990	52.15	115.96	20.19
142	DALYs (Disability-Adjusted Life	European Union	Male	Age-standardized	Periodontal diseases	Rate	1990	74.39	157.03	29.09

	Years)										
143	DALYs (Disability-Adjusted Life Years)	European Union	Female	Age-standardized	Periodontal diseases	Rate	1990	68.78	142.55	26.88
144	DALYs (Disability-Adjusted Life Years)	European Union	Both	Age-standardized	Periodontal diseases	Rate	1990	71.47	148.90	27.87
145	DALYs (Disability-Adjusted Life Years)	Kingdom of Saudi Arabia	Male	Age-standardized	Periodontal diseases	Rate	1992	54.29	115.88	21.35
146	DALYs (Disability-Adjusted Life Years)	Kingdom of Saudi Arabia	Female	Age-standardized	Periodontal diseases	Rate	1992	50.35	107.33	19.34
147	DALYs (Disability-Adjusted Life Years)	Kingdom of Saudi Arabia	Both	Age-standardized	Periodontal diseases	Rate	1992	52.90	113.50	20.61
148	DALYs (Disability-Adjusted Life Years)	Japan	Male	Age-standardized	Periodontal diseases	Rate	1991	71.24	150.69	28.18
149	DALYs (Disability-Adjusted Life Years)	Japan	Female	Age-standardized	Periodontal diseases	Rate	1991	51.28	109.26	19.57
150	DALYs (Disability-Adjusted Life Years)	Japan	Both	Age-standardized	Periodontal diseases	Rate	1991	61.05	131.45	23.86
151	DALYs (Disability-Adjusted Life Years)	Argentine Republic	Male	Age-standardized	Periodontal diseases	Rate	1992	84.41	180.46	33.73
152	DALYs (Disability-Adjusted Life Years)	Argentine Republic	Female	Age-standardized	Periodontal diseases	Rate	1992	71.56	156.40	27.91
153	DALYs (Disability-Adjusted Life Years)	Argentine Republic	Both	Age-standardized	Periodontal diseases	Rate	1992	77.64	167.32	30.72
154	DALYs (Disability-Adjusted Life Years)	Republic of Italy	Male	Age-standardized	Periodontal diseases	Rate	1992	62.07	136.45	24.09
155	DALYs (Disability-Adjusted Life Years)	Republic of Italy	Female	Age-standardized	Periodontal diseases	Rate	1992	70.74	149.33	27.93
156	DALYs (Disability-Adjusted Life Years)	Republic of Italy	Both	Age-standardized	Periodontal diseases	Rate	1992	66.51	143.81	25.94
157	DALYs	Federative	Male	Age-standardized	Periodontal	Rate	1993	73.76	154.06	30.16

	(Disability-Adjusted Life Years)	Republic of Brazil			diseases					
158	DALYs (Disability-Adjusted Life Years)	Federative Republic of Brazil	Female	Age-standardized	Periodontal diseases	Rate	1993	62.04	131.45	24.87
159	DALYs (Disability-Adjusted Life Years)	Federative Republic of Brazil	Both	Age-standardized	Periodontal diseases	Rate	1993	67.66	141.89	27.57
160	DALYs (Disability-Adjusted Life Years)	Republic of South Africa	Male	Age-standardized	Periodontal diseases	Rate	1993	51.65	110.98	19.29
161	DALYs (Disability-Adjusted Life Years)	Republic of South Africa	Female	Age-standardized	Periodontal diseases	Rate	1993	50.66	108.56	18.99
162	DALYs (Disability-Adjusted Life Years)	Republic of South Africa	Both	Age-standardized	Periodontal diseases	Rate	1993	51.15	109.74	19.15
163	DALYs (Disability-Adjusted Life Years)	Federative Republic of Brazil	Male	Age-standardized	Periodontal diseases	Rate	1995	73.69	153.88	30.04
164	DALYs (Disability-Adjusted Life Years)	Federative Republic of Brazil	Female	Age-standardized	Periodontal diseases	Rate	1995	62.14	131.14	25.03
165	DALYs (Disability-Adjusted Life Years)	Federative Republic of Brazil	Both	Age-standardized	Periodontal diseases	Rate	1995	67.67	141.67	27.56
166	DALYs (Disability-Adjusted Life Years)	French Republic	Male	Age-standardized	Periodontal diseases	Rate	1993	35.21	70.26	14.04
167	DALYs (Disability-Adjusted Life Years)	French Republic	Female	Age-standardized	Periodontal diseases	Rate	1993	34.81	74.54	13.60
168	DALYs (Disability-Adjusted Life Years)	French Republic	Both	Age-standardized	Periodontal diseases	Rate	1993	35.00	69.36	14.24
169	DALYs (Disability-Adjusted Life Years)	Russian Federation	Male	Age-standardized	Periodontal diseases	Rate	1992	76.81	160.76	30.77
170	DALYs (Disability-Adjusted Life Years)	Russian Federation	Female	Age-standardized	Periodontal diseases	Rate	1992	76.49	159.95	30.83
171	DALYs (Disability-Adjusted Life Years)	Russian Federation	Both	Age-standardized	Periodontal diseases	Rate	1992	76.47	160.00	30.75

172	DALYs (Disability-Adjusted Life Years)	Canada	Male	Age-standardized	Periodontal diseases	Rate	1992	99.02	197.60	39.26
173	DALYs (Disability-Adjusted Life Years)	Canada	Female	Age-standardized	Periodontal diseases	Rate	1992	80.36	163.90	31.37
174	DALYs (Disability-Adjusted Life Years)	Canada	Both	Age-standardized	Periodontal diseases	Rate	1992	89.53	181.77	34.98
175	DALYs (Disability-Adjusted Life Years)	Republic of Indonesia	Male	Age-standardized	Periodontal diseases	Rate	1993	82.96	174.94	33.00
176	DALYs (Disability-Adjusted Life Years)	Republic of Indonesia	Female	Age-standardized	Periodontal diseases	Rate	1993	90.53	193.24	36.32
177	DALYs (Disability-Adjusted Life Years)	Republic of Indonesia	Both	Age-standardized	Periodontal diseases	Rate	1993	86.73	184.07	34.82
178	DALYs (Disability-Adjusted Life Years)	Japan	Male	Age-standardized	Periodontal diseases	Rate	1992	72.94	153.49	28.87
179	DALYs (Disability-Adjusted Life Years)	Japan	Female	Age-standardized	Periodontal diseases	Rate	1992	53.23	113.30	20.29
180	DALYs (Disability-Adjusted Life Years)	Japan	Both	Age-standardized	Periodontal diseases	Rate	1992	62.91	135.15	24.65
181	DALYs (Disability-Adjusted Life Years)	Republic of Korea	Male	Age-standardized	Periodontal diseases	Rate	1996	61.29	127.85	23.26
182	DALYs (Disability-Adjusted Life Years)	Republic of Korea	Female	Age-standardized	Periodontal diseases	Rate	1996	39.99	82.65	15.85
183	DALYs (Disability-Adjusted Life Years)	Republic of Korea	Both	Age-standardized	Periodontal diseases	Rate	1996	50.10	99.77	19.24
184	DALYs (Disability-Adjusted Life Years)	French Republic	Male	Age-standardized	Periodontal diseases	Rate	1992	37.19	76.41	14.33
185	DALYs (Disability-Adjusted Life Years)	French Republic	Female	Age-standardized	Periodontal diseases	Rate	1992	36.47	78.26	14.25
186	DALYs (Disability-Adjusted Life	French Republic	Both	Age-standardized	Periodontal diseases	Rate	1992	36.83	72.79	15.01

	Years)									
187	DALYs (Disability-Adjusted Life Years)	Federal Republic of Germany	Male	Age-standardized	Periodontal diseases	Rate	1995	139.97	294.44	55.67
188	DALYs (Disability-Adjusted Life Years)	Federal Republic of Germany	Female	Age-standardized	Periodontal diseases	Rate	1995	128.15	266.38	52.00
189	DALYs (Disability-Adjusted Life Years)	Federal Republic of Germany	Both	Age-standardized	Periodontal diseases	Rate	1995	133.89	280.09	54.01
190	DALYs (Disability-Adjusted Life Years)	Republic of Korea	Male	Age-standardized	Periodontal diseases	Rate	1995	61.73	127.59	23.95
191	DALYs (Disability-Adjusted Life Years)	Republic of Korea	Female	Age-standardized	Periodontal diseases	Rate	1995	39.80	82.78	15.73
192	DALYs (Disability-Adjusted Life Years)	Republic of Korea	Both	Age-standardized	Periodontal diseases	Rate	1995	50.19	100.38	18.80
193	DALYs (Disability-Adjusted Life Years)	Republic of South Africa	Male	Age-standardized	Periodontal diseases	Rate	1995	51.57	110.43	19.26
194	DALYs (Disability-Adjusted Life Years)	Republic of South Africa	Female	Age-standardized	Periodontal diseases	Rate	1995	50.77	108.30	19.09
195	DALYs (Disability-Adjusted Life Years)	Republic of South Africa	Both	Age-standardized	Periodontal diseases	Rate	1995	51.17	109.36	19.24
196	DALYs (Disability-Adjusted Life Years)	Republic of India	Male	Age-standardized	Periodontal diseases	Rate	1995	106.83	216.32	42.77
197	DALYs (Disability-Adjusted Life Years)	Republic of India	Female	Age-standardized	Periodontal diseases	Rate	1995	104.85	211.10	42.42
198	DALYs (Disability-Adjusted Life Years)	Republic of India	Both	Age-standardized	Periodontal diseases	Rate	1995	105.86	213.66	42.60
199	DALYs (Disability-Adjusted Life Years)	Federal Republic of Germany	Male	Age-standardized	Periodontal diseases	Rate	1996	140.07	296.73	55.40
200	DALYs (Disability-Adjusted Life Years)	Federal Republic of Germany	Female	Age-standardized	Periodontal diseases	Rate	1996	128.24	269.97	52.66
201	DALYs	Federal	Both	Age-standardized	Periodontal	Rate	1996	134.00	283.53	53.97

	(Disability-Adjusted Life Years)	Republic of Germany			diseases					
202	DALYs (Disability-Adjusted Life Years)	People's Republic of China	Male	Age-standardized	Periodontal diseases	Rate	1992	66.78	140.50	25.92
203	DALYs (Disability-Adjusted Life Years)	People's Republic of China	Female	Age-standardized	Periodontal diseases	Rate	1992	61.45	129.59	23.79
204	DALYs (Disability-Adjusted Life Years)	People's Republic of China	Both	Age-standardized	Periodontal diseases	Rate	1992	64.13	135.25	24.86
205	DALYs (Disability-Adjusted Life Years)	United Mexican States	Male	Age-standardized	Periodontal diseases	Rate	1993	100.52	205.93	40.61
206	DALYs (Disability-Adjusted Life Years)	United Mexican States	Female	Age-standardized	Periodontal diseases	Rate	1993	88.96	187.93	35.50
207	DALYs (Disability-Adjusted Life Years)	United Mexican States	Both	Age-standardized	Periodontal diseases	Rate	1993	94.51	196.59	37.95
208	DALYs (Disability-Adjusted Life Years)	Kingdom of Saudi Arabia	Male	Age-standardized	Periodontal diseases	Rate	1995	58.08	131.66	22.11
209	DALYs (Disability-Adjusted Life Years)	Kingdom of Saudi Arabia	Female	Age-standardized	Periodontal diseases	Rate	1995	54.03	114.94	20.55
210	DALYs (Disability-Adjusted Life Years)	Kingdom of Saudi Arabia	Both	Age-standardized	Periodontal diseases	Rate	1995	56.66	125.97	21.56
211	DALYs (Disability-Adjusted Life Years)	United States of America	Male	Age-standardized	Periodontal diseases	Rate	1992	72.01	150.59	28.32
212	DALYs (Disability-Adjusted Life Years)	United States of America	Female	Age-standardized	Periodontal diseases	Rate	1992	55.05	117.32	20.77
213	DALYs (Disability-Adjusted Life Years)	United States of America	Both	Age-standardized	Periodontal diseases	Rate	1992	63.26	134.50	24.41
214	DALYs (Disability-Adjusted Life Years)	Australia	Male	Age-standardized	Periodontal diseases	Rate	1993	51.76	107.21	20.22
215	DALYs (Disability-Adjusted Life Years)	Australia	Female	Age-standardized	Periodontal diseases	Rate	1993	40.37	82.52	16.17

216	DALYs (Disability-Adjusted Life Years)	Australia	Both	Age-standardized	Periodontal diseases	Rate	1993	45.96	94.92	18.02
217	DALYs (Disability-Adjusted Life Years)	United Kingdom of Great Britain and Northern Ireland	Male	Age-standardized	Periodontal diseases	Rate	1993	41.12	91.48	15.29
218	DALYs (Disability-Adjusted Life Years)	United Kingdom of Great Britain and Northern Ireland	Female	Age-standardized	Periodontal diseases	Rate	1993	40.20	88.63	15.00
219	DALYs (Disability-Adjusted Life Years)	United Kingdom of Great Britain and Northern Ireland	Both	Age-standardized	Periodontal diseases	Rate	1993	40.63	89.89	15.14
220	DALYs (Disability-Adjusted Life Years)	Kingdom of Saudi Arabia	Male	Age-standardized	Periodontal diseases	Rate	1996	57.71	124.19	21.57
221	DALYs (Disability-Adjusted Life Years)	Kingdom of Saudi Arabia	Female	Age-standardized	Periodontal diseases	Rate	1996	53.55	113.12	20.48
222	DALYs (Disability-Adjusted Life Years)	Kingdom of Saudi Arabia	Both	Age-standardized	Periodontal diseases	Rate	1996	56.23	120.44	21.16
223	DALYs (Disability-Adjusted Life Years)	Republic of Indonesia	Male	Age-standardized	Periodontal diseases	Rate	1992	82.60	173.32	32.87
224	DALYs (Disability-Adjusted Life Years)	Republic of Indonesia	Female	Age-standardized	Periodontal diseases	Rate	1992	90.29	193.05	36.21
225	DALYs (Disability-Adjusted Life Years)	Republic of Indonesia	Both	Age-standardized	Periodontal diseases	Rate	1992	86.42	183.18	34.75
226	DALYs (Disability-Adjusted Life Years)	Republic of India	Male	Age-standardized	Periodontal diseases	Rate	1996	107.39	217.66	43.05
227	DALYs (Disability-Adjusted Life Years)	Republic of India	Female	Age-standardized	Periodontal diseases	Rate	1996	105.39	211.81	42.62
228	DALYs (Disability-Adjusted Life	Republic of India	Both	Age-standardized	Periodontal diseases	Rate	1996	106.42	214.84	42.84

	Years)									
229	DALYs (Disability-Adjusted Life Years)	Republic of Italy	Male	Age-standardized	Periodontal diseases	Rate	1993	62.21	136.92	24.12
230	DALYs (Disability-Adjusted Life Years)	Republic of Italy	Female	Age-standardized	Periodontal diseases	Rate	1993	70.85	149.66	27.86
231	DALYs (Disability-Adjusted Life Years)	Republic of Italy	Both	Age-standardized	Periodontal diseases	Rate	1993	66.62	143.97	26.01
232	DALYs (Disability-Adjusted Life Years)	United Mexican States	Male	Age-standardized	Periodontal diseases	Rate	1992	100.39	205.81	40.50
233	DALYs (Disability-Adjusted Life Years)	United Mexican States	Female	Age-standardized	Periodontal diseases	Rate	1992	88.98	188.32	35.49
234	DALYs (Disability-Adjusted Life Years)	United Mexican States	Both	Age-standardized	Periodontal diseases	Rate	1992	94.46	196.73	37.89
235	DALYs (Disability-Adjusted Life Years)	Russian Federation	Male	Age-standardized	Periodontal diseases	Rate	1993	76.57	160.23	30.68
236	DALYs (Disability-Adjusted Life Years)	Russian Federation	Female	Age-standardized	Periodontal diseases	Rate	1993	76.08	158.89	30.56
237	DALYs (Disability-Adjusted Life Years)	Russian Federation	Both	Age-standardized	Periodontal diseases	Rate	1993	76.14	159.17	30.56
238	DALYs (Disability-Adjusted Life Years)	Federative Republic of Brazil	Male	Age-standardized	Periodontal diseases	Rate	1994	73.62	153.75	30.09
239	DALYs (Disability-Adjusted Life Years)	Federative Republic of Brazil	Female	Age-standardized	Periodontal diseases	Rate	1994	62.04	131.13	24.96
240	DALYs (Disability-Adjusted Life Years)	Federative Republic of Brazil	Both	Age-standardized	Periodontal diseases	Rate	1994	67.59	141.65	27.53
241	DALYs (Disability-Adjusted Life Years)	Argentine Republic	Male	Age-standardized	Periodontal diseases	Rate	1993	84.69	183.26	34.08
242	DALYs (Disability-Adjusted Life Years)	Argentine Republic	Female	Age-standardized	Periodontal diseases	Rate	1993	71.84	158.83	28.43
243	DALYs	Argentine	Both	Age-standardized	Periodontal	Rate	1993	77.91	170.33	31.13

	(Disability-Adjusted Life Years)	Republic			diseases					
244	DALYs (Disability-Adjusted Life Years)	United Kingdom of Great Britain and Northern Ireland	Male	Age-standardized	Periodontal diseases	Rate	1992	41.12	91.22	15.26
245	DALYs (Disability-Adjusted Life Years)	United Kingdom of Great Britain and Northern Ireland	Female	Age-standardized	Periodontal diseases	Rate	1992	40.19	88.76	15.01
246	DALYs (Disability-Adjusted Life Years)	United Kingdom of Great Britain and Northern Ireland	Both	Age-standardized	Periodontal diseases	Rate	1992	40.62	89.87	15.12
247	DALYs (Disability-Adjusted Life Years)	Republic of Turkey	Male	Age-standardized	Periodontal diseases	Rate	1993	41.24	93.34	15.36
248	DALYs (Disability-Adjusted Life Years)	Republic of Turkey	Female	Age-standardized	Periodontal diseases	Rate	1993	41.54	92.76	15.67
249	DALYs (Disability-Adjusted Life Years)	Republic of Turkey	Both	Age-standardized	Periodontal diseases	Rate	1993	41.47	93.21	15.54
250	DALYs (Disability-Adjusted Life Years)	Japan	Male	Age-standardized	Periodontal diseases	Rate	1993	74.09	155.47	29.32
251	DALYs (Disability-Adjusted Life Years)	Japan	Female	Age-standardized	Periodontal diseases	Rate	1993	54.79	116.67	20.70
252	DALYs (Disability-Adjusted Life Years)	Japan	Both	Age-standardized	Periodontal diseases	Rate	1993	64.30	137.95	25.23
253	DALYs (Disability-Adjusted Life Years)	Kingdom of Saudi Arabia	Male	Age-standardized	Periodontal diseases	Rate	1994	57.63	126.12	22.45
254	DALYs (Disability-Adjusted Life Years)	Kingdom of Saudi Arabia	Female	Age-standardized	Periodontal diseases	Rate	1994	53.55	114.94	20.49
255	DALYs (Disability-Adjusted Life Years)	Kingdom of Saudi Arabia	Both	Age-standardized	Periodontal diseases	Rate	1994	56.19	121.79	21.73

256	DALYs (Disability-Adjusted Life Years)	Federal Republic of Germany	Male	Age-standardized	Periodontal diseases	Rate	1994	138.54	293.46	54.70
257	DALYs (Disability-Adjusted Life Years)	Federal Republic of Germany	Female	Age-standardized	Periodontal diseases	Rate	1994	126.49	264.16	51.04
258	DALYs (Disability-Adjusted Life Years)	Federal Republic of Germany	Both	Age-standardized	Periodontal diseases	Rate	1994	132.33	278.53	53.08
259	DALYs (Disability-Adjusted Life Years)	Republic of India	Male	Age-standardized	Periodontal diseases	Rate	1994	106.16	214.96	42.58
260	DALYs (Disability-Adjusted Life Years)	Republic of India	Female	Age-standardized	Periodontal diseases	Rate	1994	104.25	210.71	42.19
261	DALYs (Disability-Adjusted Life Years)	Republic of India	Both	Age-standardized	Periodontal diseases	Rate	1994	105.24	212.86	42.39
262	DALYs (Disability-Adjusted Life Years)	Canada	Male	Age-standardized	Periodontal diseases	Rate	1993	98.46	197.90	38.58
263	DALYs (Disability-Adjusted Life Years)	Canada	Female	Age-standardized	Periodontal diseases	Rate	1993	78.66	155.07	30.55
264	DALYs (Disability-Adjusted Life Years)	Canada	Both	Age-standardized	Periodontal diseases	Rate	1993	88.39	175.85	34.64
265	DALYs (Disability-Adjusted Life Years)	Republic of Korea	Male	Age-standardized	Periodontal diseases	Rate	1997	60.29	121.15	22.75
266	DALYs (Disability-Adjusted Life Years)	Republic of Korea	Female	Age-standardized	Periodontal diseases	Rate	1997	40.27	83.06	15.83
267	DALYs (Disability-Adjusted Life Years)	Republic of Korea	Both	Age-standardized	Periodontal diseases	Rate	1997	49.79	98.51	19.44
268	DALYs (Disability-Adjusted Life Years)	European Union	Male	Age-standardized	Periodontal diseases	Rate	1992	77.12	159.56	30.02
269	DALYs (Disability-Adjusted Life Years)	European Union	Female	Age-standardized	Periodontal diseases	Rate	1992	71.49	148.63	27.82
270	DALYs (Disability-Adjusted Life	European Union	Both	Age-standardized	Periodontal diseases	Rate	1992	74.21	152.56	28.80

	Years)									
271	DALYs (Disability-Adjusted Life Years)	European Union	Male	Age-standardized	Periodontal diseases	Rate	1993	78.19	160.43	30.40
272	DALYs (Disability-Adjusted Life Years)	European Union	Female	Age-standardized	Periodontal diseases	Rate	1993	72.55	150.88	28.21
273	DALYs (Disability-Adjusted Life Years)	European Union	Both	Age-standardized	Periodontal diseases	Rate	1993	75.28	154.82	29.17
274	DALYs (Disability-Adjusted Life Years)	People's Republic of China	Male	Age-standardized	Periodontal diseases	Rate	1993	63.66	133.62	24.54
275	DALYs (Disability-Adjusted Life Years)	People's Republic of China	Female	Age-standardized	Periodontal diseases	Rate	1993	58.61	122.57	22.44
276	DALYs (Disability-Adjusted Life Years)	People's Republic of China	Both	Age-standardized	Periodontal diseases	Rate	1993	61.15	128.16	23.49
277	DALYs (Disability-Adjusted Life Years)	Republic of South Africa	Male	Age-standardized	Periodontal diseases	Rate	1994	51.60	110.75	19.34
278	DALYs (Disability-Adjusted Life Years)	Republic of South Africa	Female	Age-standardized	Periodontal diseases	Rate	1994	50.74	108.70	19.02
279	DALYs (Disability-Adjusted Life Years)	Republic of South Africa	Both	Age-standardized	Periodontal diseases	Rate	1994	51.17	109.73	19.26
280	DALYs (Disability-Adjusted Life Years)	Republic of Turkey	Male	Age-standardized	Periodontal diseases	Rate	1992	40.97	93.33	15.04
281	DALYs (Disability-Adjusted Life Years)	Republic of Turkey	Female	Age-standardized	Periodontal diseases	Rate	1992	41.34	91.53	15.67
282	DALYs (Disability-Adjusted Life Years)	Republic of Turkey	Both	Age-standardized	Periodontal diseases	Rate	1992	41.24	92.59	15.38
283	DALYs (Disability-Adjusted Life Years)	Federative Republic of Brazil	Male	Age-standardized	Periodontal diseases	Rate	1996	75.96	159.44	31.11
284	DALYs (Disability-Adjusted Life Years)	Federative Republic of Brazil	Female	Age-standardized	Periodontal diseases	Rate	1996	64.61	137.06	26.26
285	DALYs	Federative	Both	Age-standardized	Periodontal	Rate	1996	70.04	147.46	28.74

	(Disability-Adjusted Life Years)	Republic of Brazil			diseases					
286	DALYs (Disability-Adjusted Life Years)	Kingdom of Saudi Arabia	Male	Age-standardized	Periodontal diseases	Rate	1997	56.73	120.29	21.47
287	DALYs (Disability-Adjusted Life Years)	Kingdom of Saudi Arabia	Female	Age-standardized	Periodontal diseases	Rate	1997	52.36	106.62	20.11
288	DALYs (Disability-Adjusted Life Years)	Kingdom of Saudi Arabia	Both	Age-standardized	Periodontal diseases	Rate	1997	55.15	115.79	20.99
289	DALYs (Disability-Adjusted Life Years)	Republic of India	Male	Age-standardized	Periodontal diseases	Rate	1997	107.98	219.00	43.35
290	DALYs (Disability-Adjusted Life Years)	Republic of India	Female	Age-standardized	Periodontal diseases	Rate	1997	105.95	213.23	42.82
291	DALYs (Disability-Adjusted Life Years)	Republic of India	Both	Age-standardized	Periodontal diseases	Rate	1997	106.99	216.21	43.09
292	DALYs (Disability-Adjusted Life Years)	Republic of Korea	Male	Age-standardized	Periodontal diseases	Rate	1994	61.61	128.06	24.01
293	DALYs (Disability-Adjusted Life Years)	Republic of Korea	Female	Age-standardized	Periodontal diseases	Rate	1994	39.65	77.52	15.80
294	DALYs (Disability-Adjusted Life Years)	Republic of Korea	Both	Age-standardized	Periodontal diseases	Rate	1994	50.04	101.51	18.53
295	DALYs (Disability-Adjusted Life Years)	Federal Republic of Germany	Male	Age-standardized	Periodontal diseases	Rate	1997	140.01	293.78	55.75
296	DALYs (Disability-Adjusted Life Years)	Federal Republic of Germany	Female	Age-standardized	Periodontal diseases	Rate	1997	128.30	270.88	52.52
297	DALYs (Disability-Adjusted Life Years)	Federal Republic of Germany	Both	Age-standardized	Periodontal diseases	Rate	1997	134.01	282.45	53.78
298	DALYs (Disability-Adjusted Life Years)	Republic of South Africa	Male	Age-standardized	Periodontal diseases	Rate	1996	51.36	109.92	19.09
299	DALYs (Disability-Adjusted Life Years)	Republic of South Africa	Female	Age-standardized	Periodontal diseases	Rate	1996	49.70	105.70	18.73

300	DALYs (Disability-Adjusted Life Years)	Republic of South Africa	Both	Age-standardized	Periodontal diseases	Rate	1996	50.48	107.69	18.87
301	DALYs (Disability-Adjusted Life Years)	Republic of Italy	Male	Age-standardized	Periodontal diseases	Rate	1995	62.32	136.91	24.07
302	DALYs (Disability-Adjusted Life Years)	Republic of Italy	Female	Age-standardized	Periodontal diseases	Rate	1995	70.95	149.95	27.79
303	DALYs (Disability-Adjusted Life Years)	Republic of Italy	Both	Age-standardized	Periodontal diseases	Rate	1995	66.72	143.77	26.07
304	DALYs (Disability-Adjusted Life Years)	People's Republic of China	Male	Age-standardized	Periodontal diseases	Rate	1995	59.95	123.32	22.86
305	DALYs (Disability-Adjusted Life Years)	People's Republic of China	Female	Age-standardized	Periodontal diseases	Rate	1995	55.31	114.30	20.72
306	DALYs (Disability-Adjusted Life Years)	People's Republic of China	Both	Age-standardized	Periodontal diseases	Rate	1995	57.64	118.82	21.82
307	DALYs (Disability-Adjusted Life Years)	United States of America	Male	Age-standardized	Periodontal diseases	Rate	1995	72.29	151.05	28.70
308	DALYs (Disability-Adjusted Life Years)	United States of America	Female	Age-standardized	Periodontal diseases	Rate	1995	55.45	117.99	20.98
309	DALYs (Disability-Adjusted Life Years)	United States of America	Both	Age-standardized	Periodontal diseases	Rate	1995	63.60	134.91	24.71
310	DALYs (Disability-Adjusted Life Years)	Russian Federation	Male	Age-standardized	Periodontal diseases	Rate	1995	76.07	159.12	30.51
311	DALYs (Disability-Adjusted Life Years)	Russian Federation	Female	Age-standardized	Periodontal diseases	Rate	1995	75.41	157.30	29.99
312	DALYs (Disability-Adjusted Life Years)	Russian Federation	Both	Age-standardized	Periodontal diseases	Rate	1995	75.55	157.61	30.18
313	DALYs (Disability-Adjusted Life Years)	Republic of Indonesia	Male	Age-standardized	Periodontal diseases	Rate	1996	83.69	176.82	33.36
314	DALYs (Disability-Adjusted Life	Republic of Indonesia	Female	Age-standardized	Periodontal diseases	Rate	1996	91.30	194.60	36.67

	Years)									
315	DALYs (Disability-Adjusted Life Years)	Republic of Indonesia	Both	Age-standardized	Periodontal diseases	Rate	1996	87.46	185.65	35.13
316	DALYs (Disability-Adjusted Life Years)	Australia	Male	Age-standardized	Periodontal diseases	Rate	1992	54.74	110.96	21.70
317	DALYs (Disability-Adjusted Life Years)	Australia	Female	Age-standardized	Periodontal diseases	Rate	1992	40.16	83.82	16.16
318	DALYs (Disability-Adjusted Life Years)	Australia	Both	Age-standardized	Periodontal diseases	Rate	1992	47.30	97.62	18.69
319	DALYs (Disability-Adjusted Life Years)	United States of America	Male	Age-standardized	Periodontal diseases	Rate	1993	72.25	150.87	28.47
320	DALYs (Disability-Adjusted Life Years)	United States of America	Female	Age-standardized	Periodontal diseases	Rate	1993	55.19	117.78	20.82
321	DALYs (Disability-Adjusted Life Years)	United States of America	Both	Age-standardized	Periodontal diseases	Rate	1993	63.44	134.66	24.51
322	DALYs (Disability-Adjusted Life Years)	Canada	Male	Age-standardized	Periodontal diseases	Rate	1995	97.72	201.33	37.73
323	DALYs (Disability-Adjusted Life Years)	Canada	Female	Age-standardized	Periodontal diseases	Rate	1995	76.96	157.62	29.75
324	DALYs (Disability-Adjusted Life Years)	Canada	Both	Age-standardized	Periodontal diseases	Rate	1995	87.15	179.57	33.78
325	DALYs (Disability-Adjusted Life Years)	Republic of South Africa	Male	Age-standardized	Periodontal diseases	Rate	1998	50.46	108.61	18.84
326	DALYs (Disability-Adjusted Life Years)	Republic of South Africa	Female	Age-standardized	Periodontal diseases	Rate	1998	44.10	93.71	16.77
327	DALYs (Disability-Adjusted Life Years)	Republic of South Africa	Both	Age-standardized	Periodontal diseases	Rate	1998	46.96	101.04	17.83
328	DALYs (Disability-Adjusted Life Years)	Japan	Male	Age-standardized	Periodontal diseases	Rate	1995	74.95	156.27	29.67
329	DALYs	Japan	Female	Age-standardized	Periodontal	Rate	1995	56.26	117.49	21.07

	(Disability-Adjusted Life Years)				diseases					
330	DALYs (Disability-Adjusted Life Years)	Japan	Both	Age-standardized	Periodontal diseases	Rate	1995	65.50	139.92	25.57
331	DALYs (Disability-Adjusted Life Years)	Republic of Indonesia	Male	Age-standardized	Periodontal diseases	Rate	1995	83.48	176.63	33.22
332	DALYs (Disability-Adjusted Life Years)	Republic of Indonesia	Female	Age-standardized	Periodontal diseases	Rate	1995	91.00	194.56	36.54
333	DALYs (Disability-Adjusted Life Years)	Republic of Indonesia	Both	Age-standardized	Periodontal diseases	Rate	1995	87.21	185.54	34.96
334	DALYs (Disability-Adjusted Life Years)	French Republic	Male	Age-standardized	Periodontal diseases	Rate	1995	33.59	71.23	12.39
335	DALYs (Disability-Adjusted Life Years)	French Republic	Female	Age-standardized	Periodontal diseases	Rate	1995	33.52	70.54	12.98
336	DALYs (Disability-Adjusted Life Years)	French Republic	Both	Age-standardized	Periodontal diseases	Rate	1995	33.52	71.24	12.69
337	DALYs (Disability-Adjusted Life Years)	Federative Republic of Brazil	Male	Age-standardized	Periodontal diseases	Rate	1998	87.73	184.12	35.43
338	DALYs (Disability-Adjusted Life Years)	Federative Republic of Brazil	Female	Age-standardized	Periodontal diseases	Rate	1998	77.24	165.95	31.02
339	DALYs (Disability-Adjusted Life Years)	Federative Republic of Brazil	Both	Age-standardized	Periodontal diseases	Rate	1998	82.26	174.70	33.16
340	DALYs (Disability-Adjusted Life Years)	United Kingdom of Great Britain and Northern Ireland	Male	Age-standardized	Periodontal diseases	Rate	1996	39.61	88.19	14.68
341	DALYs (Disability-Adjusted Life Years)	United Kingdom of Great Britain and Northern Ireland	Female	Age-standardized	Periodontal diseases	Rate	1996	38.78	85.17	14.42
342	DALYs (Disability-Adjusted Life	United Kingdom of	Both	Age-standardized	Periodontal diseases	Rate	1996	39.16	86.56	14.53

	Years)	Great Britain and Northern Ireland								
343	DALYs (Disability-Adjusted Life Years)	Federal Republic of Germany	Male	Age-standardized	Periodontal diseases	Rate	1999	139.71	291.57	55.25
344	DALYs (Disability-Adjusted Life Years)	Federal Republic of Germany	Female	Age-standardized	Periodontal diseases	Rate	1999	128.02	265.43	51.44
345	DALYs (Disability-Adjusted Life Years)	Federal Republic of Germany	Both	Age-standardized	Periodontal diseases	Rate	1999	133.73	277.54	52.91
346	DALYs (Disability-Adjusted Life Years)	Kingdom of Saudi Arabia	Male	Age-standardized	Periodontal diseases	Rate	1999	54.50	109.21	21.97
347	DALYs (Disability-Adjusted Life Years)	Kingdom of Saudi Arabia	Female	Age-standardized	Periodontal diseases	Rate	1999	49.77	98.66	19.85
348	DALYs (Disability-Adjusted Life Years)	Kingdom of Saudi Arabia	Both	Age-standardized	Periodontal diseases	Rate	1999	52.74	104.92	21.46
349	DALYs (Disability-Adjusted Life Years)	Republic of India	Male	Age-standardized	Periodontal diseases	Rate	1999	109.01	221.35	43.82
350	DALYs (Disability-Adjusted Life Years)	Republic of India	Female	Age-standardized	Periodontal diseases	Rate	1999	106.98	215.86	43.22
351	DALYs (Disability-Adjusted Life Years)	Republic of India	Both	Age-standardized	Periodontal diseases	Rate	1999	108.02	218.68	43.52
352	DALYs (Disability-Adjusted Life Years)	United Mexican States	Male	Age-standardized	Periodontal diseases	Rate	1996	100.70	205.80	40.71
353	DALYs (Disability-Adjusted Life Years)	United Mexican States	Female	Age-standardized	Periodontal diseases	Rate	1996	89.32	188.00	35.73
354	DALYs (Disability-Adjusted Life Years)	United Mexican States	Both	Age-standardized	Periodontal diseases	Rate	1996	94.78	196.52	38.12
355	DALYs (Disability-Adjusted Life Years)	United Mexican States	Male	Age-standardized	Periodontal diseases	Rate	1995	100.70	205.94	40.63
356	DALYs (Disability-Adjusted Life	United Mexican States	Female	Age-standardized	Periodontal diseases	Rate	1995	89.04	187.79	35.59

	Years)										
357	DALYs (Disability-Adjusted Life Years)	United Mexican States	Both	Age-standardized	Periodontal diseases	Rate	1995	94.63	196.51	38.01
358	DALYs (Disability-Adjusted Life Years)	Republic of Korea	Male	Age-standardized	Periodontal diseases	Rate	1999	57.94	115.01	23.05
359	DALYs (Disability-Adjusted Life Years)	Republic of Korea	Female	Age-standardized	Periodontal diseases	Rate	1999	40.89	83.78	16.07
360	DALYs (Disability-Adjusted Life Years)	Republic of Korea	Both	Age-standardized	Periodontal diseases	Rate	1999	49.02	96.59	19.45
361	DALYs (Disability-Adjusted Life Years)	Australia	Male	Age-standardized	Periodontal diseases	Rate	1996	51.12	105.09	20.46
362	DALYs (Disability-Adjusted Life Years)	Australia	Female	Age-standardized	Periodontal diseases	Rate	1996	41.52	84.01	16.14
363	DALYs (Disability-Adjusted Life Years)	Australia	Both	Age-standardized	Periodontal diseases	Rate	1996	46.22	94.35	17.85
364	DALYs (Disability-Adjusted Life Years)	Republic of India	Male	Age-standardized	Periodontal diseases	Rate	1998	108.54	220.11	43.60
365	DALYs (Disability-Adjusted Life Years)	Republic of India	Female	Age-standardized	Periodontal diseases	Rate	1998	106.51	214.72	43.08
366	DALYs (Disability-Adjusted Life Years)	Republic of India	Both	Age-standardized	Periodontal diseases	Rate	1998	107.55	217.50	43.34
367	DALYs (Disability-Adjusted Life Years)	Argentine Republic	Male	Age-standardized	Periodontal diseases	Rate	1996	83.65	176.09	32.32
368	DALYs (Disability-Adjusted Life Years)	Argentine Republic	Female	Age-standardized	Periodontal diseases	Rate	1996	70.49	149.32	27.02
369	DALYs (Disability-Adjusted Life Years)	Argentine Republic	Both	Age-standardized	Periodontal diseases	Rate	1996	76.69	162.25	29.43
370	DALYs (Disability-Adjusted Life Years)	Republic of Korea	Male	Age-standardized	Periodontal diseases	Rate	1998	59.02	115.76	23.15
371	DALYs	Republic of	Female	Age-standardized	Periodontal	Rate	1998	40.57	83.89	15.76

	(Disability-Adjusted Life Years)	Korea			diseases					
372	DALYs (Disability-Adjusted Life Years)	Republic of Korea	Both	Age-standardized	Periodontal diseases	Rate	1998	49.36	94.84	19.60
373	DALYs (Disability-Adjusted Life Years)	Federal Republic of Germany	Male	Age-standardized	Periodontal diseases	Rate	1998	139.93	291.85	55.92
374	DALYs (Disability-Adjusted Life Years)	Federal Republic of Germany	Female	Age-standardized	Periodontal diseases	Rate	1998	128.22	268.87	52.11
375	DALYs (Disability-Adjusted Life Years)	Federal Republic of Germany	Both	Age-standardized	Periodontal diseases	Rate	1998	133.93	282.34	54.06
376	DALYs (Disability-Adjusted Life Years)	Republic of South Africa	Male	Age-standardized	Periodontal diseases	Rate	1997	50.95	109.20	19.02
377	DALYs (Disability-Adjusted Life Years)	Republic of South Africa	Female	Age-standardized	Periodontal diseases	Rate	1997	47.17	101.47	17.98
378	DALYs (Disability-Adjusted Life Years)	Republic of South Africa	Both	Age-standardized	Periodontal diseases	Rate	1997	48.89	104.47	18.45
379	DALYs (Disability-Adjusted Life Years)	People's Republic of China	Male	Age-standardized	Periodontal diseases	Rate	1996	59.23	121.51	22.54
380	DALYs (Disability-Adjusted Life Years)	People's Republic of China	Female	Age-standardized	Periodontal diseases	Rate	1996	54.84	112.73	20.57
381	DALYs (Disability-Adjusted Life Years)	People's Republic of China	Both	Age-standardized	Periodontal diseases	Rate	1996	57.06	117.15	21.56
382	DALYs (Disability-Adjusted Life Years)	Kingdom of Saudi Arabia	Male	Age-standardized	Periodontal diseases	Rate	1998	55.52	109.33	21.67
383	DALYs (Disability-Adjusted Life Years)	Kingdom of Saudi Arabia	Female	Age-standardized	Periodontal diseases	Rate	1998	50.94	96.06	20.01
384	DALYs (Disability-Adjusted Life Years)	Kingdom of Saudi Arabia	Both	Age-standardized	Periodontal diseases	Rate	1998	53.84	104.33	21.06
385	DALYs (Disability-Adjusted Life Years)	Federative Republic of Brazil	Male	Age-standardized	Periodontal diseases	Rate	1997	81.34	172.00	33.08

386	DALYs (Disability-Adjusted Life Years)	Federative Republic of Brazil	Female	Age-standardized	Periodontal diseases	Rate	1997	70.38	150.17	28.57
387	DALYs (Disability-Adjusted Life Years)	Federative Republic of Brazil	Both	Age-standardized	Periodontal diseases	Rate	1997	75.62	160.63	30.76
388	DALYs (Disability-Adjusted Life Years)	Republic of Italy	Male	Age-standardized	Periodontal diseases	Rate	1996	62.20	135.72	23.97
389	DALYs (Disability-Adjusted Life Years)	Republic of Italy	Female	Age-standardized	Periodontal diseases	Rate	1996	70.90	149.45	27.80
390	DALYs (Disability-Adjusted Life Years)	Republic of Italy	Both	Age-standardized	Periodontal diseases	Rate	1996	66.63	143.35	26.03
391	DALYs (Disability-Adjusted Life Years)	Russian Federation	Male	Age-standardized	Periodontal diseases	Rate	1996	75.69	158.24	30.26
392	DALYs (Disability-Adjusted Life Years)	Russian Federation	Female	Age-standardized	Periodontal diseases	Rate	1996	75.11	156.65	29.86
393	DALYs (Disability-Adjusted Life Years)	Russian Federation	Both	Age-standardized	Periodontal diseases	Rate	1996	75.21	156.86	30.00
394	DALYs (Disability-Adjusted Life Years)	Republic of Turkey	Male	Age-standardized	Periodontal diseases	Rate	1995	42.01	95.55	15.94
395	DALYs (Disability-Adjusted Life Years)	Republic of Turkey	Female	Age-standardized	Periodontal diseases	Rate	1995	41.98	95.05	15.70
396	DALYs (Disability-Adjusted Life Years)	Republic of Turkey	Both	Age-standardized	Periodontal diseases	Rate	1995	42.08	95.29	15.85
397	DALYs (Disability-Adjusted Life Years)	United Kingdom of Great Britain and Northern Ireland	Male	Age-standardized	Periodontal diseases	Rate	1995	41.00	91.19	15.22
398	DALYs (Disability-Adjusted Life Years)	United Kingdom of Great Britain and Northern Ireland	Female	Age-standardized	Periodontal diseases	Rate	1995	40.06	88.18	14.92
399	DALYs	United	Both	Age-standardized	Periodontal	Rate	1995	40.50	89.57	15.06

	(Disability-Adjusted Life Years)	Kingdom of Great Britain and Northern Ireland			diseases					
400	DALYs (Disability-Adjusted Life Years)	Republic of Turkey	Male	Age-standardized	Periodontal diseases	Rate	1996	45.95	102.51	17.05
401	DALYs (Disability-Adjusted Life Years)	Republic of Turkey	Female	Age-standardized	Periodontal diseases	Rate	1996	45.47	99.46	17.04
402	DALYs (Disability-Adjusted Life Years)	Republic of Turkey	Both	Age-standardized	Periodontal diseases	Rate	1996	45.79	100.41	17.09
403	DALYs (Disability-Adjusted Life Years)	Republic of South Africa	Male	Age-standardized	Periodontal diseases	Rate	1999	50.02	108.26	18.73
404	DALYs (Disability-Adjusted Life Years)	Republic of South Africa	Female	Age-standardized	Periodontal diseases	Rate	1999	41.41	86.57	15.85
405	DALYs (Disability-Adjusted Life Years)	Republic of South Africa	Both	Age-standardized	Periodontal diseases	Rate	1999	45.27	98.37	17.31
406	DALYs (Disability-Adjusted Life Years)	Federative Republic of Brazil	Male	Age-standardized	Periodontal diseases	Rate	1999	93.04	192.45	37.41
407	DALYs (Disability-Adjusted Life Years)	Federative Republic of Brazil	Female	Age-standardized	Periodontal diseases	Rate	1999	82.96	176.99	33.10
408	DALYs (Disability-Adjusted Life Years)	Federative Republic of Brazil	Both	Age-standardized	Periodontal diseases	Rate	1999	87.80	184.37	35.18
409	DALYs (Disability-Adjusted Life Years)	Australia	Male	Age-standardized	Periodontal diseases	Rate	1995	49.42	102.10	19.73
410	DALYs (Disability-Adjusted Life Years)	Australia	Female	Age-standardized	Periodontal diseases	Rate	1995	40.76	81.99	15.88
411	DALYs (Disability-Adjusted Life Years)	Australia	Both	Age-standardized	Periodontal diseases	Rate	1995	45.01	91.89	17.55
412	DALYs (Disability-Adjusted Life Years)	European Union	Male	Age-standardized	Periodontal diseases	Rate	1995	79.16	161.54	30.69
413	DALYs	European	Female	Age-standardized	Periodontal	Rate	1995	73.52	153.27	28.70

	(Disability-Adjusted Life Years)	Union			diseases					
414	DALYs (Disability-Adjusted Life Years)	European Union	Both	Age-standardized	Periodontal diseases	Rate	1995	76.25	157.24	29.59
415	DALYs (Disability-Adjusted Life Years)	Argentine Republic	Male	Age-standardized	Periodontal diseases	Rate	1995	84.98	187.97	34.34
416	DALYs (Disability-Adjusted Life Years)	Argentine Republic	Female	Age-standardized	Periodontal diseases	Rate	1995	71.98	161.18	28.77
417	DALYs (Disability-Adjusted Life Years)	Argentine Republic	Both	Age-standardized	Periodontal diseases	Rate	1995	78.11	174.07	31.39
418	DALYs (Disability-Adjusted Life Years)	Federative Republic of Brazil	Male	Age-standardized	Periodontal diseases	Rate	2000	95.22	195.69	38.18
419	DALYs (Disability-Adjusted Life Years)	Federative Republic of Brazil	Female	Age-standardized	Periodontal diseases	Rate	2000	85.31	181.75	33.90
420	DALYs (Disability-Adjusted Life Years)	Federative Republic of Brazil	Both	Age-standardized	Periodontal diseases	Rate	2000	90.06	188.43	35.97
421	DALYs (Disability-Adjusted Life Years)	Canada	Male	Age-standardized	Periodontal diseases	Rate	1996	97.22	202.00	37.73
422	DALYs (Disability-Adjusted Life Years)	Canada	Female	Age-standardized	Periodontal diseases	Rate	1996	77.28	159.12	30.36
423	DALYs (Disability-Adjusted Life Years)	Canada	Both	Age-standardized	Periodontal diseases	Rate	1996	87.06	180.12	34.22
424	DALYs (Disability-Adjusted Life Years)	French Republic	Male	Age-standardized	Periodontal diseases	Rate	1996	34.67	73.31	13.09
425	DALYs (Disability-Adjusted Life Years)	French Republic	Female	Age-standardized	Periodontal diseases	Rate	1996	34.52	72.57	13.65
426	DALYs (Disability-Adjusted Life Years)	French Republic	Both	Age-standardized	Periodontal diseases	Rate	1996	34.56	72.91	13.37
427	DALYs (Disability-Adjusted Life Years)	Republic of Italy	Male	Age-standardized	Periodontal diseases	Rate	1994	62.29	136.84	24.06

428	DALYs (Disability-Adjusted Life Years)	Republic of Italy	Female	Age-standardized	Periodontal diseases	Rate	1994	70.92	149.92	27.80
429	DALYs (Disability-Adjusted Life Years)	Republic of Italy	Both	Age-standardized	Periodontal diseases	Rate	1994	66.69	144.02	26.02
430	DALYs (Disability-Adjusted Life Years)	Republic of South Africa	Male	Age-standardized	Periodontal diseases	Rate	2000	49.74	107.33	18.66
431	DALYs (Disability-Adjusted Life Years)	Republic of South Africa	Female	Age-standardized	Periodontal diseases	Rate	2000	40.13	82.55	15.43
432	DALYs (Disability-Adjusted Life Years)	Republic of South Africa	Both	Age-standardized	Periodontal diseases	Rate	2000	44.42	95.02	17.04
433	DALYs (Disability-Adjusted Life Years)	United States of America	Male	Age-standardized	Periodontal diseases	Rate	1994	72.35	151.31	28.60
434	DALYs (Disability-Adjusted Life Years)	United States of America	Female	Age-standardized	Periodontal diseases	Rate	1994	55.33	117.78	20.93
435	DALYs (Disability-Adjusted Life Years)	United States of America	Both	Age-standardized	Periodontal diseases	Rate	1994	63.56	135.00	24.63
436	DALYs (Disability-Adjusted Life Years)	United States of America	Male	Age-standardized	Periodontal diseases	Rate	1996	71.80	150.39	28.43
437	DALYs (Disability-Adjusted Life Years)	United States of America	Female	Age-standardized	Periodontal diseases	Rate	1996	55.84	118.90	21.14
438	DALYs (Disability-Adjusted Life Years)	United States of America	Both	Age-standardized	Periodontal diseases	Rate	1996	63.58	134.77	24.67
439	DALYs (Disability-Adjusted Life Years)	Federal Republic of Germany	Male	Age-standardized	Periodontal diseases	Rate	2000	139.30	282.67	54.34
440	DALYs (Disability-Adjusted Life Years)	Federal Republic of Germany	Female	Age-standardized	Periodontal diseases	Rate	2000	127.74	261.06	51.05
441	DALYs (Disability-Adjusted Life Years)	Federal Republic of Germany	Both	Age-standardized	Periodontal diseases	Rate	2000	133.39	271.49	52.07
442	DALYs (Disability-Adjusted Life	People's Republic of	Male	Age-standardized	Periodontal diseases	Rate	1994	61.31	127.06	23.51

	Years)	China								
443	DALYs (Disability-Adjusted Life Years)	People's Republic of China	Female	Age-standardized	Periodontal diseases	Rate	1994	56.49	117.36	21.44
444	DALYs (Disability-Adjusted Life Years)	People's Republic of China	Both	Age-standardized	Periodontal diseases	Rate	1994	58.91	122.26	22.48
445	DALYs (Disability-Adjusted Life Years)	European Union	Male	Age-standardized	Periodontal diseases	Rate	1996	78.61	162.00	30.62
446	DALYs (Disability-Adjusted Life Years)	European Union	Female	Age-standardized	Periodontal diseases	Rate	1996	73.08	153.85	28.90
447	DALYs (Disability-Adjusted Life Years)	European Union	Both	Age-standardized	Periodontal diseases	Rate	1996	75.76	157.87	29.71
448	DALYs (Disability-Adjusted Life Years)	Japan	Male	Age-standardized	Periodontal diseases	Rate	1996	74.30	155.64	29.23
449	DALYs (Disability-Adjusted Life Years)	Japan	Female	Age-standardized	Periodontal diseases	Rate	1996	56.23	115.37	20.89
450	DALYs (Disability-Adjusted Life Years)	Japan	Both	Age-standardized	Periodontal diseases	Rate	1996	65.20	138.42	25.08
451	DALYs (Disability-Adjusted Life Years)	Kingdom of Saudi Arabia	Male	Age-standardized	Periodontal diseases	Rate	2000	54.03	114.44	21.11
452	DALYs (Disability-Adjusted Life Years)	Kingdom of Saudi Arabia	Female	Age-standardized	Periodontal diseases	Rate	2000	49.22	102.24	18.75
453	DALYs (Disability-Adjusted Life Years)	Kingdom of Saudi Arabia	Both	Age-standardized	Periodontal diseases	Rate	2000	52.22	110.07	20.75
454	DALYs (Disability-Adjusted Life Years)	Republic of India	Male	Age-standardized	Periodontal diseases	Rate	2000	109.35	222.11	43.99
455	DALYs (Disability-Adjusted Life Years)	Republic of India	Female	Age-standardized	Periodontal diseases	Rate	2000	107.31	216.86	43.39
456	DALYs (Disability-Adjusted Life Years)	Republic of India	Both	Age-standardized	Periodontal diseases	Rate	2000	108.35	219.55	43.69
457	DALYs	French	Male	Age-standardized	Periodontal	Rate	1994	34.01	68.71	13.47

	(Disability-Adjusted Life Years)	Republic			diseases					
458	DALYs (Disability-Adjusted Life Years)	French Republic	Female	Age-standardized	Periodontal diseases	Rate	1994	33.86	72.02	13.17
459	DALYs (Disability-Adjusted Life Years)	French Republic	Both	Age-standardized	Periodontal diseases	Rate	1994	33.91	69.61	13.31
460	DALYs (Disability-Adjusted Life Years)	Canada	Male	Age-standardized	Periodontal diseases	Rate	1994	98.02	200.02	38.11
461	DALYs (Disability-Adjusted Life Years)	Canada	Female	Age-standardized	Periodontal diseases	Rate	1994	77.53	156.04	30.03
462	DALYs (Disability-Adjusted Life Years)	Canada	Both	Age-standardized	Periodontal diseases	Rate	1994	87.59	178.39	34.16
463	DALYs (Disability-Adjusted Life Years)	Japan	Male	Age-standardized	Periodontal diseases	Rate	1994	74.74	156.41	29.59
464	DALYs (Disability-Adjusted Life Years)	Japan	Female	Age-standardized	Periodontal diseases	Rate	1994	55.84	118.01	21.06
465	DALYs (Disability-Adjusted Life Years)	Japan	Both	Age-standardized	Periodontal diseases	Rate	1994	65.18	139.49	25.48
466	DALYs (Disability-Adjusted Life Years)	Republic of Korea	Male	Age-standardized	Periodontal diseases	Rate	2000	57.50	118.22	22.08
467	DALYs (Disability-Adjusted Life Years)	Republic of Korea	Female	Age-standardized	Periodontal diseases	Rate	2000	41.21	84.73	15.98
468	DALYs (Disability-Adjusted Life Years)	Republic of Korea	Both	Age-standardized	Periodontal diseases	Rate	2000	49.00	99.86	18.88
469	DALYs (Disability-Adjusted Life Years)	Republic of Indonesia	Male	Age-standardized	Periodontal diseases	Rate	1994	83.27	175.83	33.14
470	DALYs (Disability-Adjusted Life Years)	Republic of Indonesia	Female	Age-standardized	Periodontal diseases	Rate	1994	90.77	193.98	36.47
471	DALYs (Disability-Adjusted Life Years)	Republic of Indonesia	Both	Age-standardized	Periodontal diseases	Rate	1994	86.99	184.87	34.90

472	DALYs (Disability-Adjusted Life Years)	Federal Republic of Germany	Male	Age-standardized	Periodontal diseases	Rate	2001	134.17	273.85	51.95
473	DALYs (Disability-Adjusted Life Years)	Federal Republic of Germany	Female	Age-standardized	Periodontal diseases	Rate	2001	122.36	250.87	48.52
474	DALYs (Disability-Adjusted Life Years)	Federal Republic of Germany	Both	Age-standardized	Periodontal diseases	Rate	2001	128.13	261.22	49.79
475	DALYs (Disability-Adjusted Life Years)	Republic of Korea	Male	Age-standardized	Periodontal diseases	Rate	2001	57.86	118.86	22.35
476	DALYs (Disability-Adjusted Life Years)	Republic of Korea	Female	Age-standardized	Periodontal diseases	Rate	2001	41.63	85.66	16.00
477	DALYs (Disability-Adjusted Life Years)	Republic of Korea	Both	Age-standardized	Periodontal diseases	Rate	2001	49.40	100.73	19.04
478	DALYs (Disability-Adjusted Life Years)	Republic of Indonesia	Male	Age-standardized	Periodontal diseases	Rate	1997	83.88	176.98	33.39
479	DALYs (Disability-Adjusted Life Years)	Republic of Indonesia	Female	Age-standardized	Periodontal diseases	Rate	1997	91.73	195.58	36.92
480	DALYs (Disability-Adjusted Life Years)	Republic of Indonesia	Both	Age-standardized	Periodontal diseases	Rate	1997	87.77	186.21	35.28
481	DALYs (Disability-Adjusted Life Years)	United Mexican States	Male	Age-standardized	Periodontal diseases	Rate	1994	100.63	206.24	40.60
482	DALYs (Disability-Adjusted Life Years)	United Mexican States	Female	Age-standardized	Periodontal diseases	Rate	1994	88.98	187.96	35.57
483	DALYs (Disability-Adjusted Life Years)	United Mexican States	Both	Age-standardized	Periodontal diseases	Rate	1994	94.57	196.75	37.99
484	DALYs (Disability-Adjusted Life Years)	Kingdom of Saudi Arabia	Male	Age-standardized	Periodontal diseases	Rate	2001	54.03	114.40	21.37
485	DALYs (Disability-Adjusted Life Years)	Kingdom of Saudi Arabia	Female	Age-standardized	Periodontal diseases	Rate	2001	49.25	102.88	18.94
486	DALYs (Disability-Adjusted Life	Kingdom of Saudi Arabia	Both	Age-standardized	Periodontal diseases	Rate	2001	52.23	110.31	20.71

	Years)									
487	DALYs (Disability-Adjusted Life Years)	Republic of India	Male	Age-standardized	Periodontal diseases	Rate	2001	109.57	222.30	44.01
488	DALYs (Disability-Adjusted Life Years)	Republic of India	Female	Age-standardized	Periodontal diseases	Rate	2001	107.59	217.61	43.38
489	DALYs (Disability-Adjusted Life Years)	Republic of India	Both	Age-standardized	Periodontal diseases	Rate	2001	108.60	220.00	43.70
490	DALYs (Disability-Adjusted Life Years)	United Kingdom of Great Britain and Northern Ireland	Male	Age-standardized	Periodontal diseases	Rate	1994	41.07	91.48	15.23
491	DALYs (Disability-Adjusted Life Years)	United Kingdom of Great Britain and Northern Ireland	Female	Age-standardized	Periodontal diseases	Rate	1994	40.15	88.43	14.98
492	DALYs (Disability-Adjusted Life Years)	United Kingdom of Great Britain and Northern Ireland	Both	Age-standardized	Periodontal diseases	Rate	1994	40.58	89.76	15.10
493	DALYs (Disability-Adjusted Life Years)	Australia	Male	Age-standardized	Periodontal diseases	Rate	1994	50.01	103.23	19.86
494	DALYs (Disability-Adjusted Life Years)	Australia	Female	Age-standardized	Periodontal diseases	Rate	1994	40.61	82.81	15.97
495	DALYs (Disability-Adjusted Life Years)	Australia	Both	Age-standardized	Periodontal diseases	Rate	1994	45.22	92.88	17.72
496	DALYs (Disability-Adjusted Life Years)	United Mexican States	Male	Age-standardized	Periodontal diseases	Rate	1997	100.64	205.10	40.68
497	DALYs (Disability-Adjusted Life Years)	United Mexican States	Female	Age-standardized	Periodontal diseases	Rate	1997	89.94	188.99	35.94
498	DALYs (Disability-Adjusted Life Years)	United Mexican States	Both	Age-standardized	Periodontal diseases	Rate	1997	95.07	196.60	38.21
499	DALYs	Federative	Male	Age-standardized	Periodontal	Rate	2001	92.81	192.30	37.31

	(Disability-Adjusted Life Years)	Republic of Brazil			diseases					
500	DALYs (Disability-Adjusted Life Years)	Federative Republic of Brazil	Female	Age-standardized	Periodontal diseases	Rate	2001	82.46	176.70	32.83
501	DALYs (Disability-Adjusted Life Years)	Federative Republic of Brazil	Both	Age-standardized	Periodontal diseases	Rate	2001	87.42	183.94	35.01
502	DALYs (Disability-Adjusted Life Years)	United Kingdom of Great Britain and Northern Ireland	Male	Age-standardized	Periodontal diseases	Rate	1997	36.44	80.99	13.53
503	DALYs (Disability-Adjusted Life Years)	United Kingdom of Great Britain and Northern Ireland	Female	Age-standardized	Periodontal diseases	Rate	1997	35.85	77.88	13.40
504	DALYs (Disability-Adjusted Life Years)	United Kingdom of Great Britain and Northern Ireland	Both	Age-standardized	Periodontal diseases	Rate	1997	36.12	79.68	13.46
505	DALYs (Disability-Adjusted Life Years)	Republic of South Africa	Male	Age-standardized	Periodontal diseases	Rate	2001	48.50	105.21	18.61
506	DALYs (Disability-Adjusted Life Years)	Republic of South Africa	Female	Age-standardized	Periodontal diseases	Rate	2001	39.47	79.90	15.19
507	DALYs (Disability-Adjusted Life Years)	Republic of South Africa	Both	Age-standardized	Periodontal diseases	Rate	2001	43.49	90.66	16.48
508	DALYs (Disability-Adjusted Life Years)	Argentine Republic	Male	Age-standardized	Periodontal diseases	Rate	1994	84.87	186.05	34.44
509	DALYs (Disability-Adjusted Life Years)	Argentine Republic	Female	Age-standardized	Periodontal diseases	Rate	1994	71.89	160.11	28.51
510	DALYs (Disability-Adjusted Life Years)	Argentine Republic	Both	Age-standardized	Periodontal diseases	Rate	1994	78.02	172.57	31.33
511	DALYs (Disability-Adjusted Life Years)	Russian Federation	Male	Age-standardized	Periodontal diseases	Rate	1994	76.31	159.55	30.60

512	DALYs (Disability-Adjusted Life Years)	Russian Federation	Female	Age-standardized	Periodontal diseases	Rate	1994	75.71	157.91	30.28
513	DALYs (Disability-Adjusted Life Years)	Russian Federation	Both	Age-standardized	Periodontal diseases	Rate	1994	75.82	158.31	30.38
514	DALYs (Disability-Adjusted Life Years)	Republic of Turkey	Male	Age-standardized	Periodontal diseases	Rate	1994	41.59	94.44	15.62
515	DALYs (Disability-Adjusted Life Years)	Republic of Turkey	Female	Age-standardized	Periodontal diseases	Rate	1994	41.71	93.86	15.62
516	DALYs (Disability-Adjusted Life Years)	Republic of Turkey	Both	Age-standardized	Periodontal diseases	Rate	1994	41.74	94.33	15.65
517	DALYs (Disability-Adjusted Life Years)	Australia	Male	Age-standardized	Periodontal diseases	Rate	1997	55.04	112.39	22.02
518	DALYs (Disability-Adjusted Life Years)	Australia	Female	Age-standardized	Periodontal diseases	Rate	1997	43.05	88.21	16.70
519	DALYs (Disability-Adjusted Life Years)	Australia	Both	Age-standardized	Periodontal diseases	Rate	1997	48.91	100.25	18.82
520	DALYs (Disability-Adjusted Life Years)	European Union	Male	Age-standardized	Periodontal diseases	Rate	1994	78.90	161.01	30.65
521	DALYs (Disability-Adjusted Life Years)	European Union	Female	Age-standardized	Periodontal diseases	Rate	1994	73.26	152.70	28.50
522	DALYs (Disability-Adjusted Life Years)	European Union	Both	Age-standardized	Periodontal diseases	Rate	1994	75.99	156.52	29.44
523	DALYs (Disability-Adjusted Life Years)	People's Republic of China	Male	Age-standardized	Periodontal diseases	Rate	1998	58.25	118.98	22.12
524	DALYs (Disability-Adjusted Life Years)	People's Republic of China	Female	Age-standardized	Periodontal diseases	Rate	1998	54.75	111.75	20.49
525	DALYs (Disability-Adjusted Life Years)	People's Republic of China	Both	Age-standardized	Periodontal diseases	Rate	1998	56.55	115.46	21.32
526	DALYs (Disability-Adjusted Life	Argentine Republic	Male	Age-standardized	Periodontal diseases	Rate	1997	80.29	162.15	31.51

	Years)									
527	DALYs (Disability-Adjusted Life Years)	Argentine Republic	Female	Age-standardized	Periodontal diseases	Rate	1997	66.81	134.80	26.70
528	DALYs (Disability-Adjusted Life Years)	Argentine Republic	Both	Age-standardized	Periodontal diseases	Rate	1997	73.15	147.77	28.98
529	DALYs (Disability-Adjusted Life Years)	Republic of Italy	Male	Age-standardized	Periodontal diseases	Rate	1998	61.53	133.43	23.36
530	DALYs (Disability-Adjusted Life Years)	Republic of Italy	Female	Age-standardized	Periodontal diseases	Rate	1998	70.55	149.23	27.43
531	DALYs (Disability-Adjusted Life Years)	Republic of Italy	Both	Age-standardized	Periodontal diseases	Rate	1998	66.11	142.86	25.38
532	DALYs (Disability-Adjusted Life Years)	United States of America	Male	Age-standardized	Periodontal diseases	Rate	1998	69.21	145.16	27.24
533	DALYs (Disability-Adjusted Life Years)	United States of America	Female	Age-standardized	Periodontal diseases	Rate	1998	57.54	122.20	21.90
534	DALYs (Disability-Adjusted Life Years)	United States of America	Both	Age-standardized	Periodontal diseases	Rate	1998	63.24	133.50	24.50
535	DALYs (Disability-Adjusted Life Years)	People's Republic of China	Male	Age-standardized	Periodontal diseases	Rate	1997	58.65	120.13	22.33
536	DALYs (Disability-Adjusted Life Years)	People's Republic of China	Female	Age-standardized	Periodontal diseases	Rate	1997	54.69	111.97	20.52
537	DALYs (Disability-Adjusted Life Years)	People's Republic of China	Both	Age-standardized	Periodontal diseases	Rate	1997	56.71	116.13	21.44
538	DALYs (Disability-Adjusted Life Years)	Republic of Italy	Male	Age-standardized	Periodontal diseases	Rate	1997	61.91	134.86	23.77
539	DALYs (Disability-Adjusted Life Years)	Republic of Italy	Female	Age-standardized	Periodontal diseases	Rate	1997	70.74	149.33	27.63
540	DALYs (Disability-Adjusted Life Years)	Republic of Italy	Both	Age-standardized	Periodontal diseases	Rate	1997	66.40	143.18	25.73
541	DALYs	Russian	Male	Age-standardized	Periodontal	Rate	1997	75.09	156.88	29.94

	(Disability-Adjusted Life Years)	Federation			diseases					
542	DALYs (Disability-Adjusted Life Years)	Russian Federation	Female	Age-standardized	Periodontal diseases	Rate	1997	74.75	155.72	29.71
543	DALYs (Disability-Adjusted Life Years)	Russian Federation	Both	Age-standardized	Periodontal diseases	Rate	1997	74.74	155.72	29.77
544	DALYs (Disability-Adjusted Life Years)	Japan	Male	Age-standardized	Periodontal diseases	Rate	1998	70.52	148.26	27.11
545	DALYs (Disability-Adjusted Life Years)	Japan	Female	Age-standardized	Periodontal diseases	Rate	1998	55.71	111.67	20.84
546	DALYs (Disability-Adjusted Life Years)	Japan	Both	Age-standardized	Periodontal diseases	Rate	1998	63.15	130.45	23.78
547	DALYs (Disability-Adjusted Life Years)	European Union	Male	Age-standardized	Periodontal diseases	Rate	1997	77.20	160.71	30.44
548	DALYs (Disability-Adjusted Life Years)	European Union	Female	Age-standardized	Periodontal diseases	Rate	1997	72.02	150.22	28.71
549	DALYs (Disability-Adjusted Life Years)	European Union	Both	Age-standardized	Periodontal diseases	Rate	1997	74.52	156.96	29.49
550	DALYs (Disability-Adjusted Life Years)	Republic of Turkey	Male	Age-standardized	Periodontal diseases	Rate	1997	54.82	114.54	20.93
551	DALYs (Disability-Adjusted Life Years)	Republic of Turkey	Female	Age-standardized	Periodontal diseases	Rate	1997	53.42	112.98	20.27
552	DALYs (Disability-Adjusted Life Years)	Republic of Turkey	Both	Age-standardized	Periodontal diseases	Rate	1997	54.20	114.50	20.49
553	DALYs (Disability-Adjusted Life Years)	Republic of Indonesia	Male	Age-standardized	Periodontal diseases	Rate	1998	84.09	177.17	33.47
554	DALYs (Disability-Adjusted Life Years)	Republic of Indonesia	Female	Age-standardized	Periodontal diseases	Rate	1998	92.18	196.48	37.18
555	DALYs (Disability-Adjusted Life Years)	Republic of Indonesia	Both	Age-standardized	Periodontal diseases	Rate	1998	88.08	186.74	35.49

556	DALYs (Disability-Adjusted Life Years)	Federal Republic of Germany	Male	Age-standardized	Periodontal diseases	Rate	2002	122.91	253.19	47.23
557	DALYs (Disability-Adjusted Life Years)	Federal Republic of Germany	Female	Age-standardized	Periodontal diseases	Rate	2002	110.31	224.90	43.40
558	DALYs (Disability-Adjusted Life Years)	Federal Republic of Germany	Both	Age-standardized	Periodontal diseases	Rate	2002	116.46	238.71	45.13
559	DALYs (Disability-Adjusted Life Years)	Canada	Male	Age-standardized	Periodontal diseases	Rate	1998	95.44	192.13	37.61
560	DALYs (Disability-Adjusted Life Years)	Canada	Female	Age-standardized	Periodontal diseases	Rate	1998	79.91	160.63	31.06
561	DALYs (Disability-Adjusted Life Years)	Canada	Both	Age-standardized	Periodontal diseases	Rate	1998	87.52	176.11	34.40
562	DALYs (Disability-Adjusted Life Years)	Kingdom of Saudi Arabia	Male	Age-standardized	Periodontal diseases	Rate	2002	54.13	113.12	21.42
563	DALYs (Disability-Adjusted Life Years)	Kingdom of Saudi Arabia	Female	Age-standardized	Periodontal diseases	Rate	2002	49.30	103.46	19.14
564	DALYs (Disability-Adjusted Life Years)	Kingdom of Saudi Arabia	Both	Age-standardized	Periodontal diseases	Rate	2002	52.31	109.51	20.69
565	DALYs (Disability-Adjusted Life Years)	Republic of India	Male	Age-standardized	Periodontal diseases	Rate	2002	109.78	222.60	44.02
566	DALYs (Disability-Adjusted Life Years)	Republic of India	Female	Age-standardized	Periodontal diseases	Rate	2002	107.87	218.15	43.39
567	DALYs (Disability-Adjusted Life Years)	Republic of India	Both	Age-standardized	Periodontal diseases	Rate	2002	108.84	220.42	43.71
568	DALYs (Disability-Adjusted Life Years)	French Republic	Male	Age-standardized	Periodontal diseases	Rate	1998	40.13	83.31	15.34
569	DALYs (Disability-Adjusted Life Years)	French Republic	Female	Age-standardized	Periodontal diseases	Rate	1998	39.76	84.06	15.75
570	DALYs (Disability-Adjusted Life	French Republic	Both	Age-standardized	Periodontal diseases	Rate	1998	39.89	83.94	15.54

	Years)									
571	DALYs (Disability-Adjusted Life Years)	United States of America	Male	Age-standardized	Periodontal diseases	Rate	1997	70.70	148.18	27.89
572	DALYs (Disability-Adjusted Life Years)	United States of America	Female	Age-standardized	Periodontal diseases	Rate	1997	56.63	120.43	21.48
573	DALYs (Disability-Adjusted Life Years)	United States of America	Both	Age-standardized	Periodontal diseases	Rate	1997	63.48	134.22	24.59
574	DALYs (Disability-Adjusted Life Years)	Republic of Korea	Male	Age-standardized	Periodontal diseases	Rate	2002	58.60	119.52	22.53
575	DALYs (Disability-Adjusted Life Years)	Republic of Korea	Female	Age-standardized	Periodontal diseases	Rate	2002	42.18	87.66	16.07
576	DALYs (Disability-Adjusted Life Years)	Republic of Korea	Both	Age-standardized	Periodontal diseases	Rate	2002	50.06	102.30	19.18
577	DALYs (Disability-Adjusted Life Years)	Japan	Male	Age-standardized	Periodontal diseases	Rate	1997	72.70	153.95	28.32
578	DALYs (Disability-Adjusted Life Years)	Japan	Female	Age-standardized	Periodontal diseases	Rate	1997	56.04	113.81	20.71
579	DALYs (Disability-Adjusted Life Years)	Japan	Both	Age-standardized	Periodontal diseases	Rate	1997	64.35	135.06	24.50
580	DALYs (Disability-Adjusted Life Years)	Republic of South Africa	Male	Age-standardized	Periodontal diseases	Rate	2002	45.85	97.02	17.50
581	DALYs (Disability-Adjusted Life Years)	Republic of South Africa	Female	Age-standardized	Periodontal diseases	Rate	2002	38.33	76.69	14.86
582	DALYs (Disability-Adjusted Life Years)	Republic of South Africa	Both	Age-standardized	Periodontal diseases	Rate	2002	41.68	85.42	15.75
583	DALYs (Disability-Adjusted Life Years)	Federative Republic of Brazil	Male	Age-standardized	Periodontal diseases	Rate	2002	87.12	183.37	35.19
584	DALYs (Disability-Adjusted Life Years)	Federative Republic of Brazil	Female	Age-standardized	Periodontal diseases	Rate	2002	75.77	164.38	30.24
585	DALYs	Federative	Both	Age-standardized	Periodontal	Rate	2002	81.21	173.39	32.65

	(Disability-Adjusted Life Years)	Republic of Brazil			diseases					
586	DALYs (Disability-Adjusted Life Years)	French Republic	Male	Age-standardized	Periodontal diseases	Rate	1997	37.16	78.15	14.03
587	DALYs (Disability-Adjusted Life Years)	French Republic	Female	Age-standardized	Periodontal diseases	Rate	1997	36.89	77.62	14.57
588	DALYs (Disability-Adjusted Life Years)	French Republic	Both	Age-standardized	Periodontal diseases	Rate	1997	36.97	77.68	14.29
589	DALYs (Disability-Adjusted Life Years)	Canada	Male	Age-standardized	Periodontal diseases	Rate	1997	96.38	198.13	37.68
590	DALYs (Disability-Adjusted Life Years)	Canada	Female	Age-standardized	Periodontal diseases	Rate	1997	78.42	161.76	31.22
591	DALYs (Disability-Adjusted Life Years)	Canada	Both	Age-standardized	Periodontal diseases	Rate	1997	87.22	180.57	34.41
592	DALYs (Disability-Adjusted Life Years)	United Mexican States	Male	Age-standardized	Periodontal diseases	Rate	1999	100.49	204.04	40.68
593	DALYs (Disability-Adjusted Life Years)	United Mexican States	Female	Age-standardized	Periodontal diseases	Rate	1999	91.24	191.03	36.51
594	DALYs (Disability-Adjusted Life Years)	United Mexican States	Both	Age-standardized	Periodontal diseases	Rate	1999	95.65	197.11	38.48
595	DALYs (Disability-Adjusted Life Years)	United Kingdom of Great Britain and Northern Ireland	Male	Age-standardized	Periodontal diseases	Rate	1998	32.66	72.12	12.12
596	DALYs (Disability-Adjusted Life Years)	United Kingdom of Great Britain and Northern Ireland	Female	Age-standardized	Periodontal diseases	Rate	1998	32.38	69.48	12.06
597	DALYs (Disability-Adjusted Life Years)	United Kingdom of Great Britain and Northern Ireland	Both	Age-standardized	Periodontal diseases	Rate	1998	32.49	70.71	12.15

598	DALYs (Disability-Adjusted Life Years)	United Mexican States	Male	Age-standardized	Periodontal diseases	Rate	1998	100.56	204.49	40.66
599	DALYs (Disability-Adjusted Life Years)	United Mexican States	Female	Age-standardized	Periodontal diseases	Rate	1998	90.64	190.20	36.21
600	DALYs (Disability-Adjusted Life Years)	United Mexican States	Both	Age-standardized	Periodontal diseases	Rate	1998	95.38	196.88	38.33
601	DALYs (Disability-Adjusted Life Years)	Australia	Male	Age-standardized	Periodontal diseases	Rate	1998	59.85	119.68	23.16
602	DALYs (Disability-Adjusted Life Years)	Australia	Female	Age-standardized	Periodontal diseases	Rate	1998	44.99	89.98	17.30
603	DALYs (Disability-Adjusted Life Years)	Australia	Both	Age-standardized	Periodontal diseases	Rate	1998	52.23	104.22	20.24
604	DALYs (Disability-Adjusted Life Years)	United Kingdom of Great Britain and Northern Ireland	Male	Age-standardized	Periodontal diseases	Rate	1999	29.52	64.42	11.04
605	DALYs (Disability-Adjusted Life Years)	United Kingdom of Great Britain and Northern Ireland	Female	Age-standardized	Periodontal diseases	Rate	1999	29.51	62.90	11.03
606	DALYs (Disability-Adjusted Life Years)	United Kingdom of Great Britain and Northern Ireland	Both	Age-standardized	Periodontal diseases	Rate	1999	29.49	63.59	10.96
607	DALYs (Disability-Adjusted Life Years)	European Union	Male	Age-standardized	Periodontal diseases	Rate	1998	75.44	156.61	30.00
608	DALYs (Disability-Adjusted Life Years)	European Union	Female	Age-standardized	Periodontal diseases	Rate	1998	70.71	144.22	28.56
609	DALYs (Disability-Adjusted Life Years)	European Union	Both	Age-standardized	Periodontal diseases	Rate	1998	72.99	150.08	29.26
610	DALYs (Disability-Adjusted Life	Argentine Republic	Male	Age-standardized	Periodontal diseases	Rate	1999	72.81	149.99	28.68

	Years)									
611	DALYs (Disability-Adjusted Life Years)	Argentine Republic	Female	Age-standardized	Periodontal diseases	Rate	1999	58.58	118.49	23.08
612	DALYs (Disability-Adjusted Life Years)	Argentine Republic	Both	Age-standardized	Periodontal diseases	Rate	1999	65.26	133.34	25.70
613	DALYs (Disability-Adjusted Life Years)	Federal Republic of Germany	Male	Age-standardized	Periodontal diseases	Rate	2003	109.57	224.15	42.12
614	DALYs (Disability-Adjusted Life Years)	Federal Republic of Germany	Female	Age-standardized	Periodontal diseases	Rate	2003	96.00	192.99	37.34
615	DALYs (Disability-Adjusted Life Years)	Federal Republic of Germany	Both	Age-standardized	Periodontal diseases	Rate	2003	102.62	207.36	39.66
616	DALYs (Disability-Adjusted Life Years)	Republic of Turkey	Male	Age-standardized	Periodontal diseases	Rate	1998	65.35	129.62	25.39
617	DALYs (Disability-Adjusted Life Years)	Republic of Turkey	Female	Age-standardized	Periodontal diseases	Rate	1998	62.97	123.84	24.56
618	DALYs (Disability-Adjusted Life Years)	Republic of Turkey	Both	Age-standardized	Periodontal diseases	Rate	1998	64.23	126.70	24.64
619	DALYs (Disability-Adjusted Life Years)	Kingdom of Saudi Arabia	Male	Age-standardized	Periodontal diseases	Rate	2003	54.21	114.07	21.30
620	DALYs (Disability-Adjusted Life Years)	Kingdom of Saudi Arabia	Female	Age-standardized	Periodontal diseases	Rate	2003	49.37	105.01	19.26
621	DALYs (Disability-Adjusted Life Years)	Kingdom of Saudi Arabia	Both	Age-standardized	Periodontal diseases	Rate	2003	52.38	110.63	20.64
622	DALYs (Disability-Adjusted Life Years)	Japan	Male	Age-standardized	Periodontal diseases	Rate	1999	68.19	140.67	26.21
623	DALYs (Disability-Adjusted Life Years)	Japan	Female	Age-standardized	Periodontal diseases	Rate	1999	55.26	109.92	20.93
624	DALYs (Disability-Adjusted Life Years)	Japan	Both	Age-standardized	Periodontal diseases	Rate	1999	61.80	125.41	23.62
625	DALYs	Republic of	Male	Age-standardized	Periodontal	Rate	2003	109.95	222.95	44.05

	(Disability-Adjusted Life Years)	India			diseases					
626	DALYs (Disability-Adjusted Life Years)	Republic of India	Female	Age-standardized	Periodontal diseases	Rate	2003	108.12	218.68	43.39
627	DALYs (Disability-Adjusted Life Years)	Republic of India	Both	Age-standardized	Periodontal diseases	Rate	2003	109.04	220.85	43.73
628	DALYs (Disability-Adjusted Life Years)	Argentine Republic	Male	Age-standardized	Periodontal diseases	Rate	1998	76.20	157.47	30.19
629	DALYs (Disability-Adjusted Life Years)	Argentine Republic	Female	Age-standardized	Periodontal diseases	Rate	1998	62.35	127.98	24.67
630	DALYs (Disability-Adjusted Life Years)	Argentine Republic	Both	Age-standardized	Periodontal diseases	Rate	1998	68.86	142.17	27.25
631	DALYs (Disability-Adjusted Life Years)	Russian Federation	Male	Age-standardized	Periodontal diseases	Rate	1998	74.43	155.54	29.59
632	DALYs (Disability-Adjusted Life Years)	Russian Federation	Female	Age-standardized	Periodontal diseases	Rate	1998	74.38	154.88	29.52
633	DALYs (Disability-Adjusted Life Years)	Russian Federation	Both	Age-standardized	Periodontal diseases	Rate	1998	74.24	154.73	29.50
634	DALYs (Disability-Adjusted Life Years)	Russian Federation	Male	Age-standardized	Periodontal diseases	Rate	1999	73.88	154.09	29.30
635	DALYs (Disability-Adjusted Life Years)	Russian Federation	Female	Age-standardized	Periodontal diseases	Rate	1999	74.07	154.50	29.39
636	DALYs (Disability-Adjusted Life Years)	Russian Federation	Both	Age-standardized	Periodontal diseases	Rate	1999	73.83	153.99	29.30
637	DALYs (Disability-Adjusted Life Years)	Australia	Male	Age-standardized	Periodontal diseases	Rate	1999	63.88	130.69	25.18
638	DALYs (Disability-Adjusted Life Years)	Australia	Female	Age-standardized	Periodontal diseases	Rate	1999	46.59	96.48	17.74
639	DALYs (Disability-Adjusted Life Years)	Australia	Both	Age-standardized	Periodontal diseases	Rate	1999	55.01	113.87	21.35

640	DALYs (Disability-Adjusted Life Years)	Federative Republic of Brazil	Male	Age-standardized	Periodontal diseases	Rate	2003	80.33	171.77	32.64
641	DALYs (Disability-Adjusted Life Years)	Federative Republic of Brazil	Female	Age-standardized	Periodontal diseases	Rate	2003	67.78	148.69	27.21
642	DALYs (Disability-Adjusted Life Years)	Federative Republic of Brazil	Both	Age-standardized	Periodontal diseases	Rate	2003	73.78	159.41	29.85
643	DALYs (Disability-Adjusted Life Years)	Republic of Korea	Male	Age-standardized	Periodontal diseases	Rate	2003	59.55	121.35	22.82
644	DALYs (Disability-Adjusted Life Years)	Republic of Korea	Female	Age-standardized	Periodontal diseases	Rate	2003	42.76	89.52	16.06
645	DALYs (Disability-Adjusted Life Years)	Republic of Korea	Both	Age-standardized	Periodontal diseases	Rate	2003	50.83	104.26	19.37
646	DALYs (Disability-Adjusted Life Years)	Republic of South Africa	Male	Age-standardized	Periodontal diseases	Rate	2003	42.78	88.39	16.07
647	DALYs (Disability-Adjusted Life Years)	Republic of South Africa	Female	Age-standardized	Periodontal diseases	Rate	2003	37.08	72.27	14.36
648	DALYs (Disability-Adjusted Life Years)	Republic of South Africa	Both	Age-standardized	Periodontal diseases	Rate	2003	39.62	78.98	15.15
649	DALYs (Disability-Adjusted Life Years)	People's Republic of China	Male	Age-standardized	Periodontal diseases	Rate	2000	57.97	118.10	21.99
650	DALYs (Disability-Adjusted Life Years)	People's Republic of China	Female	Age-standardized	Periodontal diseases	Rate	2000	55.06	111.82	20.58
651	DALYs (Disability-Adjusted Life Years)	People's Republic of China	Both	Age-standardized	Periodontal diseases	Rate	2000	56.57	115.00	21.30
652	DALYs (Disability-Adjusted Life Years)	Republic of Italy	Male	Age-standardized	Periodontal diseases	Rate	1999	61.18	131.95	23.16
653	DALYs (Disability-Adjusted Life Years)	Republic of Italy	Female	Age-standardized	Periodontal diseases	Rate	1999	70.38	149.71	27.09
654	DALYs (Disability-Adjusted Life	Republic of Italy	Both	Age-standardized	Periodontal diseases	Rate	1999	65.85	142.40	25.12

	Years)									
655	DALYs (Disability-Adjusted Life Years)	Canada	Male	Age-standardized	Periodontal diseases	Rate	1999	94.70	185.29	37.59
656	DALYs (Disability-Adjusted Life Years)	Canada	Female	Age-standardized	Periodontal diseases	Rate	1999	81.25	161.12	31.44
657	DALYs (Disability-Adjusted Life Years)	Canada	Both	Age-standardized	Periodontal diseases	Rate	1999	87.84	172.73	34.44
658	DALYs (Disability-Adjusted Life Years)	People's Republic of China	Male	Age-standardized	Periodontal diseases	Rate	1999	58.02	118.36	22.02
659	DALYs (Disability-Adjusted Life Years)	People's Republic of China	Female	Age-standardized	Periodontal diseases	Rate	1999	54.90	111.65	20.54
660	DALYs (Disability-Adjusted Life Years)	People's Republic of China	Both	Age-standardized	Periodontal diseases	Rate	1999	56.51	115.08	21.30
661	DALYs (Disability-Adjusted Life Years)	Republic of Turkey	Male	Age-standardized	Periodontal diseases	Rate	1999	74.43	146.32	28.87
662	DALYs (Disability-Adjusted Life Years)	Republic of Turkey	Female	Age-standardized	Periodontal diseases	Rate	1999	71.08	144.56	27.81
663	DALYs (Disability-Adjusted Life Years)	Republic of Turkey	Both	Age-standardized	Periodontal diseases	Rate	1999	72.82	145.00	28.23
664	DALYs (Disability-Adjusted Life Years)	European Union	Male	Age-standardized	Periodontal diseases	Rate	1999	73.87	150.20	29.62
665	DALYs (Disability-Adjusted Life Years)	European Union	Female	Age-standardized	Periodontal diseases	Rate	1999	69.53	139.22	28.35
666	DALYs (Disability-Adjusted Life Years)	European Union	Both	Age-standardized	Periodontal diseases	Rate	1999	71.62	144.49	28.99
667	DALYs (Disability-Adjusted Life Years)	Federal Republic of Germany	Male	Age-standardized	Periodontal diseases	Rate	2004	98.43	196.57	38.03
668	DALYs (Disability-Adjusted Life Years)	Federal Republic of Germany	Female	Age-standardized	Periodontal diseases	Rate	2004	84.10	167.67	32.29
669	DALYs	Federal	Both	Age-standardized	Periodontal	Rate	2004	91.08	180.85	34.99

	(Disability-Adjusted Life Years)	Republic of Germany			diseases					
670	DALYs (Disability-Adjusted Life Years)	French Republic	Male	Age-standardized	Periodontal diseases	Rate	1999	42.65	86.71	16.46
671	DALYs (Disability-Adjusted Life Years)	French Republic	Female	Age-standardized	Periodontal diseases	Rate	1999	42.27	89.41	16.76
672	DALYs (Disability-Adjusted Life Years)	French Republic	Both	Age-standardized	Periodontal diseases	Rate	1999	42.40	88.33	16.64
673	DALYs (Disability-Adjusted Life Years)	Japan	Male	Age-standardized	Periodontal diseases	Rate	2000	66.17	131.82	25.86
674	DALYs (Disability-Adjusted Life Years)	Japan	Female	Age-standardized	Periodontal diseases	Rate	2000	54.71	108.11	20.95
675	DALYs (Disability-Adjusted Life Years)	Japan	Both	Age-standardized	Periodontal diseases	Rate	2000	60.55	120.69	23.48
676	DALYs (Disability-Adjusted Life Years)	Republic of Indonesia	Male	Age-standardized	Periodontal diseases	Rate	2000	84.30	177.24	33.52
677	DALYs (Disability-Adjusted Life Years)	Republic of Indonesia	Female	Age-standardized	Periodontal diseases	Rate	2000	92.71	197.61	37.44
678	DALYs (Disability-Adjusted Life Years)	Republic of Indonesia	Both	Age-standardized	Periodontal diseases	Rate	2000	88.43	187.30	35.71
679	DALYs (Disability-Adjusted Life Years)	Republic of Indonesia	Male	Age-standardized	Periodontal diseases	Rate	1999	84.22	177.09	33.51
680	DALYs (Disability-Adjusted Life Years)	Republic of Indonesia	Female	Age-standardized	Periodontal diseases	Rate	1999	92.52	197.09	37.34
681	DALYs (Disability-Adjusted Life Years)	Republic of Indonesia	Both	Age-standardized	Periodontal diseases	Rate	1999	88.31	186.97	35.61
682	DALYs (Disability-Adjusted Life Years)	United States of America	Male	Age-standardized	Periodontal diseases	Rate	1999	67.57	141.93	26.52
683	DALYs (Disability-Adjusted Life Years)	United States of America	Female	Age-standardized	Periodontal diseases	Rate	1999	58.28	123.38	22.18

684	DALYs (Disability-Adjusted Life Years)	United States of America	Both	Age-standardized	Periodontal diseases	Rate	1999	62.84	132.51	24.30
685	DALYs (Disability-Adjusted Life Years)	United States of America	Male	Age-standardized	Periodontal diseases	Rate	2000	66.05	139.07	25.79
686	DALYs (Disability-Adjusted Life Years)	United States of America	Female	Age-standardized	Periodontal diseases	Rate	2000	58.59	123.33	22.35
687	DALYs (Disability-Adjusted Life Years)	United States of America	Both	Age-standardized	Periodontal diseases	Rate	2000	62.27	131.09	24.05
688	DALYs (Disability-Adjusted Life Years)	French Republic	Male	Age-standardized	Periodontal diseases	Rate	2000	43.83	89.25	16.98
689	DALYs (Disability-Adjusted Life Years)	French Republic	Female	Age-standardized	Periodontal diseases	Rate	2000	43.38	92.11	17.11
690	DALYs (Disability-Adjusted Life Years)	French Republic	Both	Age-standardized	Periodontal diseases	Rate	2000	43.54	90.32	17.17
691	DALYs (Disability-Adjusted Life Years)	United Mexican States	Male	Age-standardized	Periodontal diseases	Rate	2001	100.46	203.69	40.56
692	DALYs (Disability-Adjusted Life Years)	United Mexican States	Female	Age-standardized	Periodontal diseases	Rate	2001	91.52	191.01	36.62
693	DALYs (Disability-Adjusted Life Years)	United Mexican States	Both	Age-standardized	Periodontal diseases	Rate	2001	95.77	197.04	38.44
694	DALYs (Disability-Adjusted Life Years)	United Mexican States	Male	Age-standardized	Periodontal diseases	Rate	2000	100.48	203.37	40.66
695	DALYs (Disability-Adjusted Life Years)	United Mexican States	Female	Age-standardized	Periodontal diseases	Rate	2000	91.52	191.41	36.71
696	DALYs (Disability-Adjusted Life Years)	United Mexican States	Both	Age-standardized	Periodontal diseases	Rate	2000	95.79	197.05	38.52
697	DALYs (Disability-Adjusted Life Years)	United Kingdom of Great Britain and Northern Ireland	Male	Age-standardized	Periodontal diseases	Rate	2000	28.20	61.27	10.45

698	DALYs (Disability-Adjusted Life Years)	United Kingdom of Great Britain and Northern Ireland	Female	Age-standardized	Periodontal diseases	Rate	2000	28.30	59.97	10.56
699	DALYs (Disability-Adjusted Life Years)	United Kingdom of Great Britain and Northern Ireland	Both	Age-standardized	Periodontal diseases	Rate	2000	28.22	60.55	10.48
700	DALYs (Disability-Adjusted Life Years)	Republic of Korea	Male	Age-standardized	Periodontal diseases	Rate	2004	60.40	122.98	23.15
701	DALYs (Disability-Adjusted Life Years)	Republic of Korea	Female	Age-standardized	Periodontal diseases	Rate	2004	43.26	91.09	16.08
702	DALYs (Disability-Adjusted Life Years)	Republic of Korea	Both	Age-standardized	Periodontal diseases	Rate	2004	51.52	106.19	19.57
703	DALYs (Disability-Adjusted Life Years)	Kingdom of Saudi Arabia	Male	Age-standardized	Periodontal diseases	Rate	2004	54.33	115.60	21.08
704	DALYs (Disability-Adjusted Life Years)	Kingdom of Saudi Arabia	Female	Age-standardized	Periodontal diseases	Rate	2004	49.47	105.00	19.55
705	DALYs (Disability-Adjusted Life Years)	Kingdom of Saudi Arabia	Both	Age-standardized	Periodontal diseases	Rate	2004	52.48	111.34	20.61
706	DALYs (Disability-Adjusted Life Years)	Canada	Male	Age-standardized	Periodontal diseases	Rate	2000	94.41	188.59	37.77
707	DALYs (Disability-Adjusted Life Years)	Canada	Female	Age-standardized	Periodontal diseases	Rate	2000	81.97	167.12	31.36
708	DALYs (Disability-Adjusted Life Years)	Canada	Both	Age-standardized	Periodontal diseases	Rate	2000	88.07	177.97	34.53
709	DALYs (Disability-Adjusted Life Years)	Republic of India	Male	Age-standardized	Periodontal diseases	Rate	2004	110.06	223.16	44.05
710	DALYs (Disability-Adjusted Life Years)	Republic of India	Female	Age-standardized	Periodontal diseases	Rate	2004	108.30	219.11	43.48
711	DALYs	Republic of	Both	Age-standardized	Periodontal	Rate	2004	109.19	221.17	43.72

	(Disability-Adjusted Life Years)	India			diseases					
712	DALYs (Disability-Adjusted Life Years)	United Kingdom of Great Britain and Northern Ireland	Male	Age-standardized	Periodontal diseases	Rate	2001	29.12	62.72	10.76
713	DALYs (Disability-Adjusted Life Years)	United Kingdom of Great Britain and Northern Ireland	Female	Age-standardized	Periodontal diseases	Rate	2001	29.25	61.58	10.93
714	DALYs (Disability-Adjusted Life Years)	United Kingdom of Great Britain and Northern Ireland	Both	Age-standardized	Periodontal diseases	Rate	2001	29.17	62.09	10.84
715	DALYs (Disability-Adjusted Life Years)	Argentine Republic	Male	Age-standardized	Periodontal diseases	Rate	2001	72.44	148.41	28.28
716	DALYs (Disability-Adjusted Life Years)	Argentine Republic	Female	Age-standardized	Periodontal diseases	Rate	2001	58.31	118.98	22.67
717	DALYs (Disability-Adjusted Life Years)	Argentine Republic	Both	Age-standardized	Periodontal diseases	Rate	2001	64.94	133.19	25.30
718	DALYs (Disability-Adjusted Life Years)	Federative Republic of Brazil	Male	Age-standardized	Periodontal diseases	Rate	2004	74.68	159.86	30.49
719	DALYs (Disability-Adjusted Life Years)	Federative Republic of Brazil	Female	Age-standardized	Periodontal diseases	Rate	2004	61.07	135.17	24.53
720	DALYs (Disability-Adjusted Life Years)	Federative Republic of Brazil	Both	Age-standardized	Periodontal diseases	Rate	2004	67.57	146.18	27.46
721	DALYs (Disability-Adjusted Life Years)	Republic of South Africa	Male	Age-standardized	Periodontal diseases	Rate	2004	40.26	80.77	15.37
722	DALYs (Disability-Adjusted Life Years)	Republic of South Africa	Female	Age-standardized	Periodontal diseases	Rate	2004	36.16	70.33	13.87
723	DALYs (Disability-Adjusted Life Years)	Republic of South Africa	Both	Age-standardized	Periodontal diseases	Rate	2004	37.98	74.91	14.58

724	DALYs (Disability-Adjusted Life Years)	Australia	Male	Age-standardized	Periodontal diseases	Rate	2000	65.72	138.32	25.19
725	DALYs (Disability-Adjusted Life Years)	Australia	Female	Age-standardized	Periodontal diseases	Rate	2000	47.43	101.72	17.76
726	DALYs (Disability-Adjusted Life Years)	Australia	Both	Age-standardized	Periodontal diseases	Rate	2000	56.34	119.89	21.64
727	DALYs (Disability-Adjusted Life Years)	Federal Republic of Germany	Male	Age-standardized	Periodontal diseases	Rate	2005	93.79	185.34	36.78
728	DALYs (Disability-Adjusted Life Years)	Federal Republic of Germany	Female	Age-standardized	Periodontal diseases	Rate	2005	79.09	160.64	30.64
729	DALYs (Disability-Adjusted Life Years)	Federal Republic of Germany	Both	Age-standardized	Periodontal diseases	Rate	2005	86.26	171.47	33.65
730	DALYs (Disability-Adjusted Life Years)	Argentine Republic	Male	Age-standardized	Periodontal diseases	Rate	2000	71.34	146.15	28.05
731	DALYs (Disability-Adjusted Life Years)	Argentine Republic	Female	Age-standardized	Periodontal diseases	Rate	2000	57.02	114.58	22.27
732	DALYs (Disability-Adjusted Life Years)	Argentine Republic	Both	Age-standardized	Periodontal diseases	Rate	2000	63.74	129.96	24.97
733	DALYs (Disability-Adjusted Life Years)	Republic of Italy	Male	Age-standardized	Periodontal diseases	Rate	2000	60.95	131.81	23.17
734	DALYs (Disability-Adjusted Life Years)	Republic of Italy	Female	Age-standardized	Periodontal diseases	Rate	2000	70.28	149.90	27.02
735	DALYs (Disability-Adjusted Life Years)	Republic of Italy	Both	Age-standardized	Periodontal diseases	Rate	2000	65.68	141.43	25.03
736	DALYs (Disability-Adjusted Life Years)	Russian Federation	Male	Age-standardized	Periodontal diseases	Rate	2001	73.67	153.96	29.17
737	DALYs (Disability-Adjusted Life Years)	Russian Federation	Female	Age-standardized	Periodontal diseases	Rate	2001	73.70	154.08	29.25
738	DALYs (Disability-Adjusted Life	Russian Federation	Both	Age-standardized	Periodontal diseases	Rate	2001	73.53	153.70	29.18

	Years)									
739	DALYs (Disability-Adjusted Life Years)	Australia	Male	Age-standardized	Periodontal diseases	Rate	2001	65.97	139.11	25.87
740	DALYs (Disability-Adjusted Life Years)	Australia	Female	Age-standardized	Periodontal diseases	Rate	2001	47.69	102.04	18.25
741	DALYs (Disability-Adjusted Life Years)	Australia	Both	Age-standardized	Periodontal diseases	Rate	2001	56.60	121.17	22.16
742	DALYs (Disability-Adjusted Life Years)	Kingdom of Saudi Arabia	Male	Age-standardized	Periodontal diseases	Rate	2005	54.39	115.56	20.82
743	DALYs (Disability-Adjusted Life Years)	Kingdom of Saudi Arabia	Female	Age-standardized	Periodontal diseases	Rate	2005	49.61	106.77	19.71
744	DALYs (Disability-Adjusted Life Years)	Kingdom of Saudi Arabia	Both	Age-standardized	Periodontal diseases	Rate	2005	52.57	112.07	20.52
745	DALYs (Disability-Adjusted Life Years)	Republic of Turkey	Male	Age-standardized	Periodontal diseases	Rate	2000	78.90	160.89	30.48
746	DALYs (Disability-Adjusted Life Years)	Republic of Turkey	Female	Age-standardized	Periodontal diseases	Rate	2000	74.93	156.34	29.11
747	DALYs (Disability-Adjusted Life Years)	Republic of Turkey	Both	Age-standardized	Periodontal diseases	Rate	2000	76.97	158.62	29.97
748	DALYs (Disability-Adjusted Life Years)	Federative Republic of Brazil	Male	Age-standardized	Periodontal diseases	Rate	2005	72.32	154.35	29.32
749	DALYs (Disability-Adjusted Life Years)	Federative Republic of Brazil	Female	Age-standardized	Periodontal diseases	Rate	2005	58.22	129.77	23.13
750	DALYs (Disability-Adjusted Life Years)	Federative Republic of Brazil	Both	Age-standardized	Periodontal diseases	Rate	2005	64.95	141.14	26.28
751	DALYs (Disability-Adjusted Life Years)	Republic of Korea	Male	Age-standardized	Periodontal diseases	Rate	2005	60.89	123.48	23.38
752	DALYs (Disability-Adjusted Life Years)	Republic of Korea	Female	Age-standardized	Periodontal diseases	Rate	2005	43.62	91.77	15.94
753	DALYs	Republic of	Both	Age-standardized	Periodontal	Rate	2005	51.96	107.36	19.65

	(Disability-Adjusted Life Years)	Korea			diseases					
754	DALYs (Disability-Adjusted Life Years)	Japan	Male	Age-standardized	Periodontal diseases	Rate	2001	63.57	126.50	24.79
755	DALYs (Disability-Adjusted Life Years)	Japan	Female	Age-standardized	Periodontal diseases	Rate	2001	52.62	104.47	20.23
756	DALYs (Disability-Adjusted Life Years)	Japan	Both	Age-standardized	Periodontal diseases	Rate	2001	58.19	115.88	22.58
757	DALYs (Disability-Adjusted Life Years)	Republic of South Africa	Male	Age-standardized	Periodontal diseases	Rate	2005	39.27	78.16	15.21
758	DALYs (Disability-Adjusted Life Years)	Republic of South Africa	Female	Age-standardized	Periodontal diseases	Rate	2005	35.93	70.29	13.69
759	DALYs (Disability-Adjusted Life Years)	Republic of South Africa	Both	Age-standardized	Periodontal diseases	Rate	2005	37.41	72.88	14.37
760	DALYs (Disability-Adjusted Life Years)	Republic of India	Male	Age-standardized	Periodontal diseases	Rate	2005	110.09	223.13	44.02
761	DALYs (Disability-Adjusted Life Years)	Republic of India	Female	Age-standardized	Periodontal diseases	Rate	2005	108.39	219.37	43.54
762	DALYs (Disability-Adjusted Life Years)	Republic of India	Both	Age-standardized	Periodontal diseases	Rate	2005	109.25	221.28	43.73
763	DALYs (Disability-Adjusted Life Years)	People's Republic of China	Male	Age-standardized	Periodontal diseases	Rate	2002	63.11	130.99	24.35
764	DALYs (Disability-Adjusted Life Years)	People's Republic of China	Female	Age-standardized	Periodontal diseases	Rate	2002	58.78	120.66	22.49
765	DALYs (Disability-Adjusted Life Years)	People's Republic of China	Both	Age-standardized	Periodontal diseases	Rate	2002	61.00	125.87	23.48
766	DALYs (Disability-Adjusted Life Years)	French Republic	Male	Age-standardized	Periodontal diseases	Rate	2001	43.82	89.82	17.03
767	DALYs (Disability-Adjusted Life Years)	French Republic	Female	Age-standardized	Periodontal diseases	Rate	2001	43.48	91.05	17.18

768	DALYs (Disability-Adjusted Life Years)	French Republic	Both	Age-standardized	Periodontal diseases	Rate	2001	43.59	90.63	17.14
769	DALYs (Disability-Adjusted Life Years)	Republic of Italy	Male	Age-standardized	Periodontal diseases	Rate	2001	60.59	130.33	23.02
770	DALYs (Disability-Adjusted Life Years)	Republic of Italy	Female	Age-standardized	Periodontal diseases	Rate	2001	70.21	149.40	26.93
771	DALYs (Disability-Adjusted Life Years)	Republic of Italy	Both	Age-standardized	Periodontal diseases	Rate	2001	65.48	140.45	24.89
772	DALYs (Disability-Adjusted Life Years)	Russian Federation	Male	Age-standardized	Periodontal diseases	Rate	2000	73.62	153.77	29.11
773	DALYs (Disability-Adjusted Life Years)	Russian Federation	Female	Age-standardized	Periodontal diseases	Rate	2000	73.86	154.02	29.27
774	DALYs (Disability-Adjusted Life Years)	Russian Federation	Both	Age-standardized	Periodontal diseases	Rate	2000	73.60	153.60	29.15
775	DALYs (Disability-Adjusted Life Years)	United States of America	Male	Age-standardized	Periodontal diseases	Rate	2001	64.19	135.71	24.94
776	DALYs (Disability-Adjusted Life Years)	United States of America	Female	Age-standardized	Periodontal diseases	Rate	2001	57.71	121.65	21.91
777	DALYs (Disability-Adjusted Life Years)	United States of America	Both	Age-standardized	Periodontal diseases	Rate	2001	60.91	128.94	23.40
778	DALYs (Disability-Adjusted Life Years)	European Union	Male	Age-standardized	Periodontal diseases	Rate	2000	73.00	145.43	29.26
779	DALYs (Disability-Adjusted Life Years)	European Union	Female	Age-standardized	Periodontal diseases	Rate	2000	68.92	135.71	27.93
780	DALYs (Disability-Adjusted Life Years)	European Union	Both	Age-standardized	Periodontal diseases	Rate	2000	70.88	140.08	28.56
781	DALYs (Disability-Adjusted Life Years)	Republic of Indonesia	Male	Age-standardized	Periodontal diseases	Rate	2001	84.20	176.81	33.50
782	DALYs (Disability-Adjusted Life	Republic of Indonesia	Female	Age-standardized	Periodontal diseases	Rate	2001	92.64	197.21	37.31

	Years)									
783	DALYs (Disability-Adjusted Life Years)	Republic of Indonesia	Both	Age-standardized	Periodontal diseases	Rate	2001	88.35	186.88	35.65
784	DALYs (Disability-Adjusted Life Years)	People's Republic of China	Male	Age-standardized	Periodontal diseases	Rate	2001	59.52	122.23	22.73
785	DALYs (Disability-Adjusted Life Years)	People's Republic of China	Female	Age-standardized	Periodontal diseases	Rate	2001	56.22	114.73	21.28
786	DALYs (Disability-Adjusted Life Years)	People's Republic of China	Both	Age-standardized	Periodontal diseases	Rate	2001	57.93	118.47	22.03
787	DALYs (Disability-Adjusted Life Years)	Republic of Turkey	Male	Age-standardized	Periodontal diseases	Rate	2001	79.71	161.73	31.00
788	DALYs (Disability-Adjusted Life Years)	Republic of Turkey	Female	Age-standardized	Periodontal diseases	Rate	2001	75.17	156.04	28.70
789	DALYs (Disability-Adjusted Life Years)	Republic of Turkey	Both	Age-standardized	Periodontal diseases	Rate	2001	77.49	158.90	29.88
790	DALYs (Disability-Adjusted Life Years)	European Union	Male	Age-standardized	Periodontal diseases	Rate	2001	71.64	142.41	28.69
791	DALYs (Disability-Adjusted Life Years)	European Union	Female	Age-standardized	Periodontal diseases	Rate	2001	67.73	134.63	27.33
792	DALYs (Disability-Adjusted Life Years)	European Union	Both	Age-standardized	Periodontal diseases	Rate	2001	69.61	137.66	28.00
793	DALYs (Disability-Adjusted Life Years)	Japan	Male	Age-standardized	Periodontal diseases	Rate	2002	59.88	119.02	23.27
794	DALYs (Disability-Adjusted Life Years)	Japan	Female	Age-standardized	Periodontal diseases	Rate	2002	48.45	96.11	18.62
795	DALYs (Disability-Adjusted Life Years)	Japan	Both	Age-standardized	Periodontal diseases	Rate	2002	54.21	107.86	20.97
796	DALYs (Disability-Adjusted Life Years)	French Republic	Male	Age-standardized	Periodontal diseases	Rate	2002	43.73	91.05	16.87
797	DALYs	French	Female	Age-standardized	Periodontal	Rate	2002	43.51	91.56	17.21

	(Disability-Adjusted Life Years)	Republic			diseases					
798	DALYs (Disability-Adjusted Life Years)	French Republic	Both	Age-standardized	Periodontal diseases	Rate	2002	43.57	91.33	17.06
799	DALYs (Disability-Adjusted Life Years)	Federal Republic of Germany	Male	Age-standardized	Periodontal diseases	Rate	2006	97.11	193.84	37.58
800	DALYs (Disability-Adjusted Life Years)	Federal Republic of Germany	Female	Age-standardized	Periodontal diseases	Rate	2006	83.20	165.00	31.89
801	DALYs (Disability-Adjusted Life Years)	Federal Republic of Germany	Both	Age-standardized	Periodontal diseases	Rate	2006	90.00	177.27	34.68
802	DALYs (Disability-Adjusted Life Years)	Canada	Male	Age-standardized	Periodontal diseases	Rate	2001	94.56	189.60	38.04
803	DALYs (Disability-Adjusted Life Years)	Canada	Female	Age-standardized	Periodontal diseases	Rate	2001	82.22	168.09	31.63
804	DALYs (Disability-Adjusted Life Years)	Canada	Both	Age-standardized	Periodontal diseases	Rate	2001	88.27	179.15	34.78
805	DALYs (Disability-Adjusted Life Years)	Canada	Male	Age-standardized	Periodontal diseases	Rate	2002	94.89	191.71	38.21
806	DALYs (Disability-Adjusted Life Years)	Canada	Female	Age-standardized	Periodontal diseases	Rate	2002	82.62	168.01	31.45
807	DALYs (Disability-Adjusted Life Years)	Canada	Both	Age-standardized	Periodontal diseases	Rate	2002	88.64	179.65	34.77
808	DALYs (Disability-Adjusted Life Years)	United States of America	Male	Age-standardized	Periodontal diseases	Rate	2002	61.72	131.08	23.78
809	DALYs (Disability-Adjusted Life Years)	United States of America	Female	Age-standardized	Periodontal diseases	Rate	2002	55.55	116.55	20.93
810	DALYs (Disability-Adjusted Life Years)	United States of America	Both	Age-standardized	Periodontal diseases	Rate	2002	58.58	123.90	22.33
811	DALYs (Disability-Adjusted Life Years)	Republic of Indonesia	Male	Age-standardized	Periodontal diseases	Rate	2002	83.98	176.30	33.38

812	DALYs (Disability-Adjusted Life Years)	Republic of Indonesia	Female	Age-standardized	Periodontal diseases	Rate	2002	92.41	196.39	37.16
813	DALYs (Disability-Adjusted Life Years)	Republic of Indonesia	Both	Age-standardized	Periodontal diseases	Rate	2002	88.12	186.21	35.51
814	DALYs (Disability-Adjusted Life Years)	Republic of India	Male	Age-standardized	Periodontal diseases	Rate	2006	109.39	221.62	43.79
815	DALYs (Disability-Adjusted Life Years)	Republic of India	Female	Age-standardized	Periodontal diseases	Rate	2006	107.70	218.09	43.39
816	DALYs (Disability-Adjusted Life Years)	Republic of India	Both	Age-standardized	Periodontal diseases	Rate	2006	108.55	219.87	43.59
817	DALYs (Disability-Adjusted Life Years)	Kingdom of Saudi Arabia	Male	Age-standardized	Periodontal diseases	Rate	2006	54.58	115.29	20.69
818	DALYs (Disability-Adjusted Life Years)	Kingdom of Saudi Arabia	Female	Age-standardized	Periodontal diseases	Rate	2006	49.84	106.30	19.58
819	DALYs (Disability-Adjusted Life Years)	Kingdom of Saudi Arabia	Both	Age-standardized	Periodontal diseases	Rate	2006	52.77	111.99	20.33
820	DALYs (Disability-Adjusted Life Years)	Republic of Korea	Male	Age-standardized	Periodontal diseases	Rate	2006	61.21	125.45	23.34
821	DALYs (Disability-Adjusted Life Years)	Republic of Korea	Female	Age-standardized	Periodontal diseases	Rate	2006	43.82	91.39	16.24
822	DALYs (Disability-Adjusted Life Years)	Republic of Korea	Both	Age-standardized	Periodontal diseases	Rate	2006	52.22	107.32	19.78
823	DALYs (Disability-Adjusted Life Years)	United Kingdom of Great Britain and Northern Ireland	Male	Age-standardized	Periodontal diseases	Rate	2002	31.38	67.23	11.59
824	DALYs (Disability-Adjusted Life Years)	United Kingdom of Great Britain and Northern Ireland	Female	Age-standardized	Periodontal diseases	Rate	2002	31.56	66.30	11.81
825	DALYs	United	Both	Age-standardized	Periodontal	Rate	2002	31.46	66.72	11.69

	(Disability-Adjusted Life Years)	Kingdom of Great Britain and Northern Ireland			diseases					
826	DALYs (Disability-Adjusted Life Years)	Federative Republic of Brazil	Male	Age-standardized	Periodontal diseases	Rate	2006	73.74	157.29	29.97
827	DALYs (Disability-Adjusted Life Years)	Federative Republic of Brazil	Female	Age-standardized	Periodontal diseases	Rate	2006	59.73	131.73	24.01
828	DALYs (Disability-Adjusted Life Years)	Federative Republic of Brazil	Both	Age-standardized	Periodontal diseases	Rate	2006	66.42	143.40	27.04
829	DALYs (Disability-Adjusted Life Years)	Australia	Male	Age-standardized	Periodontal diseases	Rate	2003	65.64	138.30	26.55
830	DALYs (Disability-Adjusted Life Years)	Australia	Female	Age-standardized	Periodontal diseases	Rate	2003	47.85	104.28	18.74
831	DALYs (Disability-Adjusted Life Years)	Australia	Both	Age-standardized	Periodontal diseases	Rate	2003	56.54	121.25	22.54
832	DALYs (Disability-Adjusted Life Years)	United Mexican States	Male	Age-standardized	Periodontal diseases	Rate	2002	100.40	203.93	40.46
833	DALYs (Disability-Adjusted Life Years)	United Mexican States	Female	Age-standardized	Periodontal diseases	Rate	2002	91.44	190.65	36.50
834	DALYs (Disability-Adjusted Life Years)	United Mexican States	Both	Age-standardized	Periodontal diseases	Rate	2002	95.70	196.96	38.38
835	DALYs (Disability-Adjusted Life Years)	Republic of South Africa	Male	Age-standardized	Periodontal diseases	Rate	2006	39.28	77.39	15.19
836	DALYs (Disability-Adjusted Life Years)	Republic of South Africa	Female	Age-standardized	Periodontal diseases	Rate	2006	36.31	71.25	14.00
837	DALYs (Disability-Adjusted Life Years)	Republic of South Africa	Both	Age-standardized	Periodontal diseases	Rate	2006	37.63	73.25	14.54
838	DALYs (Disability-Adjusted Life Years)	Australia	Male	Age-standardized	Periodontal diseases	Rate	2002	65.93	138.70	26.46
839	DALYs	Australia	Female	Age-standardized	Periodontal	Rate	2002	47.84	103.00	18.45

	(Disability-Adjusted Life Years)				diseases					
840	DALYs (Disability-Adjusted Life Years)	Australia	Both	Age-standardized	Periodontal diseases	Rate	2002	56.67	122.15	22.35
841	DALYs (Disability-Adjusted Life Years)	Argentine Republic	Male	Age-standardized	Periodontal diseases	Rate	2003	78.40	152.95	30.76
842	DALYs (Disability-Adjusted Life Years)	Argentine Republic	Female	Age-standardized	Periodontal diseases	Rate	2003	65.12	127.34	25.33
843	DALYs (Disability-Adjusted Life Years)	Argentine Republic	Both	Age-standardized	Periodontal diseases	Rate	2003	71.34	137.60	27.88
844	DALYs (Disability-Adjusted Life Years)	Argentine Republic	Male	Age-standardized	Periodontal diseases	Rate	2002	75.23	152.43	29.45
845	DALYs (Disability-Adjusted Life Years)	Argentine Republic	Female	Age-standardized	Periodontal diseases	Rate	2002	61.37	124.59	23.93
846	DALYs (Disability-Adjusted Life Years)	Argentine Republic	Both	Age-standardized	Periodontal diseases	Rate	2002	67.87	138.08	26.51
847	DALYs (Disability-Adjusted Life Years)	Republic of Turkey	Male	Age-standardized	Periodontal diseases	Rate	2003	80.09	162.72	30.95
848	DALYs (Disability-Adjusted Life Years)	Republic of Turkey	Female	Age-standardized	Periodontal diseases	Rate	2003	73.97	152.08	28.16
849	DALYs (Disability-Adjusted Life Years)	Republic of Turkey	Both	Age-standardized	Periodontal diseases	Rate	2003	77.04	157.42	29.67
850	DALYs (Disability-Adjusted Life Years)	Federal Republic of Germany	Male	Age-standardized	Periodontal diseases	Rate	2007	105.15	213.45	40.56
851	DALYs (Disability-Adjusted Life Years)	Federal Republic of Germany	Female	Age-standardized	Periodontal diseases	Rate	2007	92.85	183.60	35.64
852	DALYs (Disability-Adjusted Life Years)	Federal Republic of Germany	Both	Age-standardized	Periodontal diseases	Rate	2007	98.87	198.14	38.05
853	DALYs (Disability-Adjusted Life Years)	Kingdom of Saudi Arabia	Male	Age-standardized	Periodontal diseases	Rate	2007	54.83	116.14	20.90

854	DALYs (Disability-Adjusted Life Years)	Kingdom of Saudi Arabia	Female	Age-standardized	Periodontal diseases	Rate	2007	50.25	106.27	19.91
855	DALYs (Disability-Adjusted Life Years)	Kingdom of Saudi Arabia	Both	Age-standardized	Periodontal diseases	Rate	2007	53.08	112.69	20.54
856	DALYs (Disability-Adjusted Life Years)	Republic of India	Male	Age-standardized	Periodontal diseases	Rate	2007	107.73	218.36	43.31
857	DALYs (Disability-Adjusted Life Years)	Republic of India	Female	Age-standardized	Periodontal diseases	Rate	2007	106.02	215.12	42.89
858	DALYs (Disability-Adjusted Life Years)	Republic of India	Both	Age-standardized	Periodontal diseases	Rate	2007	106.87	216.74	43.10
859	DALYs (Disability-Adjusted Life Years)	Republic of Korea	Male	Age-standardized	Periodontal diseases	Rate	2007	61.51	125.69	23.36
860	DALYs (Disability-Adjusted Life Years)	Republic of Korea	Female	Age-standardized	Periodontal diseases	Rate	2007	44.04	91.14	16.58
861	DALYs (Disability-Adjusted Life Years)	Republic of Korea	Both	Age-standardized	Periodontal diseases	Rate	2007	52.48	107.21	19.94
862	DALYs (Disability-Adjusted Life Years)	People's Republic of China	Male	Age-standardized	Periodontal diseases	Rate	2003	67.37	141.09	26.18
863	DALYs (Disability-Adjusted Life Years)	People's Republic of China	Female	Age-standardized	Periodontal diseases	Rate	2003	61.78	127.61	23.77
864	DALYs (Disability-Adjusted Life Years)	People's Republic of China	Both	Age-standardized	Periodontal diseases	Rate	2003	64.63	134.53	25.07
865	DALYs (Disability-Adjusted Life Years)	European Union	Male	Age-standardized	Periodontal diseases	Rate	2003	65.82	129.31	26.30
866	DALYs (Disability-Adjusted Life Years)	European Union	Female	Age-standardized	Periodontal diseases	Rate	2003	62.29	124.64	24.90
867	DALYs (Disability-Adjusted Life Years)	European Union	Both	Age-standardized	Periodontal diseases	Rate	2003	63.99	126.51	25.57
868	DALYs (Disability-Adjusted Life	French Republic	Male	Age-standardized	Periodontal diseases	Rate	2003	43.46	90.39	16.86

	Years)									
869	DALYs (Disability-Adjusted Life Years)	French Republic	Female	Age-standardized	Periodontal diseases	Rate	2003	43.48	91.20	17.01
870	DALYs (Disability-Adjusted Life Years)	French Republic	Both	Age-standardized	Periodontal diseases	Rate	2003	43.42	90.37	16.96
871	DALYs (Disability-Adjusted Life Years)	Republic of Italy	Male	Age-standardized	Periodontal diseases	Rate	2003	59.12	126.01	22.38
872	DALYs (Disability-Adjusted Life Years)	Republic of Italy	Female	Age-standardized	Periodontal diseases	Rate	2003	69.98	148.39	26.79
873	DALYs (Disability-Adjusted Life Years)	Republic of Italy	Both	Age-standardized	Periodontal diseases	Rate	2003	64.70	137.95	24.58
874	DALYs (Disability-Adjusted Life Years)	Republic of Turkey	Male	Age-standardized	Periodontal diseases	Rate	2002	80.04	163.55	31.02
875	DALYs (Disability-Adjusted Life Years)	Republic of Turkey	Female	Age-standardized	Periodontal diseases	Rate	2002	74.74	154.12	28.56
876	DALYs (Disability-Adjusted Life Years)	Republic of Turkey	Both	Age-standardized	Periodontal diseases	Rate	2002	77.43	158.08	29.84
877	DALYs (Disability-Adjusted Life Years)	Japan	Male	Age-standardized	Periodontal diseases	Rate	2003	56.06	111.36	21.65
878	DALYs (Disability-Adjusted Life Years)	Japan	Female	Age-standardized	Periodontal diseases	Rate	2003	43.67	85.98	16.75
879	DALYs (Disability-Adjusted Life Years)	Japan	Both	Age-standardized	Periodontal diseases	Rate	2003	49.86	99.06	19.20
880	DALYs (Disability-Adjusted Life Years)	Republic of Italy	Male	Age-standardized	Periodontal diseases	Rate	2002	59.90	128.28	22.77
881	DALYs (Disability-Adjusted Life Years)	Republic of Italy	Female	Age-standardized	Periodontal diseases	Rate	2002	70.10	148.77	26.88
882	DALYs (Disability-Adjusted Life Years)	Republic of Italy	Both	Age-standardized	Periodontal diseases	Rate	2002	65.11	139.22	24.80
883	DALYs	European	Male	Age-standardized	Periodontal	Rate	2002	68.95	136.49	27.57

	(Disability-Adjusted Life Years)	Union			diseases					
884	DALYs (Disability-Adjusted Life Years)	European Union	Female	Age-standardized	Periodontal diseases	Rate	2002	65.22	130.28	26.22
885	DALYs (Disability-Adjusted Life Years)	European Union	Both	Age-standardized	Periodontal diseases	Rate	2002	67.02	132.32	26.87
886	DALYs (Disability-Adjusted Life Years)	United Mexican States	Male	Age-standardized	Periodontal diseases	Rate	2003	100.34	204.07	40.42
887	DALYs (Disability-Adjusted Life Years)	United Mexican States	Female	Age-standardized	Periodontal diseases	Rate	2003	91.35	190.14	36.47
888	DALYs (Disability-Adjusted Life Years)	United Mexican States	Both	Age-standardized	Periodontal diseases	Rate	2003	95.62	196.75	38.34
889	DALYs (Disability-Adjusted Life Years)	Russian Federation	Male	Age-standardized	Periodontal diseases	Rate	2002	73.88	154.81	29.33
890	DALYs (Disability-Adjusted Life Years)	Russian Federation	Female	Age-standardized	Periodontal diseases	Rate	2002	73.50	154.43	28.86
891	DALYs (Disability-Adjusted Life Years)	Russian Federation	Both	Age-standardized	Periodontal diseases	Rate	2002	73.51	154.10	29.10
892	DALYs (Disability-Adjusted Life Years)	Federative Republic of Brazil	Male	Age-standardized	Periodontal diseases	Rate	2007	77.19	164.54	31.22
893	DALYs (Disability-Adjusted Life Years)	Federative Republic of Brazil	Female	Age-standardized	Periodontal diseases	Rate	2007	63.52	138.13	25.69
894	DALYs (Disability-Adjusted Life Years)	Federative Republic of Brazil	Both	Age-standardized	Periodontal diseases	Rate	2007	70.05	150.28	28.34
895	DALYs (Disability-Adjusted Life Years)	Republic of South Africa	Male	Age-standardized	Periodontal diseases	Rate	2007	39.08	76.24	15.14
896	DALYs (Disability-Adjusted Life Years)	Republic of South Africa	Female	Age-standardized	Periodontal diseases	Rate	2007	36.84	72.24	14.25
897	DALYs (Disability-Adjusted Life Years)	Republic of South Africa	Both	Age-standardized	Periodontal diseases	Rate	2007	37.84	73.31	14.66

898	DALYs (Disability-Adjusted Life Years)	Japan	Male	Age-standardized	Periodontal diseases	Rate	2004	53.05	105.36	20.42
899	DALYs (Disability-Adjusted Life Years)	Japan	Female	Age-standardized	Periodontal diseases	Rate	2004	39.73	77.71	15.25
900	DALYs (Disability-Adjusted Life Years)	Japan	Both	Age-standardized	Periodontal diseases	Rate	2004	46.34	91.83	17.81
901	DALYs (Disability-Adjusted Life Years)	United States of America	Male	Age-standardized	Periodontal diseases	Rate	2003	59.01	124.62	22.53
902	DALYs (Disability-Adjusted Life Years)	United States of America	Female	Age-standardized	Periodontal diseases	Rate	2003	52.72	110.18	19.71
903	DALYs (Disability-Adjusted Life Years)	United States of America	Both	Age-standardized	Periodontal diseases	Rate	2003	55.79	117.16	21.08
904	DALYs (Disability-Adjusted Life Years)	People's Republic of China	Male	Age-standardized	Periodontal diseases	Rate	2004	70.93	147.89	27.77
905	DALYs (Disability-Adjusted Life Years)	People's Republic of China	Female	Age-standardized	Periodontal diseases	Rate	2004	64.23	133.63	24.79
906	DALYs (Disability-Adjusted Life Years)	People's Republic of China	Both	Age-standardized	Periodontal diseases	Rate	2004	67.64	141.93	26.34
907	DALYs (Disability-Adjusted Life Years)	Russian Federation	Male	Age-standardized	Periodontal diseases	Rate	2003	74.15	155.45	29.43
908	DALYs (Disability-Adjusted Life Years)	Russian Federation	Female	Age-standardized	Periodontal diseases	Rate	2003	73.30	152.87	28.55
909	DALYs (Disability-Adjusted Life Years)	Russian Federation	Both	Age-standardized	Periodontal diseases	Rate	2003	73.52	154.69	28.85
910	DALYs (Disability-Adjusted Life Years)	United States of America	Male	Age-standardized	Periodontal diseases	Rate	2004	56.39	117.96	21.34
911	DALYs (Disability-Adjusted Life Years)	United States of America	Female	Age-standardized	Periodontal diseases	Rate	2004	49.89	104.08	18.68
912	DALYs (Disability-Adjusted Life	United States of America	Both	Age-standardized	Periodontal diseases	Rate	2004	53.06	110.77	19.85

	Years)									
913	DALYs (Disability-Adjusted Life Years)	Canada	Male	Age-standardized	Periodontal diseases	Rate	2003	95.22	193.98	38.55
914	DALYs (Disability-Adjusted Life Years)	Canada	Female	Age-standardized	Periodontal diseases	Rate	2003	82.92	167.74	31.64
915	DALYs (Disability-Adjusted Life Years)	Canada	Both	Age-standardized	Periodontal diseases	Rate	2003	88.96	180.65	35.03
916	DALYs (Disability-Adjusted Life Years)	French Republic	Male	Age-standardized	Periodontal diseases	Rate	2004	43.30	89.46	16.81
917	DALYs (Disability-Adjusted Life Years)	French Republic	Female	Age-standardized	Periodontal diseases	Rate	2004	43.42	90.33	16.94
918	DALYs (Disability-Adjusted Life Years)	French Republic	Both	Age-standardized	Periodontal diseases	Rate	2004	43.32	89.79	16.88
919	DALYs (Disability-Adjusted Life Years)	Republic of Indonesia	Male	Age-standardized	Periodontal diseases	Rate	2003	83.71	175.94	33.25
920	DALYs (Disability-Adjusted Life Years)	Republic of Indonesia	Female	Age-standardized	Periodontal diseases	Rate	2003	92.13	195.70	36.97
921	DALYs (Disability-Adjusted Life Years)	Republic of Indonesia	Both	Age-standardized	Periodontal diseases	Rate	2003	87.85	185.68	35.37
922	DALYs (Disability-Adjusted Life Years)	Canada	Male	Age-standardized	Periodontal diseases	Rate	2004	95.52	195.27	38.38
923	DALYs (Disability-Adjusted Life Years)	Canada	Female	Age-standardized	Periodontal diseases	Rate	2004	83.22	167.67	31.79
924	DALYs (Disability-Adjusted Life Years)	Canada	Both	Age-standardized	Periodontal diseases	Rate	2004	89.27	181.15	35.01
925	DALYs (Disability-Adjusted Life Years)	Federal Republic of Germany	Male	Age-standardized	Periodontal diseases	Rate	2008	114.65	231.89	44.59
926	DALYs (Disability-Adjusted Life Years)	Federal Republic of Germany	Female	Age-standardized	Periodontal diseases	Rate	2008	104.44	207.30	40.26
927	DALYs	Federal	Both	Age-standardized	Periodontal	Rate	2008	109.44	219.38	42.39

	(Disability-Adjusted Life Years)	Republic of Germany			diseases					
928	DALYs (Disability-Adjusted Life Years)	Republic of Indonesia	Male	Age-standardized	Periodontal diseases	Rate	2004	83.48	175.66	33.07
929	DALYs (Disability-Adjusted Life Years)	Republic of Indonesia	Female	Age-standardized	Periodontal diseases	Rate	2004	91.88	195.01	36.75
930	DALYs (Disability-Adjusted Life Years)	Republic of Indonesia	Both	Age-standardized	Periodontal diseases	Rate	2004	87.61	185.18	35.20
931	DALYs (Disability-Adjusted Life Years)	United Mexican States	Male	Age-standardized	Periodontal diseases	Rate	2004	100.31	204.34	40.32
932	DALYs (Disability-Adjusted Life Years)	United Mexican States	Female	Age-standardized	Periodontal diseases	Rate	2004	91.26	190.05	36.39
933	DALYs (Disability-Adjusted Life Years)	United Mexican States	Both	Age-standardized	Periodontal diseases	Rate	2004	95.56	196.83	38.25
934	DALYs (Disability-Adjusted Life Years)	United Kingdom of Great Britain and Northern Ireland	Male	Age-standardized	Periodontal diseases	Rate	2003	34.13	72.89	12.63
935	DALYs (Disability-Adjusted Life Years)	United Kingdom of Great Britain and Northern Ireland	Female	Age-standardized	Periodontal diseases	Rate	2003	34.41	71.86	12.87
936	DALYs (Disability-Adjusted Life Years)	United Kingdom of Great Britain and Northern Ireland	Both	Age-standardized	Periodontal diseases	Rate	2003	34.27	72.35	12.71
937	DALYs (Disability-Adjusted Life Years)	United Kingdom of Great Britain and Northern Ireland	Male	Age-standardized	Periodontal diseases	Rate	2004	36.57	78.13	13.49
938	DALYs (Disability-Adjusted Life Years)	United Kingdom of Great Britain and Northern	Female	Age-standardized	Periodontal diseases	Rate	2004	37.01	76.89	13.77

		Ireland								
939	DALYs (Disability-Adjusted Life Years)	United Kingdom of Great Britain and Northern Ireland	Both	Age-standardized	Periodontal diseases	Rate	2004	36.79	77.50	13.61
940	DALYs (Disability-Adjusted Life Years)	Kingdom of Saudi Arabia	Male	Age-standardized	Periodontal diseases	Rate	2008	55.13	117.12	21.08
941	DALYs (Disability-Adjusted Life Years)	Kingdom of Saudi Arabia	Female	Age-standardized	Periodontal diseases	Rate	2008	50.70	107.68	20.03
942	DALYs (Disability-Adjusted Life Years)	Kingdom of Saudi Arabia	Both	Age-standardized	Periodontal diseases	Rate	2008	53.43	113.39	20.71
943	DALYs (Disability-Adjusted Life Years)	Republic of India	Male	Age-standardized	Periodontal diseases	Rate	2008	105.76	214.72	42.71
944	DALYs (Disability-Adjusted Life Years)	Republic of India	Female	Age-standardized	Periodontal diseases	Rate	2008	103.98	210.87	42.23
945	DALYs (Disability-Adjusted Life Years)	Republic of India	Both	Age-standardized	Periodontal diseases	Rate	2008	104.86	212.79	42.47
946	DALYs (Disability-Adjusted Life Years)	Republic of Korea	Male	Age-standardized	Periodontal diseases	Rate	2008	61.77	127.12	23.34
947	DALYs (Disability-Adjusted Life Years)	Republic of Korea	Female	Age-standardized	Periodontal diseases	Rate	2008	44.19	91.04	16.81
948	DALYs (Disability-Adjusted Life Years)	Republic of Korea	Both	Age-standardized	Periodontal diseases	Rate	2008	52.70	107.24	20.09
949	DALYs (Disability-Adjusted Life Years)	Argentine Republic	Male	Age-standardized	Periodontal diseases	Rate	2005	82.71	168.88	31.73
950	DALYs (Disability-Adjusted Life Years)	Argentine Republic	Female	Age-standardized	Periodontal diseases	Rate	2005	70.13	145.70	26.96
951	DALYs (Disability-Adjusted Life Years)	Argentine Republic	Both	Age-standardized	Periodontal diseases	Rate	2005	76.03	157.04	29.25
952	DALYs (Disability-Adjusted Life	Federative Republic of	Male	Age-standardized	Periodontal diseases	Rate	2008	81.41	173.29	32.62

	Years)	Brazil								
953	DALYs (Disability-Adjusted Life Years)	Federative Republic of Brazil	Female	Age-standardized	Periodontal diseases	Rate	2008	68.18	145.94	27.49
954	DALYs (Disability-Adjusted Life Years)	Federative Republic of Brazil	Both	Age-standardized	Periodontal diseases	Rate	2008	74.51	159.02	29.93
955	DALYs (Disability-Adjusted Life Years)	Republic of South Africa	Male	Age-standardized	Periodontal diseases	Rate	2008	38.78	75.76	15.01
956	DALYs (Disability-Adjusted Life Years)	Republic of South Africa	Female	Age-standardized	Periodontal diseases	Rate	2008	37.38	73.36	14.53
957	DALYs (Disability-Adjusted Life Years)	Republic of South Africa	Both	Age-standardized	Periodontal diseases	Rate	2008	38.01	74.01	14.76
958	DALYs (Disability-Adjusted Life Years)	Australia	Male	Age-standardized	Periodontal diseases	Rate	2004	65.38	137.58	26.61
959	DALYs (Disability-Adjusted Life Years)	Australia	Female	Age-standardized	Periodontal diseases	Rate	2004	47.86	104.46	19.02
960	DALYs (Disability-Adjusted Life Years)	Australia	Both	Age-standardized	Periodontal diseases	Rate	2004	56.42	120.59	22.72
961	DALYs (Disability-Adjusted Life Years)	Australia	Male	Age-standardized	Periodontal diseases	Rate	2005	65.29	138.91	26.74
962	DALYs (Disability-Adjusted Life Years)	Australia	Female	Age-standardized	Periodontal diseases	Rate	2005	47.85	103.44	19.12
963	DALYs (Disability-Adjusted Life Years)	Australia	Both	Age-standardized	Periodontal diseases	Rate	2005	56.37	120.42	22.89
964	DALYs (Disability-Adjusted Life Years)	Republic of Turkey	Male	Age-standardized	Periodontal diseases	Rate	2005	80.33	164.37	30.69
965	DALYs (Disability-Adjusted Life Years)	Republic of Turkey	Female	Age-standardized	Periodontal diseases	Rate	2005	73.27	150.13	27.41
966	DALYs (Disability-Adjusted Life Years)	Republic of Turkey	Both	Age-standardized	Periodontal diseases	Rate	2005	76.79	157.23	29.16
967	DALYs	Argentine	Male	Age-standardized	Periodontal	Rate	2004	81.27	157.87	31.74

	(Disability-Adjusted Life Years)	Republic			diseases					
968	DALYs (Disability-Adjusted Life Years)	Argentine Republic	Female	Age-standardized	Periodontal diseases	Rate	2004	68.43	137.47	26.55
969	DALYs (Disability-Adjusted Life Years)	Argentine Republic	Both	Age-standardized	Periodontal diseases	Rate	2004	74.45	146.13	29.03
970	DALYs (Disability-Adjusted Life Years)	Federal Republic of Germany	Male	Age-standardized	Periodontal diseases	Rate	2009	122.71	242.93	47.99
971	DALYs (Disability-Adjusted Life Years)	Federal Republic of Germany	Female	Age-standardized	Periodontal diseases	Rate	2009	114.20	223.54	44.29
972	DALYs (Disability-Adjusted Life Years)	Federal Republic of Germany	Both	Age-standardized	Periodontal diseases	Rate	2009	118.37	232.26	46.10
973	DALYs (Disability-Adjusted Life Years)	Republic of Turkey	Male	Age-standardized	Periodontal diseases	Rate	2004	80.13	163.73	30.70
974	DALYs (Disability-Adjusted Life Years)	Republic of Turkey	Female	Age-standardized	Periodontal diseases	Rate	2004	73.34	150.54	27.71
975	DALYs (Disability-Adjusted Life Years)	Republic of Turkey	Both	Age-standardized	Periodontal diseases	Rate	2004	76.73	157.12	29.32
976	DALYs (Disability-Adjusted Life Years)	Republic of Italy	Male	Age-standardized	Periodontal diseases	Rate	2005	58.21	123.14	22.00
977	DALYs (Disability-Adjusted Life Years)	Republic of Italy	Female	Age-standardized	Periodontal diseases	Rate	2005	69.86	147.90	26.73
978	DALYs (Disability-Adjusted Life Years)	Republic of Italy	Both	Age-standardized	Periodontal diseases	Rate	2005	64.24	135.86	24.41
979	DALYs (Disability-Adjusted Life Years)	Japan	Male	Age-standardized	Periodontal diseases	Rate	2005	51.83	102.80	19.93
980	DALYs (Disability-Adjusted Life Years)	Japan	Female	Age-standardized	Periodontal diseases	Rate	2005	38.09	74.22	14.66
981	DALYs (Disability-Adjusted Life Years)	Japan	Both	Age-standardized	Periodontal diseases	Rate	2005	44.90	88.77	17.27

982	DALYs (Disability-Adjusted Life Years)	United States of America	Male	Age-standardized	Periodontal diseases	Rate	2005	54.24	112.73	20.37
983	DALYs (Disability-Adjusted Life Years)	United States of America	Female	Age-standardized	Periodontal diseases	Rate	2005	47.66	99.54	17.89
984	DALYs (Disability-Adjusted Life Years)	United States of America	Both	Age-standardized	Periodontal diseases	Rate	2005	50.86	105.68	19.03
985	DALYs (Disability-Adjusted Life Years)	Republic of Korea	Male	Age-standardized	Periodontal diseases	Rate	2009	61.97	128.72	23.27
986	DALYs (Disability-Adjusted Life Years)	Republic of Korea	Female	Age-standardized	Periodontal diseases	Rate	2009	44.32	90.44	17.06
987	DALYs (Disability-Adjusted Life Years)	Republic of Korea	Both	Age-standardized	Periodontal diseases	Rate	2009	52.88	107.46	20.11
988	DALYs (Disability-Adjusted Life Years)	Japan	Male	Age-standardized	Periodontal diseases	Rate	2006	53.59	105.62	20.88
989	DALYs (Disability-Adjusted Life Years)	Japan	Female	Age-standardized	Periodontal diseases	Rate	2006	39.66	76.46	15.36
990	DALYs (Disability-Adjusted Life Years)	Japan	Both	Age-standardized	Periodontal diseases	Rate	2006	46.56	91.00	18.10
991	DALYs (Disability-Adjusted Life Years)	European Union	Male	Age-standardized	Periodontal diseases	Rate	2004	63.24	123.68	25.18
992	DALYs (Disability-Adjusted Life Years)	European Union	Female	Age-standardized	Periodontal diseases	Rate	2004	59.87	118.91	23.81
993	DALYs (Disability-Adjusted Life Years)	European Union	Both	Age-standardized	Periodontal diseases	Rate	2004	61.49	121.80	24.47
994	DALYs (Disability-Adjusted Life Years)	French Republic	Male	Age-standardized	Periodontal diseases	Rate	2005	43.24	89.55	16.75
995	DALYs (Disability-Adjusted Life Years)	French Republic	Female	Age-standardized	Periodontal diseases	Rate	2005	43.53	90.47	16.82
996	DALYs (Disability-Adjusted Life	French Republic	Both	Age-standardized	Periodontal diseases	Rate	2005	43.35	90.03	16.81

	Years)									
997	DALYs (Disability-Adjusted Life Years)	Kingdom of Saudi Arabia	Male	Age-standardized	Periodontal diseases	Rate	2009	55.46	119.43	21.14
998	DALYs (Disability-Adjusted Life Years)	Kingdom of Saudi Arabia	Female	Age-standardized	Periodontal diseases	Rate	2009	51.16	108.79	20.18
999	DALYs (Disability-Adjusted Life Years)	Kingdom of Saudi Arabia	Both	Age-standardized	Periodontal diseases	Rate	2009	53.81	114.37	20.79
1000	DALYs (Disability-Adjusted Life Years)	Republic of Italy	Male	Age-standardized	Periodontal diseases	Rate	2004	58.49	124.48	22.09
1001	DALYs (Disability-Adjusted Life Years)	Republic of Italy	Female	Age-standardized	Periodontal diseases	Rate	2004	69.89	148.36	26.77
1002	DALYs (Disability-Adjusted Life Years)	Republic of Italy	Both	Age-standardized	Periodontal diseases	Rate	2004	64.38	136.89	24.45
1003	DALYs (Disability-Adjusted Life Years)	Russian Federation	Male	Age-standardized	Periodontal diseases	Rate	2004	74.43	156.16	29.49
1004	DALYs (Disability-Adjusted Life Years)	Russian Federation	Female	Age-standardized	Periodontal diseases	Rate	2004	73.14	149.64	28.57
1005	DALYs (Disability-Adjusted Life Years)	Russian Federation	Both	Age-standardized	Periodontal diseases	Rate	2004	73.55	153.74	28.72
1006	DALYs (Disability-Adjusted Life Years)	Republic of India	Male	Age-standardized	Periodontal diseases	Rate	2009	104.17	211.70	42.26
1007	DALYs (Disability-Adjusted Life Years)	Republic of India	Female	Age-standardized	Periodontal diseases	Rate	2009	102.32	207.76	41.62
1008	DALYs (Disability-Adjusted Life Years)	Republic of India	Both	Age-standardized	Periodontal diseases	Rate	2009	103.23	209.70	41.93
1009	DALYs (Disability-Adjusted Life Years)	European Union	Male	Age-standardized	Periodontal diseases	Rate	2005	62.14	121.83	24.61
1010	DALYs (Disability-Adjusted Life Years)	European Union	Female	Age-standardized	Periodontal diseases	Rate	2005	58.87	115.92	23.27
1011	DALYs	European	Both	Age-standardized	Periodontal	Rate	2005	60.44	119.65	23.92

	(Disability-Adjusted Life Years)	Union			diseases					
1012	DALYs (Disability-Adjusted Life Years)	People's Republic of China	Male	Age-standardized	Periodontal diseases	Rate	2005	72.40	149.77	28.44
1013	DALYs (Disability-Adjusted Life Years)	People's Republic of China	Female	Age-standardized	Periodontal diseases	Rate	2005	65.20	137.07	25.05
1014	DALYs (Disability-Adjusted Life Years)	People's Republic of China	Both	Age-standardized	Periodontal diseases	Rate	2005	68.85	144.55	26.83
1015	DALYs (Disability-Adjusted Life Years)	Canada	Male	Age-standardized	Periodontal diseases	Rate	2005	95.64	196.43	38.60
1016	DALYs (Disability-Adjusted Life Years)	Canada	Female	Age-standardized	Periodontal diseases	Rate	2005	83.45	167.09	32.05
1017	DALYs (Disability-Adjusted Life Years)	Canada	Both	Age-standardized	Periodontal diseases	Rate	2005	89.44	181.44	35.04
1018	DALYs (Disability-Adjusted Life Years)	French Republic	Male	Age-standardized	Periodontal diseases	Rate	2006	43.67	90.12	16.86
1019	DALYs (Disability-Adjusted Life Years)	French Republic	Female	Age-standardized	Periodontal diseases	Rate	2006	43.99	91.51	17.12
1020	DALYs (Disability-Adjusted Life Years)	French Republic	Both	Age-standardized	Periodontal diseases	Rate	2006	43.80	90.68	17.01
1021	DALYs (Disability-Adjusted Life Years)	Russian Federation	Male	Age-standardized	Periodontal diseases	Rate	2005	74.63	156.68	29.41
1022	DALYs (Disability-Adjusted Life Years)	Russian Federation	Female	Age-standardized	Periodontal diseases	Rate	2005	73.06	146.10	28.83
1023	DALYs (Disability-Adjusted Life Years)	Russian Federation	Both	Age-standardized	Periodontal diseases	Rate	2005	73.59	151.61	28.61
1024	DALYs (Disability-Adjusted Life Years)	Republic of South Africa	Male	Age-standardized	Periodontal diseases	Rate	2009	38.46	74.99	14.90
1025	DALYs (Disability-Adjusted Life Years)	Republic of South Africa	Female	Age-standardized	Periodontal diseases	Rate	2009	37.81	74.38	14.77

1026	DALYs (Disability-Adjusted Life Years)	Republic of South Africa	Both	Age-standardized	Periodontal diseases	Rate	2009	38.11	74.53	14.84
1027	DALYs (Disability-Adjusted Life Years)	Federative Republic of Brazil	Male	Age-standardized	Periodontal diseases	Rate	2009	85.10	180.89	33.78
1028	DALYs (Disability-Adjusted Life Years)	Federative Republic of Brazil	Female	Age-standardized	Periodontal diseases	Rate	2009	72.33	154.37	28.99
1029	DALYs (Disability-Adjusted Life Years)	Federative Republic of Brazil	Both	Age-standardized	Periodontal diseases	Rate	2009	78.44	167.06	31.28
1030	DALYs (Disability-Adjusted Life Years)	Republic of Indonesia	Male	Age-standardized	Periodontal diseases	Rate	2006	83.53	175.72	32.96
1031	DALYs (Disability-Adjusted Life Years)	Republic of Indonesia	Female	Age-standardized	Periodontal diseases	Rate	2006	91.90	195.00	36.81
1032	DALYs (Disability-Adjusted Life Years)	Republic of Indonesia	Both	Age-standardized	Periodontal diseases	Rate	2006	87.64	185.19	35.14
1033	DALYs (Disability-Adjusted Life Years)	United Kingdom of Great Britain and Northern Ireland	Male	Age-standardized	Periodontal diseases	Rate	2005	37.89	80.42	13.93
1034	DALYs (Disability-Adjusted Life Years)	United Kingdom of Great Britain and Northern Ireland	Female	Age-standardized	Periodontal diseases	Rate	2005	38.53	79.64	14.41
1035	DALYs (Disability-Adjusted Life Years)	United Kingdom of Great Britain and Northern Ireland	Both	Age-standardized	Periodontal diseases	Rate	2005	38.22	79.98	14.11
1036	DALYs (Disability-Adjusted Life Years)	United Mexican States	Male	Age-standardized	Periodontal diseases	Rate	2005	100.33	204.99	40.24
1037	DALYs (Disability-Adjusted Life Years)	United Mexican States	Female	Age-standardized	Periodontal diseases	Rate	2005	91.24	189.70	36.42
1038	DALYs (Disability-Adjusted Life	United Mexican States	Both	Age-standardized	Periodontal diseases	Rate	2005	95.55	196.77	38.23

	Years)										
1039	DALYs (Disability-Adjusted Life Years)	Republic of Indonesia	Male	Age-standardized	Periodontal diseases	Rate		2005	83.41	175.65	32.95
1040	DALYs (Disability-Adjusted Life Years)	Republic of Indonesia	Female	Age-standardized	Periodontal diseases	Rate		2005	91.81	195.30	36.81
1041	DALYs (Disability-Adjusted Life Years)	Republic of Indonesia	Both	Age-standardized	Periodontal diseases	Rate		2005	87.53	185.32	35.19
1042	DALYs (Disability-Adjusted Life Years)	People's Republic of China	Male	Age-standardized	Periodontal diseases	Rate		2006	70.45	147.97	27.42
1043	DALYs (Disability-Adjusted Life Years)	People's Republic of China	Female	Age-standardized	Periodontal diseases	Rate		2006	63.54	133.84	24.28
1044	DALYs (Disability-Adjusted Life Years)	People's Republic of China	Both	Age-standardized	Periodontal diseases	Rate		2006	67.04	141.01	25.86
1045	DALYs (Disability-Adjusted Life Years)	United Mexican States	Male	Age-standardized	Periodontal diseases	Rate		2006	100.65	205.79	40.49
1046	DALYs (Disability-Adjusted Life Years)	United Mexican States	Female	Age-standardized	Periodontal diseases	Rate		2006	91.40	189.87	36.49
1047	DALYs (Disability-Adjusted Life Years)	United Mexican States	Both	Age-standardized	Periodontal diseases	Rate		2006	95.78	197.15	38.39
1048	DALYs (Disability-Adjusted Life Years)	United Kingdom of Great Britain and Northern Ireland	Male	Age-standardized	Periodontal diseases	Rate		2006	38.31	80.60	14.10
1049	DALYs (Disability-Adjusted Life Years)	United Kingdom of Great Britain and Northern Ireland	Female	Age-standardized	Periodontal diseases	Rate		2006	39.27	80.81	14.72
1050	DALYs (Disability-Adjusted Life Years)	United Kingdom of Great Britain and Northern Ireland	Both	Age-standardized	Periodontal diseases	Rate		2006	38.80	80.71	14.37
1051	DALYs	Canada	Male	Age-standardized	Periodontal	Rate		2006	95.76	195.22	38.48

	(Disability-Adjusted Life Years)				diseases					
1052	DALYs (Disability-Adjusted Life Years)	Canada	Female	Age-standardized	Periodontal diseases	Rate	2006	83.59	167.50	31.84
1053	DALYs (Disability-Adjusted Life Years)	Canada	Both	Age-standardized	Periodontal diseases	Rate	2006	89.57	180.95	35.06
1054	DALYs (Disability-Adjusted Life Years)	Federal Republic of Germany	Male	Age-standardized	Periodontal diseases	Rate	2010	126.08	246.33	49.45
1055	DALYs (Disability-Adjusted Life Years)	Federal Republic of Germany	Female	Age-standardized	Periodontal diseases	Rate	2010	118.23	230.54	46.15
1056	DALYs (Disability-Adjusted Life Years)	Federal Republic of Germany	Both	Age-standardized	Periodontal diseases	Rate	2010	122.08	237.49	47.73
1057	DALYs (Disability-Adjusted Life Years)	Kingdom of Saudi Arabia	Male	Age-standardized	Periodontal diseases	Rate	2010	55.82	121.61	21.38
1058	DALYs (Disability-Adjusted Life Years)	Kingdom of Saudi Arabia	Female	Age-standardized	Periodontal diseases	Rate	2010	51.53	107.92	20.24
1059	DALYs (Disability-Adjusted Life Years)	Kingdom of Saudi Arabia	Both	Age-standardized	Periodontal diseases	Rate	2010	54.17	115.73	20.96
1060	DALYs (Disability-Adjusted Life Years)	Republic of India	Male	Age-standardized	Periodontal diseases	Rate	2010	103.57	210.19	42.25
1061	DALYs (Disability-Adjusted Life Years)	Republic of India	Female	Age-standardized	Periodontal diseases	Rate	2010	101.65	206.59	41.33
1062	DALYs (Disability-Adjusted Life Years)	Republic of India	Both	Age-standardized	Periodontal diseases	Rate	2010	102.59	208.36	41.88
1063	DALYs (Disability-Adjusted Life Years)	Argentine Republic	Male	Age-standardized	Periodontal diseases	Rate	2007	83.54	170.99	31.98
1064	DALYs (Disability-Adjusted Life Years)	Argentine Republic	Female	Age-standardized	Periodontal diseases	Rate	2007	71.32	149.81	27.56
1065	DALYs (Disability-Adjusted Life Years)	Argentine Republic	Both	Age-standardized	Periodontal diseases	Rate	2007	77.05	159.59	29.64

1066	DALYs (Disability-Adjusted Life Years)	Argentine Republic	Male	Age-standardized	Periodontal diseases	Rate	2006	83.14	171.59	31.77
1067	DALYs (Disability-Adjusted Life Years)	Argentine Republic	Female	Age-standardized	Periodontal diseases	Rate	2006	70.74	148.00	27.31
1068	DALYs (Disability-Adjusted Life Years)	Argentine Republic	Both	Age-standardized	Periodontal diseases	Rate	2006	76.56	158.41	29.41
1069	DALYs (Disability-Adjusted Life Years)	Republic of Korea	Male	Age-standardized	Periodontal diseases	Rate	2010	62.06	129.01	23.15
1070	DALYs (Disability-Adjusted Life Years)	Republic of Korea	Female	Age-standardized	Periodontal diseases	Rate	2010	44.37	90.17	17.32
1071	DALYs (Disability-Adjusted Life Years)	Republic of Korea	Both	Age-standardized	Periodontal diseases	Rate	2010	52.96	107.79	20.24
1072	DALYs (Disability-Adjusted Life Years)	Federative Republic of Brazil	Male	Age-standardized	Periodontal diseases	Rate	2010	87.02	185.06	34.32
1073	DALYs (Disability-Adjusted Life Years)	Federative Republic of Brazil	Female	Age-standardized	Periodontal diseases	Rate	2010	74.56	158.43	29.79
1074	DALYs (Disability-Adjusted Life Years)	Federative Republic of Brazil	Both	Age-standardized	Periodontal diseases	Rate	2010	80.53	171.18	31.95
1075	DALYs (Disability-Adjusted Life Years)	Australia	Male	Age-standardized	Periodontal diseases	Rate	2006	65.72	139.12	26.92
1076	DALYs (Disability-Adjusted Life Years)	Australia	Female	Age-standardized	Periodontal diseases	Rate	2006	48.29	105.39	19.09
1077	DALYs (Disability-Adjusted Life Years)	Australia	Both	Age-standardized	Periodontal diseases	Rate	2006	56.81	121.83	22.90
1078	DALYs (Disability-Adjusted Life Years)	Republic of South Africa	Male	Age-standardized	Periodontal diseases	Rate	2010	38.26	75.39	14.78
1079	DALYs (Disability-Adjusted Life Years)	Republic of South Africa	Female	Age-standardized	Periodontal diseases	Rate	2010	37.99	74.68	14.93
1080	DALYs (Disability-Adjusted Life	Republic of South Africa	Both	Age-standardized	Periodontal diseases	Rate	2010	38.12	74.91	14.88

	Years)									
1081	DALYs (Disability-Adjusted Life Years)	Australia	Male	Age-standardized	Periodontal diseases	Rate	2007	66.63	141.67	26.98
1082	DALYs (Disability-Adjusted Life Years)	Australia	Female	Age-standardized	Periodontal diseases	Rate	2007	49.13	105.60	19.07
1083	DALYs (Disability-Adjusted Life Years)	Australia	Both	Age-standardized	Periodontal diseases	Rate	2007	57.68	123.83	22.93
1084	DALYs (Disability-Adjusted Life Years)	European Union	Male	Age-standardized	Periodontal diseases	Rate	2006	62.98	124.41	24.97
1085	DALYs (Disability-Adjusted Life Years)	European Union	Female	Age-standardized	Periodontal diseases	Rate	2006	59.83	118.53	23.71
1086	DALYs (Disability-Adjusted Life Years)	European Union	Both	Age-standardized	Periodontal diseases	Rate	2006	61.35	122.15	24.32
1087	DALYs (Disability-Adjusted Life Years)	Republic of Italy	Male	Age-standardized	Periodontal diseases	Rate	2006	58.57	123.44	22.09
1088	DALYs (Disability-Adjusted Life Years)	Republic of Italy	Female	Age-standardized	Periodontal diseases	Rate	2006	70.00	148.14	26.82
1089	DALYs (Disability-Adjusted Life Years)	Republic of Italy	Both	Age-standardized	Periodontal diseases	Rate	2006	64.47	136.09	24.52
1090	DALYs (Disability-Adjusted Life Years)	United States of America	Male	Age-standardized	Periodontal diseases	Rate	2006	52.08	108.31	19.50
1091	DALYs (Disability-Adjusted Life Years)	United States of America	Female	Age-standardized	Periodontal diseases	Rate	2006	45.56	95.28	17.19
1092	DALYs (Disability-Adjusted Life Years)	United States of America	Both	Age-standardized	Periodontal diseases	Rate	2006	48.73	101.46	18.31
1093	DALYs (Disability-Adjusted Life Years)	Republic of Turkey	Male	Age-standardized	Periodontal diseases	Rate	2006	81.03	165.69	31.13
1094	DALYs (Disability-Adjusted Life Years)	Republic of Turkey	Female	Age-standardized	Periodontal diseases	Rate	2006	74.01	152.39	27.80
1095	DALYs	Republic of	Both	Age-standardized	Periodontal	Rate	2006	77.50	159.03	29.62

	(Disability-Adjusted Life Years)	Turkey			diseases					
1096	DALYs (Disability-Adjusted Life Years)	Japan	Male	Age-standardized	Periodontal diseases	Rate	2007	57.78	113.38	22.91
1097	DALYs (Disability-Adjusted Life Years)	Japan	Female	Age-standardized	Periodontal diseases	Rate	2007	43.40	83.36	16.94
1098	DALYs (Disability-Adjusted Life Years)	Japan	Both	Age-standardized	Periodontal diseases	Rate	2007	50.52	98.24	19.94
1099	DALYs (Disability-Adjusted Life Years)	Republic of Italy	Male	Age-standardized	Periodontal diseases	Rate	2007	59.48	124.62	22.50
1100	DALYs (Disability-Adjusted Life Years)	Republic of Italy	Female	Age-standardized	Periodontal diseases	Rate	2007	70.32	148.54	27.02
1101	DALYs (Disability-Adjusted Life Years)	Republic of Italy	Both	Age-standardized	Periodontal diseases	Rate	2007	65.04	136.78	24.81
1102	DALYs (Disability-Adjusted Life Years)	Federal Republic of Germany	Male	Age-standardized	Periodontal diseases	Rate	2011	124.51	244.78	49.10
1103	DALYs (Disability-Adjusted Life Years)	Federal Republic of Germany	Female	Age-standardized	Periodontal diseases	Rate	2011	116.62	228.42	45.33
1104	DALYs (Disability-Adjusted Life Years)	Federal Republic of Germany	Both	Age-standardized	Periodontal diseases	Rate	2011	120.49	236.21	47.19
1105	DALYs (Disability-Adjusted Life Years)	French Republic	Male	Age-standardized	Periodontal diseases	Rate	2008	45.60	94.97	17.67
1106	DALYs (Disability-Adjusted Life Years)	French Republic	Female	Age-standardized	Periodontal diseases	Rate	2008	45.99	94.92	17.93
1107	DALYs (Disability-Adjusted Life Years)	French Republic	Both	Age-standardized	Periodontal diseases	Rate	2008	45.77	95.74	17.83
1108	DALYs (Disability-Adjusted Life Years)	French Republic	Male	Age-standardized	Periodontal diseases	Rate	2007	44.58	92.33	17.26
1109	DALYs (Disability-Adjusted Life Years)	French Republic	Female	Age-standardized	Periodontal diseases	Rate	2007	44.89	93.37	17.54

1110	DALYs (Disability-Adjusted Life Years)	French Republic	Both	Age-standardized	Periodontal diseases	Rate	2007	44.71	92.58	17.45
1111	DALYs (Disability-Adjusted Life Years)	Kingdom of Saudi Arabia	Male	Age-standardized	Periodontal diseases	Rate	2011	56.19	120.21	21.55
1112	DALYs (Disability-Adjusted Life Years)	Kingdom of Saudi Arabia	Female	Age-standardized	Periodontal diseases	Rate	2011	51.85	108.76	20.42
1113	DALYs (Disability-Adjusted Life Years)	Kingdom of Saudi Arabia	Both	Age-standardized	Periodontal diseases	Rate	2011	54.53	114.92	21.13
1114	DALYs (Disability-Adjusted Life Years)	Republic of Turkey	Male	Age-standardized	Periodontal diseases	Rate	2007	82.15	167.31	31.53
1115	DALYs (Disability-Adjusted Life Years)	Republic of Turkey	Female	Age-standardized	Periodontal diseases	Rate	2007	75.33	156.32	28.55
1116	DALYs (Disability-Adjusted Life Years)	Republic of Turkey	Both	Age-standardized	Periodontal diseases	Rate	2007	78.71	161.78	30.12
1117	DALYs (Disability-Adjusted Life Years)	Republic of Indonesia	Male	Age-standardized	Periodontal diseases	Rate	2008	84.07	177.31	33.12
1118	DALYs (Disability-Adjusted Life Years)	Republic of Indonesia	Female	Age-standardized	Periodontal diseases	Rate	2008	92.30	196.38	37.02
1119	DALYs (Disability-Adjusted Life Years)	Republic of Indonesia	Both	Age-standardized	Periodontal diseases	Rate	2008	88.10	186.66	35.26
1120	DALYs (Disability-Adjusted Life Years)	Republic of India	Male	Age-standardized	Periodontal diseases	Rate	2011	104.61	213.47	42.42
1121	DALYs (Disability-Adjusted Life Years)	Republic of India	Female	Age-standardized	Periodontal diseases	Rate	2011	102.31	209.57	40.94
1122	DALYs (Disability-Adjusted Life Years)	Republic of India	Both	Age-standardized	Periodontal diseases	Rate	2011	103.45	211.49	41.93
1123	DALYs (Disability-Adjusted Life Years)	Republic of Indonesia	Male	Age-standardized	Periodontal diseases	Rate	2007	83.76	176.27	32.96
1124	DALYs (Disability-Adjusted Life	Republic of Indonesia	Female	Age-standardized	Periodontal diseases	Rate	2007	92.09	195.84	36.89

	Years)									
1125	DALYs (Disability-Adjusted Life Years)	Republic of Indonesia	Both	Age-standardized	Periodontal diseases	Rate	2007	87.85	185.88	35.15
1126	DALYs (Disability-Adjusted Life Years)	United Kingdom of Great Britain and Northern Ireland	Male	Age-standardized	Periodontal diseases	Rate	2008	38.82	81.07	14.31
1127	DALYs (Disability-Adjusted Life Years)	United Kingdom of Great Britain and Northern Ireland	Female	Age-standardized	Periodontal diseases	Rate	2008	40.54	82.57	15.25
1128	DALYs (Disability-Adjusted Life Years)	United Kingdom of Great Britain and Northern Ireland	Both	Age-standardized	Periodontal diseases	Rate	2008	39.69	81.82	14.78
1129	DALYs (Disability-Adjusted Life Years)	Russian Federation	Male	Age-standardized	Periodontal diseases	Rate	2006	74.40	154.34	29.03
1130	DALYs (Disability-Adjusted Life Years)	Russian Federation	Female	Age-standardized	Periodontal diseases	Rate	2006	72.75	141.38	28.83
1131	DALYs (Disability-Adjusted Life Years)	Russian Federation	Both	Age-standardized	Periodontal diseases	Rate	2006	73.30	146.61	28.99
1132	DALYs (Disability-Adjusted Life Years)	People's Republic of China	Male	Age-standardized	Periodontal diseases	Rate	2007	65.90	138.88	25.35
1133	DALYs (Disability-Adjusted Life Years)	People's Republic of China	Female	Age-standardized	Periodontal diseases	Rate	2007	59.76	126.28	22.73
1134	DALYs (Disability-Adjusted Life Years)	People's Republic of China	Both	Age-standardized	Periodontal diseases	Rate	2007	62.86	131.81	24.10
1135	DALYs (Disability-Adjusted Life Years)	United States of America	Male	Age-standardized	Periodontal diseases	Rate	2007	49.47	103.33	18.55
1136	DALYs (Disability-Adjusted Life Years)	United States of America	Female	Age-standardized	Periodontal diseases	Rate	2007	43.02	90.41	16.28
1137	DALYs	United States of	Both	Age-standardized	Periodontal	Rate	2007	46.15	96.73	17.38

	(Disability-Adjusted Life Years)	America			diseases					
1138	DALYs (Disability-Adjusted Life Years)	Republic of Korea	Male	Age-standardized	Periodontal diseases	Rate	2011	61.87	128.21	23.31
1139	DALYs (Disability-Adjusted Life Years)	Republic of Korea	Female	Age-standardized	Periodontal diseases	Rate	2011	44.30	91.21	17.11
1140	DALYs (Disability-Adjusted Life Years)	Republic of Korea	Both	Age-standardized	Periodontal diseases	Rate	2011	52.85	108.12	20.19
1141	DALYs (Disability-Adjusted Life Years)	Federative Republic of Brazil	Male	Age-standardized	Periodontal diseases	Rate	2011	87.34	186.24	34.50
1142	DALYs (Disability-Adjusted Life Years)	Federative Republic of Brazil	Female	Age-standardized	Periodontal diseases	Rate	2011	75.15	159.67	29.84
1143	DALYs (Disability-Adjusted Life Years)	Federative Republic of Brazil	Both	Age-standardized	Periodontal diseases	Rate	2011	80.98	172.39	32.06
1144	DALYs (Disability-Adjusted Life Years)	Russian Federation	Male	Age-standardized	Periodontal diseases	Rate	2007	73.65	148.39	28.98
1145	DALYs (Disability-Adjusted Life Years)	Russian Federation	Female	Age-standardized	Periodontal diseases	Rate	2007	72.04	141.08	28.51
1146	DALYs (Disability-Adjusted Life Years)	Russian Federation	Both	Age-standardized	Periodontal diseases	Rate	2007	72.55	141.37	28.84
1147	DALYs (Disability-Adjusted Life Years)	People's Republic of China	Male	Age-standardized	Periodontal diseases	Rate	2008	60.49	126.53	23.13
1148	DALYs (Disability-Adjusted Life Years)	People's Republic of China	Female	Age-standardized	Periodontal diseases	Rate	2008	55.33	117.03	20.93
1149	DALYs (Disability-Adjusted Life Years)	People's Republic of China	Both	Age-standardized	Periodontal diseases	Rate	2008	57.93	121.49	22.08
1150	DALYs (Disability-Adjusted Life Years)	Republic of South Africa	Male	Age-standardized	Periodontal diseases	Rate	2011	38.09	75.01	14.76
1151	DALYs (Disability-Adjusted Life Years)	Republic of South Africa	Female	Age-standardized	Periodontal diseases	Rate	2011	37.90	74.48	14.92

1152	DALYs (Disability-Adjusted Life Years)	Republic of South Africa	Both	Age-standardized	Periodontal diseases	Rate	2011	38.00	74.65	14.85
1153	DALYs (Disability-Adjusted Life Years)	Canada	Male	Age-standardized	Periodontal diseases	Rate	2007	95.86	194.53	37.98
1154	DALYs (Disability-Adjusted Life Years)	Canada	Female	Age-standardized	Periodontal diseases	Rate	2007	83.78	169.05	31.98
1155	DALYs (Disability-Adjusted Life Years)	Canada	Both	Age-standardized	Periodontal diseases	Rate	2007	89.72	180.94	34.93
1156	DALYs (Disability-Adjusted Life Years)	United Mexican States	Male	Age-standardized	Periodontal diseases	Rate	2008	102.11	208.79	41.25
1157	DALYs (Disability-Adjusted Life Years)	United Mexican States	Female	Age-standardized	Periodontal diseases	Rate	2008	92.24	191.53	36.85
1158	DALYs (Disability-Adjusted Life Years)	United Mexican States	Both	Age-standardized	Periodontal diseases	Rate	2008	96.92	199.22	38.93
1159	DALYs (Disability-Adjusted Life Years)	United Mexican States	Male	Age-standardized	Periodontal diseases	Rate	2007	101.32	207.32	40.86
1160	DALYs (Disability-Adjusted Life Years)	United Mexican States	Female	Age-standardized	Periodontal diseases	Rate	2007	91.79	190.78	36.69
1161	DALYs (Disability-Adjusted Life Years)	United Mexican States	Both	Age-standardized	Periodontal diseases	Rate	2007	96.30	198.13	38.66
1162	DALYs (Disability-Adjusted Life Years)	Canada	Male	Age-standardized	Periodontal diseases	Rate	2008	95.82	194.23	37.61
1163	DALYs (Disability-Adjusted Life Years)	Canada	Female	Age-standardized	Periodontal diseases	Rate	2008	83.92	169.61	31.97
1164	DALYs (Disability-Adjusted Life Years)	Canada	Both	Age-standardized	Periodontal diseases	Rate	2008	89.77	181.57	34.74
1165	DALYs (Disability-Adjusted Life Years)	Federal Republic of Germany	Male	Age-standardized	Periodontal diseases	Rate	2012	120.80	238.30	47.47
1166	DALYs (Disability-Adjusted Life	Federal Republic of	Female	Age-standardized	Periodontal diseases	Rate	2012	112.77	223.80	43.77

	Years)	Germany								
1167	DALYs (Disability-Adjusted Life Years)	Federal Republic of Germany	Both	Age-standardized	Periodontal diseases	Rate	2012	116.70	230.47	45.58
1168	DALYs (Disability-Adjusted Life Years)	Argentine Republic	Male	Age-standardized	Periodontal diseases	Rate	2008	83.91	171.44	32.35
1169	DALYs (Disability-Adjusted Life Years)	Argentine Republic	Female	Age-standardized	Periodontal diseases	Rate	2008	71.78	151.13	27.86
1170	DALYs (Disability-Adjusted Life Years)	Argentine Republic	Both	Age-standardized	Periodontal diseases	Rate	2008	77.47	160.71	29.90
1171	DALYs (Disability-Adjusted Life Years)	United Kingdom of Great Britain and Northern Ireland	Male	Age-standardized	Periodontal diseases	Rate	2007	38.62	81.04	14.23
1172	DALYs (Disability-Adjusted Life Years)	United Kingdom of Great Britain and Northern Ireland	Female	Age-standardized	Periodontal diseases	Rate	2007	39.96	81.86	14.98
1173	DALYs (Disability-Adjusted Life Years)	United Kingdom of Great Britain and Northern Ireland	Both	Age-standardized	Periodontal diseases	Rate	2007	39.30	81.45	14.58
1174	DALYs (Disability-Adjusted Life Years)	Republic of India	Male	Age-standardized	Periodontal diseases	Rate	2012	106.93	218.95	43.07
1175	DALYs (Disability-Adjusted Life Years)	Republic of India	Female	Age-standardized	Periodontal diseases	Rate	2012	103.85	214.60	41.32
1176	DALYs (Disability-Adjusted Life Years)	Republic of India	Both	Age-standardized	Periodontal diseases	Rate	2012	105.39	216.75	42.22
1177	DALYs (Disability-Adjusted Life Years)	Kingdom of Saudi Arabia	Male	Age-standardized	Periodontal diseases	Rate	2012	56.73	118.98	21.81
1178	DALYs (Disability-Adjusted Life Years)	Kingdom of Saudi Arabia	Female	Age-standardized	Periodontal diseases	Rate	2012	52.18	110.15	20.62
1179	DALYs	Kingdom of	Both	Age-standardized	Periodontal	Rate	2012	55.01	115.98	21.37

	(Disability-Adjusted Life Years)	Saudi Arabia			diseases					
1180	DALYs (Disability-Adjusted Life Years)	Republic of Korea	Male	Age-standardized	Periodontal diseases	Rate	2012	61.36	125.35	23.22
1181	DALYs (Disability-Adjusted Life Years)	Republic of Korea	Female	Age-standardized	Periodontal diseases	Rate	2012	43.91	90.41	16.88
1182	DALYs (Disability-Adjusted Life Years)	Republic of Korea	Both	Age-standardized	Periodontal diseases	Rate	2012	52.43	107.78	20.05
1183	DALYs (Disability-Adjusted Life Years)	Federative Republic of Brazil	Male	Age-standardized	Periodontal diseases	Rate	2012	87.21	186.48	34.50
1184	DALYs (Disability-Adjusted Life Years)	Federative Republic of Brazil	Female	Age-standardized	Periodontal diseases	Rate	2012	75.45	160.64	29.72
1185	DALYs (Disability-Adjusted Life Years)	Federative Republic of Brazil	Both	Age-standardized	Periodontal diseases	Rate	2012	81.08	173.00	32.00
1186	DALYs (Disability-Adjusted Life Years)	Republic of South Africa	Male	Age-standardized	Periodontal diseases	Rate	2012	37.84	74.43	14.63
1187	DALYs (Disability-Adjusted Life Years)	Republic of South Africa	Female	Age-standardized	Periodontal diseases	Rate	2012	37.67	73.76	14.78
1188	DALYs (Disability-Adjusted Life Years)	Republic of South Africa	Both	Age-standardized	Periodontal diseases	Rate	2012	37.77	74.05	14.72
1189	DALYs (Disability-Adjusted Life Years)	Japan	Male	Age-standardized	Periodontal diseases	Rate	2008	62.81	122.89	25.15
1190	DALYs (Disability-Adjusted Life Years)	Japan	Female	Age-standardized	Periodontal diseases	Rate	2008	47.89	92.04	18.90
1191	DALYs (Disability-Adjusted Life Years)	Japan	Both	Age-standardized	Periodontal diseases	Rate	2008	55.27	107.29	22.01
1192	DALYs (Disability-Adjusted Life Years)	Republic of Turkey	Male	Age-standardized	Periodontal diseases	Rate	2008	83.42	170.70	31.96
1193	DALYs (Disability-Adjusted Life Years)	Republic of Turkey	Female	Age-standardized	Periodontal diseases	Rate	2008	76.79	159.93	29.23

1194	DALYs (Disability-Adjusted Life Years)	Republic of Turkey	Both	Age-standardized	Periodontal diseases	Rate	2008	80.06	165.26	30.62
1195	DALYs (Disability-Adjusted Life Years)	Australia	Male	Age-standardized	Periodontal diseases	Rate	2008	67.74	144.13	26.83
1196	DALYs (Disability-Adjusted Life Years)	Australia	Female	Age-standardized	Periodontal diseases	Rate	2008	50.25	107.17	19.39
1197	DALYs (Disability-Adjusted Life Years)	Australia	Both	Age-standardized	Periodontal diseases	Rate	2008	58.79	126.81	23.01
1198	DALYs (Disability-Adjusted Life Years)	United States of America	Male	Age-standardized	Periodontal diseases	Rate	2008	46.97	98.70	17.69
1199	DALYs (Disability-Adjusted Life Years)	United States of America	Female	Age-standardized	Periodontal diseases	Rate	2008	40.57	85.60	15.42
1200	DALYs (Disability-Adjusted Life Years)	United States of America	Both	Age-standardized	Periodontal diseases	Rate	2008	43.68	92.21	16.52
1201	DALYs (Disability-Adjusted Life Years)	Japan	Male	Age-standardized	Periodontal diseases	Rate	2009	67.06	130.56	26.75
1202	DALYs (Disability-Adjusted Life Years)	Japan	Female	Age-standardized	Periodontal diseases	Rate	2009	51.70	100.36	20.49
1203	DALYs (Disability-Adjusted Life Years)	Japan	Both	Age-standardized	Periodontal diseases	Rate	2009	59.30	115.12	23.73
1204	DALYs (Disability-Adjusted Life Years)	Australia	Male	Age-standardized	Periodontal diseases	Rate	2009	68.68	147.78	26.82
1205	DALYs (Disability-Adjusted Life Years)	Australia	Female	Age-standardized	Periodontal diseases	Rate	2009	51.11	109.27	19.67
1206	DALYs (Disability-Adjusted Life Years)	Australia	Both	Age-standardized	Periodontal diseases	Rate	2009	59.69	128.29	23.23
1207	DALYs (Disability-Adjusted Life Years)	Republic of Indonesia	Male	Age-standardized	Periodontal diseases	Rate	2009	84.34	177.79	33.10
1208	DALYs (Disability-Adjusted Life	Republic of Indonesia	Female	Age-standardized	Periodontal diseases	Rate	2009	92.47	197.44	37.14

	Years)									
1209	DALYs (Disability-Adjusted Life Years)	Republic of Indonesia	Both	Age-standardized	Periodontal diseases	Rate	2009	88.31	187.42	35.27
1210	DALYs (Disability-Adjusted Life Years)	French Republic	Male	Age-standardized	Periodontal diseases	Rate	2009	46.53	97.84	17.83
1211	DALYs (Disability-Adjusted Life Years)	French Republic	Female	Age-standardized	Periodontal diseases	Rate	2009	46.86	96.29	18.38
1212	DALYs (Disability-Adjusted Life Years)	French Republic	Both	Age-standardized	Periodontal diseases	Rate	2009	46.67	97.81	18.14
1213	DALYs (Disability-Adjusted Life Years)	Federal Republic of Germany	Male	Age-standardized	Periodontal diseases	Rate	2013	116.33	231.65	45.66
1214	DALYs (Disability-Adjusted Life Years)	Federal Republic of Germany	Female	Age-standardized	Periodontal diseases	Rate	2013	108.16	217.21	41.63
1215	DALYs (Disability-Adjusted Life Years)	Federal Republic of Germany	Both	Age-standardized	Periodontal diseases	Rate	2013	112.16	224.00	43.67
1216	DALYs (Disability-Adjusted Life Years)	United States of America	Male	Age-standardized	Periodontal diseases	Rate	2009	45.10	95.83	17.04
1217	DALYs (Disability-Adjusted Life Years)	United States of America	Female	Age-standardized	Periodontal diseases	Rate	2009	38.74	82.20	14.75
1218	DALYs (Disability-Adjusted Life Years)	United States of America	Both	Age-standardized	Periodontal diseases	Rate	2009	41.82	88.92	15.87
1219	DALYs (Disability-Adjusted Life Years)	Kingdom of Saudi Arabia	Male	Age-standardized	Periodontal diseases	Rate	2013	57.26	120.52	22.23
1220	DALYs (Disability-Adjusted Life Years)	Kingdom of Saudi Arabia	Female	Age-standardized	Periodontal diseases	Rate	2013	52.46	109.70	20.67
1221	DALYs (Disability-Adjusted Life Years)	Kingdom of Saudi Arabia	Both	Age-standardized	Periodontal diseases	Rate	2013	55.45	116.78	21.65
1222	DALYs (Disability-Adjusted Life Years)	European Union	Male	Age-standardized	Periodontal diseases	Rate	2008	67.60	136.29	26.95
1223	DALYs	European	Female	Age-standardized	Periodontal	Rate	2008	64.76	130.24	25.66

	(Disability-Adjusted Life Years)	Union			diseases					
1224	DALYs (Disability-Adjusted Life Years)	European Union	Both	Age-standardized	Periodontal diseases	Rate	2008	66.12	133.58	26.28
1225	DALYs (Disability-Adjusted Life Years)	French Republic	Male	Age-standardized	Periodontal diseases	Rate	2010	46.94	99.00	17.80
1226	DALYs (Disability-Adjusted Life Years)	French Republic	Female	Age-standardized	Periodontal diseases	Rate	2010	47.28	96.99	18.59
1227	DALYs (Disability-Adjusted Life Years)	French Republic	Both	Age-standardized	Periodontal diseases	Rate	2010	47.09	98.51	18.21
1228	DALYs (Disability-Adjusted Life Years)	Republic of Italy	Male	Age-standardized	Periodontal diseases	Rate	2008	60.57	126.08	22.89
1229	DALYs (Disability-Adjusted Life Years)	Republic of Italy	Female	Age-standardized	Periodontal diseases	Rate	2008	70.67	149.09	27.23
1230	DALYs (Disability-Adjusted Life Years)	Republic of Italy	Both	Age-standardized	Periodontal diseases	Rate	2008	65.72	137.80	25.09
1231	DALYs (Disability-Adjusted Life Years)	Republic of Korea	Male	Age-standardized	Periodontal diseases	Rate	2013	60.74	124.41	23.07
1232	DALYs (Disability-Adjusted Life Years)	Republic of Korea	Female	Age-standardized	Periodontal diseases	Rate	2013	43.50	90.43	16.42
1233	DALYs (Disability-Adjusted Life Years)	Republic of Korea	Both	Age-standardized	Periodontal diseases	Rate	2013	51.93	107.88	19.73
1234	DALYs (Disability-Adjusted Life Years)	Republic of India	Male	Age-standardized	Periodontal diseases	Rate	2013	109.71	225.41	43.85
1235	DALYs (Disability-Adjusted Life Years)	Republic of India	Female	Age-standardized	Periodontal diseases	Rate	2013	105.72	216.60	42.17
1236	DALYs (Disability-Adjusted Life Years)	Republic of India	Both	Age-standardized	Periodontal diseases	Rate	2013	107.73	222.69	42.83
1237	DALYs (Disability-Adjusted Life Years)	United Kingdom of Great Britain	Male	Age-standardized	Periodontal diseases	Rate	2009	38.91	81.08	14.34

		and Northern Ireland								
1238	DALYs (Disability-Adjusted Life Years)	United Kingdom of Great Britain and Northern Ireland	Female	Age-standardized	Periodontal diseases	Rate	2009	40.94	82.89	15.44
1239	DALYs (Disability-Adjusted Life Years)	United Kingdom of Great Britain and Northern Ireland	Both	Age-standardized	Periodontal diseases	Rate	2009	39.93	81.98	14.90
1240	DALYs (Disability-Adjusted Life Years)	Federative Republic of Brazil	Male	Age-standardized	Periodontal diseases	Rate	2013	86.87	186.15	34.58
1241	DALYs (Disability-Adjusted Life Years)	Federative Republic of Brazil	Female	Age-standardized	Periodontal diseases	Rate	2013	75.52	161.08	29.65
1242	DALYs (Disability-Adjusted Life Years)	Federative Republic of Brazil	Both	Age-standardized	Periodontal diseases	Rate	2013	80.94	173.05	32.00
1243	DALYs (Disability-Adjusted Life Years)	Republic of Turkey	Male	Age-standardized	Periodontal diseases	Rate	2009	84.58	173.13	32.68
1244	DALYs (Disability-Adjusted Life Years)	Republic of Turkey	Female	Age-standardized	Periodontal diseases	Rate	2009	78.11	162.20	29.91
1245	DALYs (Disability-Adjusted Life Years)	Republic of Turkey	Both	Age-standardized	Periodontal diseases	Rate	2009	81.29	168.02	31.18
1246	DALYs (Disability-Adjusted Life Years)	Republic of Italy	Male	Age-standardized	Periodontal diseases	Rate	2009	61.47	127.82	23.27
1247	DALYs (Disability-Adjusted Life Years)	Republic of Italy	Female	Age-standardized	Periodontal diseases	Rate	2009	70.96	149.47	27.38
1248	DALYs (Disability-Adjusted Life Years)	Republic of Italy	Both	Age-standardized	Periodontal diseases	Rate	2009	66.28	138.86	25.35
1249	DALYs (Disability-Adjusted Life Years)	Republic of Indonesia	Male	Age-standardized	Periodontal diseases	Rate	2010	84.52	178.30	33.16
1250	DALYs (Disability-Adjusted Life	Republic of Indonesia	Female	Age-standardized	Periodontal diseases	Rate	2010	92.62	198.08	37.24

	Years)										
1251	DALYs (Disability-Adjusted Life Years)	Republic of Indonesia	Both	Age-standardized	Periodontal diseases	Rate		2010	88.48	187.99	35.31
1252	DALYs (Disability-Adjusted Life Years)	United Kingdom of Great Britain and Northern Ireland	Male	Age-standardized	Periodontal diseases	Rate		2010	38.90	80.75	14.39
1253	DALYs (Disability-Adjusted Life Years)	United Kingdom of Great Britain and Northern Ireland	Female	Age-standardized	Periodontal diseases	Rate		2010	41.07	83.05	15.49
1254	DALYs (Disability-Adjusted Life Years)	United Kingdom of Great Britain and Northern Ireland	Both	Age-standardized	Periodontal diseases	Rate		2010	40.00	81.90	14.95
1255	DALYs (Disability-Adjusted Life Years)	Republic of South Africa	Male	Age-standardized	Periodontal diseases	Rate		2013	37.54	73.75	14.54
1256	DALYs (Disability-Adjusted Life Years)	Republic of South Africa	Female	Age-standardized	Periodontal diseases	Rate		2013	37.39	73.49	14.63
1257	DALYs (Disability-Adjusted Life Years)	Republic of South Africa	Both	Age-standardized	Periodontal diseases	Rate		2013	37.47	73.59	14.61
1258	DALYs (Disability-Adjusted Life Years)	United Mexican States	Male	Age-standardized	Periodontal diseases	Rate		2009	102.79	210.29	41.58
1259	DALYs (Disability-Adjusted Life Years)	United Mexican States	Female	Age-standardized	Periodontal diseases	Rate		2009	92.61	192.62	37.03
1260	DALYs (Disability-Adjusted Life Years)	United Mexican States	Both	Age-standardized	Periodontal diseases	Rate		2009	97.44	200.35	39.18
1261	DALYs (Disability-Adjusted Life Years)	People's Republic of China	Male	Age-standardized	Periodontal diseases	Rate		2009	55.96	117.45	21.31
1262	DALYs (Disability-Adjusted Life Years)	People's Republic of China	Female	Age-standardized	Periodontal diseases	Rate		2009	51.62	109.52	19.43
1263	DALYs	People's	Both	Age-standardized	Periodontal	Rate		2009	53.80	113.50	20.37

	(Disability-Adjusted Life Years)	Republic of China			diseases					
1264	DALYs (Disability-Adjusted Life Years)	Russian Federation	Male	Age-standardized	Periodontal diseases	Rate	2009	71.94	142.07	28.57
1265	DALYs (Disability-Adjusted Life Years)	Russian Federation	Female	Age-standardized	Periodontal diseases	Rate	2009	70.52	142.48	27.62
1266	DALYs (Disability-Adjusted Life Years)	Russian Federation	Both	Age-standardized	Periodontal diseases	Rate	2009	70.89	141.22	27.92
1267	DALYs (Disability-Adjusted Life Years)	Russian Federation	Male	Age-standardized	Periodontal diseases	Rate	2008	72.72	142.39	29.02
1268	DALYs (Disability-Adjusted Life Years)	Russian Federation	Female	Age-standardized	Periodontal diseases	Rate	2008	71.20	141.93	28.04
1269	DALYs (Disability-Adjusted Life Years)	Russian Federation	Both	Age-standardized	Periodontal diseases	Rate	2008	71.64	140.93	28.37
1270	DALYs (Disability-Adjusted Life Years)	United Mexican States	Male	Age-standardized	Periodontal diseases	Rate	2010	103.12	211.05	41.82
1271	DALYs (Disability-Adjusted Life Years)	United Mexican States	Female	Age-standardized	Periodontal diseases	Rate	2010	92.77	193.01	37.09
1272	DALYs (Disability-Adjusted Life Years)	United Mexican States	Both	Age-standardized	Periodontal diseases	Rate	2010	97.67	200.80	39.33
1273	DALYs (Disability-Adjusted Life Years)	People's Republic of China	Male	Age-standardized	Periodontal diseases	Rate	2010	54.04	114.57	20.37
1274	DALYs (Disability-Adjusted Life Years)	People's Republic of China	Female	Age-standardized	Periodontal diseases	Rate	2010	50.08	106.56	18.72
1275	DALYs (Disability-Adjusted Life Years)	People's Republic of China	Both	Age-standardized	Periodontal diseases	Rate	2010	52.07	110.58	19.55
1276	DALYs (Disability-Adjusted Life Years)	Argentine Republic	Male	Age-standardized	Periodontal diseases	Rate	2009	84.19	172.82	32.50
1277	DALYs (Disability-Adjusted Life Years)	Argentine Republic	Female	Age-standardized	Periodontal diseases	Rate	2009	72.19	153.00	27.89

1278	DALYs (Disability-Adjusted Life Years)	Argentine Republic	Both	Age-standardized	Periodontal diseases	Rate	2009	77.83	162.34	29.95
1279	DALYs (Disability-Adjusted Life Years)	Canada	Male	Age-standardized	Periodontal diseases	Rate	2009	95.94	193.09	37.03
1280	DALYs (Disability-Adjusted Life Years)	Canada	Female	Age-standardized	Periodontal diseases	Rate	2009	84.03	169.02	32.18
1281	DALYs (Disability-Adjusted Life Years)	Canada	Both	Age-standardized	Periodontal diseases	Rate	2009	89.89	180.55	34.56
1282	DALYs (Disability-Adjusted Life Years)	Federal Republic of Germany	Male	Age-standardized	Periodontal diseases	Rate	2014	112.60	227.90	43.98
1283	DALYs (Disability-Adjusted Life Years)	Federal Republic of Germany	Female	Age-standardized	Periodontal diseases	Rate	2014	104.29	210.67	39.84
1284	DALYs (Disability-Adjusted Life Years)	Federal Republic of Germany	Both	Age-standardized	Periodontal diseases	Rate	2014	108.36	218.71	42.10
1285	DALYs (Disability-Adjusted Life Years)	Argentine Republic	Male	Age-standardized	Periodontal diseases	Rate	2010	84.53	172.23	32.50
1286	DALYs (Disability-Adjusted Life Years)	Argentine Republic	Female	Age-standardized	Periodontal diseases	Rate	2010	72.51	153.61	27.99
1287	DALYs (Disability-Adjusted Life Years)	Argentine Republic	Both	Age-standardized	Periodontal diseases	Rate	2010	78.16	162.38	29.95
1288	DALYs (Disability-Adjusted Life Years)	European Union	Male	Age-standardized	Periodontal diseases	Rate	2007	65.11	129.77	25.88
1289	DALYs (Disability-Adjusted Life Years)	European Union	Female	Age-standardized	Periodontal diseases	Rate	2007	62.08	123.77	24.61
1290	DALYs (Disability-Adjusted Life Years)	European Union	Both	Age-standardized	Periodontal diseases	Rate	2007	63.54	127.25	25.22
1291	DALYs (Disability-Adjusted Life Years)	Canada	Male	Age-standardized	Periodontal diseases	Rate	2010	95.98	190.60	36.43
1292	DALYs (Disability-Adjusted Life	Canada	Female	Age-standardized	Periodontal diseases	Rate	2010	84.13	168.78	32.30

	Years)									
1293	DALYs (Disability-Adjusted Life Years)	Canada	Both	Age-standardized	Periodontal diseases	Rate	2010	89.95	179.49	34.32
1294	DALYs (Disability-Adjusted Life Years)	Republic of India	Male	Age-standardized	Periodontal diseases	Rate	2014	112.13	231.27	44.89
1295	DALYs (Disability-Adjusted Life Years)	Republic of India	Female	Age-standardized	Periodontal diseases	Rate	2014	107.35	216.70	43.02
1296	DALYs (Disability-Adjusted Life Years)	Republic of India	Both	Age-standardized	Periodontal diseases	Rate	2014	109.77	227.16	43.62
1297	DALYs (Disability-Adjusted Life Years)	Kingdom of Saudi Arabia	Male	Age-standardized	Periodontal diseases	Rate	2014	57.69	120.12	22.44
1298	DALYs (Disability-Adjusted Life Years)	Kingdom of Saudi Arabia	Female	Age-standardized	Periodontal diseases	Rate	2014	52.69	111.15	20.86
1299	DALYs (Disability-Adjusted Life Years)	Kingdom of Saudi Arabia	Both	Age-standardized	Periodontal diseases	Rate	2014	55.83	117.08	21.85
1300	DALYs (Disability-Adjusted Life Years)	Republic of Korea	Male	Age-standardized	Periodontal diseases	Rate	2014	60.23	123.43	23.08
1301	DALYs (Disability-Adjusted Life Years)	Republic of Korea	Female	Age-standardized	Periodontal diseases	Rate	2014	43.18	90.16	16.00
1302	DALYs (Disability-Adjusted Life Years)	Republic of Korea	Both	Age-standardized	Periodontal diseases	Rate	2014	51.54	107.49	19.56
1303	DALYs (Disability-Adjusted Life Years)	Republic of South Africa	Male	Age-standardized	Periodontal diseases	Rate	2014	37.28	73.50	14.49
1304	DALYs (Disability-Adjusted Life Years)	Republic of South Africa	Female	Age-standardized	Periodontal diseases	Rate	2014	37.14	72.98	14.49
1305	DALYs (Disability-Adjusted Life Years)	Republic of South Africa	Both	Age-standardized	Periodontal diseases	Rate	2014	37.22	72.98	14.51
1306	DALYs (Disability-Adjusted Life Years)	Federative Republic of Brazil	Male	Age-standardized	Periodontal diseases	Rate	2014	86.57	185.83	34.52
1307	DALYs	Federative	Female	Age-standardized	Periodontal	Rate	2014	75.52	161.25	29.55

	(Disability-Adjusted Life Years)	Republic of Brazil			diseases					
1308	DALYs (Disability-Adjusted Life Years)	Federative Republic of Brazil	Both	Age-standardized	Periodontal diseases	Rate	2014	80.79	172.97	31.92
1309	DALYs (Disability-Adjusted Life Years)	Japan	Male	Age-standardized	Periodontal diseases	Rate	2011	68.96	133.86	27.28
1310	DALYs (Disability-Adjusted Life Years)	Japan	Female	Age-standardized	Periodontal diseases	Rate	2011	53.56	105.36	21.20
1311	DALYs (Disability-Adjusted Life Years)	Japan	Both	Age-standardized	Periodontal diseases	Rate	2011	61.19	119.40	24.39
1312	DALYs (Disability-Adjusted Life Years)	European Union	Male	Age-standardized	Periodontal diseases	Rate	2009	69.71	141.74	27.82
1313	DALYs (Disability-Adjusted Life Years)	European Union	Female	Age-standardized	Periodontal diseases	Rate	2009	66.97	135.67	26.60
1314	DALYs (Disability-Adjusted Life Years)	European Union	Both	Age-standardized	Periodontal diseases	Rate	2009	68.28	138.58	27.19
1315	DALYs (Disability-Adjusted Life Years)	Federal Republic of Germany	Male	Age-standardized	Periodontal diseases	Rate	2015	110.98	227.14	43.19
1316	DALYs (Disability-Adjusted Life Years)	Federal Republic of Germany	Female	Age-standardized	Periodontal diseases	Rate	2015	102.63	205.92	39.21
1317	DALYs (Disability-Adjusted Life Years)	Federal Republic of Germany	Both	Age-standardized	Periodontal diseases	Rate	2015	106.73	218.76	41.36
1318	DALYs (Disability-Adjusted Life Years)	United States of America	Male	Age-standardized	Periodontal diseases	Rate	2011	46.61	99.59	17.79
1319	DALYs (Disability-Adjusted Life Years)	United States of America	Female	Age-standardized	Periodontal diseases	Rate	2011	39.98	84.07	15.14
1320	DALYs (Disability-Adjusted Life Years)	United States of America	Both	Age-standardized	Periodontal diseases	Rate	2011	43.21	91.78	16.46
1321	DALYs (Disability-Adjusted Life Years)	Japan	Male	Age-standardized	Periodontal diseases	Rate	2010	68.95	134.00	27.22

1322	DALYs (Disability-Adjusted Life Years)	Japan	Female	Age-standardized	Periodontal diseases	Rate	2010	53.41	104.79	21.18
1323	DALYs (Disability-Adjusted Life Years)	Japan	Both	Age-standardized	Periodontal diseases	Rate	2010	61.10	119.19	24.31
1324	DALYs (Disability-Adjusted Life Years)	French Republic	Male	Age-standardized	Periodontal diseases	Rate	2011	46.85	98.49	17.84
1325	DALYs (Disability-Adjusted Life Years)	French Republic	Female	Age-standardized	Periodontal diseases	Rate	2011	47.19	97.84	18.51
1326	DALYs (Disability-Adjusted Life Years)	French Republic	Both	Age-standardized	Periodontal diseases	Rate	2011	47.00	98.68	18.19
1327	DALYs (Disability-Adjusted Life Years)	Republic of Turkey	Male	Age-standardized	Periodontal diseases	Rate	2010	85.42	174.47	32.94
1328	DALYs (Disability-Adjusted Life Years)	Republic of Turkey	Female	Age-standardized	Periodontal diseases	Rate	2010	78.95	162.28	30.50
1329	DALYs (Disability-Adjusted Life Years)	Republic of Turkey	Both	Age-standardized	Periodontal diseases	Rate	2010	82.12	169.59	31.66
1330	DALYs (Disability-Adjusted Life Years)	Australia	Male	Age-standardized	Periodontal diseases	Rate	2010	69.22	148.68	26.56
1331	DALYs (Disability-Adjusted Life Years)	Australia	Female	Age-standardized	Periodontal diseases	Rate	2010	51.44	107.36	19.56
1332	DALYs (Disability-Adjusted Life Years)	Australia	Both	Age-standardized	Periodontal diseases	Rate	2010	60.13	129.14	22.97
1333	DALYs (Disability-Adjusted Life Years)	Australia	Male	Age-standardized	Periodontal diseases	Rate	2011	70.03	150.25	26.68
1334	DALYs (Disability-Adjusted Life Years)	Australia	Female	Age-standardized	Periodontal diseases	Rate	2011	51.41	108.33	19.71
1335	DALYs (Disability-Adjusted Life Years)	Australia	Both	Age-standardized	Periodontal diseases	Rate	2011	60.50	129.86	23.24
1336	DALYs (Disability-Adjusted Life	United States of America	Male	Age-standardized	Periodontal diseases	Rate	2010	44.40	95.36	16.90

	Years)										
1337	DALYs (Disability-Adjusted Life Years)	United States of America	Female	Age-standardized	Periodontal diseases	Rate		2010	38.05	80.75	14.46
1338	DALYs (Disability-Adjusted Life Years)	United States of America	Both	Age-standardized	Periodontal diseases	Rate		2010	41.13	87.87	15.68
1339	DALYs (Disability-Adjusted Life Years)	Republic of Indonesia	Male	Age-standardized	Periodontal diseases	Rate		2011	84.66	178.50	33.34
1340	DALYs (Disability-Adjusted Life Years)	Republic of Indonesia	Female	Age-standardized	Periodontal diseases	Rate		2011	92.77	198.39	37.32
1341	DALYs (Disability-Adjusted Life Years)	Republic of Indonesia	Both	Age-standardized	Periodontal diseases	Rate		2011	88.63	188.24	35.48
1342	DALYs (Disability-Adjusted Life Years)	Republic of Indonesia	Male	Age-standardized	Periodontal diseases	Rate		2012	84.83	179.00	33.60
1343	DALYs (Disability-Adjusted Life Years)	Republic of Indonesia	Female	Age-standardized	Periodontal diseases	Rate		2012	92.95	198.51	37.41
1344	DALYs (Disability-Adjusted Life Years)	Republic of Indonesia	Both	Age-standardized	Periodontal diseases	Rate		2012	88.80	188.56	35.63
1345	DALYs (Disability-Adjusted Life Years)	Republic of India	Male	Age-standardized	Periodontal diseases	Rate		2015	113.32	234.63	45.30
1346	DALYs (Disability-Adjusted Life Years)	Republic of India	Female	Age-standardized	Periodontal diseases	Rate		2015	108.18	215.95	43.47
1347	DALYs (Disability-Adjusted Life Years)	Republic of India	Both	Age-standardized	Periodontal diseases	Rate		2015	110.79	226.64	44.37
1348	DALYs (Disability-Adjusted Life Years)	Kingdom of Saudi Arabia	Male	Age-standardized	Periodontal diseases	Rate		2015	57.89	120.53	22.54
1349	DALYs (Disability-Adjusted Life Years)	Kingdom of Saudi Arabia	Female	Age-standardized	Periodontal diseases	Rate		2015	52.85	111.51	21.07
1350	DALYs (Disability-Adjusted Life Years)	Kingdom of Saudi Arabia	Both	Age-standardized	Periodontal diseases	Rate		2015	56.04	117.91	22.00
1351	DALYs	United	Male	Age-standardized	Periodontal	Rate		2012	38.24	78.61	14.26

	(Disability-Adjusted Life Years)	Kingdom of Great Britain and Northern Ireland			diseases					
1352	DALYs (Disability-Adjusted Life Years)	United Kingdom of Great Britain and Northern Ireland	Female	Age-standardized	Periodontal diseases	Rate	2012	40.17	80.48	15.37
1353	DALYs (Disability-Adjusted Life Years)	United Kingdom of Great Britain and Northern Ireland	Both	Age-standardized	Periodontal diseases	Rate	2012	39.21	79.26	14.82
1354	DALYs (Disability-Adjusted Life Years)	Republic of Korea	Male	Age-standardized	Periodontal diseases	Rate	2015	60.04	124.00	23.26
1355	DALYs (Disability-Adjusted Life Years)	Republic of Korea	Female	Age-standardized	Periodontal diseases	Rate	2015	43.04	88.84	15.64
1356	DALYs (Disability-Adjusted Life Years)	Republic of Korea	Both	Age-standardized	Periodontal diseases	Rate	2015	51.41	106.62	19.52
1357	DALYs (Disability-Adjusted Life Years)	United Kingdom of Great Britain and Northern Ireland	Male	Age-standardized	Periodontal diseases	Rate	2011	38.68	79.81	14.37
1358	DALYs (Disability-Adjusted Life Years)	United Kingdom of Great Britain and Northern Ireland	Female	Age-standardized	Periodontal diseases	Rate	2011	40.80	82.23	15.51
1359	DALYs (Disability-Adjusted Life Years)	United Kingdom of Great Britain and Northern Ireland	Both	Age-standardized	Periodontal diseases	Rate	2011	39.75	80.95	14.95
1360	DALYs (Disability-Adjusted Life Years)	Federative Republic of Brazil	Male	Age-standardized	Periodontal diseases	Rate	2015	86.47	185.98	34.60
1361	DALYs (Disability-Adjusted Life Years)	Federative Republic of Brazil	Female	Age-standardized	Periodontal diseases	Rate	2015	75.50	161.43	29.49

1362	DALYs (Disability-Adjusted Life Years)	Federative Republic of Brazil	Both	Age-standardized	Periodontal diseases	Rate		2015	80.73	173.14	31.87
1363	DALYs (Disability-Adjusted Life Years)	Republic of Turkey	Male	Age-standardized	Periodontal diseases	Rate		2011	85.90	172.52	33.31
1364	DALYs (Disability-Adjusted Life Years)	Republic of Turkey	Female	Age-standardized	Periodontal diseases	Rate		2011	79.38	163.28	30.19
1365	DALYs (Disability-Adjusted Life Years)	Republic of Turkey	Both	Age-standardized	Periodontal diseases	Rate		2011	82.59	167.83	31.77
1366	DALYs (Disability-Adjusted Life Years)	European Union	Male	Age-standardized	Periodontal diseases	Rate		2010	70.58	144.41	28.25
1367	DALYs (Disability-Adjusted Life Years)	European Union	Female	Age-standardized	Periodontal diseases	Rate		2010	67.88	137.14	27.01
1368	DALYs (Disability-Adjusted Life Years)	European Union	Both	Age-standardized	Periodontal diseases	Rate		2010	69.17	140.63	27.60
1369	DALYs (Disability-Adjusted Life Years)	United Mexican States	Male	Age-standardized	Periodontal diseases	Rate		2012	103.10	210.62	41.78
1370	DALYs (Disability-Adjusted Life Years)	United Mexican States	Female	Age-standardized	Periodontal diseases	Rate		2012	92.47	192.38	36.95
1371	DALYs (Disability-Adjusted Life Years)	United Mexican States	Both	Age-standardized	Periodontal diseases	Rate		2012	97.51	200.48	39.11

Table S3. Age distribution of periodontal diseases incidence in China

	measure_name	location_name	sex_name	age_name	cause_name	metric_name	year	val	upper	lower	measure_name	location_name	sex_name	age_name	cause_name	metric_name	year	val	upper	lower
1	Incidence	People's Republic of China	Male	95+ years	Periodontal diseases	Number	2023	7457.00	10380.93	3956.11	Incidence	People's Republic of China	Male	95+ years	Periodontal diseases	Rate	2023	1759.95	2450.04	933.70
2	Incidence	People's Republic of China	Female	95+ years	Periodontal diseases	Number	2023	14401.86	20215.22	7588.97	Incidence	People's Republic of China	Female	95+ years	Periodontal diseases	Rate	2023	1760.86	2471.64	927.87
3	Incidence	People's Republic of China	Male	5-9 years	Periodontal diseases	Number	2023	2283.50	4333.09	869.40	Incidence	People's Republic of China	Male	5-9 years	Periodontal diseases	Rate	2023	4.25	8.06	1.62
4	Incidence	People's Republic of China	Female	5-9 years	Periodontal diseases	Number	2023	1960.93	3641.44	753.84	Incidence	People's Republic of China	Female	5-9 years	Periodontal diseases	Rate	2023	4.14	7.68	1.59
5	Incidence	People's Republic of China	Male	10-14 years	Periodontal diseases	Number	2023	13879.71	25893.95	6725.43	Incidence	People's Republic of China	Male	10-14 years	Periodontal diseases	Rate	2023	29.42	54.89	14.26
6	Incidence	People's Republic of China	Female	10-14 years	Periodontal diseases	Number	2023	11675.69	22126.74	5777.42	Incidence	People's Republic of China	Female	10-14 years	Periodontal diseases	Rate	2023	28.07	53.19	13.89
7	Incidence	People's Republic of China	Male	15-19 years	Periodontal diseases	Number	2023	53044.87	88721.71	30853.89	Incidence	People's Republic of China	Male	15-19 years	Periodontal diseases	Rate	2023	123.10	205.89	71.60
8	Incidence	People's Republic of China	Female	15-19 years	Periodontal diseases	Number	2023	43070.03	70867.81	24678.64	Incidence	People's Republic of China	Female	15-19 years	Periodontal diseases	Rate	2023	114.95	189.13	65.86

9	Incidence	People's Republic of China	Male	20-24 years	Periodontal diseases	Number	2023	135415.63	213627.54	79593.23	Incidence	People's Republic of China	Male	20-24 years	Periodontal diseases	Rate	2023	358.15	565.01	210.51
10	Incidence	People's Republic of China	Female	20-24 years	Periodontal diseases	Number	2023	108738.68	172238.70	63318.00	Incidence	People's Republic of China	Female	20-24 years	Periodontal diseases	Rate	2023	330.05	522.78	192.18
11	Incidence	People's Republic of China	Male	25-29 years	Periodontal diseases	Number	2023	311130.23	498923.04	168079.00	Incidence	People's Republic of China	Male	25-29 years	Periodontal diseases	Rate	2023	748.58	1200.41	404.40
12	Incidence	People's Republic of China	Female	25-29 years	Periodontal diseases	Number	2023	250235.83	407473.01	136964.42	Incidence	People's Republic of China	Female	25-29 years	Periodontal diseases	Rate	2023	686.95	1118.59	375.99
13	Incidence	People's Republic of China	Male	30-34 years	Periodontal diseases	Number	2023	687935.95	1074169.85	434443.06	Incidence	People's Republic of China	Male	30-34 years	Periodontal diseases	Rate	2023	1219.18	1903.67	769.93
14	Incidence	People's Republic of China	Female	30-34 years	Periodontal diseases	Number	2023	576284.69	919686.37	352238.92	Incidence	People's Republic of China	Female	30-34 years	Periodontal diseases	Rate	2023	1130.15	1803.60	690.78
15	Incidence	People's Republic of China	Male	35-39 years	Periodontal diseases	Number	2023	1029929.34	1486962.71	638602.78	Incidence	People's Republic of China	Male	35-39 years	Periodontal diseases	Rate	2023	1693.69	2445.27	1050.17
16	Incidence	People's Republic of China	Female	35-39 years	Periodontal diseases	Number	2023	927333.98	1349513.64	569038.88	Incidence	People's Republic of China	Female	35-39 years	Periodontal diseases	Rate	2023	1615.30	2350.68	991.19
17	Incidence	People's Republic of	Male	40-44 years	Periodontal disea	Number	2023	921155.11	1247815.61	581089.33	Incidence	People's Republic of	Male	40-44 years	Periodontal diseases	Rate	2023	1971.80	2671.03	1243.86

		China			ses							China								
18	Incidence	People's Republic of China	Female	40-44 years	Periodontal diseases	Number	2023	857755.99	1183598.69	540646.27	Incidence	People's Republic of China	Female	40-44 years	Periodontal diseases	Rate	2023	1939.33	2676.04	1222.37
19	Incidence	People's Republic of China	Male	45-49 years	Periodontal diseases	Number	2023	1034632.16	1342418.01	676519.50	Incidence	People's Republic of China	Male	45-49 years	Periodontal diseases	Rate	2023	2040.94	2648.08	1334.52
20	Incidence	People's Republic of China	Female	45-49 years	Periodontal diseases	Number	2023	1002229.73	1304102.23	644760.80	Incidence	People's Republic of China	Female	45-49 years	Periodontal diseases	Rate	2023	2043.24	2658.67	1314.47
21	Incidence	People's Republic of China	Male	50-54 years	Periodontal diseases	Number	2023	1219075.90	1566143.45	834194.10	Incidence	People's Republic of China	Male	50-54 years	Periodontal diseases	Rate	2023	2028.49	2605.99	1388.06
22	Incidence	People's Republic of China	Female	50-54 years	Periodontal diseases	Number	2023	1190975.21	1531357.09	825532.66	Incidence	People's Republic of China	Female	50-54 years	Periodontal diseases	Rate	2023	2042.87	2626.72	1416.03
23	Incidence	People's Republic of China	Male	55-59 years	Periodontal diseases	Number	2023	1153736.64	1514398.70	816562.03	Incidence	People's Republic of China	Male	55-59 years	Periodontal diseases	Rate	2023	1959.14	2571.58	1386.59
24	Incidence	People's Republic of China	Female	55-59 years	Periodontal diseases	Number	2023	1170630.37	1531317.44	825786.91	Incidence	People's Republic of China	Female	55-59 years	Periodontal diseases	Rate	2023	1972.99	2580.89	1391.79
25	Incidence	People's Republic of China	Male	60-64 years	Periodontal diseases	Number	2023	770596.95	993390.21	518517.86	Incidence	People's Republic of China	Male	60-64 years	Periodontal diseases	Rate	2023	1901.34	2451.05	1279.37
26	Incidence	People's Repub	Female	60-64 year	Periodontal	Number	202	780079.09	1002804.45	519450.79	Incidence	People's Repub	Female	60-64 yea	Periodontal diseases	Rate	2023	1907.33	2451.90	1270.08

		lic of China		s	diseases		3					lic of China		rs						
27	Incidence	People's Republic of China	Male	65-69 years	Periodontal diseases	Number	2023	714988.60	911956.03	477678.41	Incidence	People's Republic of China	Male	65-69 years	Periodontal diseases	Rate	2023	1896.55	2419.02	1267.07
28	Incidence	People's Republic of China	Female	65-69 years	Periodontal diseases	Number	2023	731501.85	929275.39	482503.14	Incidence	People's Republic of China	Female	65-69 years	Periodontal diseases	Rate	2023	1894.42	2406.61	1249.57
29	Incidence	People's Republic of China	Male	70-74 years	Periodontal diseases	Number	2023	557754.05	722010.36	385240.42	Incidence	People's Republic of China	Male	70-74 years	Periodontal diseases	Rate	2023	1901.76	2461.82	1313.55
30	Incidence	People's Republic of China	Female	70-74 years	Periodontal diseases	Number	2023	601803.24	776605.58	414922.15	Incidence	People's Republic of China	Female	70-74 years	Periodontal diseases	Rate	2023	1896.83	2447.79	1307.80
31	Incidence	People's Republic of China	Male	75-79 years	Periodontal diseases	Number	2023	329166.01	410497.99	232698.91	Incidence	People's Republic of China	Male	75-79 years	Periodontal diseases	Rate	2023	1910.39	2382.42	1350.52
32	Incidence	People's Republic of China	Female	75-79 years	Periodontal diseases	Number	2023	373089.90	465994.68	262557.71	Incidence	People's Republic of China	Female	75-79 years	Periodontal diseases	Rate	2023	1907.68	2382.72	1342.51
33	Incidence	People's Republic of China	Male	<1 year	Periodontal diseases	Number	2023	0.00	0.00	0.00	Incidence	People's Republic of China	Male	<1 year	Periodontal diseases	Rate	2023	0.00	0.00	0.00
34	Incidence	People's Republic of China	Female	<1 year	Periodontal diseases	Number	2023	0.00	0.00	0.00	Incidence	People's Republic of China	Female	<1 year	Periodontal diseases	Rate	2023	0.00	0.00	0.00
35	Incidence	People's	Male	80-84	Periodontal	Number	20	190970.	250944.	132761.	Incidence	People's	Male	80-84	Periodontal	Rate	2023	1897.87	2493.90	1319.39

		Republic of China		years	l diseases		23	36	73	37		Republic of China		years	diseases					
36	Incidence	People's Republic of China	Female	80-84 years	Periodontal diseases	Number	2023	235774.06	307549.07	164267.82	Incidence	People's Republic of China	Female	80-84 years	Periodontal diseases	Rate	2023	1901.08	2479.82	1324.52
37	Incidence	People's Republic of China	Male	85-89 years	Periodontal diseases	Number	2023	95533.28	127826.80	60927.04	Incidence	People's Republic of China	Male	85-89 years	Periodontal diseases	Rate	2023	1870.94	2503.38	1193.21
38	Incidence	People's Republic of China	Female	85-89 years	Periodontal diseases	Number	2023	138315.42	186016.29	88586.29	Incidence	People's Republic of China	Female	85-89 years	Periodontal diseases	Rate	2023	1877.75	2525.33	1202.63
39	Incidence	People's Republic of China	Male	90-94 years	Periodontal diseases	Number	2023	31079.26	41504.39	17932.26	Incidence	People's Republic of China	Male	90-94 years	Periodontal diseases	Rate	2023	1825.60	2437.97	1053.34
40	Incidence	People's Republic of China	Female	90-94 years	Periodontal diseases	Number	2023	51683.39	68856.05	29640.98	Incidence	People's Republic of China	Female	90-94 years	Periodontal diseases	Rate	2023	1828.82	2436.48	1048.85
41	Incidence	People's Republic of China	Male	2-4 years	Periodontal diseases	Number	2023	0.00	0.00	0.00	Incidence	People's Republic of China	Male	2-4 years	Periodontal diseases	Rate	2023	0.00	0.00	0.00
42	Incidence	People's Republic of China	Female	2-4 years	Periodontal diseases	Number	2023	0.00	0.00	0.00	Incidence	People's Republic of China	Female	2-4 years	Periodontal diseases	Rate	2023	0.00	0.00	0.00

Table S4. Age distribution of DALYs due to periodontal diseases in China

	measure_name	location_name	sex_name	age_name	cause_name	metric_name	year	val	upper	lower	measure_name	location_name	sex_name	age_name	cause_name	metric_name	year	val	upper	lower
1	Incidence	G20	Male	95+ years	Periodontal diseases	Number	2023	23247.66	32007.62	13892.41	Incidence	G20	Male	95+ years	Periodontal diseases	Rate	2023	1808.41	2489.84	1080.68
2	Incidence	G20	Female	95+ years	Periodontal diseases	Number	2023	61184.24	84068.19	37560.20	Incidence	G20	Female	95+ years	Periodontal diseases	Rate	2023	1838.42	2526.01	1128.58
3	Incidence	G20	Male	5-9 years	Periodontal diseases	Number	2023	12009.30	23076.51	4922.25	Incidence	G20	Male	5-9 years	Periodontal diseases	Rate	2023	6.75	12.96	2.76
4	Incidence	G20	Female	5-9 years	Periodontal diseases	Number	2023	10977.51	21190.34	4518.70	Incidence	G20	Female	5-9 years	Periodontal diseases	Rate	2023	6.69	12.91	2.75
5	Incidence	G20	Male	10-14 years	Periodontal diseases	Number	2023	121383.41	225084.74	62267.87	Incidence	G20	Male	10-14 years	Periodontal diseases	Rate	2023	68.10	126.28	34.93
6	Incidence	G20	Female	10-14 years	Periodontal diseases	Number	2023	111037.07	206049.34	56242.72	Incidence	G20	Female	10-14 years	Periodontal diseases	Rate	2023	67.41	125.09	34.14
7	Incidence	G20	Male	15-19 years	Periodontal diseases	Number	2023	617759.91	946925.72	365452.47	Incidence	G20	Male	15-19 years	Periodontal diseases	Rate	2023	351.39	538.62	207.87
8	Incidence	G20	Female	15-19	Periodonta	Number	20	565895.	873696.	331284.	Incidence	G20	Female	15-19 years	Periodontal	Rate	2023	349.51	539.61	204.61

				years	l diseases		23	97	96	93					diseases					
9	Incidence	G20	Male	20-24 years	Periodontal diseases	Number	2023	1576446.29	2331650.53	987511.85	Incidence	G20	Male	20-24 years	Periodontal diseases	Rate	2023	924.36	1367.17	579.03
10	Incidence	G20	Female	20-24 years	Periodontal diseases	Number	2023	1470719.80	2140114.66	931099.36	Incidence	G20	Female	20-24 years	Periodontal diseases	Rate	2023	927.30	1349.36	587.07
11	Incidence	G20	Male	25-29 years	Periodontal diseases	Number	2023	2435579.52	3573294.49	1465347.60	Incidence	G20	Male	25-29 years	Periodontal diseases	Rate	2023	1410.44	2069.29	848.58
12	Incidence	G20	Female	25-29 years	Periodontal diseases	Number	2023	2289587.00	3353462.17	1377685.92	Incidence	G20	Female	25-29 years	Periodontal diseases	Rate	2023	1412.91	2069.43	850.17
13	Incidence	G20	Male	30-34 years	Periodontal diseases	Number	2023	3018135.07	4256712.73	1976771.76	Incidence	G20	Male	30-34 years	Periodontal diseases	Rate	2023	1625.35	2292.36	1064.55
14	Incidence	G20	Female	30-34 years	Periodontal diseases	Number	2023	2806402.43	3928680.40	1829521.06	Incidence	G20	Female	30-34 years	Periodontal diseases	Rate	2023	1601.80	2242.36	1044.23
15	Incidence	G20	Male	35-39 years	Periodontal diseases	Number	2023	3399278.28	4736526.37	2206517.74	Incidence	G20	Male	35-39 years	Periodontal diseases	Rate	2023	1825.86	2544.13	1185.19
16	Incidence	G20	Female	35-39 years	Periodontal diseases	Number	2023	3191255.95	4458023.31	2076493.35	Incidence	G20	Female	35-39 years	Periodontal diseases	Rate	2023	1787.54	2497.11	1163.12
1	Incidenc	G2	Mal	40-4	Perio	Num	2	314	418	205	Incidence	G2	Mal	40-44	Periodon	Rate	2023	1930.	2570.	1260.

7	e	0	e	4 years	dontal diseases	ber	023	4676.93	8293.54	4088.47		0	e	years	tal diseases			29	89	86
18	Incidence	G20	Female	40-44 years	Periodontal diseases	Number	2023	2993984.59	3980826.81	1947706.28	Incidence	G20	Female	40-44 years	Periodontal diseases	Rate	2023	1898.78	2524.63	1235.23
19	Incidence	G20	Male	45-49 years	Periodontal diseases	Number	2023	2973635.10	3844141.44	1948144.82	Incidence	G20	Male	45-49 years	Periodontal diseases	Rate	2023	1936.29	2503.12	1268.54
20	Incidence	G20	Female	45-49 years	Periodontal diseases	Number	2023	2895533.87	3738330.07	1903652.68	Incidence	G20	Female	45-49 years	Periodontal diseases	Rate	2023	1918.50	2476.91	1261.31
21	Incidence	G20	Male	50-54 years	Periodontal diseases	Number	2023	2965802.07	3873252.31	1901815.26	Incidence	G20	Male	50-54 years	Periodontal diseases	Rate	2023	1923.33	2511.81	1233.33
22	Incidence	G20	Female	50-54 years	Periodontal diseases	Number	2023	2931119.82	3838613.16	1882844.80	Incidence	G20	Female	50-54 years	Periodontal diseases	Rate	2023	1914.06	2506.67	1229.52
23	Incidence	G20	Male	55-59 years	Periodontal diseases	Number	2023	2655361.56	3484104.93	1812919.55	Incidence	G20	Male	55-59 years	Periodontal diseases	Rate	2023	1867.61	2450.50	1275.09
24	Incidence	G20	Female	55-59 years	Periodontal diseases	Number	2023	2719905.11	3562058.76	1843265.16	Incidence	G20	Female	55-59 years	Periodontal diseases	Rate	2023	1862.97	2439.80	1262.53
25	Incidence	G20	Male	60-64 years	Periodontal diseases	Number	2023	2096512.13	2745313.04	1382943.38	Incidence	G20	Male	60-64 years	Periodontal diseases	Rate	2023	1829.29	2395.40	1206.67

26	Incidence	G20	Female	60-64 years	Periodontal diseases	Number	2023	2195977.84	2880903.05	1445126.89	Incidence	G20	Female	60-64 years	Periodontal diseases	Rate	2023	1824.72	2393.84	1200.81
27	Incidence	G20	Male	65-69 years	Periodontal diseases	Number	2023	1814230.01	2331583.64	1162556.27	Incidence	G20	Male	65-69 years	Periodontal diseases	Rate	2023	1850.93	2378.75	1186.07
28	Incidence	G20	Female	65-69 years	Periodontal diseases	Number	2023	1953237.46	2531972.03	1250529.24	Incidence	G20	Female	65-69 years	Periodontal diseases	Rate	2023	1849.17	2397.07	1183.90
29	Incidence	G20	Male	70-74 years	Periodontal diseases	Number	2023	1420822.69	1833852.23	954167.95	Incidence	G20	Male	70-74 years	Periodontal diseases	Rate	2023	1877.32	2423.05	1260.73
30	Incidence	G20	Female	70-74 years	Periodontal diseases	Number	2023	1609862.92	2079399.71	1073017.19	Incidence	G20	Female	70-74 years	Periodontal diseases	Rate	2023	1878.92	2426.93	1252.35
31	Incidence	G20	Male	75-79 years	Periodontal diseases	Number	2023	912757.89	1179427.36	604812.84	Incidence	G20	Male	75-79 years	Periodontal diseases	Rate	2023	1893.68	2446.93	1254.79
32	Incidence	G20	Female	75-79 years	Periodontal diseases	Number	2023	1092379.06	1408544.46	722773.03	Incidence	G20	Female	75-79 years	Periodontal diseases	Rate	2023	1899.29	2448.99	1256.66
33	Incidence	G20	Male	<1 year	Periodontal diseases	Number	2023	0.00	0.00	0.00	Incidence	G20	Male	<1 year	Periodontal diseases	Rate	2023	0.00	0.00	0.00
34	Incidence	G20	Female	<1 year	Periodontal disea	Number	2023	0.00	0.00	0.00	Incidence	G20	Female	<1 year	Periodontal diseases	Rate	2023	0.00	0.00	0.00

					ses															
35	Incidence	G20	Male	80-84 years	Periodontal diseases	Number	2023	545434.31	721860.50	373339.03	Incidence	G20	Male	80-84 years	Periodontal diseases	Rate	2023	1887.95	2498.63	1292.27
36	Incidence	G20	Female	80-84 years	Periodontal diseases	Number	2023	733844.11	973983.88	488404.98	Incidence	G20	Female	80-84 years	Periodontal diseases	Rate	2023	1896.89	2517.62	1262.46
37	Incidence	G20	Male	85-89 years	Periodontal diseases	Number	2023	274616.03	367563.93	165953.65	Incidence	G20	Male	85-89 years	Periodontal diseases	Rate	2023	1891.74	2532.03	1143.20
38	Incidence	G20	Female	85-89 years	Periodontal diseases	Number	2023	438897.01	587652.27	264980.93	Incidence	G20	Female	85-89 years	Periodontal diseases	Rate	2023	1905.00	2550.66	1150.13
39	Incidence	G20	Male	90-94 years	Periodontal diseases	Number	2023	94174.87	124580.45	54493.53	Incidence	G20	Male	90-94 years	Periodontal diseases	Rate	2023	1873.99	2479.04	1084.37
40	Incidence	G20	Female	90-94 years	Periodontal diseases	Number	2023	186615.84	245223.97	102641.23	Incidence	G20	Female	90-94 years	Periodontal diseases	Rate	2023	1894.60	2489.61	1042.05
41	Incidence	G20	Male	2-4 years	Periodontal diseases	Number	2023	0.00	0.00	0.00	Incidence	G20	Male	2-4 years	Periodontal diseases	Rate	2023	0.00	0.00	0.00
42	Incidence	G20	Female	2-4 years	Periodontal diseases	Number	2023	0.00	0.00	0.00	Incidence	G20	Female	2-4 years	Periodontal diseases	Rate	2023	0.00	0.00	0.00

Table S5. Age distribution of periodontal diseases incidence in G20 countries

	measure_name	location_name	sex_name	age_name	cause_name	metric_name	year	val	upper	lower	measure_name	location_name	sex_name	age_name	cause_name	metric_name	year	val	upper	lower
1	DALYs (Disability-Adjusted Life Years)	People's Republic of China	Male	5-9 years	Periodontal diseases	Number	2023	25.16	63.32	6.08	DALYs (Disability-Adjusted Life Years)	People's Republic of China	Male	5-9 years	Periodontal diseases	Rate	2023	0.05	0.12	0.01
2	DALYs (Disability-Adjusted Life Years)	People's Republic of China	Female	5-9 years	Periodontal diseases	Number	2023	21.64	54.33	5.12	DALYs (Disability-Adjusted Life Years)	People's Republic of China	Female	5-9 years	Periodontal diseases	Rate	2023	0.05	0.11	0.01
3	DALYs (Disability-Adjusted Life Years)	People's Republic of China	Male	10-14 years	Periodontal diseases	Number	2023	231.83	522.61	68.47	DALYs (Disability-Adjusted Life Years)	People's Republic of China	Male	10-14 years	Periodontal diseases	Rate	2023	0.49	1.11	0.15
4	DALYs (Disability-Adjusted Life Years)	People's Republic of China	Female	10-14 years	Periodontal diseases	Number	2023	194.53	447.04	55.25	DALYs (Disability-Adjusted Life Years)	People's Republic of China	Female	10-14 years	Periodontal diseases	Rate	2023	0.47	1.07	0.13
5	DALYs (Disability-Adjusted Life Years)	People's Republic of China	Male	15-19 years	Periodontal diseases	Number	2023	1107.42	2649.17	362.21	DALYs (Disability-Adjusted Life Years)	People's Republic of China	Male	15-19 years	Periodontal diseases	Rate	2023	2.57	6.15	0.84
6	DALYs (Disability-Adjusted Life Years)	People's Republic of China	Female	15-19 years	Periodontal diseases	Number	2023	896.97	2102.50	305.26	DALYs (Disability-Adjusted Life Years)	People's Republic of China	Female	15-19 years	Periodontal diseases	Rate	2023	2.39	5.61	0.81
7	DALYs (Disability-Adjusted Life Years)	People's Republic of China	Male	20-24 years	Periodontal diseases	Number	2023	3529.58	8322.49	1223.21	DALYs (Disability-Adjusted Life Years)	People's Republic of China	Male	20-24 years	Periodontal diseases	Rate	2023	9.34	22.01	3.24
8	DALYs (Disability-Adjusted Life Years)	People's Republic of China	Female	20-24 years	Periodontal diseases	Number	2023	2824.86	6477.15	965.75	DALYs (Disability-Adjusted Life Years)	People's Republic of China	Female	20-24 years	Periodontal diseases	Rate	2023	8.57	19.66	2.93

9	DALYs (Disability-Adjusted Life Years)	People's Republic of China	Male	25-29 years	Periodontal diseases	Number	2023	10310.81	24995.60	3670.30	DALYs (Disability-Adjusted Life Years)	People's Republic of China	Male	25-29 years	Periodontal diseases	Rate	2023	24.81	60.14	8.83
10	DALYs (Disability-Adjusted Life Years)	People's Republic of China	Female	25-29 years	Periodontal diseases	Number	2023	8228.91	20117.94	2910.06	DALYs (Disability-Adjusted Life Years)	People's Republic of China	Female	25-29 years	Periodontal diseases	Rate	2023	22.59	55.23	7.99
11	DALYs (Disability-Adjusted Life Years)	People's Republic of China	Male	30-34 years	Periodontal diseases	Number	2023	28959.10	70611.47	10575.06	DALYs (Disability-Adjusted Life Years)	People's Republic of China	Male	30-34 years	Periodontal diseases	Rate	2023	51.32	125.14	18.74
12	DALYs (Disability-Adjusted Life Years)	People's Republic of China	Female	30-34 years	Periodontal diseases	Number	2023	23801.77	58087.08	8656.83	DALYs (Disability-Adjusted Life Years)	People's Republic of China	Female	30-34 years	Periodontal diseases	Rate	2023	46.68	113.91	16.98
13	DALYs (Disability-Adjusted Life Years)	People's Republic of China	Male	35-39 years	Periodontal diseases	Number	2023	53715.80	116132.13	21543.72	DALYs (Disability-Adjusted Life Years)	People's Republic of China	Male	35-39 years	Periodontal diseases	Rate	2023	88.33	190.98	35.43
14	DALYs (Disability-Adjusted Life Years)	People's Republic of China	Female	35-39 years	Periodontal diseases	Number	2023	46485.09	100924.92	18594.24	DALYs (Disability-Adjusted Life Years)	People's Republic of China	Female	35-39 years	Periodontal diseases	Rate	2023	80.97	175.80	32.39
15	DALYs (Disability-Adjusted Life Years)	People's Republic of China	Male	40-44 years	Periodontal diseases	Number	2023	60899.60	132218.92	24109.62	DALYs (Disability-Adjusted Life Years)	People's Republic of China	Male	40-44 years	Periodontal diseases	Rate	2023	130.36	283.02	51.61
16	DALYs (Disability-Adjusted Life Years)	People's Republic of China	Female	40-44 years	Periodontal diseases	Number	2023	53932.53	117008.43	21278.64	DALYs (Disability-Adjusted Life Years)	People's Republic of China	Female	40-44 years	Periodontal diseases	Rate	2023	121.94	264.55	48.11
17	DALYs (Disability-Adjusted Life Years)	People's Republic of	Male	45-49 years	Periodontal diseases	Number	2023	85849.59	175278.65	35077.74	DALYs (Disability-Adjusted Life Years)	People's Republic of China	Male	45-49 years	Periodontal diseases	Rate	2023	169.35	345.76	69.20

		China																		
18	DALYs (Disability-Adjusted Life Years)	People's Republic of China	Female	45-49 years	Periodontal diseases	Number	2023	78736.73	162801.20	31655.54	DALYs (Disability-Adjusted Life Years)	People's Republic of China	Female	45-49 years	Periodontal diseases	Rate	2023	160.52	331.90	64.54
19	DALYs (Disability-Adjusted Life Years)	People's Republic of China	Male	50-54 years	Periodontal diseases	Number	2023	118268.46	237153.52	46787.64	DALYs (Disability-Adjusted Life Years)	People's Republic of China	Male	50-54 years	Periodontal diseases	Rate	2023	196.79	394.61	77.85
20	DALYs (Disability-Adjusted Life Years)	People's Republic of China	Female	50-54 years	Periodontal diseases	Number	2023	109652.31	220965.57	43441.09	DALYs (Disability-Adjusted Life Years)	People's Republic of China	Female	50-54 years	Periodontal diseases	Rate	2023	188.09	379.02	74.51
21	DALYs (Disability-Adjusted Life Years)	People's Republic of China	Male	55-59 years	Periodontal diseases	Number	2023	122493.27	239177.16	47068.66	DALYs (Disability-Adjusted Life Years)	People's Republic of China	Male	55-59 years	Periodontal diseases	Rate	2023	208.00	406.14	79.93
22	DALYs (Disability-Adjusted Life Years)	People's Republic of China	Female	55-59 years	Periodontal diseases	Number	2023	118255.44	230846.44	44578.60	DALYs (Disability-Adjusted Life Years)	People's Republic of China	Female	55-59 years	Periodontal diseases	Rate	2023	199.31	389.07	75.13
23	DALYs (Disability-Adjusted Life Years)	People's Republic of China	Male	60-64 years	Periodontal diseases	Number	2023	82678.30	156549.91	30384.35	DALYs (Disability-Adjusted Life Years)	People's Republic of China	Male	60-64 years	Periodontal diseases	Rate	2023	204.00	386.26	74.97
24	DALYs (Disability-Adjusted Life Years)	People's Republic of China	Female	60-64 years	Periodontal diseases	Number	2023	79399.94	150065.53	29079.00	DALYs (Disability-Adjusted Life Years)	People's Republic of China	Female	60-64 years	Periodontal diseases	Rate	2023	194.14	366.92	71.10
25	DALYs (Disability-Adjusted Life Years)	People's Republic of China	Male	65-69 years	Periodontal diseases	Number	2023	72718.67	146651.01	27316.56	DALYs (Disability-Adjusted Life Years)	People's Republic of China	Male	65-69 years	Periodontal diseases	Rate	2023	192.89	389.00	72.46
26	DALYs (Disability-Adjusted Life	People's Republi	Female	65-69 year	Periodontal diseases	Number	2023	69673.92	140120.10	25721.50	DALYs (Disability-Adjusted Life	People's Republic of China	Female	65-69 years	Periodontal disease	Rate	2023	180.44	362.88	66.61

	Years)	c of China		s							Years)				s					
27	DALYs (Disability-Adjusted Life Years)	People's Republic of China	Male	70-74 years	Periodontal diseases	Number	2023	51334.70	105418.00	19723.79	DALYs (Disability-Adjusted Life Years)	People's Republic of China	Male	70-74 years	Periodontal diseases	Rate	2023	175.03	359.44	67.25
28	DALYs (Disability-Adjusted Life Years)	People's Republic of China	Female	70-74 years	Periodontal diseases	Number	2023	51031.14	104083.38	19163.28	DALYs (Disability-Adjusted Life Years)	People's Republic of China	Female	70-74 years	Periodontal diseases	Rate	2023	160.85	328.06	60.40
29	DALYs (Disability-Adjusted Life Years)	People's Republic of China	Male	75-79 years	Periodontal diseases	Number	2023	26807.88	54859.64	10321.32	DALYs (Disability-Adjusted Life Years)	People's Republic of China	Male	75-79 years	Periodontal diseases	Rate	2023	155.59	318.39	59.90
30	DALYs (Disability-Adjusted Life Years)	People's Republic of China	Female	75-79 years	Periodontal diseases	Number	2023	27624.00	56522.15	10443.16	DALYs (Disability-Adjusted Life Years)	People's Republic of China	Female	75-79 years	Periodontal diseases	Rate	2023	141.25	289.01	53.40
31	DALYs (Disability-Adjusted Life Years)	People's Republic of China	Male	<1 year	Periodontal diseases	Number	2023	0.00	0.00	0.00	DALYs (Disability-Adjusted Life Years)	People's Republic of China	Male	<1 year	Periodontal diseases	Rate	2023	0.00	0.00	0.00
32	DALYs (Disability-Adjusted Life Years)	People's Republic of China	Female	<1 year	Periodontal diseases	Number	2023	0.00	0.00	0.00	DALYs (Disability-Adjusted Life Years)	People's Republic of China	Female	<1 year	Periodontal diseases	Rate	2023	0.00	0.00	0.00
33	DALYs (Disability-Adjusted Life Years)	People's Republic of China	Male	80-84 years	Periodontal diseases	Number	2023	13602.41	27900.18	5331.56	DALYs (Disability-Adjusted Life Years)	People's Republic of China	Male	80-84 years	Periodontal diseases	Rate	2023	135.18	277.27	52.99
34	DALYs (Disability-Adjusted Life Years)	People's Republic of China	Female	80-84 years	Periodontal diseases	Number	2023	15074.17	30529.05	5797.83	DALYs (Disability-Adjusted Life Years)	People's Republic of China	Female	80-84 years	Periodontal diseases	Rate	2023	121.55	246.16	46.75
35	DALYs (Disability-A	People's	Male	85-89	Periodontal diseases	Numbe	2023	6027.43	12130.5	2333.01	DALYs (Disability-A	People's Republic of	Male	85-89 years	Periodontal	Rate	2023	118.04	237.57	45.69

	djusted Life Years)	Republic of China		years		r			3		djusted Life Years)	China			diseases					
36	DALYs (Disability-Adjusted Life Years)	People's Republic of China	Female	85-89 years	Periodontal diseases	Number	2023	7859.74	16097.59	2961.93	DALYs (Disability-Adjusted Life Years)	People's Republic of China	Female	85-89 years	Periodontal diseases	Rate	2023	106.70	218.54	40.21
37	DALYs (Disability-Adjusted Life Years)	People's Republic of China	Male	90-94 years	Periodontal diseases	Number	2023	1794.83	3702.69	686.46	DALYs (Disability-Adjusted Life Years)	People's Republic of China	Male	90-94 years	Periodontal diseases	Rate	2023	105.43	217.50	40.32
38	DALYs (Disability-Adjusted Life Years)	People's Republic of China	Female	90-94 years	Periodontal diseases	Number	2023	2727.31	5747.63	1037.25	DALYs (Disability-Adjusted Life Years)	People's Republic of China	Female	90-94 years	Periodontal diseases	Rate	2023	96.51	203.38	36.70
39	DALYs (Disability-Adjusted Life Years)	People's Republic of China	Male	2-4 years	Periodontal diseases	Number	2023	0.00	0.00	0.00	DALYs (Disability-Adjusted Life Years)	People's Republic of China	Male	2-4 years	Periodontal diseases	Rate	2023	0.00	0.00	0.00
40	DALYs (Disability-Adjusted Life Years)	People's Republic of China	Female	2-4 years	Periodontal diseases	Number	2023	0.00	0.00	0.00	DALYs (Disability-Adjusted Life Years)	People's Republic of China	Female	2-4 years	Periodontal diseases	Rate	2023	0.00	0.00	0.00
41	DALYs (Disability-Adjusted Life Years)	People's Republic of China	Male	95+ years	Periodontal diseases	Number	2023	401.13	830.10	149.46	DALYs (Disability-Adjusted Life Years)	People's Republic of China	Male	95+ years	Periodontal diseases	Rate	2023	94.67	195.92	35.28
42	DALYs (Disability-Adjusted Life Years)	People's Republic of China	Female	95+ years	Periodontal diseases	Number	2023	714.78	1452.23	269.09	DALYs (Disability-Adjusted Life Years)	People's Republic of China	Female	95+ years	Periodontal diseases	Rate	2023	87.39	177.56	32.90

Table S6. Age distribution of DALYs due to periodontal diseases in G20 countries

	measure_name	location_name	sex_name	age_name	cause_name	metric_name	year	val	upper	lower	measure_name	location_name	sex_name	age_name	cause_name	metric_name	year	val	upper	lower
1	DALYs (Disability-Adjusted Life Years)	G20	Male	5-9 years	Periodontal diseases	Number	2023	130.88	343.04	34.78	DALYs (Disability-Adjusted Life Years)	G20	Male	5-9 years	Periodontal diseases	Rate	2023	0.07	0.19	0.02
2	DALYs (Disability-Adjusted Life Years)	G20	Female	5-9 years	Periodontal diseases	Number	2023	119.44	310.27	26.81	DALYs (Disability-Adjusted Life Years)	G20	Female	5-9 years	Periodontal diseases	Rate	2023	0.07	0.19	0.02
3	DALYs (Disability-Adjusted Life Years)	G20	Male	10-14 years	Periodontal diseases	Number	2023	1796.83	4345.40	540.27	DALYs (Disability-Adjusted Life Years)	G20	Male	10-14 years	Periodontal diseases	Rate	2023	1.01	2.44	0.30
4	DALYs (Disability-Adjusted Life Years)	G20	Female	10-14 years	Periodontal diseases	Number	2023	1637.67	3995.86	505.90	DALYs (Disability-Adjusted Life Years)	G20	Female	10-14 years	Periodontal diseases	Rate	2023	0.99	2.43	0.31
5	DALYs (Disability-Adjusted Life Years)	G20	Male	15-19 years	Periodontal diseases	Number	2023	11600.81	25450.94	3809.31	DALYs (Disability-Adjusted Life Years)	G20	Male	15-19 years	Periodontal diseases	Rate	2023	6.60	14.48	2.17
6	DALYs (Disability-Adjusted Life Years)	G20	Female	15-19 years	Periodontal diseases	Number	2023	10542.56	23072.80	3556.01	DALYs (Disability-Adjusted Life Years)	G20	Female	15-19 years	Periodontal diseases	Rate	2023	6.51	14.25	2.20
7	DALYs (Disability-Adjusted Life Years)	G20	Male	20-24 years	Periodontal diseases	Number	2023	42737.42	98453.79	14935.94	DALYs (Disability-Adjusted Life Years)	G20	Male	20-24 years	Periodontal diseases	Rate	2023	25.06	57.73	8.76
8	DALYs (Disability-Adjusted Life Years)	G20	Female	20-24 years	Periodontal diseases	Number	2023	39361.20	90371.79	13904.68	DALYs (Disability-Adjusted Life Years)	G20	Female	20-24 years	Periodontal diseases	Rate	2023	24.82	56.98	8.77
9	DALYs (Disability-Adjusted Life Years)	G20	Male	25-29 years	Periodontal diseases	Number	2023	98470.69	225469.73	35851.20	DALYs (Disability-Adjusted Life Years)	G20	Male	25-29 years	Periodontal diseases	Rate	2023	57.02	130.57	20.76
10	DALYs (Disability-Adjusted	G20	Female	25-29 years	Periodontal disease	Number	2023	91691.96	210581.57	33341.55	DALYs (Disability-Adjusted	G20	Female	25-29 years	Periodontal diseases	Rate	2023	56.58	129.95	20.58

	Life Years)				s						Life Years)									
11	DALYs (Disability-Adjusted Life Years)	G20	Male	30-34 years	Periodontal diseases	Number	2023	168857.86	385644.41	67281.14	DALYs (Disability-Adjusted Life Years)	G20	Male	30-34 years	Periodontal diseases	Rate	2023	90.93	207.68	36.23
12	DALYs (Disability-Adjusted Life Years)	G20	Female	30-34 years	Periodontal diseases	Number	2023	155772.27	351439.46	62172.81	DALYs (Disability-Adjusted Life Years)	G20	Female	30-34 years	Periodontal diseases	Rate	2023	88.91	200.59	35.49
13	DALYs (Disability-Adjusted Life Years)	G20	Male	35-39 years	Periodontal diseases	Number	2023	237314.36	500152.88	94043.60	DALYs (Disability-Adjusted Life Years)	G20	Male	35-39 years	Periodontal diseases	Rate	2023	127.47	268.65	50.51
14	DALYs (Disability-Adjusted Life Years)	G20	Female	35-39 years	Periodontal diseases	Number	2023	219419.85	460158.80	87221.77	DALYs (Disability-Adjusted Life Years)	G20	Female	35-39 years	Periodontal diseases	Rate	2023	122.91	257.75	48.86
15	DALYs (Disability-Adjusted Life Years)	G20	Male	40-44 years	Periodontal diseases	Number	2023	267938.01	560014.75	106723.87	DALYs (Disability-Adjusted Life Years)	G20	Male	40-44 years	Periodontal diseases	Rate	2023	164.47	343.75	65.51
16	DALYs (Disability-Adjusted Life Years)	G20	Female	40-44 years	Periodontal diseases	Number	2023	249423.07	520293.08	100032.12	DALYs (Disability-Adjusted Life Years)	G20	Female	40-44 years	Periodontal diseases	Rate	2023	158.18	329.97	63.44
17	DALYs (Disability-Adjusted Life Years)	G20	Male	45-49 years	Periodontal diseases	Number	2023	291463.02	590295.06	115572.78	DALYs (Disability-Adjusted Life Years)	G20	Male	45-49 years	Periodontal diseases	Rate	2023	189.79	384.37	75.26
18	DALYs (Disability-Adjusted Life Years)	G20	Female	45-49 years	Periodontal diseases	Number	2023	274590.55	552744.08	109539.18	DALYs (Disability-Adjusted Life Years)	G20	Female	45-49 years	Periodontal diseases	Rate	2023	181.94	366.23	72.58
19	DALYs (Disability-Adjusted Life Years)	G20	Male	50-54 years	Periodontal diseases	Number	2023	314882.87	611472.09	122011.38	DALYs (Disability-Adjusted Life Years)	G20	Male	50-54 years	Periodontal diseases	Rate	2023	204.20	396.54	79.12
20	DALYs (Disability-Adjusted Life Years)	G20	Female	50-54 years	Periodontal diseases	Number	2023	299222.53	588025.29	116475.11	DALYs (Disability-Adjusted Life Years)	G20	Female	50-54 years	Periodontal diseases	Rate	2023	195.40	383.99	76.06
21	DALYs (Disability-Adjusted	G20	Male	55-59 years	Periodontal disease	Number	2023	293994.43	552861.75	111261.54	DALYs (Disability-Adjusted	G20	Male	55-59 years	Periodontal diseases	Rate	2023	206.78	388.85	78.25

	Life Years)				s						Life Years)									
22	DALYs (Disability-Adjusted Life Years)	G20	Female	55-59 years	Periodontal diseases	Number	2023	288313.42	543133.02	108890.26	DALYs (Disability-Adjusted Life Years)	G20	Female	55-59 years	Periodontal diseases	Rate	2023	197.48	372.01	74.58
23	DALYs (Disability-Adjusted Life Years)	G20	Male	60-64 years	Periodontal diseases	Number	2023	225831.14	427212.83	84838.91	DALYs (Disability-Adjusted Life Years)	G20	Male	60-64 years	Periodontal diseases	Rate	2023	197.05	372.76	74.03
24	DALYs (Disability-Adjusted Life Years)	G20	Female	60-64 years	Periodontal diseases	Number	2023	224072.08	423252.51	83759.19	DALYs (Disability-Adjusted Life Years)	G20	Female	60-64 years	Periodontal diseases	Rate	2023	186.19	351.70	69.60
25	DALYs (Disability-Adjusted Life Years)	G20	Male	65-69 years	Periodontal diseases	Number	2023	180079.40	364735.09	66876.01	DALYs (Disability-Adjusted Life Years)	G20	Male	65-69 years	Periodontal diseases	Rate	2023	183.72	372.11	68.23
26	DALYs (Disability-Adjusted Life Years)	G20	Female	65-69 years	Periodontal diseases	Number	2023	180539.43	363911.33	66797.88	DALYs (Disability-Adjusted Life Years)	G20	Female	65-69 years	Periodontal diseases	Rate	2023	170.92	344.52	63.24
27	DALYs (Disability-Adjusted Life Years)	G20	Male	70-74 years	Periodontal diseases	Number	2023	126635.93	262239.82	48444.98	DALYs (Disability-Adjusted Life Years)	G20	Male	70-74 years	Periodontal diseases	Rate	2023	167.32	346.49	64.01
28	DALYs (Disability-Adjusted Life Years)	G20	Female	70-74 years	Periodontal diseases	Number	2023	131414.96	269246.49	49821.35	DALYs (Disability-Adjusted Life Years)	G20	Female	70-74 years	Periodontal diseases	Rate	2023	153.38	314.25	58.15
29	DALYs (Disability-Adjusted Life Years)	G20	Male	75-79 years	Periodontal diseases	Number	2023	73046.05	151254.78	27992.21	DALYs (Disability-Adjusted Life Years)	G20	Male	75-79 years	Periodontal diseases	Rate	2023	151.55	313.80	58.07
30	DALYs (Disability-Adjusted Life Years)	G20	Female	75-79 years	Periodontal diseases	Number	2023	79090.73	163222.15	30355.57	DALYs (Disability-Adjusted Life Years)	G20	Female	75-79 years	Periodontal diseases	Rate	2023	137.51	283.79	52.78
31	DALYs (Disability-Adjusted Life Years)	G20	Male	<1 year	Periodontal diseases	Number	2023	0.00	0.00	0.00	DALYs (Disability-Adjusted Life Years)	G20	Male	<1 year	Periodontal diseases	Rate	2023	0.00	0.00	0.00
32	DALYs (Disability-Adjusted	G20	Female	<1 year	Periodontal disease	Number	2023	0.00	0.00	0.00	DALYs (Disability-Adjusted	G20	Female	<1 year	Periodontal diseases	Rate	2023	0.00	0.00	0.00

	Life Years)				s						Life Years)									
33	DALYs (Disability-Adjusted Life Years)	G20	Male	80-84 years	Periodontal diseases	Number	2023	39565.06	79559.72	14968.05	DALYs (Disability-Adjusted Life Years)	G20	Male	80-84 years	Periodontal diseases	Rate	2023	136.95	275.39	51.81
34	DALYs (Disability-Adjusted Life Years)	G20	Female	80-84 years	Periodontal diseases	Number	2023	47766.24	95852.28	18212.61	DALYs (Disability-Adjusted Life Years)	G20	Female	80-84 years	Periodontal diseases	Rate	2023	123.47	247.77	47.08
35	DALYs (Disability-Adjusted Life Years)	G20	Male	85-89 years	Periodontal diseases	Number	2023	18040.62	35708.74	6623.68	DALYs (Disability-Adjusted Life Years)	G20	Male	85-89 years	Periodontal diseases	Rate	2023	124.28	245.99	45.63
36	DALYs (Disability-Adjusted Life Years)	G20	Female	85-89 years	Periodontal diseases	Number	2023	25986.68	51305.50	9643.72	DALYs (Disability-Adjusted Life Years)	G20	Female	85-89 years	Periodontal diseases	Rate	2023	112.79	222.69	41.86
37	DALYs (Disability-Adjusted Life Years)	G20	Male	90-94 years	Periodontal diseases	Number	2023	5825.68	11664.82	2155.63	DALYs (Disability-Adjusted Life Years)	G20	Male	90-94 years	Periodontal diseases	Rate	2023	115.93	232.12	42.90
38	DALYs (Disability-Adjusted Life Years)	G20	Female	90-94 years	Periodontal diseases	Number	2023	10600.83	20863.63	3842.25	DALYs (Disability-Adjusted Life Years)	G20	Female	90-94 years	Periodontal diseases	Rate	2023	107.62	211.82	39.01
39	DALYs (Disability-Adjusted Life Years)	G20	Male	2-4 years	Periodontal diseases	Number	2023	0.00	0.00	0.00	DALYs (Disability-Adjusted Life Years)	G20	Male	2-4 years	Periodontal diseases	Rate	2023	0.00	0.00	0.00
40	DALYs (Disability-Adjusted Life Years)	G20	Female	2-4 years	Periodontal diseases	Number	2023	0.00	0.00	0.00	DALYs (Disability-Adjusted Life Years)	G20	Female	2-4 years	Periodontal diseases	Rate	2023	0.00	0.00	0.00
41	DALYs (Disability-Adjusted Life Years)	G20	Male	95+ years	Periodontal diseases	Number	2023	1386.44	2841.60	503.55	DALYs (Disability-Adjusted Life Years)	G20	Male	95+ years	Periodontal diseases	Rate	2023	107.85	221.05	39.17
42	DALYs (Disability-Adjusted Life Years)	G20	Female	95+ years	Periodontal diseases	Number	2023	3487.78	7005.50	1270.74	DALYs (Disability-Adjusted Life Years)	G20	Female	95+ years	Periodontal diseases	Rate	2023	104.80	210.50	38.18

Table S7. Decomposition analysis of temporal changes in periodontal diseases incidence in China

	sex_name	Overll difference	Aging	Population	Epidemiological change	a_percent	p_percent	r_percent	val_1990	val_2023	diffl
1	Both	7172577.85	4814531.167	3168655.483	-810608.805	67.12	44.18	-11.3	10678710	18327304	7648594
2	Male	3406276.28	2326334.399	1567657.772	-487715.894	68.3	46.02	-14.32	5607145	9259765	3652620
3	Female	3768868.9	2493428.631	1600501.103	-325060.835	66.16	42.47	-8.62	5071566	9067540	3995974

Table S8. Decomposition analysis of temporal changes in DALYs due to periodontal diseases in China

	sex_name	Overll difference	Aging	Population	Epidemiological change	a_percent	p_percent	r_percent	val_1990	val_2023	diff1
1	Both	30431875.99	-9200255.457	36781654.35	2850477.092	-30.23	120.87	9.37	33984056	60360281	26376225
2	Male	14454058.34	-4997087.293	18342606.19	1108539.441	-34.57	126.9	7.67	17296967	30101863	12804896
3	Female	15890068.17	-4321445.727	18402884.21	1808629.683	-27.2	115.81	11.38	16687089	30258418	13571328

Table S9. Decomposition analysis of temporal changes in periodontal diseases incidence in G20 countries

	sex_name	Overll difference	Aging	Population	Epidemiological change	a_percent	p_percent	r_percent	val_1990	val_2023	diff1
1	Both	640472.47	425125.051	265318.218	-49970.803	66.38	41.43	-7.8	722991.8	1437891.8	714900
2	Male	319776.03	216669.539	134928.957	-31822.463	67.76	42.19	-9.95	386035.5	740756	354720.4
3	Female	321545.9	209822.256	130349.798	-18626.154	65.25	40.54	-5.79	336956.2	697135.8	360179.6

Table S10. Decomposition analysis of temporal changes in DALYs due to periodontal diseases in G20 countries

	sex_name	Overll difference	Aging	Population	Epidemiological change	a_percent	p_percent	r_percent	val_1990	val_2023	diff1
1	Both	30431875.99	-9200255.457	36781654.35	2850477.092	-30.23	120.87	9.37	33984056	60360281	26376225
2	Male	14454058.34	-4997087.293	18342606.19	1108539.441	-34.57	126.9	7.67	17296967	30101863	12804896
3	Female	15890068.17	-4321445.727	18402884.21	1808629.683	-27.2	115.81	11.38	16687089	30258418	13571328

Table S11. ARIMA model forecasts of periodontal diseases incidence in China

	Year	Value	Lower	Upper	Type
1	1990	1019.56			Actual
2	1991	978.67			Actual
3	1992	941.31			Actual
4	1993	909.64			Actual
5	1994	885.72			Actual
6	1995	871.74			Actual
7	1996	864.36			Actual
8	1997	858.68			Actual
9	1998	854.73			Actual
10	1999	852.43			Actual
11	2000	851.82			Actual
12	2001	865.94			Actual
13	2002	899.10			Actual
14	2003	938.27			Actual
15	2004	970.42			Actual
16	2005	982.58			Actual
17	2006	963.05			Actual
18	2007	919.60			Actual
19	2008	868.47			Actual
20	2009	825.85			Actual
21	2010	807.91			Actual
22	2011	827.25			Actual
23	2012	873.76			Actual
24	2013	929.44			Actual
25	2014	976.36			Actual
26	2015	996.66			Actual
27	2016	994.85			Actual
28	2017	989.42			Actual
29	2018	983.42			Actual
30	2019	979.88			Actual
31	2020	977.53			Actual
32	2021	979.30			Actual
33	2022	978.92			Actual
34	2023	959.68			Actual
35	2024	919.33	696.30	936.87	Forecast
36	2025	869.35	716.84	978.58	Forecast
37	2026	827.44	756.98	1022.50	Forecast
38	2027	808.12	796.86	1062.89	Forecast
39	2028	816.58	822.17	1095.78	Forecast
40	2029	847.71	829.91	1117.44	Forecast
41	2030	889.74	908.40	930.25	Forecast
42	2031	929.87	834.81	903.90	Forecast

43	2032	958.98	760.97	893.90	Forecast
44	2033	973.68	710.35	905.89	Forecast

Table S12. ARIMA model forecasts of periodontal diseases incidence among males in China

	Year	Value	Lower	Upper	Type
1	1990	1019.56			Actual
2	1991	978.67			Actual
3	1992	941.31			Actual
4	1993	909.64			Actual
5	1994	885.72			Actual
6	1995	871.74			Actual
7	1996	864.36			Actual
8	1997	858.68			Actual
9	1998	854.73			Actual
10	1999	852.43			Actual
11	2000	851.82			Actual
12	2001	865.94			Actual
13	2002	899.10			Actual
14	2003	938.27			Actual
15	2004	970.42			Actual
16	2005	982.58			Actual
17	2006	963.05			Actual
18	2007	919.60			Actual
19	2008	868.47			Actual
20	2009	825.85			Actual
21	2010	807.91			Actual
22	2011	827.25			Actual
23	2012	873.76			Actual
24	2013	929.44			Actual
25	2014	976.36			Actual
26	2015	996.66			Actual
27	2016	994.85			Actual
28	2017	989.42			Actual
29	2018	983.42			Actual
30	2019	979.88			Actual
31	2020	977.53			Actual
32	2021	979.30			Actual
33	2022	978.92			Actual
34	2023	959.68			Actual
35	2024	919.33	696.30	936.87	Forecast
36	2025	869.35	716.84	978.58	Forecast
37	2026	827.44	756.98	1022.50	Forecast
38	2027	808.12	796.86	1062.89	Forecast
39	2028	816.58	822.17	1095.78	Forecast
40	2029	847.71	829.91	1117.44	Forecast
41	2030	889.74	908.40	930.25	Forecast
42	2031	929.87	834.81	903.90	Forecast

43	2032	958.98	760.97	893.90	Forecast
44	2033	973.68	710.35	905.89	Forecast

Table S13. ARIMA model forecasts of periodontal diseases incidence among females in China

	Year	Value	Lower	Upper	Type
1	1990	1001.09			Actual
2	1991	961.03			Actual
3	1992	924.48			Actual
4	1993	893.53			Actual
5	1994	870.15			Actual
6	1995	856.48			Actual
7	1996	849.38			Actual
8	1997	844.16			Actual
9	1998	840.72			Actual
10	1999	838.84			Actual
11	2000	838.45			Actual
12	2001	851.32			Actual
13	2002	881.41			Actual
14	2003	916.88			Actual
15	2004	945.87			Actual
16	2005	956.65			Actual
17	2006	938.06			Actual
18	2007	896.98			Actual
19	2008	848.74			Actual
20	2009	808.59			Actual
21	2010	791.78			Actual
22	2011	811.80			Actual
23	2012	859.65			Actual
24	2013	916.87			Actual
25	2014	965.05			Actual
26	2015	985.82			Actual
27	2016	983.77			Actual
28	2017	977.89			Actual
29	2018	971.45			Actual
30	2019	967.73			Actual
31	2020	967.78			Actual
32	2021	970.06			Actual
33	2022	969.13			Actual
34	2023	950.20			Actual
35	2024	914.79	737.39	944.01	Forecast
36	2025	878.90	743.24	965.43	Forecast
37	2026	852.54	763.53	989.51	Forecast
38	2027	839.50	789.21	1015.20	Forecast
39	2028	840.70	811.46	1041.09	Forecast
40	2029	854.34	824.85	1064.11	Forecast
41	2030	876.52	903.71	925.86	Forecast
42	2031	902.20	843.36	914.44	Forecast

43	2032	926.27	788.95	916.12	Forecast
44	2033	944.48	752.16	926.83	Forecast

Table S14. ARIMA model forecasts of periodontal diseases incidence in G20 countries

	Year	Value	Lower	Upper	Type
1	1990	1071.11			Actual
2	1991	1059.27			Actual
3	1992	1048.73			Actual
4	1993	1040.01			Actual
5	1994	1033.98			Actual
6	1995	1031.18			Actual
7	1996	1031.35			Actual
8	1997	1033.25			Actual
9	1998	1035.99			Actual
10	1999	1038.87			Actual
11	2000	1040.84			Actual
12	2001	1045.27			Actual
13	2002	1054.18			Actual
14	2003	1064.43			Actual
15	2004	1072.85			Actual
16	2005	1076.43			Actual
17	2006	1070.71			Actual
18	2007	1057.25			Actual
19	2008	1041.71			Actual
20	2009	1029.61			Actual
21	2010	1026.40			Actual
22	2011	1038.53			Actual
23	2012	1063.11			Actual
24	2013	1091.51			Actual
25	2014	1115.29			Actual
26	2015	1126.31			Actual
27	2016	1124.63			Actual
28	2017	1118.50			Actual
29	2018	1112.14			Actual
30	2019	1109.74			Actual
31	2020	1109.82			Actual
32	2021	1110.02			Actual
33	2022	1110.61			Actual
34	2023	1093.38			Actual
35	2024	1054.66	878.02	1069.68	Forecast
36	2025	1013.24	874.75	1100.23	Forecast
37	2026	983.10	879.12	1128.67	Forecast
38	2027	970.39	883.91	1150.12	Forecast
39	2028	973.85	884.51	1162.86	Forecast
40	2029	987.49	879.54	1167.94	Forecast
41	2030	1003.90	1047.24	1062.08	Forecast
42	2031	1017.02	987.92	1038.56	Forecast

43	2032	1023.68	933.89	1032.31	Forecast
44	2033	1023.74	896.36	1044.41	Forecast

Table S15. ARIMA model forecasts of periodontal diseases incidence among males in G20 countries

	Year	Value	Lower	Upper	Type
1	1990	1030.73			Actual
2	1991	1021.72			Actual
3	1992	1014.07			Actual
4	1993	1007.96			Actual
5	1994	1003.95			Actual
6	1995	1002.25			Actual
7	1996	1003.36			Actual
8	1997	1006.66			Actual
9	1998	1010.97			Actual
10	1999	1015.42			Actual
11	2000	1018.83			Actual
12	2001	1022.53			Actual
13	2002	1027.72			Actual
14	2003	1033.18			Actual
15	2004	1037.54			Actual
16	2005	1039.43			Actual
17	2006	1034.97			Actual
18	2007	1024.33			Actual
19	2008	1012.04			Actual
20	2009	1002.58			Actual
21	2010	1000.53			Actual
22	2011	1012.42			Actual
23	2012	1036.25			Actual
24	2013	1063.74			Actual
25	2014	1086.79			Actual
26	2015	1097.68			Actual
27	2016	1098.24			Actual
28	2017	1096.82			Actual
29	2018	1095.50			Actual
30	2019	1096.23			Actual
31	2020	1098.33			Actual
32	2021	1100.63			Actual
33	2022	1102.19			Actual
34	2023	1085.82			Actual
35	2024	1048.50	875.68	1055.31	Forecast
36	2025	1008.03	871.15	1083.96	Forecast
37	2026	977.74	874.68	1111.60	Forecast
38	2027	963.80	879.48	1133.33	Forecast
39	2028	965.50	880.92	1147.09	Forecast
40	2029	977.56	877.25	1153.41	Forecast
41	2030	993.14	1041.85	1055.15	Forecast

42	2031	1006.41	984.91	1031.14	Forecast
43	2032	1014.00	932.36	1023.11	Forecast
44	2033	1015.33	894.96	1032.64	Forecast

Table S16. ARIMA model forecasts of periodontal diseases incidence among females in G20 countries

	Year	Value	Lower	Upper	Type
1	1990	1051.57			Actual
2	1991	1041.11			Actual
3	1992	1031.98			Actual
4	1993	1024.52			Actual
5	1994	1019.47			Actual
6	1995	1017.19			Actual
7	1996	1017.79			Actual
8	1997	1020.33			Actual
9	1998	1023.80			Actual
10	1999	1027.41			Actual
11	2000	1030.06			Actual
12	2001	1034.12			Actual
13	2002	1041.20			Actual
14	2003	1049.07			Actual
15	2004	1055.48			Actual
16	2005	1058.22			Actual
17	2006	1053.12			Actual
18	2007	1041.03			Actual
19	2008	1027.09			Actual
20	2009	1016.29			Actual
21	2010	1013.65			Actual
22	2011	1025.67			Actual
23	2012	1049.91			Actual
24	2013	1077.90			Actual
25	2014	1101.36			Actual
26	2015	1112.33			Actual
27	2016	1111.76			Actual
28	2017	1107.92			Actual
29	2018	1104.02			Actual
30	2019	1103.14			Actual
31	2020	1104.21			Actual
32	2021	1105.44			Actual
33	2022	1106.51			Actual
34	2023	1089.70			Actual
35	2024	1051.66	877.18	1062.37	Forecast
36	2025	1010.65	873.59	1092.12	Forecast
37	2026	980.37	877.88	1120.33	Forecast
38	2027	967.06	882.97	1142.01	Forecast
39	2028	969.78	884.16	1155.22	Forecast
40	2029	982.86	879.87	1160.76	Forecast
41	2030	999.10	1044.64	1058.68	Forecast

42	2031	1012.49	986.50	1034.81	Forecast
43	2032	1019.69	933.20	1027.55	Forecast
44	2033	1020.32	895.80	1038.32	Forecast

Table S17. ARIMA model forecasts of DALYs due to periodontal diseases in China

	Year	Value	Lower	Upper	Type
1	1990	74.47			Actual
2	1991	70.46			Actual
3	1992	66.78			Actual
4	1993	63.66			Actual
5	1994	61.31			Actual
6	1995	59.95			Actual
7	1996	59.23			Actual
8	1997	58.65			Actual
9	1998	58.25			Actual
10	1999	58.02			Actual
11	2000	57.97			Actual
12	2001	59.52			Actual
13	2002	63.11			Actual
14	2003	67.37			Actual
15	2004	70.93			Actual
16	2005	72.40			Actual
17	2006	70.45			Actual
18	2007	65.90			Actual
19	2008	60.49			Actual
20	2009	55.96			Actual
21	2010	54.04			Actual
22	2011	56.27			Actual
23	2012	61.61			Actual
24	2013	67.99			Actual
25	2014	73.35			Actual
26	2015	75.64			Actual
27	2016	75.10			Actual
28	2017	73.85			Actual
29	2018	72.54			Actual
30	2019	71.86			Actual
31	2020	71.58			Actual
32	2021	71.88			Actual
33	2022	72.13			Actual
34	2023	70.97			Actual
35	2024	68.47	47.61	78.04	Forecast
36	2025	65.65	46.27	82.05	Forecast
37	2026	63.46	46.29	85.53	Forecast
38	2027	62.48	46.85	88.05	Forecast
39	2028	62.82	47.18	89.45	Forecast
40	2029	64.16	46.87	89.84	Forecast
41	2030	65.91	67.28	69.67	Forecast
42	2031	67.45	61.74	69.57	Forecast

43	2032	68.32	55.80	71.11	Forecast
44	2033	68.36	50.82	74.14	Forecast

Table S18. ARIMA model forecasts of DALYs due to periodontal diseases among males in China

	Year	Value	Lower	Upper	Type
1	1990	68.69			Actual
2	1991	64.87			Actual
3	1992	61.45			Actual
4	1993	58.61			Actual
5	1994	56.49			Actual
6	1995	55.31			Actual
7	1996	54.84			Actual
8	1997	54.69			Actual
9	1998	54.75			Actual
10	1999	54.90			Actual
11	2000	55.06			Actual
12	2001	56.22			Actual
13	2002	58.78			Actual
14	2003	61.78			Actual
15	2004	64.23			Actual
16	2005	65.20			Actual
17	2006	63.54			Actual
18	2007	59.76			Actual
19	2008	55.33			Actual
20	2009	51.62			Actual
21	2010	50.08			Actual
22	2011	52.32			Actual
23	2012	57.61			Actual
24	2013	63.92			Actual
25	2014	69.21			Actual
26	2015	71.48			Actual
27	2016	71.00			Actual
28	2017	69.85			Actual
29	2018	68.64			Actual
30	2019	68.00			Actual
31	2020	67.67			Actual
32	2021	67.98			Actual
33	2022	67.66			Actual
34	2023	66.54			Actual
35	2024	64.96	47.86	77.30	Forecast
36	2025	63.45	45.64	81.02	Forecast
37	2026	62.46	44.29	83.90	Forecast
38	2027	62.20	43.38	85.81	Forecast
39	2028	62.58	42.54	86.87	Forecast
40	2029	63.33	41.54	87.38	Forecast
41	2030	64.10	63.83	66.09	Forecast
42	2031	64.60	59.86	67.04	Forecast

43	2032	64.70	55.35	69.57	Forecast
44	2033	64.46	51.15	73.24	Forecast

Table S19. ARIMA model forecasts of DALYs due to periodontal diseases among females in China

	Year	Value	Lower	Upper	Type
1	1990	71.61			Actual
2	1991	67.69			Actual
3	1992	64.13			Actual
4	1993	61.15			Actual
5	1994	58.91			Actual
6	1995	57.64			Actual
7	1996	57.06			Actual
8	1997	56.71			Actual
9	1998	56.55			Actual
10	1999	56.51			Actual
11	2000	56.57			Actual
12	2001	57.93			Actual
13	2002	61.00			Actual
14	2003	64.63			Actual
15	2004	67.64			Actual
16	2005	68.85			Actual
17	2006	67.04			Actual
18	2007	62.86			Actual
19	2008	57.93			Actual
20	2009	53.80			Actual
21	2010	52.07			Actual
22	2011	54.30			Actual
23	2012	59.61			Actual
24	2013	65.96			Actual
25	2014	71.29			Actual
26	2015	73.56			Actual
27	2016	73.05			Actual
28	2017	71.85			Actual
29	2018	70.59			Actual
30	2019	69.93			Actual
31	2020	69.62			Actual
32	2021	69.93			Actual
33	2022	69.90			Actual
34	2023	68.76			Actual
35	2024	66.61	45.51	76.54	Forecast
36	2025	64.11	43.16	80.69	Forecast
37	2026	62.04	42.05	84.26	Forecast
38	2027	60.97	41.50	86.86	Forecast
39	2028	61.02	40.91	88.39	Forecast
40	2029	61.93	39.90	89.06	Forecast
41	2030	63.16	65.47	67.74	Forecast

42	2031	64.18	60.45	67.76	Forecast
43	2032	64.65	54.70	69.38	Forecast
44	2033	64.48	49.44	72.50	Forecast

Table S20. ARIMA model forecasts of DALYs due to periodontal diseases in G20 countries

	Year	Value	Lower	Upper	Type
1	1990	79.67			Actual
2	1991	78.89			Actual
3	1992	78.15			Actual
4	1993	77.51			Actual
5	1994	77.03			Actual
6	1995	76.78			Actual
7	1996	76.73			Actual
8	1997	76.76			Actual
9	1998	76.85			Actual
10	1999	76.92			Actual
11	2000	76.95			Actual
12	2001	77.19			Actual
13	2002	77.83			Actual
14	2003	78.62			Actual
15	2004	79.31			Actual
16	2005	79.63			Actual
17	2006	79.05			Actual
18	2007	77.64			Actual
19	2008	75.95			Actual
20	2009	74.60			Actual
21	2010	74.17			Actual
22	2011	75.54			Actual
23	2012	78.46			Actual
24	2013	81.89			Actual
25	2014	84.81			Actual
26	2015	86.19			Actual
27	2016	85.99			Actual
28	2017	85.24			Actual
29	2018	84.45			Actual
30	2019	84.13			Actual
31	2020	84.08			Actual
32	2021	84.14			Actual
33	2022	84.38			Actual
34	2023	83.46			Actual
35	2024	81.08	68.22	84.03	Forecast
36	2025	78.45	67.99	86.36	Forecast
37	2026	76.52	68.35	88.43	Forecast
38	2027	75.76	68.71	89.89	Forecast
39	2028	76.12	68.72	90.66	Forecast
40	2029	77.18	68.26	90.85	Forecast
41	2030	78.39	80.44	81.72	Forecast
42	2031	79.30	76.30	80.60	Forecast

43	2032	79.69	72.38	80.66	Forecast
44	2033	79.56	69.59	81.93	Forecast

Table S21. ARIMA model forecasts of DALYs due to periodontal diseases among males in G20 countries

	Year	Value	Lower	Upper	Type
1	1990	73.40			Actual
2	1991	72.71			Actual
3	1992	72.09			Actual
4	1993	71.60			Actual
5	1994	71.26			Actual
6	1995	71.10			Actual
7	1996	71.27			Actual
8	1997	71.74			Actual
9	1998	72.35			Actual
10	1999	72.91			Actual
11	2000	73.26			Actual
12	2001	73.43			Actual
13	2002	73.57			Actual
14	2003	73.70			Actual
15	2004	73.81			Actual
16	2005	73.89			Actual
17	2006	73.50			Actual
18	2007	72.51			Actual
19	2008	71.32			Actual
20	2009	70.37			Actual
21	2010	70.13			Actual
22	2011	71.37			Actual
23	2012	73.97			Actual
24	2013	77.03			Actual
25	2014	79.63			Actual
26	2015	80.88			Actual
27	2016	80.90			Actual
28	2017	80.62			Actual
29	2018	80.34			Actual
30	2019	80.31			Actual
31	2020	80.26			Actual
32	2021	80.41			Actual
33	2022	80.55			Actual
34	2023	79.72			Actual
35	2024	78.01	67.84	81.06	Forecast
36	2025	76.21	67.23	82.82	Forecast
37	2026	74.89	67.10	84.38	Forecast
38	2027	74.32	67.10	85.57	Forecast
39	2028	74.45	67.00	86.33	Forecast
40	2029	75.03	66.69	86.72	Forecast
41	2030	75.74	77.46	78.55	Forecast

42	2031	76.34	74.43	77.98	Forecast
43	2032	76.67	71.49	78.30	Forecast
44	2033	76.71	69.22	79.43	Forecast

Table S22. ARIMA model forecasts of DALYs due to periodontal diseases among females in G20 countries

	Year	Value	Lower	Upper	Type
1	1990	76.49			Actual
2	1991	75.75			Actual
3	1992	75.07			Actual
4	1993	74.51			Actual
5	1994	74.10			Actual
6	1995	73.90			Actual
7	1996	73.96			Actual
8	1997	74.22			Actual
9	1998	74.57			Actual
10	1999	74.90			Actual
11	2000	75.09			Actual
12	2001	75.30			Actual
13	2002	75.68			Actual
14	2003	76.13			Actual
15	2004	76.53			Actual
16	2005	76.72			Actual
17	2006	76.24			Actual
18	2007	75.03			Actual
19	2008	73.59			Actual
20	2009	72.44			Actual
21	2010	72.10			Actual
22	2011	73.41			Actual
23	2012	76.17			Actual
24	2013	79.41			Actual
25	2014	82.18			Actual
26	2015	83.49			Actual
27	2016	83.41			Actual
28	2017	82.89			Actual
29	2018	82.35			Actual
30	2019	82.18			Actual
31	2020	82.13			Actual
32	2021	82.23			Actual
33	2022	82.42			Actual
34	2023	81.54			Actual
35	2024	79.51	68.05	82.35	Forecast
36	2025	77.29	67.65	84.40	Forecast
37	2026	75.65	67.80	86.23	Forecast
38	2027	74.97	68.04	87.58	Forecast
39	2028	75.20	68.04	88.35	Forecast
40	2029	76.02	67.70	88.64	Forecast

41	2030	77.02	78.93	80.08	Forecast
42	2031	77.81	75.38	79.20	Forecast
43	2032	78.20	71.96	79.35	Forecast
44	2033	78.17	69.43	80.51	Forecast

Table S23. Best-fitting ARIMA model parameters and goodness-of-fit statistics for age-standardized incidence and DALY rates of periodontal diseases in China and G20 countries, by sex, 1990–2023.

Entity	Sex	Outcome	Model (p,d,q)	AIC	BIC	AICc	Ljung-Box p-value	ACF1	MAPE (%)	RMSE
China	Both	ASIR	ARIMA(2,0,2)	231.79	240.95	234.9	0.74	0.09	0.48	5.22
China	Male	ASIR	ARIMA(4,0,0)	231	240.16	234.12	0.83	-0.14	0.47	5.15
China	Female	ASIR	ARIMA(2,1,1)	216.22	222.21	217.65	0.89	0.02	0.43	5.08
G20	Both	ASIR	ARIMA(2,1,1)	188.84	194.82	190.26	0.91	0.02	0.18	3.36
G20	Male	ASIR	ARIMA(2,1,1)	192.23	198.22	193.66	0.999	0.00	0.19	3.56
G20	Female	ASIR	ARIMA(2,1,1)	185.69	191.68	187.12	0.82	0.04	0.17	3.18
China	Both	ASDR	ARIMA(3,1,0)	67.83	73.81	69.26	0.81	0.00	0.64	0.54
China	Male	ASDR	ARIMA(2,1,1)	71.35	77.33	72.77	0.85	0.03	0.69	0.57
China	Female	ASDR	ARIMA(3,1,0)	67.46	73.44	68.89	0.8	-0.03	0.62	0.54
G20	Both	ASDR	ARIMA(2,1,1)	22.85	28.84	24.28	0.93	0.01	0.23	0.28
G20	Male	ASDR	ARIMA(2,1,1)	30.39	36.38	31.82	0.95	-0.01	0.24	0.31
G20	Female	ASDR	ARIMA(2,1,1)	18.69	24.68	20.12	0.89	0.02	0.24	0.26

Table S24. The RECORD statement – checklist of items, extended from the STROBE statement, that should be reported in observational studies using routinely collected health data.

	Item No.	STROBE items	Location in manuscript where items are reported	RECORD items	Location in manuscript where items are reported
**Title and abstract**					
	1	(a) Indicate the study's design with a commonly used term in the title or the abstract (b) Provide in the abstract an informative and balanced summary of what was done and what was found	Title, Abstract	RECORD 1.1: The type of data used should be specified in the title or abstract. When possible, the name of the databases used should be included.<br><br>RECORD 1.2: If applicable, the geographic region and timeframe within which the study took place should be reported in the title or abstract.<br><br>RECORD 1.3: If linkage between databases was conducted for the study, this should be clearly stated in the title or abstract.	Abstract: "using Global Burden of Disease 2023 data"<br><br>Title & Abstract: "China and G20 nations, 1990–2023 "<br><br>Not applicable (single database)
**Introduction**					
Background rationale	2	Explain the scientific background and rationale for the investigation being reported	Introduction, paragraphs 1-2		
Objectives	3	State specific objectives, including any prespecified hypotheses	Introduction, paragraph 4		
**Methods**					
Study	4	Present key elements	Method		

Design		of study design early in the paper	s, line 1: "large-scale descriptive epidemiological analysis"		
Setting	5	Describe the setting, locations, and relevant dates, including periods of recruitment, exposure, follow-up, and data collection	Methods 2.1: "1990-2023", G20 countries including China		
Participants	6	*(a) Cohort study* - Give the eligibility criteria, and the sources and methods of selection of participants. Describe methods of follow-up<br>*Case-control study* - Give the eligibility criteria, and the sources and methods of case ascertainment and control selection. Give the rationale for the choice of cases and controls<br>*Cross-sectional study* - Give the eligibility criteria, and the sources and methods of selection of participants<br><br>*(b) Cohort study* - For matched studies,	Methods 2.1: inclusion/exclusion criteria for G20 entities	RECORD 6.1: The methods of study population selection (such as codes or algorithms used to identify subjects) should be listed in detail. If this is not possible, an explanation should be provided.<br><br>RECORD 6.2: Any validation studies of the codes or algorithms used to select the population should be referenced. If validation was conducted for this study and not published elsewhere, detailed methods and results should be provided.<br><br>RECORD 6.3: If the study involved linkage of databases, consider use	Methods 2.1: "Community Periodontal Index of Treatment Needs (CPITN) Class IV, attachment loss >6 mm, or pocket depth >5 mm" Methods 2.1: "harmonized with reference definitions via MR-BRT meta-regression" and

		give matching criteria and number of exposed and unexposed<br>*Case-control study* - For matched studies, give matching criteria and the number of controls per case		of a flow diagram or other graphical display to demonstrate the data linkage process, including the number of individuals with linked data at each stage.	reference 14, 15<br>Not applicable (no linkage)
Variables	7	Clearly define all outcomes, exposures, predictors, potential confounders, and effect modifiers. Give diagnostic criteria, if applicable.	Methods 2.1: incidence, DALYs; 2.2: decomposition components	RECORD 7.1: A complete list of codes and algorithms used to classify exposures, outcomes, confounders, and effect modifiers should be provided. If these cannot be reported, an explanation should be provided.	Methods 2.1: ICD-9 523.0–523.9, ICD-10 K05.0–K05.6; CPITN Class IV, AL>6mm, PD>5mm
Data sources/ measurement	8	For each variable of interest, give sources of data and details of methods of assessment (measurement). Describe comparability of assessment methods if there is more than one group	Methods 2.1: "Data obtained from GBD 2023 repository via GHDx"		
Bias	9	Describe any efforts to address potential sources of bias	Discussion, Limitations paragraph; also Methods 2.1 (MR-BRT harmonization)		
Study size	10	Explain how the study size was arrived at	Not applicable (all available data from GBD 2023)		

Quantitative variables	11	Explain how quantitative variables were handled in the analyses. If applicable, describe which groupings were chosen, and why	Methods 2.2: Joinpoint (log-linear), decomposition (Das Gupta), ARIMA (stationarity, ACF/PACF)		
Statistical methods	12	(a) Describe all statistical methods, including those used to control for confounding<br>(b) Describe any methods used to examine subgroups and interactions<br>(c) Explain how missing data were addressed<br>(d) *Cohort study* - If applicable, explain how loss to follow-up was addressed<br>*Case-control study* - If applicable, explain how matching of cases and controls was addressed<br>*Cross-sectional study* - If applicable, describe analytical methods taking account of sampling strategy<br>(e) Describe any sensitivity analyses	Methods 2.2: Joinpoint, decomposition, ARIMA. Subgroups by sex. Missing data not applicable (GBD complete estimates). No loss to follow-up. Sensitivity not performed.		
Data access and cleaning methods		..		RECORD 12.1: Authors should describe the extent to which the investigators had access to the database	Methods 2.2: "All GBD 2023 estimates are

				population used to create the study population.<br><br>RECORD 12.2: Authors should provide information on the data cleaning methods used in the study.	produced using DisMod-MR 2.1" – authors accessed aggregated estimates, not individual-level data Not applicable (aggregated estimates used)
Linkage		..		RECORD 12.3: State whether the study included person-level, institutional-level, or other data linkage across two or more databases. The methods of linkage and methods of linkage quality evaluation should be provided.	Not applicable (single database)
**Results**					
Participants	13	(a) Report the numbers of individuals at each stage of the study (*e.g.*, numbers potentially eligible, examined for eligibility, confirmed eligible, included in the study, completing follow-up, and analysed)<br>(b) Give reasons for non-participation at each stage.<br>(c) Consider use of a	Not applicable (no participant recruitment)	RECORD 13.1: Describe in detail the selection of the persons included in the study (*i.e.,* study population selection) including filtering based on data quality, data availability and linkage. The selection of included persons can be described in the text and/or by means of the study flow diagram.	Not applicable (population-level aggregated data)

		flow diagram			
Descriptive data	14	(a) Give characteristics of study participants (*e.g.*, demographic, clinical, social) and information on exposures and potential confounders<br>(b) Indicate the number of participants with missing data for each variable of interest<br>(c) *Cohort study* - summarise follow-up time (*e.g.*, average and total amount)	Not applicable		
Outcome data	15	*Cohort study* - Report numbers of outcome events or summary measures over time<br>*Case-control study* - Report numbers in each exposure category, or summary measures of exposure<br>*Cross-sectional study* - Report numbers of outcome events or summary measures	Results 3.1–3.5; Tables S1–S25; Figures 1–5		
Main results	16	(a) Give unadjusted estimates and, if applicable, confounder-adjusted estimates and their precision (e.g., 95% confidence interval). Make clear which confounders were adjusted for and why	Results 3.3 (APC, AAPC with 95% CI); 3.4 (decomposition percentages)		

		they were included<br>(b) Report category boundaries when continuous variables were categorized<br>(c) If relevant, consider translating estimates of relative risk into absolute risk for a meaningful time period			
Other analyses	17	Report other analyses done—e.g., analyses of subgroups and interactions, and sensitivity analyses	Results by sex throughout; no sensitivity analyses		
**Discussion**					
Key results	18	Summarise key results with reference to study objectives	Discussion, first paragraph		
Limitation s	19	Discuss limitations of the study, taking into account sources of potential bias or imprecision. Discuss both direction and magnitude of any potential bias	Discussion, final paragraph (Limitations)	RECORD 19.1: Discuss the implications of using data that were not created or collected to answer the specific research question(s). Include discussion of misclassification bias, unmeasured confounding, missing data, and changing eligibility over time, as they pertain to the study being reported.	Discussion , Limitation s: "case definition.. .may not fully capture clinical severity"; "rural-urba n disparity... unmeasure d confounder "; "diagnostic criteria have evolved...r esidual bias"

					(Methods 2.1); "COVID-19 disruption"
Interpretation	20	Give a cautious overall interpretation of results considering objectives, limitations, multiplicity of analyses, results from similar studies, and other relevant evidence	Discussion, final paragraph of each subsection and Conclusion		
Generalisability	21	Discuss the generalisability (external validity) of the study results	Discussion: comparisons between China and G20, applicability to other settings		
**Other Information**					
Funding	22	Give the source of funding and the role of the funders for the present study and, if applicable, for the original study on which the present article is based	Funding section (after Author Contributions)		
Accessibility of protocol, raw data, and programming code		..		RECORD 22.1: Authors should provide information on how to access any supplemental information such as the study protocol, raw data, or programming code.	Data availability statement: "publicly available through GHDx"

*Reference: Benchimol EI, Smeeth L, Guttmann A, Harron K, Moher D, Petersen I, Sørensen HT, von Elm E, Langan SM, the RECORD Working Committee. The REporting of studies Conducted using Observational Routinely-collected health Data (RECORD) Statement. *PLoS Medicine* 2015; in press.

*Checklist is protected under Creative Commons Attribution (CC BY) license.

Table S25. STROBE Statement—checklist of items that should be included in reports of observational studies

	Item No.	Recommendation	Page No.	Relevant text from manuscript
**Title and abstract**	1	(*a*) Indicate the study's design with a commonly used term in the title or the abstract	Title; Abstract	Title: "Insights from the Global Burden of Disease 2023 Study" (descriptive epidemiological analysis). Abstract: "using Global Burden of Disease 2023 data"
		(*b*) Provide in the abstract an informative and balanced summary of what was done and what was found	Abstract	Abstract (Aims, Methods, Results, Conclusion) – describes comparison of burden, joinpoint regression, decomposition analysis, ARIMA forecasting
**Introduction**				
Background/rationale	2	Explain the scientific background and rationale for the investigation being reported	Introduction, paragraphs 1-3	Periodontal disease is a chronic inflammatory condition...", "A comparative study between China and G20 countries holds significant public health relevance..."
Objectives	3	State specific objectives, including any prespecified hypotheses	Introduction, paragraph 4	"Therefore, the specific aims of this study are to compare the periodontal disease burden

				between China and G20 countries from 1990 to 2023, to identify temporal trends and joinpoints using Joinpoint regression, to quantify the demographic drivers through decomposition analysis, and to project the burden to 2033 using ARIMA modeling."
**Methods**				
Study design	4	Present key elements of study design early in the paper	Methods 2.1, first sentence	"This study is a large-scale descriptive epidemiological analysis based on the GBD 2023 dataset, constituting a specific sub-analysis focused on Periodontal Disease across G20 nations."
Setting	5	Describe the setting, locations, and relevant dates, including periods of recruitment, exposure, follow-up, and data collection	Methods 2.1	"Data were obtained from the GBD 2023 repository...", "Inclusion criteria for years: The full time range 1990-2023 was included."; locations: G20 countries including China
Participants	6	(*a*) *Cohort study*—Give the eligibility criteria, and the sources and methods of selection of participants. Describe methods of follow-up<br>*Case-control study*—Give the eligibility criteria, and the sources and methods of case	Methods 2.1	"Inclusion criteria for countries/entities: All 20 member entities of the G20

		ascertainment and control selection. Give the rationale for the choice of cases and controls<br>*Cross-sectional study*—Give the eligibility criteria, and the sources and methods of selection of participants		were included: the 19 sovereign nations... and the European Union... Exclusion criteria: No entities were excluded."
		(*b*) *Cohort study*—For matched studies, give matching criteria and number of exposed and unexposed<br>*Case-control study*—For matched studies, give matching criteria and the number of controls per case	Not applicable	No matching performed
Variables	7	Clearly define all outcomes, exposures, predictors, potential confounders, and effect modifiers. Give diagnostic criteria, if applicable	Methods 2.1	"In the GBD 2023 framework, 'periodontal disease' refers specifically to severe periodontitis. The reference case definition is: Community Periodontal Index of Treatment Needs (CPITN) Class IV, attachment loss (AL) > 6 mm, or gingival pocket depth (PD) > 5 mm. These definitions correspond to ICD-9 codes 523.0–523.9 and ICD-10 codes K05.0–K05.6."
Data sources/ measurement	8*	For each variable of interest, give sources of data and details of methods of assessment (measurement). Describe comparability of assessment methods if there is more than one group	Methods 2.1	"Data were obtained from the GBD 2023 repository via the Global Health Data Exchange (GHDx; http://ghdx.healthdata.org)... To

				maximize data inclusion, severe periodontitis cases identified using alternative criteria were harmonized with the reference definitions via the MR-BRT meta-regression approach."
Bias	9	Describe any efforts to address potential sources of bias	Methods 2.1; Discussion, Limitations	"The GBD 2023 study attempts to address this by applying a consistent case definition across all years and countries through its modeling framework, and by using MR-BRT to harmonize data from different criteria."; also discussion of detection bias, rural-urban disparity, and COVID-19 disruption in Limitations
Study size	10	Explain how the study size was arrived at	Not applicable	No sample size calculation; all available GBD 2023 data for G20 countries from 1990-2023 were used

Continued on next page

Quantitative variables	11	Explain how quantitative variables were handled in the analyses. If applicable, describe which groupings were chosen and why	Methods 2.2	"Joinpoint regression... models trends by fitting a segmented log-linear function... Decomposition analysis... ARIMA model... Age groups as per GBD 2023 global standard population: 0–4, 5–9, ..., 85+ years."
Statistical methods	12	(*a*) Describe all statistical methods, including those used to control for confounding	Methods 2.2	"Joinpoint regression... Decomposition analysis... ARIMA modeling... All GBD 2023 estimates are produced using DisMod-MR 2.1." No confounding adjustment (descriptive study).
		(*b*) Describe any methods used to examine subgroups and interactions	Methods 2.2; Results	Analyses by sex (male/female) and age groups. See Figures 2, 3, 5 and Tables S3-S6, S11-S22.
		(*c*) Explain how missing data were addressed	Not applicable	GBD 2023 provides complete estimates; no missing data.
		(*d*) *Cohort study*—If applicable, explain how loss to follow-up was addressed<br>*Case-control study*—If applicable, explain how matching of cases and controls was addressed<br>*Cross-sectional study*—If applicable, describe analytical methods taking account of sampling strategy	Not applicable	No loss to follow-up, no matching, cross-sectional analysis of aggregated data.
		(*e*) Describe any sensitivity analyses	Discussion, Limitations	"No sensitivity analyses performed" (implicitly acknowledged).
**Results**				

Participants	13*	(a) Report numbers of individuals at each stage of study—eg numbers potentially eligible, examined for eligibility, confirmed eligible, included in the study, completing follow-up, and analysed	Not applicable	Population-level aggregated data; no individual participant recruitment.
		(b) Give reasons for non-participation at each stage	Not applicable	Not applicable.
		(c) Consider use of a flow diagram	Not applicable	Not applicable.
Descriptive data	14*	(a) Give characteristics of study participants (eg demographic, clinical, social) and information on exposures and potential confounders	Results 3.2; Tables S3-S6	Age-specific incidence and DALY rates by sex for China and G20 average.
		(b) Indicate number of participants with missing data for each variable of interest	Not applicable	No missing data.
		(c) *Cohort study*—Summarise follow-up time (eg, average and total amount)	Not applicable	Not applicable.
Outcome data	15*	*Cohort study*—Report numbers of outcome events or summary measures over time	Not applicable (not a cohort study)	
		Case-control study—Report numbers in each exposure category, or summary measures of exposure	Not applicable (not a cohort study)	
		Cross-sectional study—Report numbers of outcome events or summary measures	Results 3.1-3.5; Figures 1-5; Tables	Incidence and DALY rates (age-standardized and age-specific) for each G20 country and China, 1990-2023, with projections to

			S1-S25	2033. For example: "In 2023, Brazil (1157.38 per 100,000), Turkey (1164 per 100,000), and China (950.20 per 100,000) had the highest incidence rates" (Results 3.1). "For DALY rates in China, the peak occurred at 55–59 years, with male rates consistently higher than female rates across all ages" (Results 3.2).
Main results	16	(*a*) Give unadjusted estimates and, if applicable, confounder-adjusted estimates and their precision (eg, 95% confidence interval). Make clear which confounders were adjusted for and why they were included	Results 3.3; Results 3.4	Joinpoint regression: APC and AAPC with 95% CI. Decomposition: percentage contributions (no adjustment).
		(*b*) Report category boundaries when continuous variables were categorized	Methods 2.1	Outcome data
		(*c*) If relevant, consider translating estimates of relative risk into absolute risk for a meaningful time period	Not applicable	No relative risk estimates.

Continued on next page

Other analyses	17	Report other analyses done—eg analyses of subgroups and interactions, and sensitivity analyses	Results by sex throughout	Subgroup analyses by sex presented in Figure 5 and corresponding supplementary tables. No sensitivity analyses.
**Discussion**				
Key results	18	Summarise key results with reference to study objectives	Discussion, first paragraph	"Our study reveals three principal findings: (1) substantial heterogeneity in periodontal disease burden across G20 nations, with China occupying an intermediate-to-high burden position; (2) fundamentally different demographic drivers, with aging dominating in China and population growth in G20 countries; and (3) projected divergence, with China's DALY rates potentially stabilizing while G20 rates remain persistently higher."
Limitations	19	Discuss limitations of the study, taking into account sources of potential bias or imprecision. Discuss both direction and magnitude of any potential bias	Discussion, final paragraph (Limitations)	"First, the case definition of periodontal disease in the GBD framework primarily relies on population-based surveys and models... Second, while our analysis identified demographic

				drivers, it did not incorporate specific behavioral, socioeconomic, or healthcare access variables... Third, the forecasting analysis using ARIMA models is based solely on extrapolation... Finally, our study period includes the initial phase of the COVID-19 pandemic..."
Interpretation	20	Give a cautious overall interpretation of results considering objectives, limitations, multiplicity of analyses, results from similar studies, and other relevant evidence	Discussion, final paragraph of each subsection and Conclusion	Throughout Discussion: "should be interpreted with caution", "cannot establish causal mechanisms", "these interpretations should be viewed as hypotheses". Conclusion states tailored strategies needed.
Generalisability	21	Discuss the generalisability (external validity) of the study results	Discussion	Comparisons between China and G20 countries; implications for other middle-income and high-income nations.
**Other information**				
Funding	22	Give the source of funding and the role of the funders for the present study and, if applicable, for the original study on which the present article is based	Funding section (after Author Contributions)	"This study was supported by the Shandong Provincial Natural Science Foundation (No. ZR2025QC908) and the National Traditional Chinese Medicine

	Comprehensive Reform Demonstration Zone Science and Technology Co-construction Project (No. GZY-KJS-SD-2024-106). The funders had no role in study design, data collection, analysis, interpretation, manuscript writing, or the decision to submit for publication."

*Give information separately for cases and controls in case-control studies and, if applicable, for exposed and unexposed groups in cohort and cross-sectional studies.

Note: An Explanation and Elaboration article discusses each checklist item and gives methodological background and published examples of transparent reporting. The STROBE checklist is best used in conjunction with this article (freely available on the Web sites of PLoS Medicine at http://www.plosmedicine.org/, Annals of Internal Medicine at http://www.annals.org/, and Epidemiology at http://www.epidem.com/). Information on the STROBE Initiative is available at www.strobe-statement.org.
